# Cladribine Analogues via *O*^6^-(Benzotriazolyl) Derivatives of Guanine Nucleosides

**DOI:** 10.3390/molecules201018437

**Published:** 2015-10-09

**Authors:** Sakilam Satishkumar, Prasanna K. Vuram, Siva Subrahmanyam Relangi, Venkateshwarlu Gurram, Hong Zhou, Robert J. Kreitman, Michelle M. Martínez Montemayor, Lijia Yang, Muralidharan Kaliyaperumal, Somesh Sharma, Narender Pottabathini, Mahesh K. Lakshman

**Affiliations:** 1 Department of Chemistry, The City College and The City University of New York, 160 Convent Avenue, New York, NY 10031, USA; E-Mails: ssakilam@ccny.cuny.edu (S.S.); vkumar@ccny.cuny.edu (P.K.V.); lyang@sci.ccny.cuny.edu (L.Y.); 2 Discovery and Analytical Services, GVK Biosciences Pvt. Ltd., 28A IDA Nacharam, Hyderabad 500076, India; E-Mails: siva.relangi@gvkbio.com (S.S.R.); venkateshwarlu.gurram@gvkbio.com (V.G.); muralidharan.k@gvkbio.com (M.K.); somesh.sharma@gvkbio.com (S.S.); narender.pottabathini@gvkbio.com (N.P.); 3 Clinical Immunotherapy Section, Laboratory of Molecular Biology, National Cancer Institute, National Institutes of Health, 9000 Rockville Pike, Bethesda, MD 20892, USA; E-Mails: hong.zhou@nih.gov (H.Z.); kreitmar@mail.nih.gov (R.J.K.); 4 Department of Biochemistry, Universidad Central del Caribe-School of Medicine, P. O. Box 60327, Bayamón, PR 00960, USA; E-Mail: mmmtz92@gmail.com

**Keywords:** cladribine, nucleoside, guanosine, benzotriazole, (benzotriazol-1yl-oxy)-tris(dimethylamino)phosphonium hexafluorophosphate, BOP

## Abstract

Cladribine, 2-chloro-2′-deoxyadenosine, is a highly efficacious, clinically used nucleoside for the treatment of hairy cell leukemia. It is also being evaluated against other lymphoid malignancies and has been a molecule of interest for well over half a century. In continuation of our interest in the amide bond-activation in purine nucleosides via the use of (benzotriazol-1yl-oxy)tris(dimethylamino)phosphonium hexafluorophosphate, we have evaluated the use of *O*^6^-(benzotriazol-1-yl)-2′-deoxyguanosine as a potential precursor to cladribine and its analogues. These compounds, after appropriate deprotection, were assessed for their biological activities, and the data are presented herein. Against hairy cell leukemia (HCL), T-cell lymphoma (TCL) and chronic lymphocytic leukemia (CLL), cladribine was the most active against all. The bromo analogue of cladribine showed comparable activity to the ribose analogue of cladribine against HCL, but was more active against TCL and CLL. The bromo ribose analogue of cladribine showed activity, but was the least active among the C6-NH_2_-containing compounds. Substitution with alkyl groups at the exocyclic amino group appears detrimental to activity, and only the C6 piperidinyl cladribine analogue demonstrated any activity. Against adenocarcinoma MDA-MB-231 cells, cladribine and its ribose analogue were most active.

## 1. Introduction

Cladribine, 2-chloro-2′-deoxyadenosine, has been a molecule of interest for well over five decades. The history of this compound dates back to 1960, when it was used in the synthesis of 2′-deoxyguanosine and 2′-deoxyinosine [1]. A decade later, cladribine was shown to be a poor substrate for adenosine deaminase that underwent phosphorylation by deoxycytidine kinase, finally resulting in the triphosphate, and inhibiting DNA synthesis rather than RNA synthesis [2,3]. Later still, it was shown to be a substrate for deoxyguanosine kinase, which is responsible for phosphorylation of purine nucleosides in mitochondria [3]. Several mechanisms have been proposed by which cladribine can cause mitochondrial damage and apoptotic cell death [4,5,6,7,8].

In contemporary medicine, cladribine is used in the treatment of lymphoid malignancies, most notably for its efficacy against hairy cell leukemia [9]. Cladribine is also being evaluated against several other indolent lymphoid malignancies, also in combination with other drug candidates [10,11].

The synthesis of cladribine has primarily relied on three major methods: (a) glycosylation reactions of a nucleobase with a sugar [12,13,14,15,16,17,18], and its variations; (b) deoxygenation of the C2′ hydroxyl group of a suitable nucleoside derivative [12,15,19,20]; (c) enzymatic glycosyl transfer reactions [21,22,23,24]; and (d) conversion of readily available nucleoside precursors (some utilizing nucleosides for glycosyl transfer reactions) [21,22,24,25,26]. Each of these methods has been used with varying levels of convenience and success. Among the many approaches, one convenient method is the selective displacement of a leaving group (chloride or aryl sulfonate) from the C6 position of a suitable purine nucleoside precursor. Despite the availability of this selective S_N_Ar displacement, we find that no other N6-substituted cladribine analogues have been synthesized by such a method. Because of our interest in broadening the utilities of *O*^6^-(benzotriazol-1-yl)purine nucleoside derivatives, we elected to evaluate the synthesis of N6-substituted cladribine analogues via amide-bond activation of guanine nucleosides with (benzotriazol-1yl-oxy)tris(dimethylamino)phosphonium hexafluorophosphate (BOP).

## 2. Results and Discussion

For the modification of the nucleobases of inosine, and 2′-deoxyinosine (3 examples), BOP had been used for *in situ* activation of the amide moieties in these substrates, followed by S_N_Ar displacement with amines [27,28]. It was proposed that reaction of the amide group with BOP proceeded via a phosphonium intermediate, which could be directly captured by a reactive amine [27]. On the other hand, with less reactive amines, the *O*-(benzotriazol-1-yl) intermediate can be formed by competitive capture of the phosphonium intermediate by the benzotriazol-1-yloxy anion [27].

In 2007, we first reported the isolation of *O*^6^-(benzotriazol-1-yl)inosine and -2′-deoxyinosine by amide-bond activation with BOP, in the absence of a nucleophile [29], an observation that was later reconfirmed by others [30,31]. These electrophilic nucleosides, which are stable to storage, are exceptionally good partners in S_N_Ar reactions with oxygen, nitrogen, and sulfur nucleophiles [29]. Subsequently, we demonstrated that *O*^6^-(benzotriazol-1-yl)inosine and -2′-deoxyinosine can also be prepared via the use of PPh_3_/I_2_ and 1-hydroxybenzotriazole [32]. The amide-bond activation protocol was then modified to tether the *O*^6^-(benzotriazol-1-yl) nucleosides onto a polymer support for high-throughput type of applications [33]. Interestingly, the amide bond activation when applied to the urea functionality of *O*^6^-benzyl-protected 2′-deoxyxanthosine did not yield the *O*^2^-(benzotriazol-1-yl) derivative but rather terminated in an isolable and synthetically useful phosphonium salt [34]. We also showed that guanosine and 2′-deoxyguanosine undergo facile reactions with BOP, and that *O*^6^-(benzotriazol-1-yl) guanine nucleosides are effective substrates for S_N_Ar reactions as well [35]. In the combined course of these investigations we had ascertained plausible operative mechanisms of these amide-activation reactions, results that were later applied to a one-pot etherification protocol for purine nucleosides and pyrimidines [36].

Considering only the nucleoside modification literature, our BOP-mediated amide-activation methodology has found wide application [37,38,39,40,41,42,43]. Of specific interest to our current work was a report wherein some of our results on guanosine derivatives were reevaluated [44]. In addition to 2′,3′,5′-tri-*O*-(*t*-butyldimethylsilyl)guanosine, which we had originally investigated, five other compounds were also studied: unprotected 2′-deoxyguanosine, its 2′,3′,5′-triacetyl and the 2′,3′-isopropylidene derivatives, and two 2′,3′-isopropylidiene 5′-carboxamides [44]. These products were subsequently tested in diazotization-halogenation reactions, *en route* to 2-chloro and 2-iodo adenosine analogues [44]. 2,6-Dihalopurine ribonucleosides are more readily accessible for this purpose, as compared to 2′-deoxy derivatives. Thus, we were intrigued by the fact that diazotization-halogenation reactions of *O*^6^-(benzotriazol-1yl)-2′-deoxyguanosine have not been reported. This prompted use to investigate the chlorination and bromination of this compound, and in this context we decided to evaluate the synthesis of cladribine and other cladribine analogues via such an approach.

**Scheme 1:**
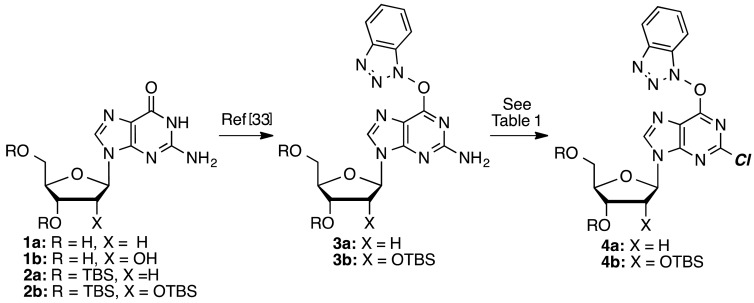
Preparation of *O*^6^-(benzotriazol-1-yl) guanosine derivatives and the chlorination reaction.

In 2001, diazotization reactions leading to cladribine and other halo derivatives have been performed on unprotected 2,6-diaminopurine 2′-deoxyribonucleoside [45]. However, no substituents other than NH_2_ were introduced into the C6 position, and the synthesis of the precursor is not particularly convenient. For the present study, we anticipated the need for saccharide protection, and both acetyl and *t*-butyldimethysilyl (TBS) protecting groups were considered. Between these, TBS was selected because acetyl groups can be susceptible to cleavage with amines, which could be a complicating problem in the method development stage. Thus, nucleosides **1a** and **1b** were silylated to give the corresponding products **2a** and **2b**, which were converted to the *O*^6^-(benzotriazol-1-yl) guanosine derivatives **3a** and **3b**, respectively (Scheme 1).

We opted for diazotization-chlorination conditions using *t*-BuONO/TMSCl, a reagent combination initially introduced for nucleoside modification in 2003 [46]. We [47] and others [44] have previously used non-aqueous conditions for halogenation at the C2 position of purine nucleoside derivatives. Under these conditions, reactions of substrate **3a** proceeded modestly. However, the obtained product was contaminated with ~30% of the C2 protio *O*^6^-(benzotriazol-1-yl)-3′,5′-di-*O*-(*t*-butyldimethylsilyl)-2′-deoxyinosine (Table 1, entries 1 and 2). With the combination of *t*-BuONO/TMSCl/(BnNEt_3_)^+^Cl^−^, no C2 protio product was observed, but only a low product yield was obtained (entry 3).

**Table 1:** Evaluation of conditions for the diazotization/chlorination of *O*^6^-(benzotriazol-1-yl)-3′,5′-di-*O*-(*t*-butyldimethylsilyl)-2′-deoxyguanosine.

Entry	Conditions	Scale	Yield
1	*t*-BuONO (2.6 equiv), TMSCl (2.6 equiv), CH_2_Cl_2_, −10 °C to 0 °C, 2 h	73 μmol of **3a**	43% ^a^
2	*t*-BuONO (10 equiv), TMSCl (5 equiv), CH_2_Cl_2_, 0 °C, 1 h	73 μmol of **3a**	58% ^a^
3	*t*-BuONO (10 equiv), TMSCl (0.4 equiv), (BnNEt_3_)^+^Cl^−^ (10 equiv), CH_2_Cl_2_, −10 °C to 0 °C, 2 h	49 μmol of **3a**	26%
4	*t*-BuONO (3.5 equiv), SbCl_3_ (1.4 equiv), ClCH_2_CH_2_Cl, −10 °C to −15 °C, 1.5 h	49 μmol of **3a**	35%
5	*t*-BuONO (10 equiv), (BnNEt_3_)^+^Cl^−^ (10 equiv), CH_2_Cl_2_, −78 °C and then rt, 1 h	49 μmol of **3a**	39%
6	*t*-BuONO (20 equiv), SbCl_3_ (0.4 equiv), (BnNEt_3_)^+^Cl^−^ (20 equiv), CH_2_Cl_2_, −10 °C to 0 °C, 5 h	49 μmol of **3a**	39%
7	*t*-BuONO (10 equiv), (BnNEt_3_)^+^Cl^−^ (10 equiv), (Me_3_Si)_2_NH (10 equiv), CH_2_Cl_2_, −10 °C to 0 °C, 4 h	49 μmol of **3a**	35%
8	*t*-BuONO (3.5 equiv), SbCl_3_ (1.4 equiv), CH_2_Cl_2_, −10 °C, 2 h	49 μmol of **3a**	39% ^b^
9	*t*-BuONO (3.5 equiv), SbCl_3_ (1.4 equiv), CH_2_Cl_2_, −10 °C to −15 °C, 2 h	73 μmol of **3a**	51%
10	*t*-BuONO (3.5 equiv), SbCl_3_ (1.4 equiv), CH_2_Cl_2_, −10 °C, 2 h	0.14 mmol of **3a**	60%
11	*t*-BuONO (3.5 equiv), SbCl_3_ (1.4 equiv), CH_2_Cl_2_, −10 °C to −15 °C, 2 h	1.6 mmol of **3a**	65%
12	*t*-BuONO (3.5 equiv), SbCl_3_ (1.4 equiv), CH_2_Cl_2_, −10 °C to −15 °C, 3.5 h	0.27 mmol of **3b**	61%
13	*t*-BuONO (10 equiv), TMSCl (5 equiv), CH_2_Cl_2_, 0 °C, 1 h	2.69 mmol of **3b**	68%

^a^ Yield was calculated on the basis of the molecular weight of compound **4a**. However, by ^1^H-NMR (500 MHz, CDCl_3_), the chromatographically homogenous product band was observed to contain a 2:1 ratio of compound **4a** and the C2 protio *O*^6^-(benzotriazol-1-yl)-3′,5′-di-*O*-(*t*-butyldimethylsilyl)-2′-deoxyinosine. In addition to these, a minor uncharacterized nucleoside byproduct was also formed; ^b^ Compound **3a** dissolved in CH_2_Cl_2_ was added to the mixture of reagents in CH_2_Cl_2_.

These results compelled us to consider other conditions. SbCl_3_ and SbBr_3_ have previously been used for the halogenation of nucleosides [48,49] Thus, the next series of experiments involved SbCl_3_, (BnNEt_3_)^+^Cl^−^, and combinations of these reagents (entries 4–11). Whereas most experiments yielded only 35%–39% of product **4a**, a reasonable yield improvement was observed in entry 9. It was noted that efficient filtration of the reaction mixture after workup is critical to obtaining a good product recovery due to the pasty nature of the mixture (see the Experimental Section). On larger scales, better product recoveries were observed (entries 10 and 11). By comparison, diazotization/chlorination of the ribose derivative **3b** proceeded well with both *t*-BuONO/SbCl_3_ (entry 12) and *t*-BuONO/TMSCl (entry 13), with the latter providing a better yield of compound **4b**. No C2 protio product was apparent in the reactions of precursor **3b**.

**Figure 1:**
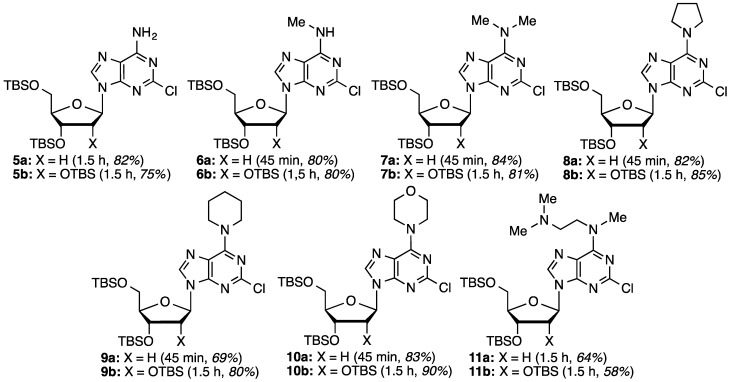
Structures of the products, reaction times, and yields obtained in the S_N_Ar reactions.

The next stage in the chemistry involved S_N_Ar reactions at the C6 positions of substrates **4a** and **4b**. Cladribine and its ribose analogue were prepared by reactions with aqueous ammonia (see the Experimental Section for details). In order to prepare other analogues, reactions were conducted with 1.5 equiv each of methylamine, dimethylamine, pyrrolidine, piperidine, morpholine, and *N*,*N*,*N′*-trimethylethylenediamine (products, reaction times, and yields are shown in Figure 1).

Most reactions proceeded smoothly and in good to high yields. Methylamine (2 M in THF) was used for the synthesis of **6a** and **6b**, whereas a 40% aqueous solution of dimethylamine was used for the synthesis of **7a** and **7b**. As we have previously shown, water is not generally detrimental to reactions of these benzotriazolyl purine nucleosides [35]. In reactions of **4a** and **4b** with *N*,*N*,*N′*-tri-methylethylenediamine, yields were lower. In each ~10%–15% of compounds **7a** and **7b** were isolated as byproducts. The source of dimethylamine is currently unknown but its origin can be linked to *N*,*N*,*N′*-trimethylethylenediamine.

**Scheme 2:**
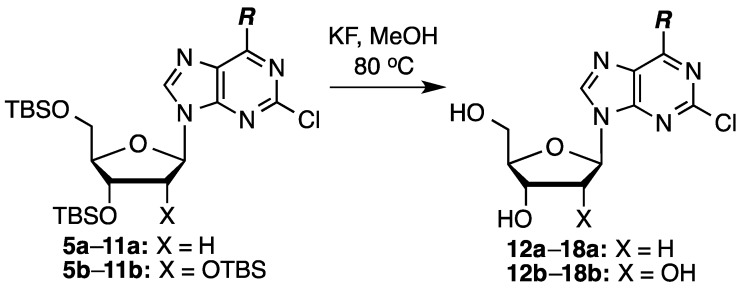
Desilylation of the protected nucleosides.

Finally, for biological testing, desilylation was performed. Because we did not anticipate decomposition of starting materials or products, we only tested the use of KF (2 equiv/silyl group) in MeOH at 80 °C (Scheme 2). The results of the desilylation reactions are shown in Table 2.

**Table 2:** Structures of the products, reaction times, and yields of the desilylated compounds.

Entry	*R*	Reaction Times/KF Equiv	Yields
1	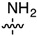	24 h with 4 equiv KF	**12a**: 72%
2		24 h with 6 equiv KF	**12b**: 74%
3	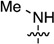	24 h with 4 equiv KF	**13a**: 85%
4		16 h with 6 equiv KF	**13b**: 75%
5	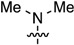	24 h with 4 equiv KF	**14a**: 82%
6		16 h with 6 equiv KF	**14b**: 75%
7	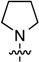	20 h with 4 equiv KF	**15a**: 81%
8		16 h with 6 equiv KF	**15b**: 85%
9	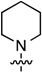	24 h with 4 equiv KF	**16a**: 71%
10		16 h with 6 equiv KF	**16b**: 84%
11	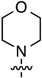	26 h with 4 equiv KF	**17a**: 74%
12		16 h with 6 equiv KF	**17b**: 78%
13	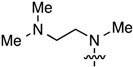	24 h with 4 equiv KF	**18a**: 84%
14		24 h with 6 equiv KF	**18b**: 65%

Because there is currently no known method for the diazotization/bromination of compound **3a**, we investigated a route similar to that for chlorination. Results from these experiments are listed in Table 3. What is notable with the diazotization/bromination, in contrast to the chlorination, was that use of 3.5 equiv of *t*-BuONO led to incomplete conversion over 2 h at −10 to −15 °C. Addition of another 3.5 equiv of *t*-BuONO then led to complete conversion over an additional 1 h.

**Scheme 3:**
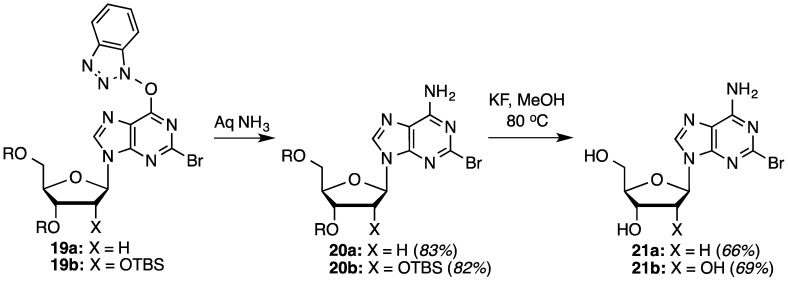
Synthesis of 2-bromo-2′-deoxyadenosine.

A similar conversion of **3b** led to the ribose analogue **19b** in a comparable 64% yield (Table 3, entry 3). Compound **19a** was then converted to the bromo analogue of cladribine as shown in Scheme 3. S_N_Ar displacement with aqueous NH_3_ proceeded in 83% yield producing compound **20a**, which was finally desilylated with KF in anhydrous MeOH at 80 °C (28 h) to yield 2-bromo-2-deoxyadenosine (**21a**) in 66% yield. Corresponding conversions of the ribose analog **19b**, via intermediate **20b**, gave compound **21b**. Yields for these conversions were comparable to the deoxyribose series.

**Table 3:** Diazotization-bromination of silyl-protected *O*^6^-(benzotriazol-1-yl) guanine nucleosides. 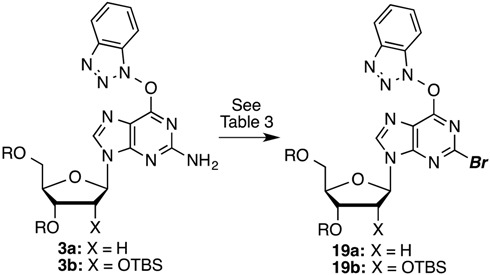

Entry	Conditions	Scale	Yield
1	*t*-BuONO (7.0 equiv), SbBr_3_ (1.4 equiv), CH_2_Br_2_, −10 °C to −15 °C, 3 h	73 μmol of **3a**	69%
2	*t*-BuONO (7.0 equiv), SbBr_3_ (1.4 equiv), CH_2_Br_2_, −10 °C to −15 °C, 3 h	0.49 mmol of **3a**	63%
3	*t*-BuONO (7.0 equiv), SbBr_3_ (1.4 equiv), CH_2_Br_2_, −10 °C to −15 °C, 3 h	0.49 mmol of **3b**	64%

Notably, previously unknown compound **19a** and **19b** are relatively easily prepared, *new bifunctional reactive nucleosides* that can undergo S_N_Ar reactions at the C6 and metal-mediated reactions at the C2 position. To the extent that C-Cl bonds can be activated by metal catalysts compounds **4a** and **4b** also offer this type of orthogonal reactivity. Results from the orthogonal reactivities of these new halo nucleosides will be reported in the future.

### Results of Tests against HCL, TCL, CLL, and MDA-MB-231 Breast Cancer Cells

The newly synthesized compounds as well as cladribine and its bromo analogue were tested against HCL, TCL, and CLL. Data from these assays are shown in Table 4.

From these data, across the entire series, cladribine (**12a**) was best. The bromo analogue of cladribine (**21a**) showed lower activities. A comparison of the ribose analogue of cladribine (**12b**) and the bromo analogue of cladribine (**21a**) is interesting. Both compounds **12b** and **21a** show comparable activities against HCL, but the latter shows higher activities against TCL and CLL. The bromo ribose derivative **21b** showed activity but was inferior to compounds **12a**, **12b**, and **21a**. In this series, the only other compound to demonstrate any activity was the piperidinyl derivative **16a**.

**Table 4:** IC_50_ values (μM) of the cladribine and its analogues on HCL, TCL, and CLL ^a^.

Compound	HCL		TCL		CLL	
	ATP	^3^H-Leu	ATP	^3^H-Leu	ATP	^3^H-Leu
**12a**	0.065	0.090	0.020	0.039	0.004	0.011
**12b**	0.81	0.85	4.81	9.40	1.33	0.88
**13a**	>33	>33	>33	>33	>33	>33
**13b**	>32	>32	>32	>32	ND ^b^	>32
**14a**	>32	>32	>32	>32	>32	>32
**14b**	>30	>30	>30	>30	>30	>30
**15a**	>29	>29	>29	>29	>29	>29
**15b**	>28	>28	>28	>28	>28	>28
**16a**	11.5	16.3	9.6	9.0	4.3	5.4
**16b**	>27	>27	>27	>27	ND ^b^	ND ^b^
**17a**	>28	>28	>28	>28	>28	>28
**17b**	>27	>27	>27	>27	>27	>27
**18a**	>27	>27	>27	>27	>27	>27
**18b**	>26	>26	>26	>26	>26	>26
**21a**	0.76	0.96	0.13	0.16	0.13	0.14
**21b**	1.8	7.2	8.6	10.2	2.0	4.0

^a^ One patient per disease type; ^b^ ND = not determined.

**Figure 2:**
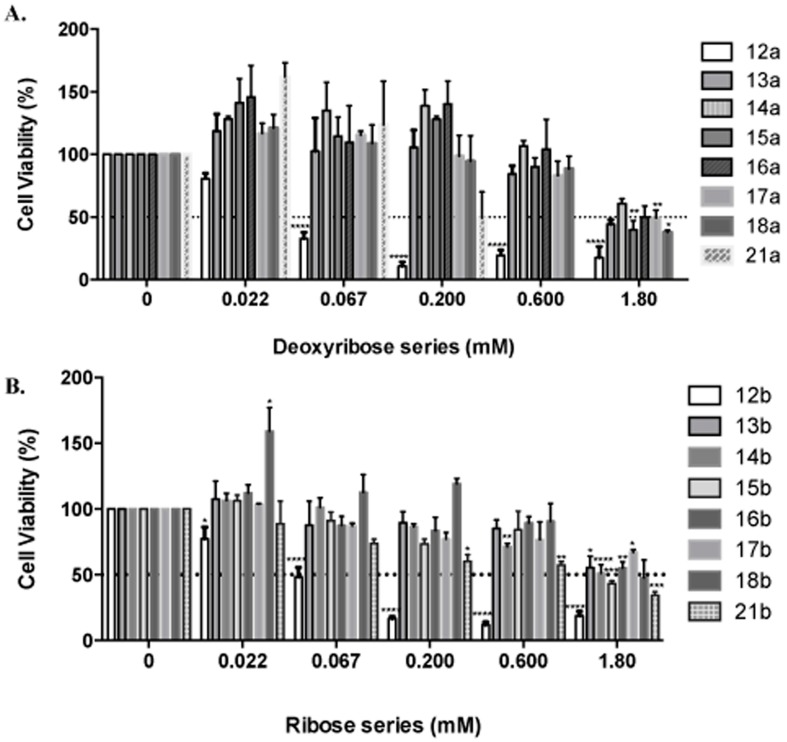
Effects of cladribine analogues on breast cancer cell viability. (**A**): Deoxyribose series and (**B**): Ribose series. Significant differences are described with * *p* ≤ 0.05, ** *p* ≤ 0.01, *** *p* ≤ 0.001, **** *p* ≤ 0.0001 compared to control.

The compounds were also tested against adenocarcinoma MDA-MB-231 breast cancer cells. These cells were treated with three-fold serial dilutions of the compounds ranging from 0–1.8 mM for 24 h. Viable cells were fixed with cold methanol and nuclei were stained with 0.4% propidium iodide (PI). Cell viability was calculated as the percentage of surviving cells after treatment as measured by differences in fluorescence units between treated and untreated wells (Figure 2).

IC_50_ values were obtained from dose response curve fittings using the non-linear regression function of GraphPad Prism^®^ (La Jolla, CA, USA). Dashed horizontal line represents 50% cell viability. Columns represent means ± SEM of at least three independent experiments. Significant differences are described with * *p* ≤ 0.05, ** *p* ≤ 0.01, *** *p* ≤ 0.001, **** *p* ≤ 0.0001 compared to control.

From the IC_50_ values shown in Table 5, the two compounds that emerged as most promising were cladribine **12a** and its ribose analogue **12b**, followed by the bromo derivatives **21a** and **21b**, which were about 10 times less active.

**Table 5:** IC_50_ values (mM) of the compounds synthesized on MDA-MB-231 breast cancer cells.

2′-Deoxyribose Series								Ribose Series							
**12a**	**13a**	**14a**	**15a**	**16a**	**17a**	**18a**	**21a**	**12b**	**13b**	**14b**	**15b**	**16b**	**17b**	**18b**	**21b**
0.05	2.15	6.28	2.54	4.18	2.32	1.88	0.64	0.06	2.39	1.70	1.54	2.45	2.57	2.91	0.55

## 3. Experimental Section

### 3.1. General Considerations

Thin-layer chromatography was performed on 200 μm aluminum-foil-backed silica gel plates for the 2′-deoxynucleosides and on Merck 60F_254_ (Merck, Billerica, MA, USA) for the ribose analogues. Column chromatographic purifications were performed on 100–200 mesh silica gel. CH_2_Cl_2_ for the chlorination reactions was distilled over CaH_2_. Precursors **3a** and **3b** were prepared as described previously [35]. The yield of **3a** was 71% on a 2.52 mmol scale and the yield of **3b** was 71% on a 2.1 mmol scale. TMSCl was redistilled prior to use and all other commercially available compounds were used without further purification. ^1^H-NMR spectra were recorded at 500 MHz or at 400 MHz in the solvents indicated under the individual compound headings and are referenced to residual protonated solvent resonances. ^13^C-NMR spectra were recorded at 125 MHz or at 100 MHz in the solvents indicated under the individual compound headings and are referenced to the solvent resonances (Supplementary Materials). Chemical shifts (δ) are reported in parts per million (ppm), and coupling constants (*J*) are in hertz (Hz). Standard abbreviations are used to designate resonance multiplicities (s = singlet, d = doublet, t = triplet, dd = doublet of doublet, ddd = doublet of doublet of doublet, quint = quintet, m = multiplet, br = broad, app = apparent). The saccharide carbons of the nucleoside are numbered 1′ through 5′ starting at the anomeric carbon atom and proceeding via the carbon chain to the primary carbinol carbon atom. The purinyl proton is designated as H-8 and the saccharide protons are designated on the basis of the carbon atom they are attached to.

*O^6^-(Benzotriazol-1-yl)-2-chloro-9-[2-deoxy-3,5-di-O-(t-butyldimethylsilyl)-*β*-d-ribofuranosyl]purine* (**4a**): A mixture of compound **3a** (1.0 g, 1.63 mmol) and SbCl_3_ (520.6 mg, 2.28 mmol) in dry CH_2_Cl_2_ (16.3 mL) was cooled to −15 °C using dry ice and acetone, in a nitrogen atmosphere. *t*-BuONO (0.678 mL, 5.70 mmol) was added dropwise and the mixture was stirred at −10 to −15 °C for 3.5 h, at which time TLC indicated the reaction to be complete. The reaction mixture was poured into ice-cold, saturated aqueous NaHCO_3_ (25 mL) with stirring. The mixture was filtered using vacuum (*note*: use of vacuum for this filtration is critical for maximizing product recovery) and the residue was washed with CH_2_Cl_2_ (25 mL). The organic layer was separated and the aqueous layer was back extracted with CH_2_Cl_2_ (2 × 15 mL). The combined organic layer was washed with water (15 mL) and brine (15 mL), dried over anhydrous Na_2_SO_4_, and evaporated under reduced pressure. Purification of the crude material on a silica gel column sequentially eluted with hexanes, 5% EtOAc in hexanes, followed by 20% EtOAc in hexanes gave 0.67 g (65% yield) of compound **4a** as a white foam. *R_f_* (SiO_2_ and 30% EtOAc in hexanes) = 0.55. ^1^H-NMR (500 MHz, CDCl_3_): δ 8.47 (s, 1H, H-8), 8.09 (d, *J* = 8.3 Hz, 1H, Ar-H), 7.52–7.41 (m, 3H, Ar-H), 6.45 (t, *J* = 5.8 Hz, 1H, H-1′), 4.62 (m, 1H, H-3′), 4.01 (m, 1H, H-4′), 3.89 (dd, *J* = 3.4, 11.2 Hz, 1H, H-5′), 3.76 (dd, *J* = 2.4, 11.2 Hz, 1H, H-5′), 2.61 (app quint, *J*_app_ ~ 6.2 Hz, 1H, H-2′), 2.49–2.48 (app quint, *J*_app_ ~ 5.8 Hz, 1H, H-2′), 0.88 (s, 18H, *t*-Bu), 0.08 and 0.07 (2s, 12H, SiCH_3_). ^13^C-NMR (125 MHz, CDCl_3_): δ 159.2, 154.9, 152.6, 144.4, 143.5, 129.0, 128.8, 125.0, 120.7, 119.1, 108.6, 88.4, 85.3, 71.6, 62.6, 41.6, 26.1, 25.9, 18.5, 18.1, −4.5, −4.7, −5.2, −5.3. HRMS (TOF) calcd for C_28_H_43_ClN_7_O_4_Si_2_ [M + H]^+^ 632.2598, found 632.2583.

*2-Chloro-3′,5′-di-O-(t-butyldimethylsilyl)-2′-deoxyadenosine* (**5a**): To a solution of compound **4a** (126.0 mg, 0.2 mmol) in 1,2-DME (2 mL), 28%–30% aqueous ammonia (32 μL) was added, and the mixture was stirred at room temperature for 1.5 h. The mixture was diluted with EtOAc (5 mL) and washed with 5% aqueous NaCl (5 mL). The organic layer was separated and the aqueous layer was back extracted with EtOAc (5 mL). The combined organic layer was dried over anhydrous Na_2_SO_4_ and evaporated under reduced pressure. The crude material was chromatographed on a silica gel column by sequential elution with 20% EtOAc in hexanes followed by 5% MeOH in CH_2_Cl_2_ to afford 85.0 mg (82% yield) of compound **5a** as a white solid. *R_f_* (SiO_2_ and 10% MeOH in CH_2_Cl_2_) = 0.40. ^1^H-NMR (500 MHz, CDCl_3_): δ 8.12 (s, 1H, H-8), 6.54 (br s, 2H, NH_2_), 6.38 (t, *J* = 6.1 Hz, 1H, H-1′), 4.61 (m, 1H, H-3′), 3.99 (app q, *J*_app_ ~ 3.4 Hz, 1H, H-4′) 3.88 (dd, *J* = 3.9, 11.2 Hz, 1H, H-5′), 3.76 (dd, *J* = 3.0, 11.2 Hz, 1H, H-5′), 2.61 (app quint, *J*_app_ ~ 6.4 Hz, 1H, H-2′), 2.44–2.39 (ddd, *J* = 3.1, 6.3, 13.2 Hz, 1H, H-2′), 0.90 (s, 18H, *t*-Bu), 0.09 and 0.08 (2s, 12H, SiCH_3_). ^13^C-NMR (125 MHz, CDCl_3_): δ 156.2, 154.0, 150.4, 139.5, 118.7, 87.9, 84.5, 71.7, 62.6, 41.3, 25.9, 25.7, 18.4, 18.0, −4.7, −4.8, −5.4, −5.5. HRMS (TOF) calcd for C_22_H_40_ClN_5_O_3_Si_2_Na [M + Na]^+^ 536.2250, found 536.2252.

### 3.2. General Procedure for the Synthesis of Cladribine Analogues

To a solution of compound **4a** (126.0 mg, 0.2 mmol) in 1,2-DME (2 mL) the appropriate amine (1.5 equiv) was added, and the mixture was stirred at room temperature. The mixture was diluted with EtOAc (5 mL for compounds **6a**, **9a**, **11a**, and 15 mL for compounds **7a**, **8a**, **10a**) and washed 5% aqueous NaCl (5 mL for compounds **6a**, **9a**, **11a**, and 15 mL for compounds **7a**, **8a**, **10a**). The organic layer was separated and the aqueous layer was back extracted with EtOAc (5 mL for compounds **6a**, **9a**, **11a**, and 15 mL for compounds **7a**, **8a**, **10a**). The combined organic layer was dried over anhydrous Na_2_SO_4_ and evaporated under reduced pressure. The crude material was chromatographed on a silica gel column; see individual compound headings for details.

*2-Chloro-N^6^-methyl-3′,5′-di-O-(t-butyldimethylsilyl)-2′-deoxyadenosine* (**6a**): Compound **6a** (85.0 mg, 80% yield) was obtained as a white, foamy solid after chromatography on a silica gel column by sequential elution with 5% EtOAc in hexanes and 15% EtOAc in hexanes. *R_f_* (SiO_2_ and 30% EtOAc in hexanes) = 0.26. ^1^H-NMR (500 MHz, CDCl_3_): δ 8.02 (s, 1H, H-8), 6.37 (t, *J* = 6.4 Hz, 1H, H-1′), 6.17 (br s, 1H, NH), 4.60 (m, 1H, H-3′), 3.97 (app q, *J*_app_ ~ 3.4 Hz, 1H, H-4′), 3.87 (dd, *J* = 4.1, 11.2 Hz, 1H, H-5′), 3.75 (dd, *J* = 3.0, 11.2 Hz, 1H, H-5′), 2.61 (app quint, *J*_app_ ~ 6.4 Hz, 1H, H-2′), 2.42–2.37 (ddd, *J* = 2.8, 6.1, 12.7 Hz, 1H, H-2′), 0.90 (s, 18H, *t*-Bu), 0.09 and 0.08 (2s, 12H, SiCH_3_). ^13^C-NMR (125 MHz, CDCl_3_): δ 155.9, 154.5, 149.2; 138.7, 119.1, 87.9, 84.4, 71.8, 62.7, 41.2, 27.5, 25.9, 25.7, 18.4, 18.0, −4.7, −4.8, −5.4, −5.5. HRMS (TOF) calcd for C_23_H_42_ClN_5_O_3_Si_2_Na [M + Na]^+^ 550.2407, found 550.2419.

*2-Chloro-N^6^,N^6^-dimethyl-3′,5′-di-O-(t-butyldimethylsilyl)-2′-deoxyadenosine* (**7a**): Compound **7a** (91.1 mg, 84% yield) was obtained as a colorless, thick gum after chromatography on a silica gel column by sequential elution with hexanes, 5% EtOAc in hexanes, and 20% EtOAc in hexanes. *R_f_* (SiO_2_ and 30% EtOAc in hexanes) = 0.70. ^1^H-NMR (500 MHz, CDCl_3_): δ 7.93 (s, 1H, H-8), 6.36 (t, *J* = 6.3 Hz, 1H, H-1′), 4.58 (m, 1H, H-3′), 3.95 (m, 1H, H-4′), 3.83 (dd, *J* = 4.4, 11.2 Hz, 1H, H-5′), 3.73 (dd, *J* = 2.9, 11.2 Hz, 1H, H-5′), 3.50 (br s, 6H, N(CH_3_)_2_), 2.58 (app quint, *J*_app_ ~ 6.5 Hz, 1H, H-2′), 2.39–2.35 (m, 1H, H-2′), 0.88 (s, 18H, *t*-Bu), 0.08 and 0.06 (2s, 12H, SiCH_3_). ^13^C-NMR (125 MHz, CDCl_3_): δ 155.2, 153.8, 151.4, 137.2, 119.6, 87.9, 84.4, 72.1, 62.9, 41.1, 26.1, 26.0, 25.9, 25.8, 18.6, 18.2, −4.5, −4.6, −5.2, −5.3. HRMS (TOF) calcd for C_24_H_45_ClN_5_O_3_Si_2_ [M + H]^+^ 542.2744, found 542.2749.

*2-Chloro-6-(pyrrolidin-1-yl)-9-[2-deoxy-3,5-di-O-(t-butyldimethylsilyl)-*β*-d-ribofuranosyl]purine* (**8a**): Compound **8a** (93.3 mg, 82% yield) was obtained as a colorless, thick gum after chromatography on a silica gel column by sequential elution with hexanes, 5% EtOAc in hexanes, and 20% EtOAc in hexanes. *R_f_* (SiO_2_ and 30% EtOAc in hexanes) = 0.65. ^1^H-NMR (500 MHz, CDCl_3_): δ 7.92 (s, 1H, H-8), 6.37 (t, *J* = 6.6 Hz, 1H, H-1′), 4.59 (m, 1H, H-3′), 4.13 (br s, 2H, pyrrolidinyl NCH), 3.96 (m, 1H, H-4′), 3.83 (dd, *J* = 4.9, 11.2 Hz, 1H, H-5′), 3.74 (dd, *J* = 3.9, 11.2 Hz, 1H, H-5′), 3.79–3.73 (br m, 2H, pyrrolidinyl NCH), 2.61 (app quint, *J*_app_ ~ 6.5 Hz, 1H, H-2′), 2.39–2.35 (m, 1H, H-2′), 2.10–2.0 (br m, 2H, pyrrolidinyl CH), 1.96–1.85 (br m, 2H, pyrrolidinyl CH), 0.90 (s, 18H, *t*-Bu), 0.09 and 0.07 (2s, 12H, SiCH_3_). ^13^C-NMR (125 MHz, CDCl_3_): δ 154.3, 153.5, 151.0, 137.8, 119.8, 88.1, 84.4, 72.3, 63.1, 49.1, 41.08, 26.4, 26.2, 25.9, 24.3, 18.6, 18.2, −4.5, −4.6, −5.2, −5.3. HRMS (TOF) calcd. for C_26_H_47_ClN_5_O_3_Si_2_ [M + H]^+^ 568.2900, found 568.2906.

*2-Chloro-6-(piperidin-1-yl)-9-[2-deoxy-3,5-di-O-(t-butyldimethylsilyl)-*β*-d-ribofuranosyl]purine* (**9a**): Compound **9a** (99.0 mg, 85% yield) was obtained as a white, foamy solid after chromatography on a silica gel column by sequential elution with 5% EtOAc in hexanes and 15% EtOAc in hexanes. *R_f_* (SiO_2_ and 30% EtOAc in hexanes) = 0.65. ^1^H-NMR (500 MHz, CDCl_3_): δ 7.92 (s, 1H, H-8), 6.37 (t, *J* = 6.6 Hz, 1H, H-1′), 4.59 (m, 1H, H-3′), 4.45–3.73 (br m, 4H, piperidinyl N(CH_2_)_2_), 3.96 (app q, *J*_app_ ~ 3.6 Hz, 1H, H-4′), 3.83 (dd, *J* = 4.9, 11.2 Hz, 1H, H-5′), 3.74 (dd, *J* = 3.4, 11.2 Hz, 1H, H-5′), 2.59 (app quint, *J*_app_ ~ 6.3 Hz, 1H, H-2′), 2.40–2.35 (ddd, *J* = 3.3, 6.2, 13.3 Hz, 1H, H-2′), 1.68 (br m, 6H, piperidinyl CH), 0.90 (s, 18H, *t*-Bu), 0.09 and 0.07 (2s, 12H, SiCH_3_). ^13^C-NMR (125 MHz, CDCl_3_): δ 153.8, 151.4, 136.7, 119.0, 109.9, 87.8, 84.2, 72.0, 62.8, 46.3, 40.9, 26.1, 25.9, 25.7, 24.6, 18.4, 18.0, −4.7, −4.8, −5.4, −5.5. HRMS (TOF) calcd for C_27_H_49_ClN_5_O_3_Si_2_ [M + H] ^+^ 582.3057 found 582.3074.

*2-Chloro-6-(morpholin-4-yl)-9-[2-deoxy-3,5-di-O-(t-butyldimethylsilyl)-*β*-d-ribofuranosyl]purine* (**10a**): Compound **10a** (97.4 mg, 83% yield) was obtained as a colorless, thick gum after chromatography on a silica gel column by sequential elution with hexanes, 5% EtOAc in hexanes, and 20% EtOAc in hexanes. *R_f_* (SiO_2_ and 30% EtOAc in hexanes) = 0.68. ^1^H-NMR (500 MHz, CDCl_3_): δ 7.98 (s, 1H, H-8), 6.37 (t, *J* = 6.3 Hz, 1H, H-1′), 4.58 (m, 1H, H-3′), 4.55–3.98 (br m, 4H, morpholinyl N(CH_2_)_2_), 3.96 (m, 1H, H-4′), 3.84 (dd, *J* = 4.4, 11.2 Hz, 1H, H-5′), 3.78 (t, *J* = 4.6 Hz, 4H, morpholinyl CH_2_OCH_2_), 3.74 (dd, *J* = 2.9, 11.2 Hz, 1H, H-5′), 2.56 (app quint, *J*_app_ ~ 6.5 Hz, 1H, H-2′), 2.40–2.36 (m, 1H, H-2′), 0.89 (s, 18H, *t*-Bu), 0.08 and 0.07 (2s, 12H, SiCH_3_). ^13^C-NMR (125 MHz, CDCl_3_): δ 154.0, 153.9, 151.8, 137.6, 119.3, 88.0, 84.4, 72.0, 67.1, 62.9, 43.5 (br), 41.3, 26.1, 26.0, 25.9, 18.6, 18.2, −4.5, −4.6, −5.2, −5.3. HRMS (TOF) calcd for C_26_H_47_ClN_5_O_4_Si_2_ [M + H]^+^ 584.2850, found 584.2855.

*2-Chloro-6-[(2-dimethylamino)ethyl)(methyl)amino)]-9-[2-deoxy-3,5-di-O-(t-butyldimethylsilyl)-*β*-d-ribofuranosyl]purine* (**11a**): Compound **11a** (77.0 mg, 64% yield) was obtained as a pale-yellow, thick gum after chromatography on a silica gel column by sequential elution with 10% EtOAc in hexanes, 20% EtOAc in hexanes, and 10% MeOH in CH_2_Cl_2_. *R_f_* (SiO_2_ and 10% MeOH in CH_2_Cl_2_) = 0.10. ^1^H-NMR (500 MHz, CDCl_3_): δ 7.94 (s, 1H, H-8), 6.36 (t, *J* = 6.3 Hz, 1H, H-1′), 4.62 (m, 1H, H-3′), 4.28–3.84 (br m, 2H, NCH_2_), 3.99 (app q, *J*_app_ ~ 3.7 Hz, 1H, H-4′), 3.85 (dd, *J* = 4.4, 11.2 Hz, 1H, H-5′), 3.78 (dd, *J* = 3.4, 11.2 Hz, 1H, H-5′), 3.47 (br s, 3H, NCH_3_), 2.65–2.59 (m, 2H, NCH_2_ and 1H, H-2′), 2.41–2.37 (ddd, *J* = 4.2, 6.2, 13.1 Hz, 1H, H-2′), 2.34 (s, 6H, N(CH_3_)_2_), 0.92 and 0.91 (s, 18H, *t*-Bu), 0.11, 0.083, and 0.079 (3s, 12H, SiCH_3_). ^13^C-NMR (125 MHz, CDCl_3_): δ 154.9, 153.8, 151.4, 137.2, 119.4, 87.9, 84.4, 72.0, 62.9, 57.0, 48.6, 45.7, 41.0, 36.9, 26.0, 25.8, 18.4, 8.0, −4.6, −4.8, −5.4, −5.5. HRMS (TOF) calcd for C_27_H_52_ClN_6_O_3_Si_2_ [M + H]^+^ 599.3322 found 599.3313.

*O^6^-(Benzotriazol-1-yl)-2-chloro-9-[2,3,5-tri-O-(t-butyldimethylsilyl)-β-d-ribofuranosyl]purine* (**4b**): To a solution of *t-*BuONO (2.78 g, 26.91 mmol) in CH_2_Cl_2_ (197 mL) was added TMSCl (1.46 g, 13.45 mmol) at 0 °C. To this mixture a solution of **3b** (2 g, 2.69 mmol) in CH_2_Cl_2_ (197 mL) was added dropwise, and then the reaction mixture was stirred at 0 °C for 1 h at which time the reaction was completed as indicated by TLC. The reaction mixture was diluted with CH_2_Cl_2_ (200 mL), washed with saturated NaHCO_3_ (100 mL), H_2_O (100 mL), and brine (100 mL). The organic layer was dried over anhydrous Na_2_SO_4_ and evaporated under reduced pressure. Purification of the crude material on a silica gel column sequentially eluted with hexanes, 5% EtOAc in hexanes, and 20% EtOAc in hexanes gave 1.4 g (68% yield) of compound **4b** as a white foam. *R_f_* (SiO_2_ and 30% EtOAc in hexanes) = 0.55. ^1^H-NMR (400 MHz, CDCl_3_): δ 8.56 (s, 1H, H-8), 8.14 (d, *J* = 8.0 Hz, 1H, Ar-H), 7.55–7.45 (m, 3H, Ar-H), 6.05 (d, *J* = 4.0 Hz, 1H, H-1′), 4.57 (t, *J* = 4.2 Hz, 1H, H-2′), 4.34 (t, *J* = 4.4 Hz, 1H, H-3′), 4.18–4.15 (m, 1H, H-4′), 4.08 (dd, *J* = 3.6, 11.6 Hz, 1H, H-5′), 3.83 (dd, *J* = 2.4, 11.6 Hz, 1H, H-5′), 0.96, 0.93, and 0.88 (3s, 27H, *t*-Bu), 0.15, 0.11, 0.02, and −0.11 (4s, 18H, SiCH_3_). ^13^C-NMR (100 MHz, CDCl_3_): δ 159.0, 154.9, 152.6, 144.4, 143.4, 128.9, 128.7, 124.9, 120.6, 119.0, 108.5, 89.4, 85.3, 71.2, 62.0, 26.1, 25.8, 25.6, 18.5, 18.0, 17.9, −4.3, −4.6, −4.7, −4.9, −5.3, −5.4. HRMS (TOF) calcd for C_34_H_57_ClN_7_O_5_Si_3_ [M + H]^+^ 762.3412, found 762.3427.

*2-Chloro-2′,3′,5′-tri-O-(t-butyldimethylsilyl)adenosine* (**5b**): To a solution of compound **4b** (500 mg, 0.655 mmol) in 1,2-DME (8 mL), 28%–30% aqueous ammonia (64 μL) was added and the mixture was stirred at room temperature for 1.5 h. The reaction mixture was diluted with EtOAc (25 mL) and washed 5% aqueous NaCl (25 mL). The organic layer was separated and the aqueous layer was back extracted with EtOAc (25 mL). The combined organic layer was dried over anhydrous Na_2_SO_4_ and evaporated under reduced pressure. The crude material was chromatographed on a silica gel column by sequential elution with 20% EtOAc in hexanes and 5% MeOH in EtOAc to afford 310 mg (75% yield) of compound **5b** as an off-white solid. *R_f_* (SiO_2_ and 10% MeOH in EtOAc) = 0.21. ^1^H-NMR (400 MHz, CDCl_3_): δ 8.12 (s, 1H, H-8), 6.17 (br s, 2H, NH_2_), 5.93 (d, *J* = 4.8 Hz, 1H, H-1′), 4.69 (t, *J* = 4.6 Hz, 1H, H-2′), 4.32 (t, *J* = 4.6 Hz, H-3′), 4.14–4.06 (m, 1H, H-4′), 4.06 (dd, *J* = 4.8, 11.6 Hz, 1H, H-5′), 3.80 (dd, *J* = 2.8, 11.2 Hz, 1H, H-5′), 0.94, 0.91, and 0.84 (3s, 27H, *t*-Bu), 0.14, 0.09, −0.01, and −0.17 (4s, 18H, SiCH_3_). ^13^C-NMR (100 MHz, CDCl_3_): δ 156.1, 154.0, 150.7, 140.2, 118.9, 88.9, 85.4, 75.5, 71.7, 62.3, 26.0, 25.8, 25.7, 18.5, 18.0, 17.9, −4.3, −4.5, −4.7, −5.0, −5.3, −5.4. HRMS (TOF) calcd. for C_28_H_55_ClN_5_O_4_Si_3_ [M + H]^+^ 644.3245 found 644.3217.

### 3.3. General Procedure for the Synthesis of Ribose Analogues of Cladribine

To a solution of compound **4b** (500 mg, 0.655 mmol) in 1,2-DME (8 mL) was added the appropriate amine (1.5 equiv) and the mixture was stirred at room temperature. The mixture was diluted with EtOAc (25 mL) and washed with 5% aqueous NaCl (15 mL). The organic layer was separated and the aqueous layer was back extracted with EtOAc (15 mL). The combined organic layers were dried over anhydrous Na_2_SO_4_ and evaporated under reduced pressure. The crude material was chromatographed on a silica gel column. See individual compound headings for details.

*2-Chloro-N^6^-methyl-2′,3′,5′-tri-O-(t-butyldimethylsilyl)adenosine* (**6b**): Compound **6b** (340 mg, 80% yield) was obtained as a pale-yellow, thick gum after chromatography on a silica gel column by sequential elution with 10% EtOAc in hexanes, 20% EtOAc in hexanes, and 50% EtOAc in hexanes. R_f_ (SiO_2_ and EtOAc) = 0.20. ^1^H-NMR (400 MHz, CDCl_3_): δ 8.02 (s, 1H, H-8), 6.04 (br s, 1H, NH), 5.91 (d, J = 5.2 Hz, 1H, H-1′), 4.72 (t, J = 4.6 Hz, 1H, H-2′), 4.32 (t, J = 4.0 Hz, 1H, H-3′), 4.12–4.10 (m, 1H, H-4′), 4.06 (dd, J = 5.2, 11.6 Hz, 1H, H-5′), 3.79 (dd, J = 2.8, 11.2 Hz, 1H, H-5′), 3.17 (s, 3H, CH_3_), 0.94, 0.92, and 0.81 (3s, 27H, t-Bu), 0.13, 0.06, −0.02, and −0.19 (4s, 18H, SiCH_3_). ^13^C-NMR (100 MHz, CDCl_3_): δ 155.9, 154.5, 139.3, 119.3, 88.8, 85.4, 75.3, 71.5, 65.2, 62.4, 26.0, 25.8, 25.7, 18.4, 18.0, 17.8, 14.0, 11.0, −4.4, −4.7, −5.1, −5.4, −5.7. HRMS (TOF) calcd for C_29_H_57_ClN_5_O_4_Si_3_ [M + H]^+^ 658.3401, found 658.3416.

*2-Chloro-N^6^,N^6^-dimethyl- 2′,3′,5′-tri-O-(t-butyldimethylsilyl)adenosine* (**7b**): Compound **7b** (360 mg, 81% yield) was obtained as a pale-yellow, thick gum after chromatography on a silica gel column by sequential elution with 10% EtOAc in hexanes and 20% EtOAc in hexanes. *R_f_* (SiO_2_ and 20% EtOAc in hexanes) = 0.40. ^1^H-NMR (400 MHz, CDCl_3_): δ 7.93 (s, 1H, H-8), 5.90 (d, *J* = 5.2 Hz, 1H, H-1′), 4.77 (t, *J* = 4.8 Hz, 1H, H-2′), 4.31 (t, *J* = 3.8 Hz, 1H, H-3′), 4.04–4.09 (m, 1H, H-4′), 4.06 (dd, *J* = 5.6, 11.2 Hz, 1H, H-5′), 3.78 (dd, *J* = 2.8, 11.2 Hz, 1H, H-5′), 3.58 (br s, 6H, CH_3_), 0.94, 0.92, and 0.82 (3s, 27H, *t*-Bu), 0.13, 0.10, −0.02, and −0.18 (4s, 18H, SiCH_3_). ^13^C-NMR (100 MHz, CDCl_3_): δ 155.9, 153.7, 151.4, 137.9, 119.7, 88.8, 85.4, 74.8, 72.0, 62.5, 38.4, 26.0, 25.8, 25.7, 18.4, 18.0, 17.9, −4.3, −4.7, −5.0, −5.4. HRMS (TOF) calcd for C_30_H_59_ClN_5_O_4_Si_3_ [M + H]^+^ 672.3558, found 672.3561.

*2-Chloro-6-(pyrrolidin-1-yl)-9-[2,3,5-tri-O-(t-butyldimethylsilyl)-*β*-d-ribofuranosyl]purine* (**8b**): Compound **8b** (390 mg, 85% yield) was obtained as a pale-yellow, thick gum after chromatography on a silica gel column by sequential elution with 10% EtOAc in hexanes and 20% EtOAc in hexanes. *R_f_* (SiO_2_ and 20% EtOAc in hexanes) = 0.30. ^1^H-NMR (400 MHz, CDCl_3_): δ 7.90 (s, 1H, H-8), 5.90 (d, *J* = 5.2 Hz, 1H, H-1′), 4.81 (t, *J* = 5.0 Hz, 1H, H-2′), 4.32 (t, *J* = 4.0 Hz, 1H, H-3′), 4.14–4.08 (m, 1H, H-4′ and br m, 2H, pyrrolidinyl NCH), 4.06 (dd, *J* = 5.6, 11.2 Hz, 1H, H-5′), 3.77–3.73 (m, 3H, H-5′ and pyrrolidinyl NCH), 2.04 (br m, 2H, pyrrolidinyl CH), 1.98 (br m, 2H, pyrrolidinyl CH), 0.93, 0.89, and 0.81 (3s, 27H, *t*-Bu), 0.12, 0.11, −0.03, and −0.20 (4s, 18H, SiCH_3_). ^13^C-NMR (100 MHz, CDCl_3_): δ 154.0, 153.4, 151.0, 138.0, 119.9, 88.7, 85.5, 74.6, 72.2, 62.6, 48.9, 47.6, 26.0, 25.8, 25.7, 18.4, 18.1, 17.9, −4.4, −4.6, −4.7, −5.0, −5.3. HRMS (TOF) calcd for C_32_H_61_ClN_5_O_4_Si_3_ [M + H]^+^ 698.3714 found 698.3731.

*2-Chloro-6-(piperidin-1-yl)-9-[2,3,5-tri-O-(t-butyldimethylsilyl)-*β*-d-ribofuranosyl]purine* (**9b**): Compound **9b** (375 mg, 80% yield) was obtained as a pale-yellow, thick gum after chromatography on a silica gel column by sequential elution with 10% EtOAc in hexanes and 20% EtOAc in hexanes. *R_f_* (SiO_2_ and 20% EtOAc in hexanes) = 0.50. ^1^H-NMR (400 MHz, CDCl_3_): δ 7.91 (s, 1H, H-8), 5.89 (d, *J* = 5.6 Hz, 1H, H-1′), 4.79 (t, *J* = 4.8 Hz, 1H, H-2′), 4.32 (t, *J* = 4.0 Hz, 1H, H-3′), 4.22 (br s, 4H, piperidinyl NCH_2_), 4.11–4.09 (m, 1H, H-4′), 4.06 (dd, *J* = 5.2, 10.8 Hz, 1H, H-5′), 3.77 (dd, *J* = 2.8, 10.8 Hz, 1H, H-5′), 1.69 (br m, 6H, piperidinyl CH_2_), 0.94, 0.90, and 0.84 (3s, 27H, *t*-Bu), 0.12, 0.10, −0.02, and −0.17 (4s, 18H, SiCH_3_). ^13^C-NMR (100 MHz, CDCl_3_): δ 153.9, 153.8, 151.6, 137.6, 119.4, 88.8, 85.3, 74.7, 71.9, 62.5, 29.6, 25.8, 25.7, 24.6, 22.6, 18.4, 18.0, 17.9, 14.0, −4.3, −4.7, −5.0, −5.3. HRMS (TOF) calcd for C_33_H_63_ClN_5_O_4_Si_3_ [M + H]^+^ 712.3871, found 712.3875.

*2-Chloro-6-(morpholin-4-yl)-9-[2,3,5-tri-O-(t-butyldimethylsilyl)-*β*-d-ribofuranosyl]purine* (**10b**): Compound **10b** (430 mg, 90% yield) was obtained as a pale-yellow, thick gum after chromatography on a silica gel column by sequential elution with 10% EtOAc in hexanes and 20% EtOAc in hexanes. *R_f_* (SiO_2_ and 20% EtOAc in hexanes) = 0.47. ^1^H-NMR (400 MHz, CDCl_3_): δ 7.98 (s, 1H, H-8), 5.92 (d, *J* = 4.8 Hz, 1H, H-1′), 4.73 (t, *J* = 4.8 Hz, 1H, H-2′), 4.31–4.20 (t, *J* = 4.2 Hz, 1H, H-3′and br m, 4H, morpholinyl N(CH_2_)_2_), 4.12–4.09 (m, 1H, H-4′), 4.06 (dd, *J* = 5.2, 11.2 Hz, 1H, H-5′), 3.83 (t, *J* = 5.0 Hz, 4H, morpholinyl CH_2_OCH_2_), 3.77 (dd, *J* = 3.2, 11.2 Hz, 1H, H-5′), 0.94, 0.92, and 0.83 (3s, 27H, *t*-Bu), 0.13, 0.10, −0.01, and −0.16 (4s, 18H, SiCH_3_). ^13^C-NMR (100 MHz, CDCl_3_): δ 153.9, 153.7, 151.8, 138.1, 119.4, 88.8, 85.3,77.3,77.0,76.6,75.0, 71.8, 66.9, 62.3, 45.60, 29.6, 29.3, 26.0,25.8, 25.7, 22.6, 18.4, 18.0, 17.8,14.0, −4.3, −4.7, −5.0, −5.4. HRMS (TOF) calcd for C_32_H_61_ClN_5_O_5_Si_3_ [M + H]^+^ 714.3664 found 714.3668.

*2-Chloro-6-[(2-dimethylamino)ethyl)(methyl)amino)]-9-[2,3,5-tri-O-(t-butyldimethylsilyl)-*β*-d-ribofuranosyl] purine* (**11b**): Compound **11b** (280 mg, 58%) was obtained as a pale-yellow, thick gum after chromatography on a silica gel column by sequential elution with 10% MeOH in CH_2_Cl_2_, 20% MeOH in CH_2_Cl_2_, and 30% MeOH in CH_2_Cl_2_. *R_f_* (SiO_2_ and 10% MeOH in CH_2_Cl_2_) = 0.10. ^1^H-NMR (500 MHz, CDCl_3_): δ 7.97 (s, 1H, H-8), 5.90 (d, *J* = 4.8 Hz, 1H, H-1′), 4.72 (t, *J* = 4.8 Hz, 1H, H-2′), 4.32 (t, *J* = 3.8 Hz, 1H, H-3′), 4.15–4.10 (m, 1H, H-4′ and br s, 2H, NCH_2_), 4.07 (dd, *J* = 5.2, 11.2 Hz, 1H, H-5′), 3.78 (dd, *J* = 2.8, 11.2 Hz, 1H, H-5′ and br m, 2H, NCH_2_), 2.59 (br s, 2H, NCH_2_), 2.32 (s, 6H, N(CH_3_)_2_), 0.94, 0.92, and 0.82 (3s, 27H, *t*-Bu), 0.13, 0.10, −0.01, and −0.15 (4s, 18H, SiCH_3_). ^13^C-NMR (100 MHz, CDCl_3_): δ 154.8, 153.7, 151.4, 137.9, 119.5, 88.8, 85.2, 75.0, 71.9, 62.4, 45.7, 29.6, 26.0, 25.8, 25.7, 18.4, 18.0, 17.9, −4.3, −4.7, −5.0, −5.4. HRMS (TOF) calcd for C_33_H_66_ClN_6_O_4_Si_3_ [M + H]^+^ 729.4136, found 729.4157.

### 3.4. General Procedure for the Desilylation of Cladribine Analogues

To a 0.1 M solution of the silylated compound in anhydrous MeOH, KF (2 equiv/silyl group) was added. The mixture was heated at 80 °C for 20–26 h, cooled, and silica gel was added. The mixture was evaporated to dryness and the compound-impregnated silica gel was loaded onto a wet-packed silica gel column. The products were obtained by elution with appropriate solvents (see the individual compound headings for details).

*2-Chloro-2′-deoxyadenosine* (**12a**): Prepared from compound **5a** (60.0 mg, 0.117 mmol) and KF (27.0 mg, 0.467 mmol) in MeOH (1.17 mL). Chromatography on a silica gel column sequentially eluted with 5% MeOH in EtOAc and 10% MeOH in EtOAc gave 24.0 mg (72% yield) of compound **12a** as an off-white solid. *R_f_* (SiO_2_ and 10% MeOH in EtOAc) = 0.13. ^1^H-NMR (500 MHz, CD_3_OD): δ 8.28 (s, 1H, H-8), 6.36 (t, *J* = 6.8 Hz, 1H, H-1′), 4.57 (m, 1H, H-3′), 4.04 (m, 1H, H-4′), 3.84 (dd, *J* = 2.9, 12.2 Hz, 1H, H-5′), 3.74 (dd, *J* = 3.4, 12.2 Hz, 1H, H-5′), 2.76 (app quint, *J*_app_ ~ 6.7 Hz, 1H, H-2′), 2.43–2.39 (ddd, *J* = 2.9, 5.9, 13.2 Hz, 1H, H-2′). ^13^C-NMR (125 MHz, CD_3_OD): δ 158.3, 155.3, 151.4, 141.9, 119.8, 89.9, 87.0, 73.0, 63.6, 41.6. HRMS (TOF) calcd for C_10_H_12_ClN_5_O_3_Na [M + Na]^+^ 308.0521, found 308.0523.

*2-Chloro-N^6^-methyl-2′-deoxyadenosine* (**13a**) [50]: Prepared from compound **6a** (80.0 mg, 0.154 mmol) and KF (36.0 mg, 0.618 mmol) in MeOH (1.54 mL). Chromatography on a silica gel column sequentially eluted with 2.5% MeOH in EtOAc and 5% MeOH in EtOAc gave 39.0 mg (85% yield) of compound **13a** as a white, foamy solid. *R_f_* (SiO_2_ and 10% MeOH in EtOAc) = 0.21. ^1^H-NMR (500 MHz, CD_3_OD): δ 8.18 (s, 1H, H-8), 6.34 (t, *J* = 6.8 Hz, 1H, H-1′), 4.56 (m, 1H, H-3′), 4.04 (m, 1H, H-4′), 3.84 (dd, *J* = 2.4, 12.2 Hz, 1H, H-5′), 3.74 (dd, *J* = 3.4, 12.2 Hz, 1H, H-5′), 3.05 (br s, 3H, NCH_3_), 2.76 (app quint, *J*_app_ ~ 6.8 Hz, 1H, H-2′), 2.42–2.38 (ddd, *J* = 2.9, 5.8, 13.7 Hz, 1H, H-2′). ^13^C-NMR (125 MHz, CD_3_OD): δ 157.3, 155.5, 150.1, 141.2, 120.4, 89.9, 87.0, 73.0, 63.7, 41.6, 27.8. HRMS (TOF) calcd for C_11_H_14_ClN_5_O_3_Na [M + Na]^+^ 322.0677, found 322.0682.

*2-Chloro-N^6^,N^6^-dimethyl-2′-deoxyadenosine* (Cladribine, **14a**): Prepared from compound **7a** (80.0 mg, 0.147 mmol) and KF (34.3 mg, 0.590 mmol) in MeOH (1.4 mL). Chromatography on a silica gel column sequentially eluted with 50% EtOAc in hexanes, EtOAc, and 10% MeOH in EtOAc gave 38.0 mg (82% yield) of compound **14a** as a white, foamy solid. *R_f_* (SiO_2_ and 5% MeOH in EtOAc) = 0.29. ^1^H-NMR (500 MHz, CD_3_OD): δ 8.16 (s, 1H, H-8), 6.35 (t, *J* = 6.8 Hz, 1H, H-1′), 4.56 (m, 1H, H-3′), 4.04 (m, 1H, H-4′), 3.84 (dd, *J* = 2.9, 12.2 Hz, 1H, H-5′), 3.73 (dd, *J* = 3.4, 12.2 Hz, 1H, H-5′), 3.70–3.10 (br m, 6H, N(CH_3_)_2_), 2.74 (app quint, *J*_app_ ~ 6.8 Hz, 1H, H-2′), 2.41–2.36 (ddd, *J* = 2.9, 5.8, 13.2 Hz, 1H, H-2′). ^13^C-NMR (125 MHz, CD_3_OD): δ 156.4, 154.6, 152.2, 140.0, 120.72, 89.8, 86.9, 73.0, 63.7, 41.5, 39.0 (br). HRMS (TOF) calcd for C_12_H_16_ClN_5_O_3_Na [M + Na]^+^ 336.0834, found 336.0823.

*2-Chloro-6-(pyrrolidin-1-yl)-9-(2-deoxy-*β*-d-ribofuranosyl)purine* (**15a**): Prepared from compound **8a** (60.0 mg, 0.105 mmol) and KF (24.5 mg, 0.422 mmol) in MeOH (1.0 mL). Chromatography on a silica gel column sequentially eluted with 50% EtOAc in hexanes, EtOAc, and 10% MeOH in EtOAc gave 29.1 mg (81% yield) of compound **15a** as a white solid. *R_f_* (SiO_2_ and 5% MeOH in EtOAc) = 0.19. ^1^H-NMR (500 MHz, CD_3_OD): δ 8.19 (s, 1H, H-8), 6.36 (t, *J* = 7.1 Hz, 1H, H-1′), 4.59 (m, 1H, H-3′), 4.14–4.07 (br m, 2H, pyrrolidinyl NCH), 4.04 (m, 1H, H-4′), 3.84 (dd, *J* = 3.4, 12.7 Hz, 1H, H-5′), 3.74 (dd, *J* = 3.4, 12.2 Hz, 1H, H-5′), 3.71–3.62 (br m, 2H, pyrrolidinyl NCH), 2.75 (app quint, *J*_app_ ~ 6.7 Hz, 1H, H-2′), 2.40–2.36 (ddd, *J* = 2.9, 5.8, 13.2 Hz, 1H, H-2′), 2.15–2.06 (br m, 2H, pyrrolidinyl CH), 2.04–1.92 (br m, 2H, pyrrolidinyl CH). ^13^C-NMR (125 MHz, CD_3_OD): δ 155.0, 154.7, 151.7, 140.6, 120.8, 89.9, 87.0, 73.0, 63.7, 50.2, 49.6, 41.6, 27.3, 25.2. HRMS (TOF) calcd for C_14_H_19_ClN_5_O_3_ [M + H]^+^ 340.1171, found 340.1149.

*2-Chloro-6-(piperidin-1-yl)-9-(2-deoxy-*β*-d-ribofuranosyl)purine* (**16a**): Prepared from compound **9a** (70.0 mg, 0.120 mmol) and KF (28.0 mg, 0.481 mmol) in MeOH (1.20 mL). Chromatography on a silica gel column sequentially eluted with EtOAc and 2% MeOH in EtOAc gave 30.0 mg (71% yield) of compound **16a** as a white, foamy solid. *R_f_* (SiO_2_ and 10% MeOH in EtOAc) = 0.46. ^1^H-NMR (500 MHz, CD_3_OD): δ 8.15 (s, 1H, H-8), 6.34 (t, *J* = 6.6 Hz, 1H, H-1′), 4.55 (m, 1H, H-3′), 4.18 (br s, 4H, piperidinyl N(CH_2_)_2_), 4.03 (m, 1H, H-4′), 3.83 (dd, *J* = 2.4, 12.2 Hz, 1H, H-5′), 3.73 (dd, *J* = 3.4, 12.2 Hz, 1H, H-5′), 2.74 (app quint, *J*_app_ ~ 6.8 Hz, 1H, H-2′), 2.40–2.35 (ddd, *J* = 2.7, 5.9, 13.5 Hz, 1H, H-2′), 1.74 (br m, 2H, piperidinyl CH_2_), 1.65 (br m, 4H, piperidinyl CH_2_). ^13^C-NMR (125 MHz, CD_3_OD): δ 155.3, 154.9, 152.7, 139.6, 120.5, 89.8, 86.8, 73.0, 63.7, 47.8, 41.6, 27.3, 25.7. HRMS (TOF) calcd for C_15_H_20_ClN_5_O_3_Na [M + Na]^+^ 376.1147, found 376.1148.

*2-Chloro-6-(morpholin-4-yl)-9-(2-deoxy-*β*-d-ribofuranosyl)purine* (**17a**): Prepared from compound **10a** (80.0 mg, 0.137 mmol) and KF (31.8 mg, 0.548 mmol) in MeOH (1.4 mL). Chromatography on a silica gel column sequentially eluted with 50% EtOAc in hexanes, EtOAc, and 10% MeOH in EtOAc gave 36.1 mg (74% yield) of compound **17a** as a white, foamy solid. *R_f_* (SiO_2_ and 5% MeOH in EtOAc) = 0.21. ^1^H-NMR (500 MHz, CD_3_OD): δ 8.22 (s, 1H, H-8), 6.37 (t, *J* = 7.1 Hz, 1H, H-1′), 4.56 (m, 1H, H-3′), 4.40–4.12 (br m, 4H, morpholinyl N(CH_2_)_2_), 4.04 (m, 1H, H-4′), 3.83 (dd, *J* = 2.9, 12.2 Hz, 1H, H-5′), 3.79 (t, *J* = 4.9 Hz, 4H, morpholinyl CH_2_OCH_2_), 3.74 (dd, *J* = 3.4, 12.2 Hz, 1H, H-5′), 2.74 (app quint, *J*_app_ ~ 6.7 Hz, 1H, H-2′), 2.41–2.37 (ddd, *J* = 2.9, 5.8, 13.2 Hz, 1H, H-2′). ^13^C-NMR (125 MHz, CD_3_OD): δ 155.2, 154.7, 152.7, 140.3, 120.6, 89.8, 86.8, 72.9, 68.0, 63.6, 47.0 (br), 41.5. HRMS (TOF) calcd for C_14_H_19_ClN_5_O_4_ [M + H]^+^ 356.1120, found 356.1107.

*2-Chloro-6-[(2-dimethylamino)ethyl)(methyl)amino)]-9-(2-deoxy-*β*-d-ribofuranosyl)purine* (**18a**): Prepared from compound **11a** (70.0 mg, 0.117 mmol) and KF (27.0 mg, 0.467 mmol) in MeOH (1.17 mL). Chromatography on a silica gel column sequentially eluted with 10% MeOH in EtOAc and 20% MeOH in EtOAc gave 36.0 mg (84% yield) of compound **18a** as a white, foamy solid. *R_f_* (SiO_2_ and 10% MeOH in EtOAc) = 0.10. ^1^H-NMR (500 MHz, CD_3_OD): δ 8.14 (s, 1H, H-8), 6.35 (t, *J* = 6.9 Hz, 1H, H-1′), 4.56 (m, 1H, H-3′), 4.12 (br s, 2H, NCH_2_), 4.03 (m, 1H, H-4′), 3.83 (dd, *J* = 3.4, 12.2 Hz, 1H, H-5′), 3.74 (dd, *J* = 3.9, 12.2 Hz, 1H, H-5′), 3.48 (br s, 3H, NCH_3_), 2.76–2.71 (br m, 3H, NCH_2_ and 1H, H-2′), 2.42–2.41 (m, 1H, H-2′), 2.39 (s, 6H, N(CH_3_)_2_). ^13^C-NMR (125 MHz, CD_3_OD): δ 156.4, 154.7, 152.5, 140.1, 120.8, 89.8, 86.8, 73.0, 63.7, 57.5, 45.8, 41.6, 37.6, (one broadened resonance could not be identified). HRMS (TOF) calcd for C_15_H_24_ClN_6_O_3_ [M + H]^+^ 371.1598, found 371.1584.

### 3.5. General Procedure for the Desilylation of Ribose Cladribine Analogues

To a 0.1 M solution of the silylated compound in anhydrous MeOH, KF (2 equiv/silyl group) was added. The mixture was heated at 80 °C for 24 h, cooled, and silica gel was added. The mixture was evaporated to dryness and the compound-impregnated silica gel was loaded onto a wet-packed silica gel column. The products were obtained by elution with appropriate solvents (see the individual compound headings for details).

*2-Chloroadenosine* (**12b**): Prepared from compound **5b** (100 mg, 0.155 mmol) and KF (54.0 mg, 0.93 mmol) in MeOH (1.55 mL). Chromatography on a silica gel column sequentially eluted with 5% MeOH in EtOAc and 10% MeOH in EtOAc gave 35.0 mg (74% yield) of compound **12b** as an off-white solid. *R_f_* (SiO_2_ and 10% MeOH in EtOAc) = 0.13. ^1^H-NMR (400 MHz, CD_3_OD): δ 8.27 (s, 1H, H-8), 5.92 (d, *J* = 6.0 Hz, 1H, H-1′), 4.72 (t, *J* = 5.6, 1H, H-2′), 4.32 (dd, *J* = 2.8, 5.2 Hz, 1H, H-3′), 4.14 (m, 1H, H-4′), 3.90 (dd, *J* = 2.8, 12.4 Hz, 1H, H-5′), 3.76 (dd, *J* = 2.8, 12.4 Hz, 1H, H-5′). ^13^C-NMR (100 MHz, DMSO-*d*_6_): δ 174.5, 156.7, 152.9, 150.3, 140.0, 118.1, 87.4, 85.7, 73.8, 70.3, 61.3, 25.3. HRMS (TOF) calcd for C_10_H_13_ClN_5_O_4_ [M + H]^+^ 302.0651, found 302.0627.

*2-Chloro-N^6^-methyladenosine* (**13b**): Prepared from compound **6b** (100 mg, 0.152 mmol) and KF (52.9 mg, 0.912 mmol) in MeOH (1.52 mL). Chromatography on a silica gel column sequentially eluted with 2.5% MeOH in EtOAc and 5% MeOH in EtOAc gave 36.0 mg (75% yield) of compound **13b** as a white, foamy solid. *R_f_* (SiO_2_ and 10% MeOH in EtOAc) = 0.21. ^1^H-NMR (400 MHz, CD_3_OD): δ 8.20 (s, 1H, H-8), 5.90 (d, *J* = 6.0 Hz, 1H, H-1′), 4.68 (t, *J* = 5.6 Hz, 1H, H-2′), 4.31 (dd, *J* = 2,8, 4.8 Hz, 1H, H-3′), 4.14 (m, 1H, H-4′), 3.96 (dd, *J* = 2.4, 12.4 Hz, 1H, H-5′), 3.76 (dd, *J* = 2.8, 12.4 Hz, 1H, H-5′), 3.07 (br s, 3H, NCH_3_). ^13^C-NMR (100 MHz, DMSO-*d*_6_): δ 155.5, 153.2, 149.2, 139.7, 118.6, 87.3, 85.6, 73.6, 70.6, 70.3, 61.6, 61.3, 53.9, 27.1. HRMS (TOF) calcd for C_11_H_15_ClN_5_O_4_ [M + H]^+^ 316.0807, found 316.0808.

*2-Chloro-N^6^,N^6^-dimethyladenosine* (**14b**): Prepared from compound **7b** (100 mg, 0.148 mmol) and KF (51.8 mg, 0.892 mmol) in MeOH (1.48 mL). Chromatography on a silica gel column sequentially eluted with 50% EtOAc in hexanes, EtOAc, and 10% MeOH in EtOAc gave 36.8 mg (75% yield) of compound **14b** as an off-white, foamy solid. *R_f_* (SiO_2_ and 5% MeOH in EtOAc) = 0.32. ^1^H-NMR (400 MHz, CD_3_OD): δ 8.17 (s, 1H, H-8), 5.91 (d, *J* = 6.4 Hz, 1H, H-1′), 4.67 (t, *J* = 5.4 Hz, 1H, H-2′), 4.31 (dd, *J* = 2.8, 4.8 Hz, 1H, H-3′), 4.14 (m, 1H, H-4′), 3.90 (dd, *J* = 2.8, 12.4 Hz, 1H, H-5′), 3.76 (dd, *J* = 2.4, 12.4 Hz, 1H, H-5′), 3.60–3.49 (br m, H, N(CH_3_)_2_). ^13^C-NMR (100 MHz, DMSO-*d*_6_): δ 154.4, 152.5, 151.1, 138.6, 118.5, 87.2, 85.6, 73.6, 70.2, 61.2, 37.5 (br). HRMS (TOF) calcd for C_12_H_17_ClN_5_O_4_ [M + H]^+^ 330.0964, found 330.0964.

*2-Chloro-6-(pyrrolidin-1-yl)-9-(*β*-d-ribofuranosyl)purine* (**15b**): Prepared from compound **8b** (100 mg, 0.143 mmol) and KF (49.8 mg, 0.858 mmol) in MeOH (1.43 mL). Chromatography on a silica gel column sequentially eluted with 50% EtOAc in hexanes, EtOAc, and 10% MeOH in EtOAc gave 43.3 mg (85% yield) of compound **15b** as a white solid. *R_f_* (SiO_2_ and 5% MeOH in EtOAc) = 0.38. ^1^H-NMR (400 MHz, CD_3_OD): δ 8.18 (s, 1H, H-8), 5.91 (d, *J* = 6.4 Hz, 1H, H-1′), 4.67 (t, *J* = 5.4 Hz, 1H, H-2′), 4.32 (dd, *J* = 2.8, 4.8 Hz, 1H, H-3′), 4.14 (m, 1H, H-4′), 4.13–4.10 (br m, 2H, pyrrolidinyl NCH), 3.90 (dd, *J* = 2.8, 12.8 Hz, 1H, H-5′), 3.74 (dd, *J* = 2.4, 12.4 Hz, 1H, H-5′), 3.72–3.65 (br m, 2H, pyrrolidinyl NCH), 2.13–2.07 (br m, 2H, pyrrolidinyl CH), 2.05–1.95 (br m, 2H, pyrrolidinyl CH). ^13^C-NMR (100 MHz, DMSO-*d*_6_): δ 152.7, 152.7, 150.7, 139.1, 118.7, 87.2, 85.6, 73.7, 70.2, 61.2, 48.6, 47.3, 25.6, 23.6. HRMS (TOF) calcd for C_14_H_19_ClN_5_O_4_ [M + H]^+^ 356.1120, found 356.1127.

*2-Chloro-6-(piperidin-1-yl)-9-(*β*-d-ribofuranosyl)purine* (**16b**): Prepared from compound **9b** (100 mg, 0.140 mmol) and KF (48.9 mg, 0.842 mmol) in MeOH (1.40 mL). Chromatography on a silica gel column sequentially eluted with EtOAc and 2% MeOH in EtOAc gave 43.6 mg (84% yield) of compound **16b** as a white, foamy solid. *R_f_* (SiO_2_ and 10% MeOH in EtOAc) = 0.41. ^1^H-NMR (400 MHz, CD_3_OD): δ 8.13 (s, 1H, H-8), 5.87 (d, *J* = 6.0 Hz, 1H, H-1′), 4.64 (t, *J* = 5.4 Hz, 1H, H-2′), 4.27 (dd, *J* = 2.8, 5.2 Hz, 1H, H-3′), 4.19 (br s, 4H, piperidinyl N(CH_2_)_2_), 4.11 (m, 1H, H-4′), 3.89 (dd, *J* = 2.8, 12.2 Hz, 1H, H-5′), 3.73 (dd, *J* = 2.4, 12.2 Hz, 1H, H-5′), 1.74 (br m, 2H, piperidinyl CH_2_), 1.64 (br m, 4H, piperidinyl CH_2_). ^13^C-NMR (100 MHz, DMSO-*d*_6_): δ 153.1, 152.6, 151.4, 138.5, 118.2, 87.2, 85.6, 73.6, 70.4, 70.2, 70.1, 61.2, 44.8 (br), 25.6, 25.2, 23.9, 14.0. HRMS (TOF) calcd for C_15_H_21_ClN_5_O_4_ [M + H]^+^ 370.1277, found 370.1302.

*2-Chloro-6-(morpholin-4-yl)-9-(*β*-d-ribofuranosyl)purine* (**17b**): Prepared from compound **10b** (100 mg, 0.139 mmol) and KF (48.7 mg, 0.839 mmol) in MeOH (1.40 mL). Chromatography on a silica gel column sequentially eluted with 50% EtOAc in hexanes, EtOAc, and 10% MeOH in EtOAc gave 40.6 mg (78% yield) of compound **17b** as a white, foamy solid. *R_f_* (SiO_2_ and 5% MeOH in EtOAc) = 0.21. ^1^H-NMR (400 MHz, CD_3_OD): δ 8.21 (s, 1H, H-8), 5.92 (d, *J* = 6.0 Hz, 1H, H-1′), 4.66 (t, *J* = 5.6 Hz, 1H, H-2′), 4.32 (dd, *J* = 2.8, 4.8 Hz, 1H, H-3′), 4.30–4.24 (br m, 4H, morpholinyl N(CH_2_)_2_), 4.14 (m, 1H, H-4′), 3.90 (dd, *J* = 2.8, 12.4 Hz, 1H, H-5′), 3.80 (t, *J* = 4.8 Hz, 4H, morpholinyl CH_2_OCH_2_), 3.76 (dd, *J* = 2.8, 12.4 Hz, 1H, H-5′). ^13^C-NMR (100 MHz, DMSO-*d*_6_): δ 153.3, 152.5, 151.6, 139.0, 118.3, 87.3, 85.6, 85.4, 73.7, 70.4, 70.2, 66.0, 61.4, 61.1, 48.5, 45.0 (br). HRMS (TOF) calcd for C_14_H_19_ClN_5_O_5_ [M + H]^+^ 372.1069, found 372.1056.

*2-Chloro-6-[(2-dimethylamino)ethyl)(methyl)amino)]-9-(*β*-d-ribofuranosyl)purine* (**18b**): Prepared from compound **11b** (100 mg, 0.137 mmol) and KF (47.7 mg, 0.822 mmol) in MeOH (1.37 mL). Chromatography on a silica gel column sequentially eluted with 10% MeOH in EtOAc and 20% MeOH in EtOAc gave 34.5 mg (65% yield) of compound **18b** as a white, foamy solid. *R_f_* (SiO_2_ and 15% MeOH in EtOAc) = 0.10. ^1^H-NMR (400 MHz, CD_3_OD): δ 8.19 (s, 1H, H-8), 5.92 (d, *J* = 6 Hz, 1H, H-1′), 4.67 (t, 1H, *J* = 5.6 Hz, 1H, H-2′), 4.34 (dd, *J* = 3.2, 5.2 Hz 1H, H-3′), 4.14 (d, *J* = 2.4 Hz, 3H, H-4′), 3.90 (dd, *J* = 2.4, 12.4 Hz, 1H, H-5′), 3.76 (dd, *J* = 2.8, 12.4 Hz, 1H, H-5′), 3.49 (br s, 3H, NCH_3_), 2.83 (t, 2H, NCH_2,_ *J* = 6.8 Hz ), 2.47 (s, 6H, N(CH_3_)_2_). ^13^C-NMR (100 MHz, CD_3_OD): δ 156.1, 154.7, 152.3, 140.5, 120.7, 90.8, 87.7, 75.3, 72.3, 63.2, 57.0, 49.6, 49.4, 49.2, 49.0, 48.7, 48.5, 48.3, 45.5, 37.4. HRMS (TOF) calcd for C_15_H_24_ClN_6_O_4_ [M + H]^+^ 387.1542, found 387.1513.

*O^6^-(Benzotriazol-1-yl)-2-bromo-9-[2-deoxy-3,5-di-O-(t-butyldimethylsilyl)-*β*-d-ribofuranosyl]purine* (**19a**): A mixture of compound **3a** (300.0 mg, 0.489 mmol) and SbBr_3_ (247.7 mg, 0.685 mmol) in dry CH_2_Br_2_ (4.9 mL) was cooled to −15 °C using dry ice and acetone, in a nitrogen atmosphere. *t*-BuONO (203.8 μL, 1.713 mmol) was added dropwise and the mixture was stirred at −10 °C to −15 °C for 2 h. Because TLC indicated the presence of starting material, another aliquot of *t*-BuONO (203.8 μL, 1.713 mmol) was added and the reaction proceeded for 1 h at −15 °C, at which time TLC indicated the reaction to be complete. The reaction mixture was poured into ice-cold, saturated aqueous NaHCO_3_ (5 mL) with stirring. The mixture was filtered using vacuum (*note:* use of vacuum for this filtration is critical for maximizing product recovery) and the residue was washed with CH_2_Cl_2_ (5 mL). The organic layer was separated and the aqueous layer was back extracted with CH_2_Cl_2_ (2 × 5 mL). The combined organic layer was washed with water (5 mL) and brine (5 mL), dried over anhydrous Na_2_SO_4_, and evaporated under reduced pressure. Purification of the crude material on a silica gel column sequentially eluted with hexanes, 5% EtOAc in hexanes, and 30% EtOAc in hexanes gave 208.5 mg (63% yield) of compound **19a** as a white foam. *R_f_* (SiO_2_ and 30% EtOAc in hexanes) = 0.60. ^1^H-NMR (500 MHz, CDCl_3_): δ 8.45 (s, 1H, H-8), 8.13 (d, *J* = 8.3 Hz, 1H, Ar-H), 7.56–7.45 (m, 3H, Ar-H), 6.47 (t, *J* = 5.9 Hz, 1H, H-1′), 4.63 (m, 1H, H-3′), 4.03 (m, 1H, H-4′), 3.90 (dd, *J* = 3.4, 11.2 Hz, 1H, H-5′), 3.78 (dd, *J* = 1.5, 11.2 Hz, 1H, H-5′), 2.61 (app quint, *J*_app_ ~ 6.2 Hz, 1H, H-2′), 2.49 (app quint, *J*_app_ ~ 5.8 Hz, 1H, H-2′), 0.91 (s, 18H, *t*-Bu), 0.11 and 0.09 (2s, 12H, SiCH_3_). ^13^C-NMR (125 MHz, CDCl_3_): δ 158.7, 154.9, 144.3, 143.6, 142.6, 129.1, 128.9, 125.1, 120.8, 119.5, 108.7, 88.5, 85.4, 71.7, 62.7, 41.8, 26.2, 25.9, 18.6, 18.2, −4.4, −4.6, −5.2, −5.3. HRMS (TOF) calcd for C_28_H_43_BrN_7_O_4_Si_2_ [M + H]^+^ 676.2093, found 676.2078.

*2-Bromo-3′,5′-di-O-(t-butyldimethylsilyl)-2′-deoxyadenosine* (**20a**): To a solution of compound **19a** (135.3 mg, 0.20 mmol) in 1,2-DME (2 mL), 28%–30% aqueous ammonia (48.6 μL) was added, and the mixture was stirred at room temperature for 45 min. The mixture was diluted with EtOAc (15 mL) and washed with 5% aqueous NaCl (10 mL). The organic layer was separated and the aqueous layer was back extracted with EtOAc (15 mL). The combined organic layer was dried over anhydrous Na_2_SO_4_ and evaporated under reduced pressure. The crude material was chromatographed on a silica gel column by sequential elution with hexanes, 20% EtOAc in hexanes and 40% EtOAc in hexanes to afford 93.1 mg (83% yield) of compound **20a** as a white foam. *R_f_* (SiO_2_ and EtOAc) = 0.50. ^1^H-NMR (500 MHz, CDCl_3_): δ 8.08 (s, 1H, H-8), 6.63 (br s, 2H, Ar-NH_2_), 6.37 (t, *J* = 6.1 Hz, 1H, H-1′), 4.61 (m, 1H, H-3′), 3.98 (m, 1H, H-4′), 3.88 (dd, *J* = 3.9, 11.2 Hz, 1H, H-5′), 3.75 (dd, *J* = 2.4, 11.2 Hz, 1H, H-5′), 2.61 (app quint, *J*_app_ ~ 6.3 Hz, 1H, H-2′), 2.43–2.38 (m, 1H, H-2′), 0.90 (s, 18H, *t*-Bu), 0.09 and 0.08 (2s, 12H, SiCH_3_). ^13^C-NMR (125 MHz, CDCl_3_): δ 156.3, 150.4, 144.9, 139.6, 119.3, 88.2, 84.7, 71.9, 62.9, 41.4, 26.1, 25.9, 18.6, 18.2, −4.4, −4.6, −5.2, −5.3. HRMS (TOF) calcd for C_22_H_41_BrN_5_O_3_Si_2_ [M + H]^+^ 558.1926, found 558.1902.

*2-Bromo-2′-deoxyadenosine* (**21a**): As described in the general desilylation procedures, compound **21a** was prepared from compound **20a** (80.0 mg, 0.143 mmol) and KF (33.2 mg, 0.572 mmol) in MeOH (1.4 mL), over 28 h. Chromatography on a silica gel column sequentially eluted with 50% EtOAc in hexanes, EtOAc, and 10% MeOH in EtOAc gave 31.1 mg (66% yield) of compound **21a** as a white/off-white solid. *R_f_* (SiO_2_ and 10% MeOH in EtOAc) = 0.40. ^1^H-NMR (500 MHz, CD_3_OD): δ 8.25 (s, 1H, H-8), 6.36 (t, *J* = 6.8 Hz, 1H, H-1′), 4.57 (m, 1H, H-3′), 4.04 (m, 1H, H-4′), 3.84 (dd, *J* = 2.9, 12.2 Hz, 1H, H-5′), 3.74 (dd, *J* = 3.4, 12.2 Hz, 1H, H-5′), 2.76 (app quint, *J*_app_ ~ 6.7 Hz, 1H, H-2′), 2.43–2.39 (ddd, *J* = 2.9, 5.9, 13.2 Hz, 1H, H-2′). ^13^C-NMR (125 MHz, CD_3_OD): δ 158.1, 151.4, 145.9, 141.7, 120.3, 89.9, 86.9, 72.9, 63.7, 41.6. HRMS (TOF) calcd for C_10_H_12_BrN_5_O_3_Na [M + Na]^+^ 352.0016, found 352.0021.

*O^6^-(Benzotriazol-1-yl)-2-bromo-9-[2,3,5-tri-O-(t-butyldimethylsilyl)-*β*-d-ribofuranosyl]purine* (**19b**): A mixture of compound **3b** (500.0 mg, 0.67 mmol) and SbBr_3_ (340.8 mg, 0.94 mmol) in dry CH_2_Br_2_ (8.3 mL) was cooled to −15 °C using dry ice and acetone, in a nitrogen atmosphere. *t*-BuONO (280 μL, 2.352 mmol) was added dropwise and the mixture was stirred at −10 °C to −15 °C for 2 h. Because TLC indicated the presence of starting material, another aliquot of *t*-BuONO (280 μL, 2.352 mmol) was added and the reaction was allowed to progress for 1 h at −15 °C, at which time TLC indicated the reaction to be complete. The reaction mixture was poured into ice-cold, saturated aqueous NaHCO_3_ (10 mL) with stirring. The mixture was filtered using vacuum (*note:* use of vacuum for this filtration is critical for maximizing product recovery) and the residue was washed with CH_2_Cl_2_ (15 mL). The organic layer was separated and the aqueous layer was back extracted with CH_2_Cl_2_ (2 × 15 mL). The combined organic layer was washed with water (10 mL) and brine (10 mL), dried over anhydrous Na_2_SO_4_, and evaporated under reduced pressure. Purification of the crude material on a silica gel column sequentially eluted with hexanes, 5% EtOAc in hexanes, and 30% EtOAc in hexanes gave 350 mg (64% yield) of compound **19b** as a white foam. *R_f_* (SiO_2_ and 30% EtOAc in hexanes) = 0.80. ^1^H-NMR (400 MHz, CDCl_3_): δ 8.54 (s, 1H, H-8), 8.15 (d, *J* = 8.4 Hz, 1H, Ar-H), 7.58–7.45 (m, 3H, Ar-H), 6.05 (d, *J* = 4.0 Hz, 1H, H-1′), 4.57 (t, *J* = 4.2 Hz, 1H, H-2′), 4.33 (t, *J* = 4.6 Hz, 1H, H-3′), 4.17 (m, 1H, H-4′), 4.08 (dd, *J* = 3.6, 11.6 Hz, 1H, H-5′), 3.83 (dd, *J* = 2.4, 11.6 Hz, 1H, H-5′), 0.96, 0.93, and 0.85 (3s, 27H, *t*-Bu), 0.16, 0.11, −0.03, and −0.09 (4s, 18H, SiCH_3_). ^13^C-NMR (100 MHz, CDCl_3_): δ 158.5, 154.8, 144.2, 143.4, 142.5, 128.8, 128.7, 124.8, 120.6, 119.4, 108.5, 89.5, 85.2, 76.1, 71.1, 61.9, 29.6, 26.1, 25.8, 25.6, 18.5, 18.0, 17.9, −4.2, −4.6, −4.7, −4.9, −5.3, −5.4. HRMS (TOF) calcd for C_34_H_57_BrN_7_O_5_Si_3_ [M + H]^+^ 806.2907, found 806.2912.

*2-Bromo-2′,3′,5′-tri-O-(t-butyldimethylsilyl)-adenosine* (**20b**): To a solution of compound **19b** (200 mg, 0.247 mmol) in 1,2-DME (4 mL), 28%–30% aqueous ammonia (60 μL) was added, and the mixture was stirred at room temperature for 45 min. The mixture was diluted with EtOAc (20 mL) and washed with 5% aqueous NaCl (20 mL). The organic layer was separated and the aqueous layer was back extracted with EtOAc (25 mL). The combined organic layer was dried over anhydrous Na_2_SO_4_ and evaporated under reduced pressure. The crude material was chromatographed on a silica gel column by sequential elution with hexanes, 20% EtOAc in hexanes, and 40% EtOAc in hexanes to afford 140 mg (82% yield) of compound **20b** as a white foam. *R_f_* (SiO_2_ and EtOAc) = 0.55. ^1^H-NMR (400 MHz, CDCl_3_): δ 8.10 (s, 1H, H-8), 5.92 (d, *J* = 4.8 Hz, 1H, H-1′), 5.77 (br s, 2H, Ar-NH_2_), 4.69 (t, *J* = 4.6 Hz, 1H, H-2′), 4.32 (t, *J* = 4.2 Hz, 1H, H-3′), 4.13 (m, 1H, H-4′), 4.06 (dd, *J* = 4.8, 11.2 Hz, 1H, H-5′), 3.80 (dd, *J* = 2.8, 11.2 Hz, 1H, H-5′), 0.95, 0.93, and 0.83 (3s, 27H, *t*-Bu), 0.14, 0.11, −0.00, and −0.15 (4s, 18H, SiCH_3_). ^13^C-NMR (100 MHz, CDCl_3_): δ 155.8, 150.5, 144.6, 140.0, 119.4, 110.0, 89.0, 85.3, 75.4, 71.7, 62.3, 26.0, 25.8, 25.7, 18.4, 18.0, 17.9, −4.3, −4.7, −5.0, −5.3, −5.4. HRMS (TOF) calcd for C_28_H_55_BrN_5_O_4_Si_3_ [M + H]^+^ 688.2740, found 688.2728.

*2-Bromoadenosine* (**21b**): As described in the general desilylation procedures, compound **21b** was prepared from compound **20b** (100 mg, 0.145 mmol) and KF (50.5 mg, 0.870 mmol) in MeOH (1.9 mL), over 24 h. Chromatography on a silica gel column sequentially eluted with 50% EtOAc in hexanes, EtOAc, and 10% MeOH in EtOAc which gave 34.9 mg (69% yield) of compound **21b** as a pale yellow solid. *R_f_* (SiO_2_ and 10% MeOH in EtOAc) = 0.39. ^1^H-NMR (400 MHz, CD_3_OD): δ 8.25 (s, 1H, H-8), 5.92 (d, *J* = 6.4 Hz, 1H, H-1′), 4.67 (d, *J* = 5.6 Hz, 1H, H-2′), 4.32 (dd, *J* = 2.8, 4.8 Hz, 1H, H-3′), 4.15 (m, 1H, H-4′), 3.90 (dd, *J* = 2.8, 12.4 Hz, 1H, H-5′), 3.76 (dd, *J* = 2.8, 12.4 Hz, 1H, H-5′). ^13^C-NMR (100 MHz, CD_3_OD): δ 156.5, 150.2, 144.1, 139.8, 118.4, 87.2, 85.7, 73.6, 70.3, 61.3. HRMS (TOF) calcd for C_10_H_13_BrN_5_O_4_ [M + H]^+^ 346.0145, found 346.0154.

### 3.6. Protocols for Tests against HCL, TCL, and CCL

Blood was obtained in sodium heparin tubes from patients as part of protocols with consent forms approved by the investigators review board (IRB) of the National Cancer Institute. The blood was diluted 1:1 with PBS without calcium or magnesium, layered over 15 mL Ficoll in 50 mL tubes, and centrifuged to obtain mononuclear cells. Patients with high lymphocytosis had leukemic cells >80%–90% pure after Ficoll. The cells were viably frozen in 7.5% DMSO in leucine-poor media (LPM, 88% leucine-free RPMI, 2% RPMI, and 10% FBS) in cryovials and stored under liquid nitrogen. LPM also contained penicillin, streptomycin, glutamine, gentamycin, and doxycycline. To assay, thawed cells were washed, suspended in LPM, added to 96-well round-bottom plates (15 μL/well), and treated with 15 μL of purine analogues diluted in LPM. The aliquots were incubated 3 days, then treated with 10 μL of either ATP (CellTiter-Glo, Promega, Madison, WI, USA) or {^3^H}-leucine (Perkin-Elmer, Waltham, MA, USA) diluted in leucine-free RPMI. After 30 min of ATP, the plate is read for bioluminescence. After 6 h of {^3^H}-leucine, the cells were liberated by freeze-thaw, harvested on to glass-fiber filters, counted either by a beta scintillation counter. The number of cells cultured in 30 μL aliquots for ATP assay was 20,000, 20,000, and 100,000 for HCL, TCL, and CLL, respectively. The cell number for {^3^H}-leucine assay was 60,000, 60,000 and 200,000 for HCL, TCL, and CLL, respectively. HCL and TCL cells were pulsed with 1 μCi of {^3^H}-leucine, while CLL cells were pulsed with 1.5–2 μCi/well. The IC_50_ was the calculated concentration needed for 50% inhibition, defined as the ATP uptake or {^3^H}-leucine incorporation corresponding to halfway between that of control (cells with LPM alone) and that of cycloheximide 10 μg/mL. Reported IC_50_ values were the means of 3 triplicate experiments.

### 3.7. Protocols for Tests against Breast Cancer Cells

MDA-MB-231 breast cancer (BC) cells were obtained from the American Type Culture Collection (ATCC, Manassas, VA, USA), and were cultured at 37 °C in 5% CO_2_ using culture medium recommended by the supplier. Each tested compound was dissolved in sterile DMSO, then diluted (4.8–57.7 mM) in sterile 1X PBS for a final DMSO concentration of 0.1%. Then, 2 × 10^5^ cells/well, were seeded and cultured for 24 h as described [51,52]. Afterwards, the media was changed to 5% FBS for 1 h and cells were treated in duplicate with dilutions of each treatment (0–1.8 mM) for 24 h. Cells were fixed (cold methanol), and nuclei stained (0.4% propidium iodide, (PI)) (Sigma-Aldrich, St Louis, MO, USA), and measured using a GloMax^®^ Microplate Reader (Promega, Madison, WI, USA). Cell viability was calculated as percent of surviving cells after treatment relative to vehicle wells. The IC_50_ was obtained from dose response curve fittings using the non-linear regression function of GraphPad Prism^®^ version 6.0b for Mac (GraphPad Software, San Diego, CA, USA) [53].

## 4. Conclusions

We have demonstrated, for the first time, the synthesis of *O*^6^-(benzotriazol-1-yl)-2-chloro-9-[2-deoxy-3,5-di-*O*-(*t*-butyldimethylsilyl)-β-d-ribofuranosyl]purine (**4a**) by diazotization-chlorination of *O*^6^-(benzotriazol-1-yl)-3′,5′-di-*O*-(*t*-butyldimethylsilyl)-2′-deoxyguanosine (**3a**) with *t*-BuONO and SbCl_3_ in CH_2_Cl_2_. This procedure afforded better yields than the chlorination using *t*-BuONO and Me_3_SiCl. This compound and its ribose analogue, *O*^6^-(benzotriazol-1-yl)-2-chloro-9-[2,3,5-tri-*O*-(*t*-butyldimethylsilyl)-β-d-ribofuranosyl]purine, both undergo smooth reactions with ammonia, and primary, and secondary amines to produce cladribine (**12a**), its N-modified analogues (**13a**–**18a**), and the corresponding ribose derivatives (**12b**–**18b**), after a simple desilylation with KF in MeOH. These compounds were tested against HCL, TCL, and CLL, but none of the new compounds was more active than cladribine itself. The bromo as well as ribose analogues of cladribine displayed activity but the bromo analogue of cladribine was more active against TCL and CLL as compared to both the ribose equivalent and the bromo ribose analogue of cladribine. The compound containing both the bromine atom and a ribose ring was least active among the compounds possessing a primary amino group at the C6 position. Thus, it appears that a free amino group at this location is critical to the activity of cladribine. Interestingly, the C6 piperidinyl analog of cladribine showed low activity. Tests against MDA-MB-231 breast cancer cells showed that only cladribine and its ribose analogue showed some activity. The bromo analogues were about 10 times less active and all others showed no potential. Despite the lack of a major improvement in the activity of cladribine, or the identification of new compounds with activity against breast cancer, this work has provided a route to four doubly functionalizable nucleoside derivatives. The orthogonal reactivities of these compounds, *i.e.*, S_N_Ar at the C6 position and metal catalysis at the C2 position, can be used for development of novel nucleoside analogues. We anticipate pursuing further work along these lines in the future.

## Supplementary Materials

Supplementary can be accessed at: http://www.mdpi.com/1420-3049/20/10/18437/s1.
