# Supplementary material for: Cladribine Analogues via O6-(Benzotriazolyl) Derivatives of Guanine Nucleosides
Source: Molecules. 2015 Oct 9;20(10):18437–63. doi: 10.3390/molecules201018437 (PMC4841790; doi:10.3390/molecules201018437)

Supplementary Materials

Cladribine Analogues via *O*[6]-(Benzotriazolyl) Derivatives of Guanine Nucleosides

Sakilam Satishkumar, Prasanna K. Vuram, Siva Subrahmanyam Relangi, Venkateshwarlu Gurram, Hong Zhou, Robert J. Kreitman, Michelle M. Martínez Montemayor, Lijia Yang, Muralidharan Kaliyaperumal, Somesh Sharma, Narender Pottabathini and Mahesh K. Lakshman *

Table of Contents

**Information**	**Page**
500 MHz ^{1}H-NMR spectrum of compound **4a** in $CDCl_3$	S-3
125 MHz ^{13}C-NMR spectrum of compound **4a** in $CDCl_3$	S-4
500 MHz ^{1}H-NMR spectrum of compound **5a** in $CDCl_3$	S-5
125 MHz ^{13}C-NMR spectrum of compound **5a** in $CDCl_3$	S-6
500 MHz ^{1}H-NMR spectrum of compound **6a** in $CDCl_3$	S-7
125 MHz ^{13}C-NMR spectrum of compound **6a** in $CDCl_3$	S-8
500 MHz ^{1}H-NMR spectrum of compound **7a** in $CDCl_3$	S-9
125 MHz ^{13}C-NMR spectrum of compound **7a** in $CDCl_3$	S-10
500 MHz ^{1}H-NMR spectrum of compound **8a** in $CDCl_3$	S-11
125 MHz ^{13}C-NMR spectrum of compound **8a** in $CDCl_3$	S-12
500 MHz ^{1}H-NMR spectrum of compound **9a** in $CDCl_3$	S-13
125 MHz ^{13}C-NMR spectrum of compound **9a** in $CDCl_3$	S-14
500 MHz ^{1}H-NMR spectrum of compound **10a** in $CDCl_3$	S-15
125 MHz ^{13}C-NMR spectrum of compound **10a** in $CDCl_3$	S-16
500 MHz ^{1}H-NMR spectrum of compound **11a** in $CDCl_3$	S-17
125 MHz ^{13}C-NMR spectrum of compound **11a** in $CDCl_3$	S-18
400 MHz ^{1}H-NMR spectrum of compound **4b** in $CDCl_3$	S-19
100 MHz ^{13}C-NMR spectrum of compound **4b** in $CDCl_3$	S-20
400 MHz ^{1}H-NMR spectrum of compound **5b** in $CDCl_3$	S-21
100 MHz ^{13}C-NMR spectrum of compound **5b** in $CDCl_3$	S-22
400 MHz ^{1}H-NMR spectrum of compound **6b** in $CDCl_3$	S-23
100 MHz ^{13}C-NMR spectrum of compound **6b** in $CDCl_3$	S-24
400 MHz ^{1}H-NMR spectrum of compound **7b** in $CDCl_3$	S-25
100 MHz ^{13}C-NMR spectrum of compound **7b** in $CDCl_3$	S-26
400 MHz ^{1}H-NMR spectrum of compound **8b** in $CDCl_3$	S-27
100 MHz ^{13}C-NMR spectrum of compound **8b** in $CDCl_3$	S-28
400 MHz ^{1}H-NMR spectrum of compound **9b** in $CDCl_3$	S-29
100 MHz ^{13}C-NMR spectrum of compound **9b** in $CDCl_3$	S-30
400 MHz ^{1}H-NMR spectrum of compound **10b** in $CDCl_3$	S-31
100 MHz ^{13}C-NMR spectrum of compound **10b** in $CDCl_3$	S-32
400 MHz ^{1}H-NMR spectrum of compound **11b** in $CDCl_3$	S-33
100 MHz ^{13}C-NMR spectrum of compound **11b** in $CDCl_3$	S-34
500 MHz ^{1}H-NMR spectrum of compound **12a** in CD_3OD	S-35
125 MHz ^{13}C-NMR spectrum of compound **12a** in CD_3OD	S-36
500 MHz ^{1}H-NMR spectrum of compound **13a** in CD_3OD	S-37

Cont.

Information	Page
125 MHz ^{13}C-NMR spectrum of compound **13a** in CD_3OD	S-38
500 MHz ^{1}H-NMR spectrum of compound **14a** in CD_3OD	S-39
125 MHz ^{13}C-NMR spectrum of compound **14a** in CD_3OD	S-40
500 MHz ^{1}H-NMR spectrum of compound **15a** in CD_3OD	S-41
125 MHz ^{13}C-NMR spectrum of compound **15a** in CD_3OD	S-42
500 MHz ^{1}H-NMR spectrum of compound **16a** in CD_3OD	S-43
125 MHz ^{13}C-NMR spectrum of compound **16a** in CD_3OD	S-44
500 MHz ^{1}H-NMR spectrum of compound **17a** in CD_3OD	S-45
125 MHz ^{13}C-NMR spectrum of compound **17a** in CD_3OD	S-46
500 MHz ^{1}H-NMR spectrum of compound **18a** in CD_3OD	S-47
125 MHz ^{13}C-NMR spectrum of compound **18a** in CD_3OD	S-48
400 MHz ^{1}H-NMR spectrum of compound **12b** in CD_3OD	S-49
100 MHz ^{13}C-NMR spectrum of compound **12b** in DMSO-d_6	S-50
400 MHz ^{1}H-NMR spectrum of compound **13b** in CD_3OD	S-51
100 MHz ^{13}C-NMR spectrum of compound **13b** in DMSO-d_6	S-52
400 MHz ^{1}H-NMR spectrum of compound **14b** in CD_3OD	S-53
100 MHz ^{13}C-NMR spectrum of compound **14b** in DMSO-d_6	S-54
400 MHz ^{1}H-NMR spectrum of compound **15b** in CD_3OD	S-55
100 MHz ^{13}C-NMR spectrum of compound **15b** in DMSO-d_6	S-56
400 MHz ^{1}H-NMR spectrum of compound **16b** in CD_3OD	S-57
100 MHz ^{13}C-NMR spectrum of compound **16b** in DMSO-d_6	S-58
400 MHz ^{1}H-NMR spectrum of compound **17b** in CD_3OD	S-59
100 MHz ^{13}C-NMR spectrum of compound **17b** in DMSO-d_6	S-60
400 MHz ^{1}H-NMR spectrum of compound **18b** in CD_3OD	S-61
100 MHz ^{13}C-NMR spectrum of compound **18b** in CD_3OD	S-62
500 MHz ^{1}H-NMR spectrum of compound **19a** in $CDCl_3$	S-63
125 MHz ^{13}C-NMR spectrum of compound **19a** in $CDCl_3$	S-64
500 MHz ^{1}H-NMR spectrum of compound **20a** in $CDCl_3$	S-65
125 MHz ^{13}C-NMR spectrum of compound **20a** in $CDCl_3$	S-66
500 MHz ^{1}H-NMR spectrum of compound **21a** in CD_3OD	S-67
125 MHz ^{13}C-NMR spectrum of compound **21a** in CD_3OD	S-68
400 MHz ^{1}H-NMR spectrum of compound **19b** in $CDCl_3$	S-69
100 MHz ^{13}C-NMR spectrum of compound **19b** in $CDCl_3$	S-70
400 MHz ^{1}H-NMR spectrum of compound **20b** in $CDCl_3$	S-71
100 MHz ^{13}C-NMR spectrum of compound **20b** in $CDCl_3$	S-72
400 MHz ^{1}H-NMR spectrum of compound **21b** in CD_3OD	S-73
100 MHz ^{13}C-NMR spectrum of compound **21b** in DMSO-d_6	S-74

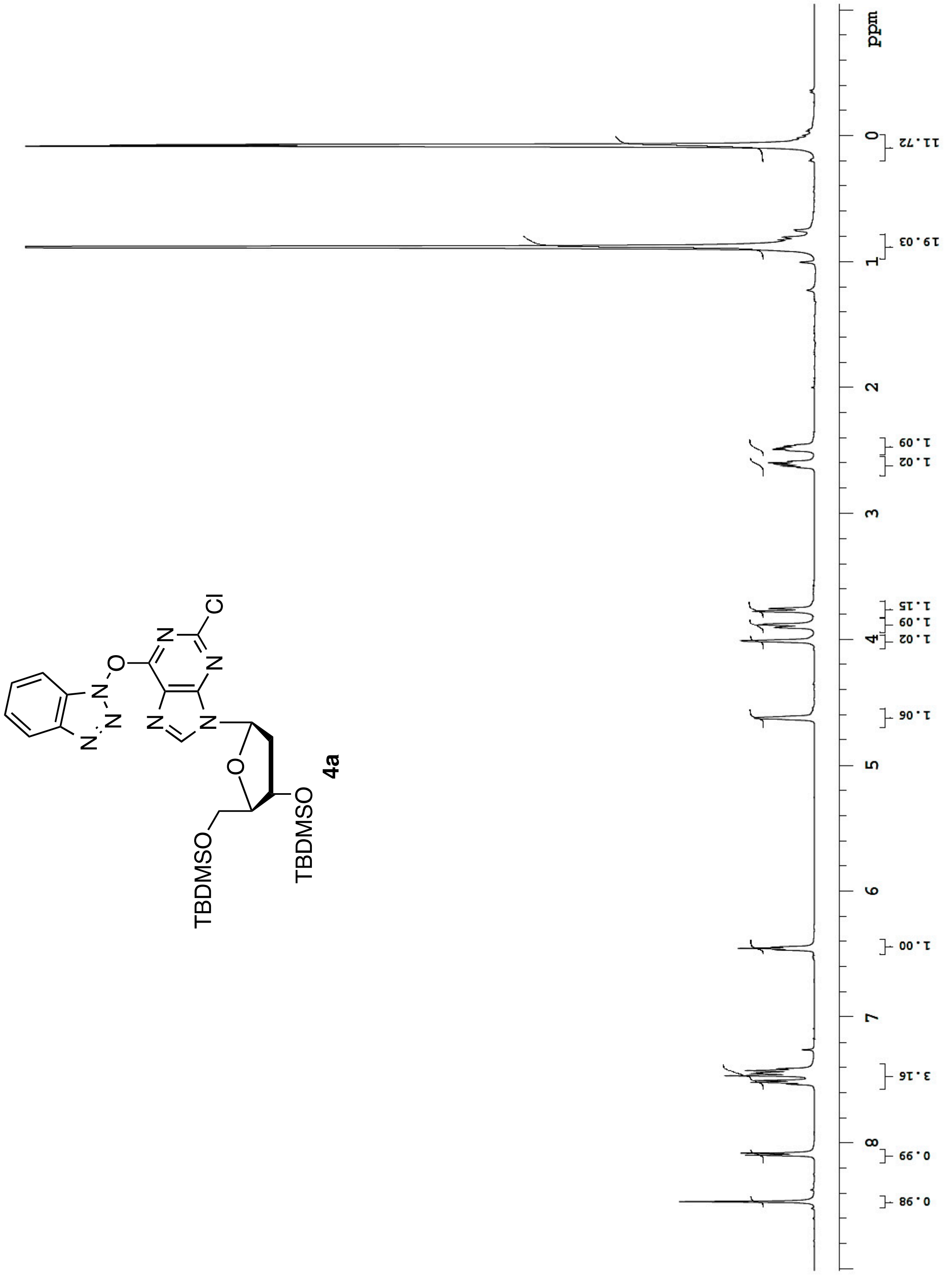
ppm
0
1
2
3
4
5
6
7
8
11.72
19.03
1.09
1.02
1.15
1.09
1.02
1.06
1.00
3.16
0.99
0.98
Cl
TBDMSO
TBDMSO

4a

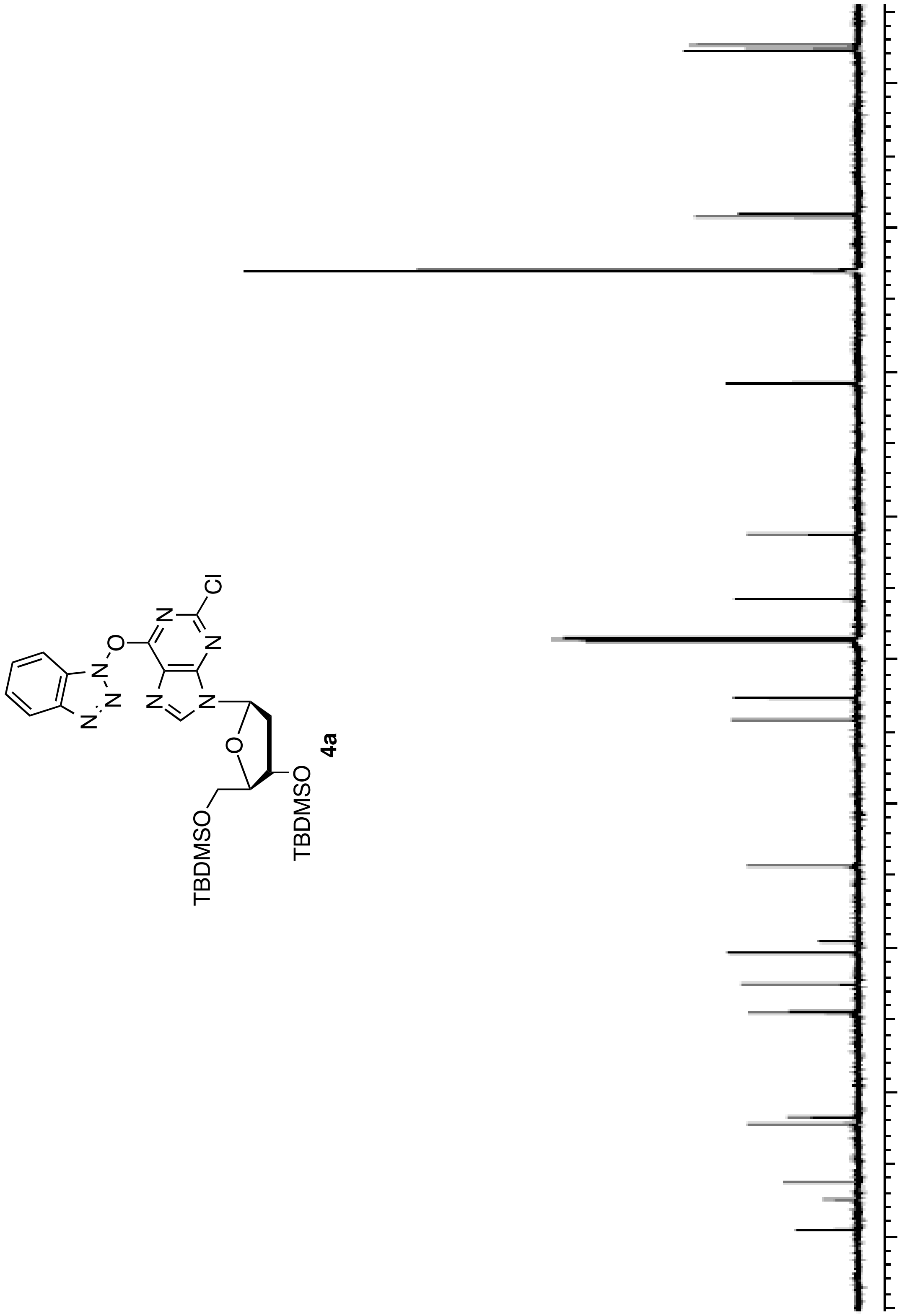
Cl
TBDMSO
TBDMSO
4a
ppm
0
20
40
60
80
100
120
140
160

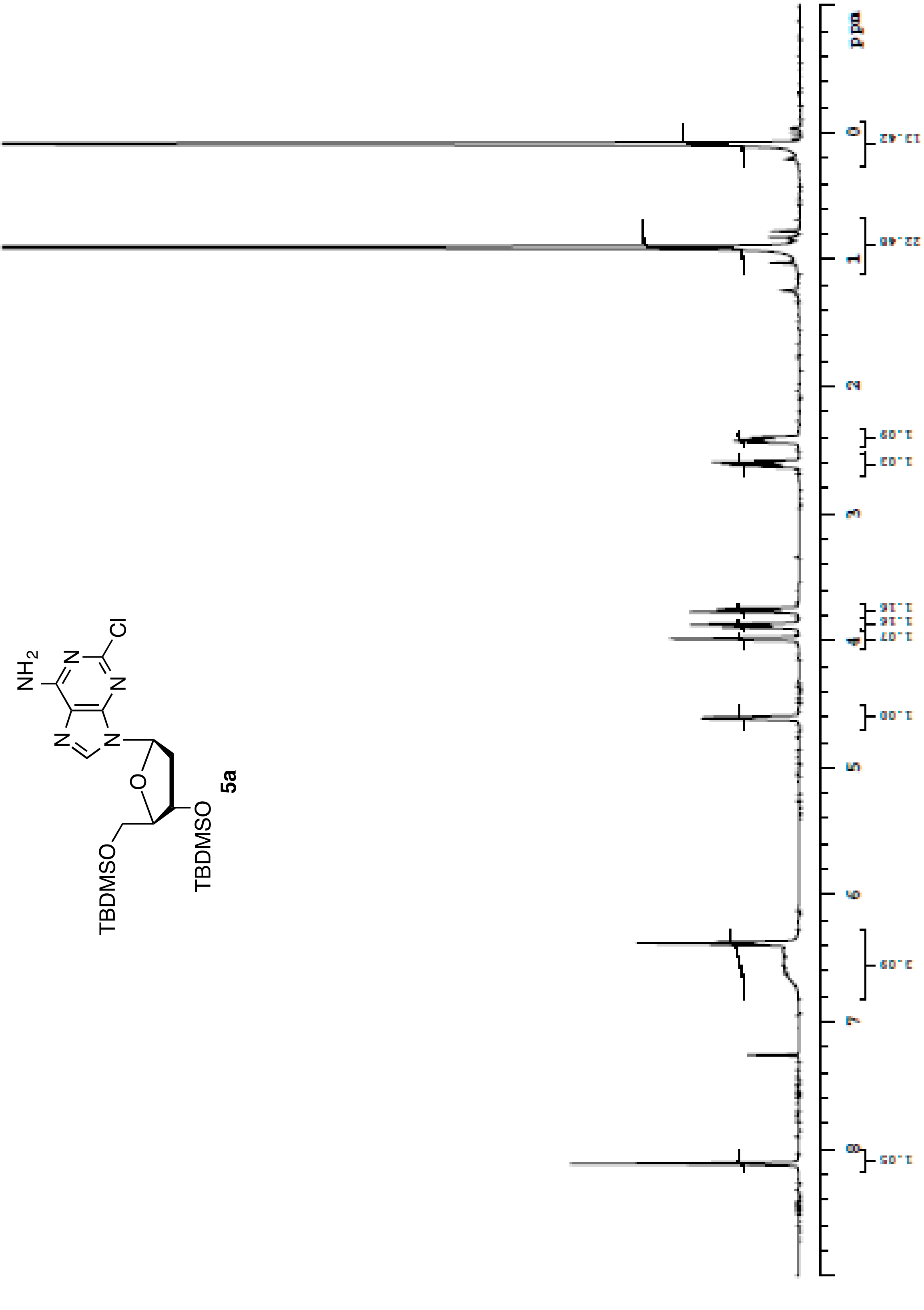

NH2
N
Cl
TBDMSO
TBDMSO
O
5a
ppm
0
1
2
3
4
5
6
7
8

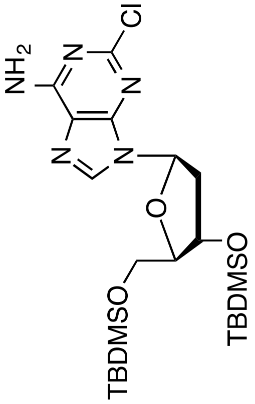

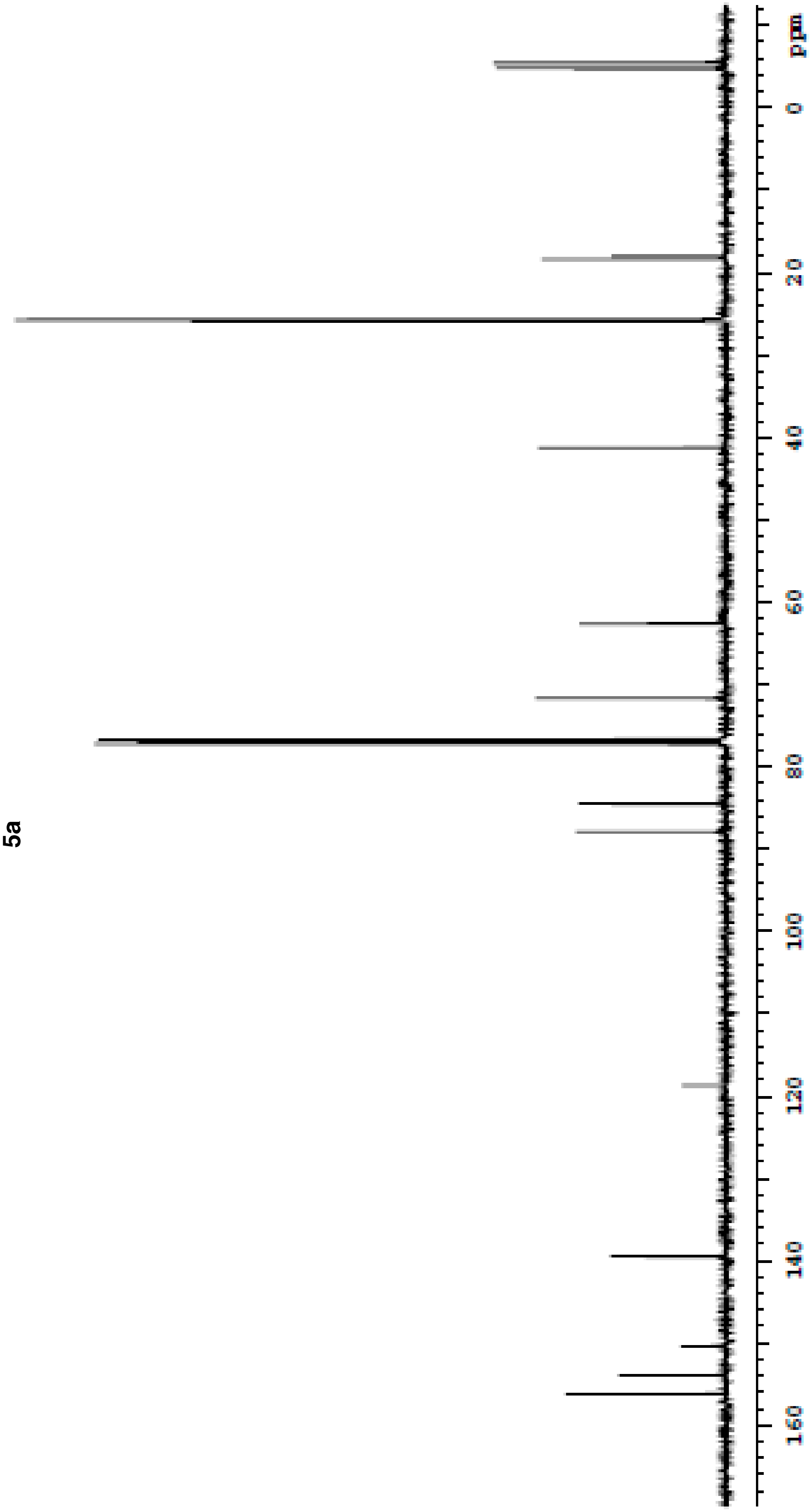

5a

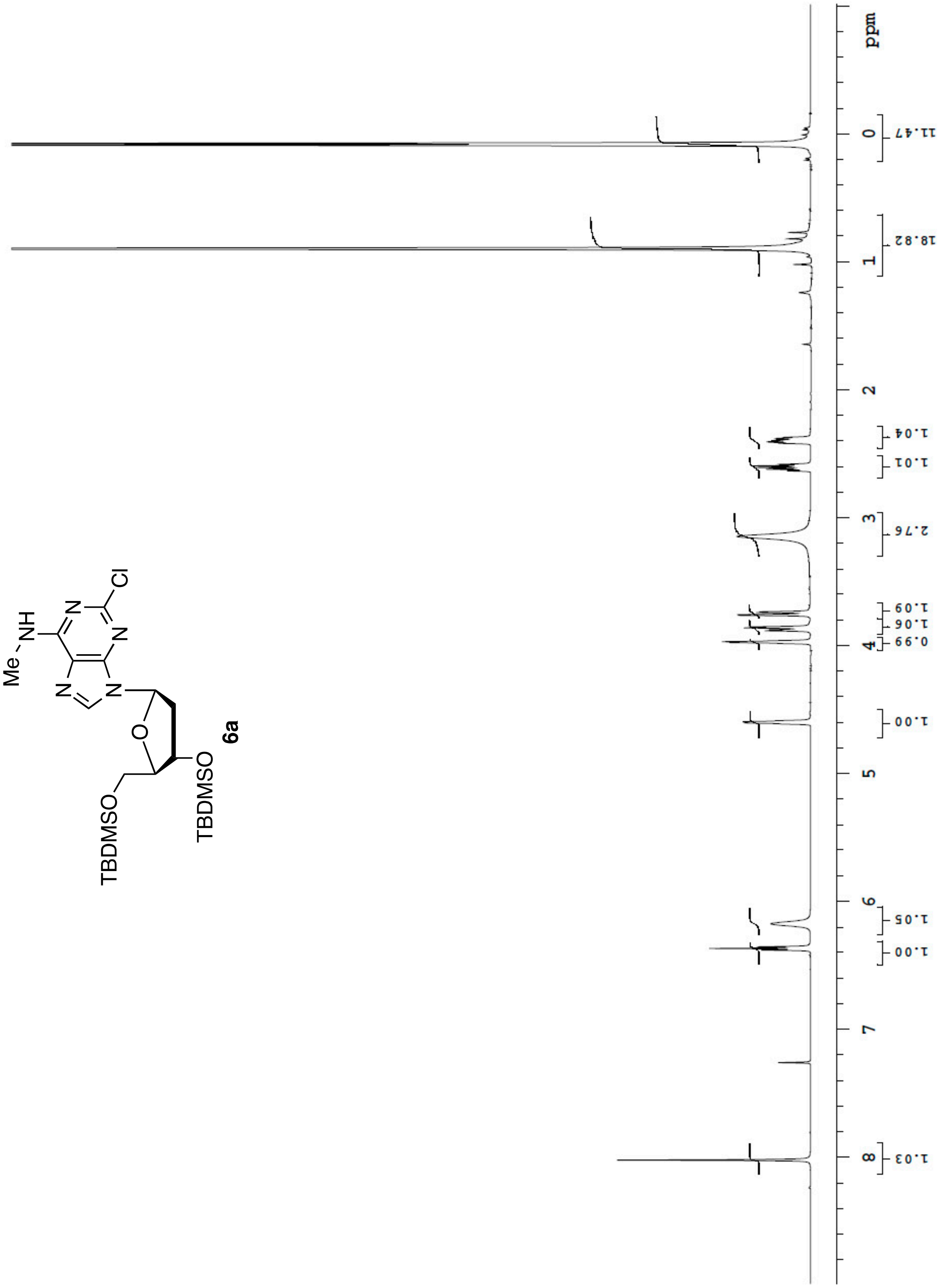

Me
NH
N
Cl
N
N
N
O
TBDMSO
TBDMSO
6a
ppm
0
1
2
3
4
5
6
7
8
11.47
18.82
1.04
1.01
2.76
1.09
1.06
0.99
1.00
1.05
1.00
1.03

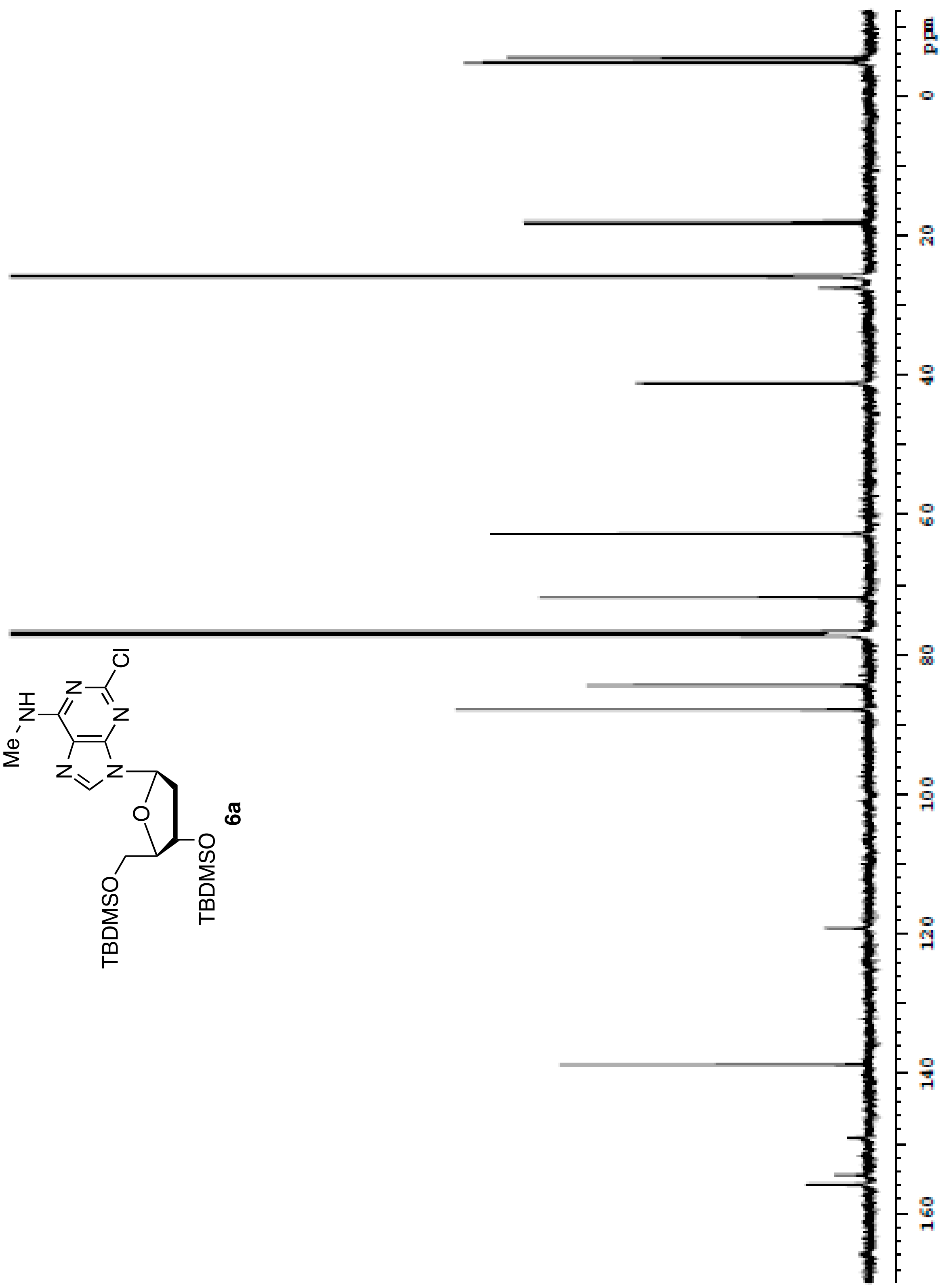

ppm
0
20
40
60
80
100
120
140
160
Me
NH
N
Cl
TBDMSO
O
6a

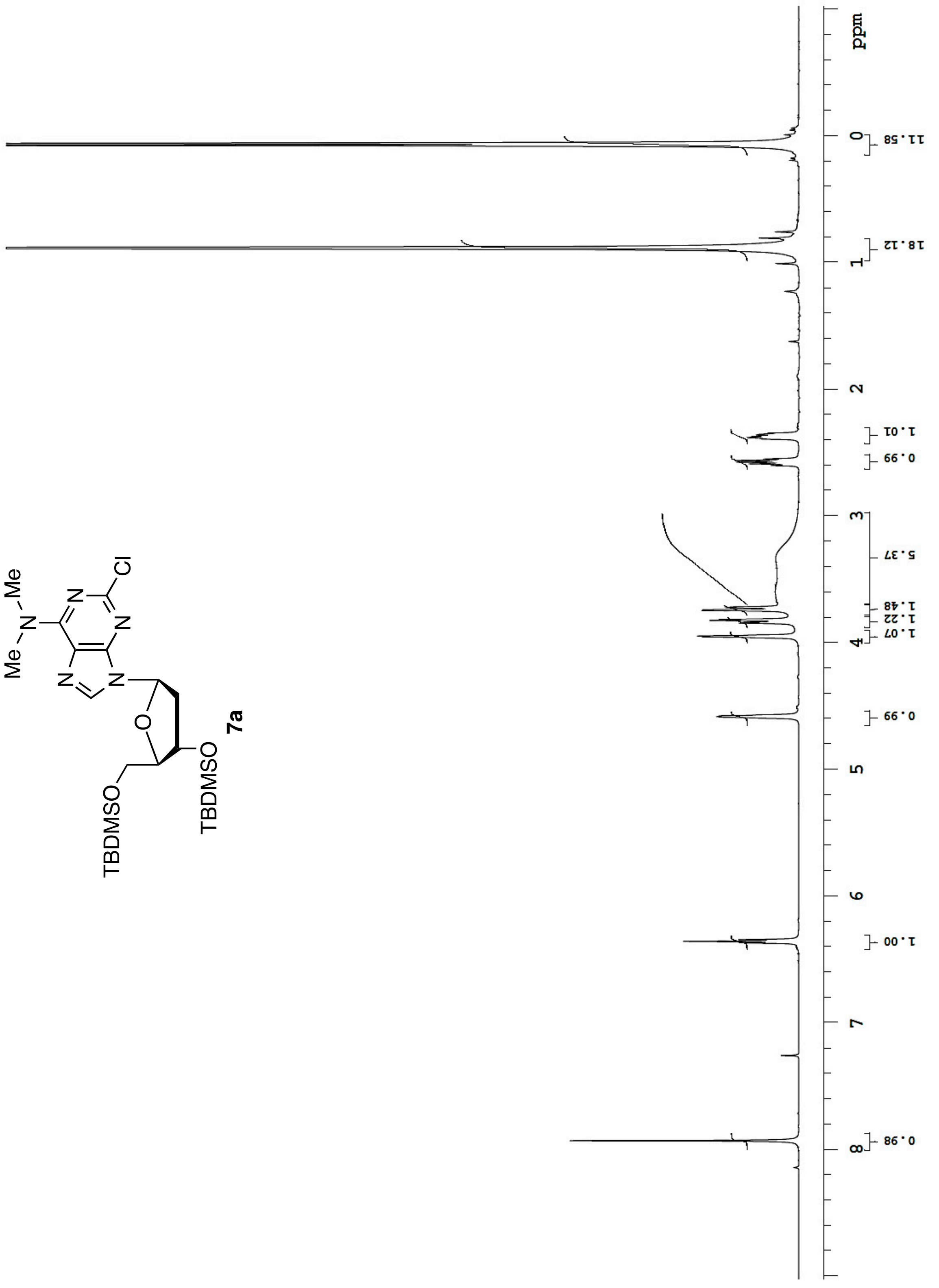
ppm
7a
TBDMSO
TBDMSO
Me
Me
Cl

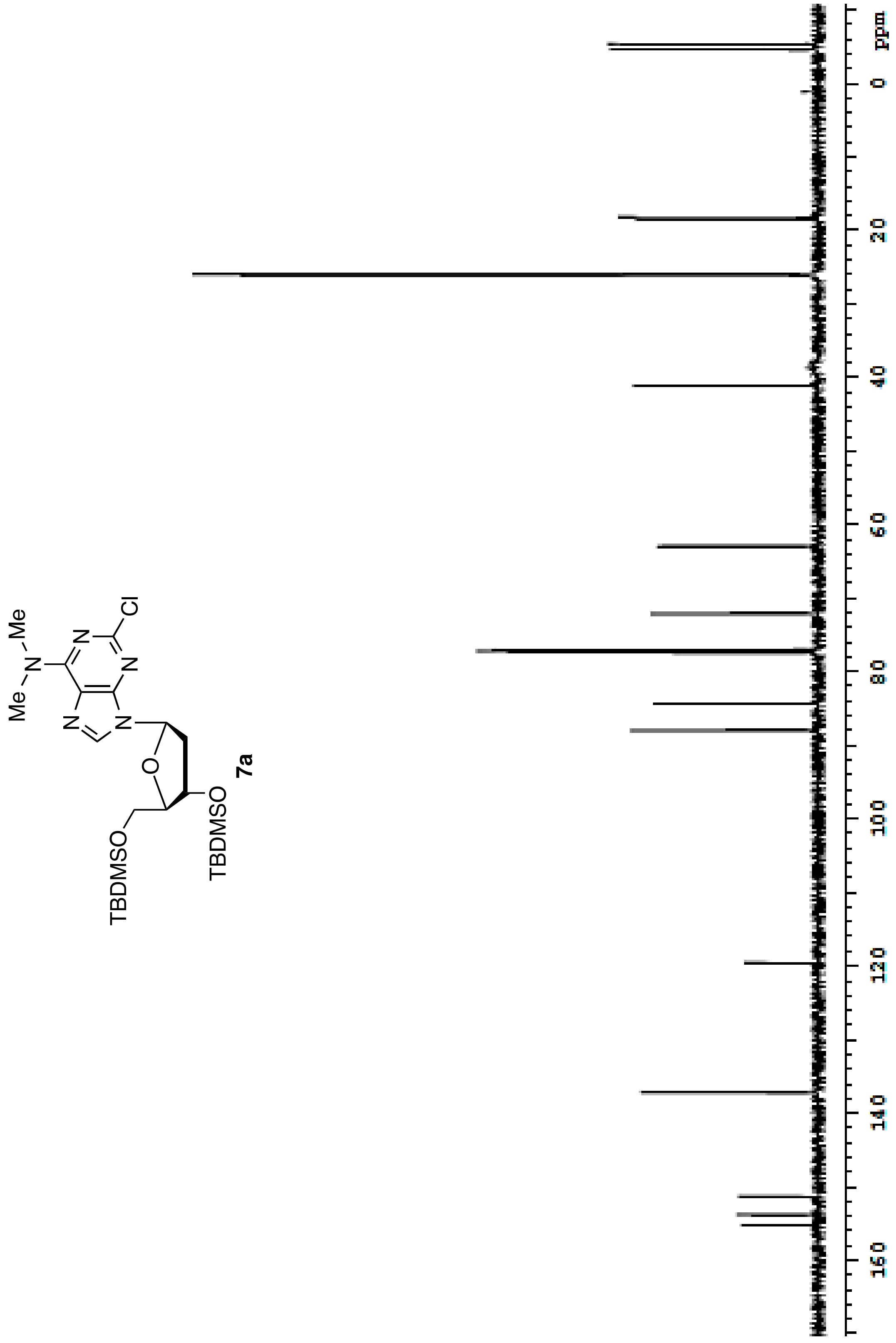
Me
Me
N
Cl
O
TBDMSO
TBDMSO
7a
ppm
0
20
40
60
80
100
120
140
160

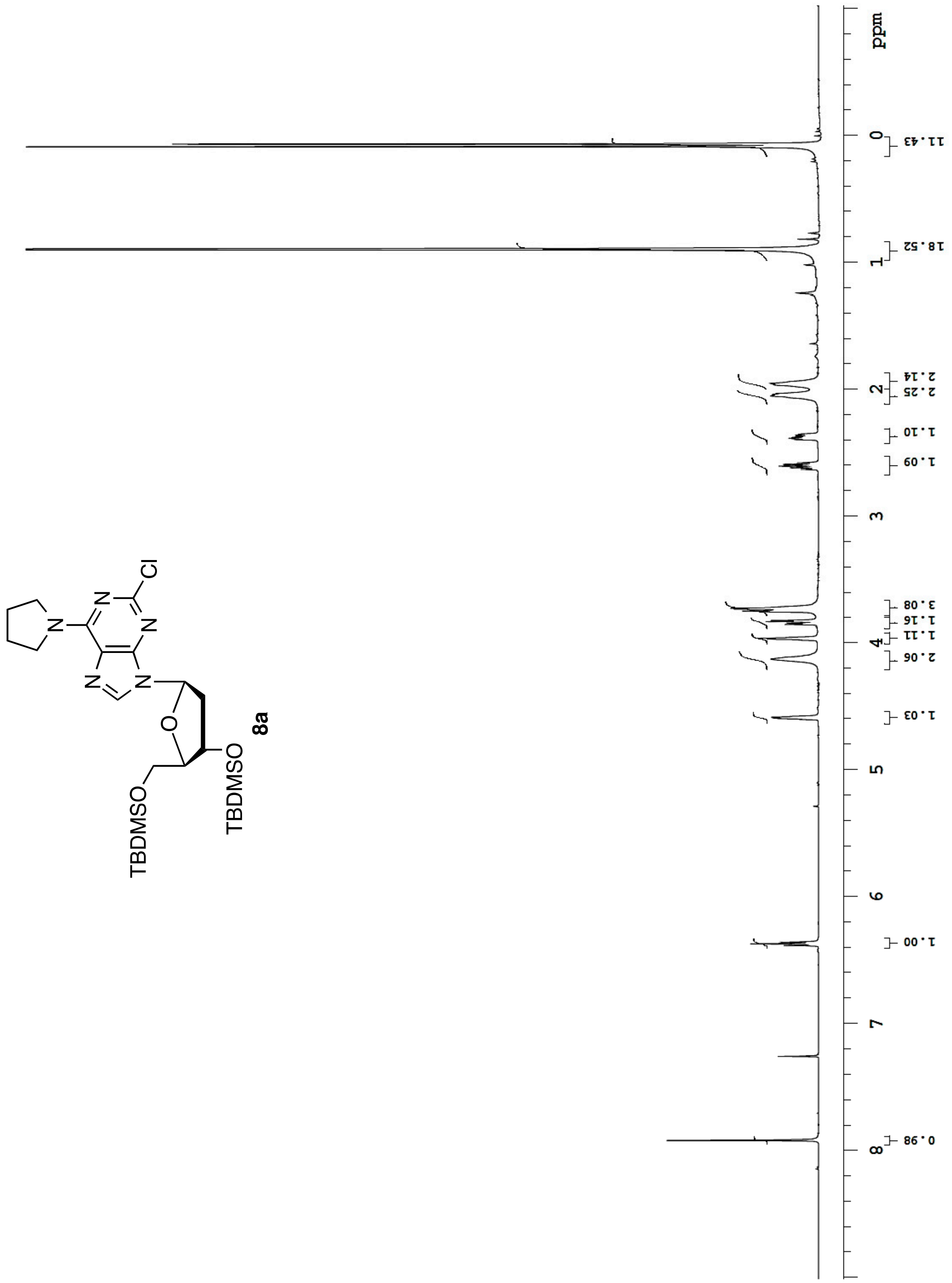

8a
TBDMSO
TBDMSO
Cl
ppm
0
1
2
3
4
5
6
7
8
11.43
18.52
2.14
2.25
1.10
1.09
3.08
1.16
1.11
2.06
1.03
1.00
0.98

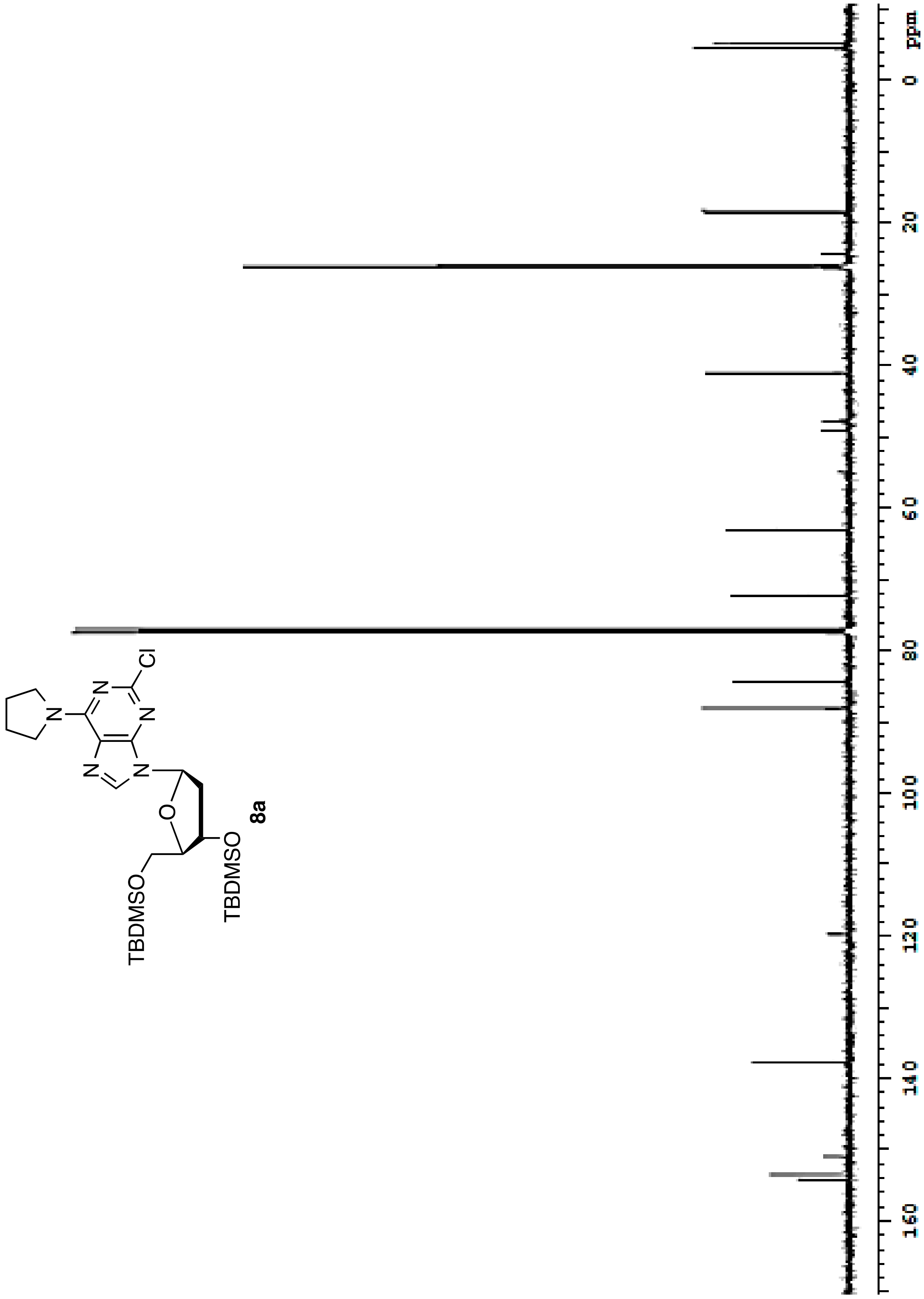

N
Cl
TBDMSO
TBDMSO
8a
ppm
0
20
40
60
80
100
120
140
160

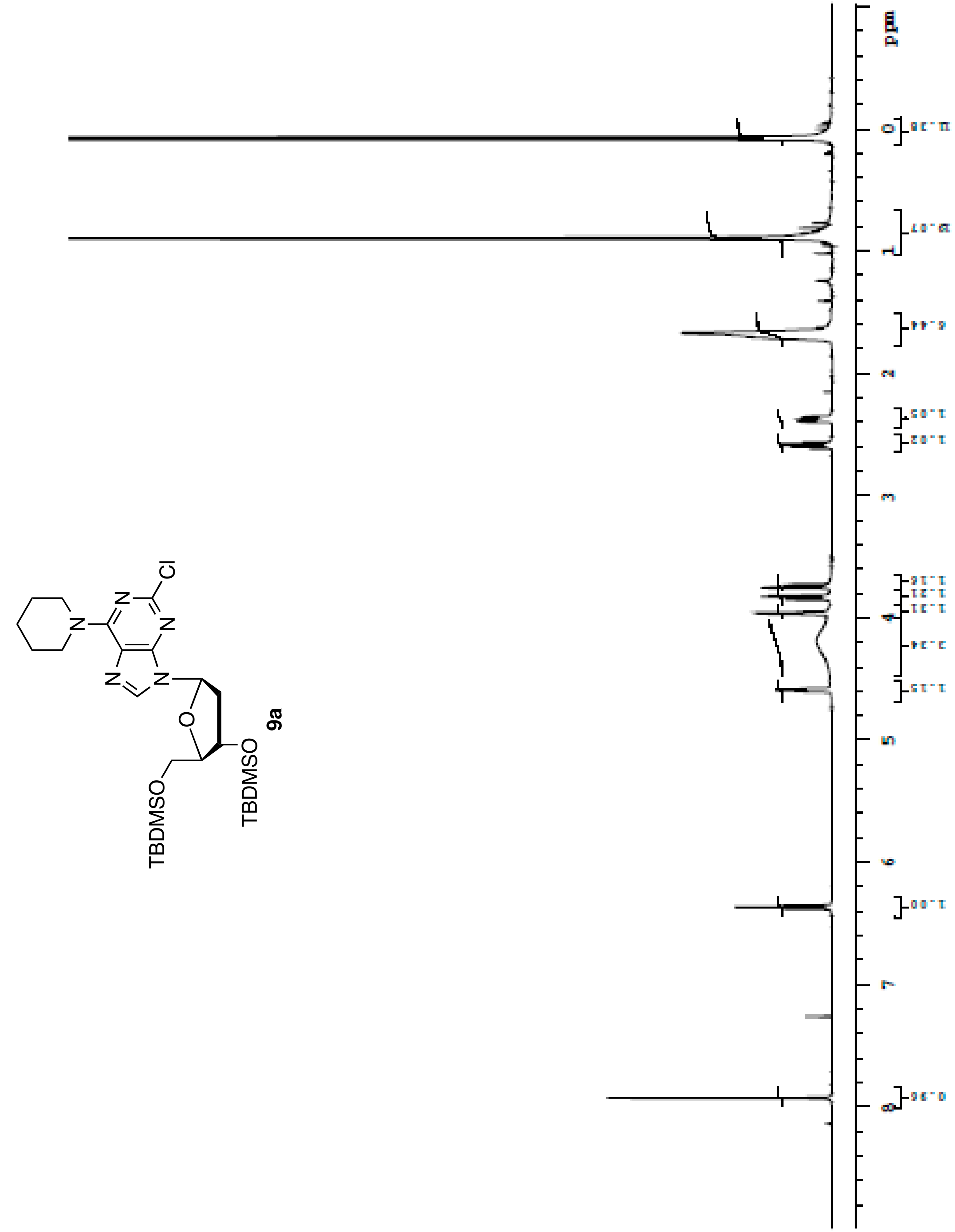

9a

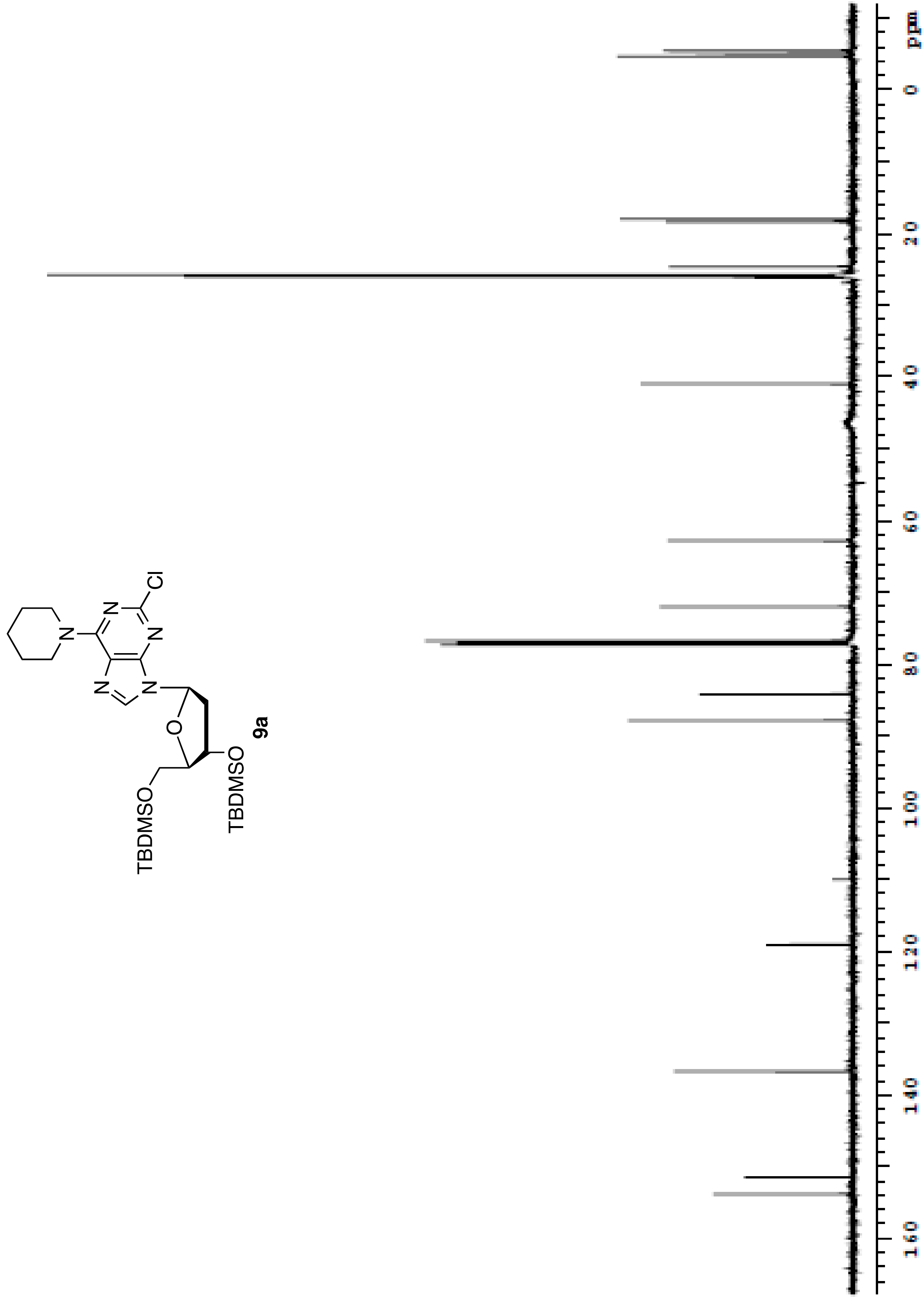
Cl
N
N
N
N
N
O
TBDMSO
TBDMSO
9a
ppm
0
20
40
60
80
100
120
140
160

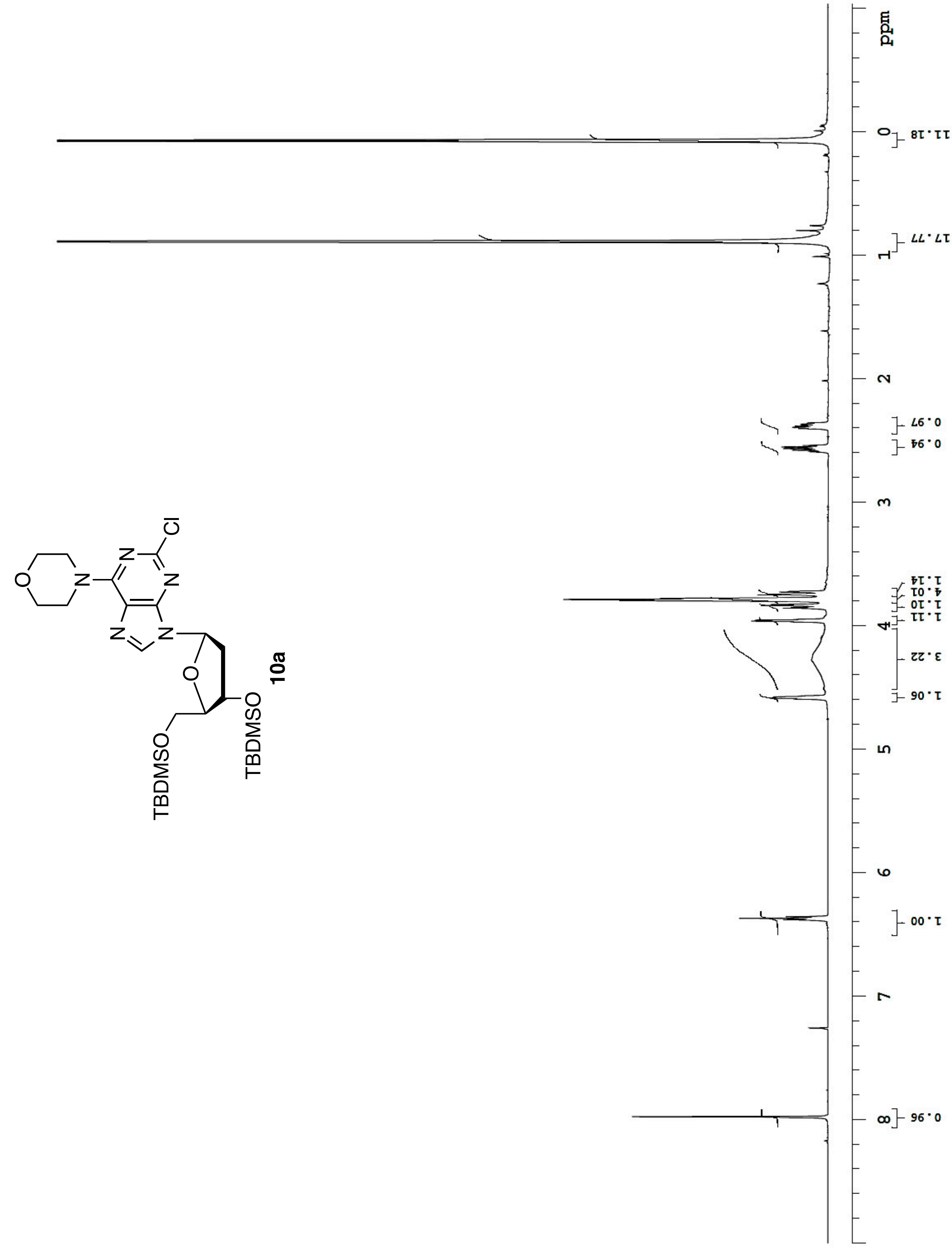
Cl
TBDMSO
TBDMSO
10a
ppm
11.18
17.77
0.97
0.94
1.14
4.01
1.10
1.11
3.22
1.06
1.00
0.96

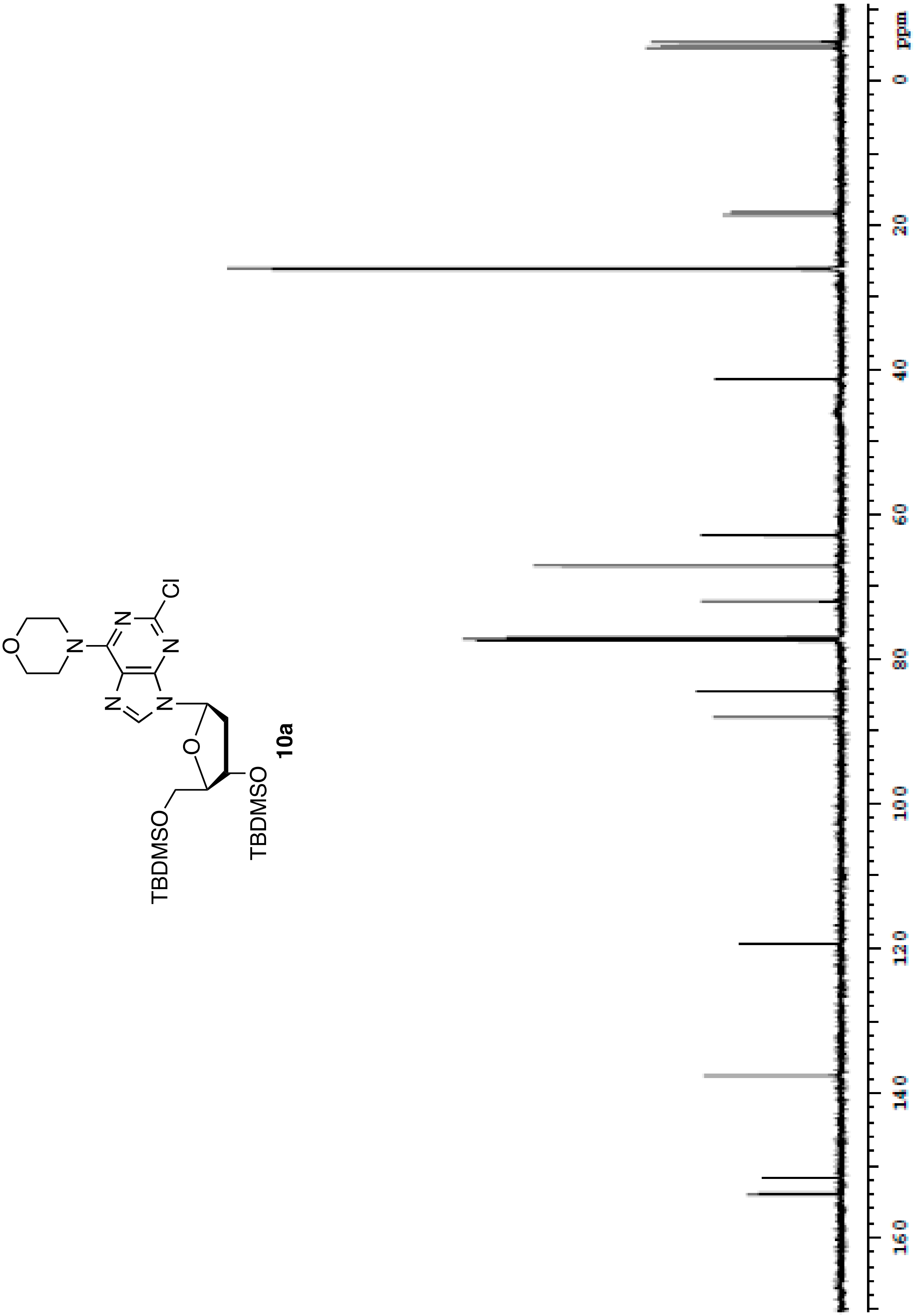
O
N
N
N
Cl
N
N
O
TBDMSO
TBDMSO
10a
ppm
0
20
40
60
80
100
120
140
160

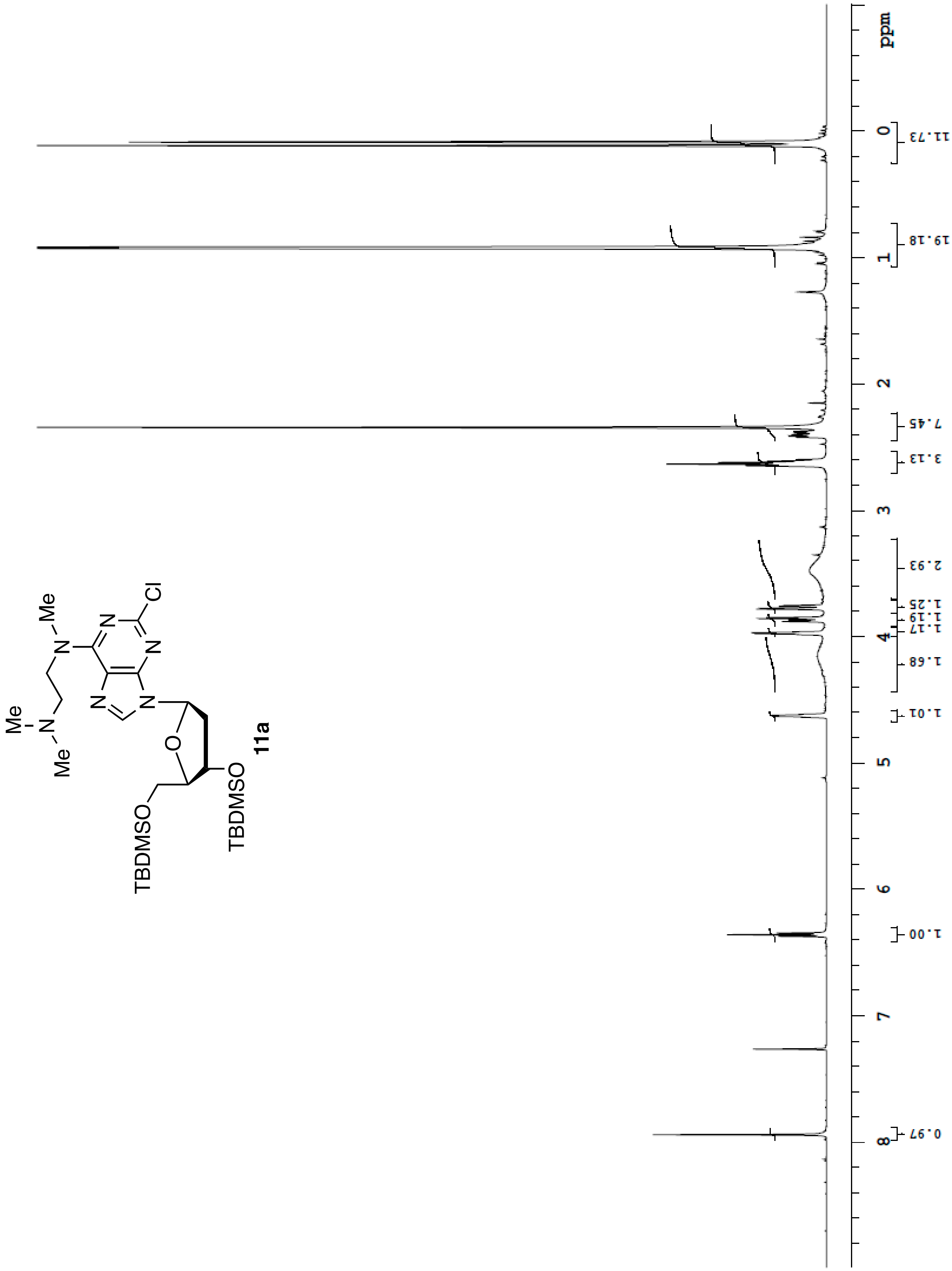
11a
TBDMSO
TBDMSO
Cl
Me
ppm
11.73
19.18
7.45
3.13
2.93
1.25
1.19
1.17
1.68
1.01
1.00
0.97

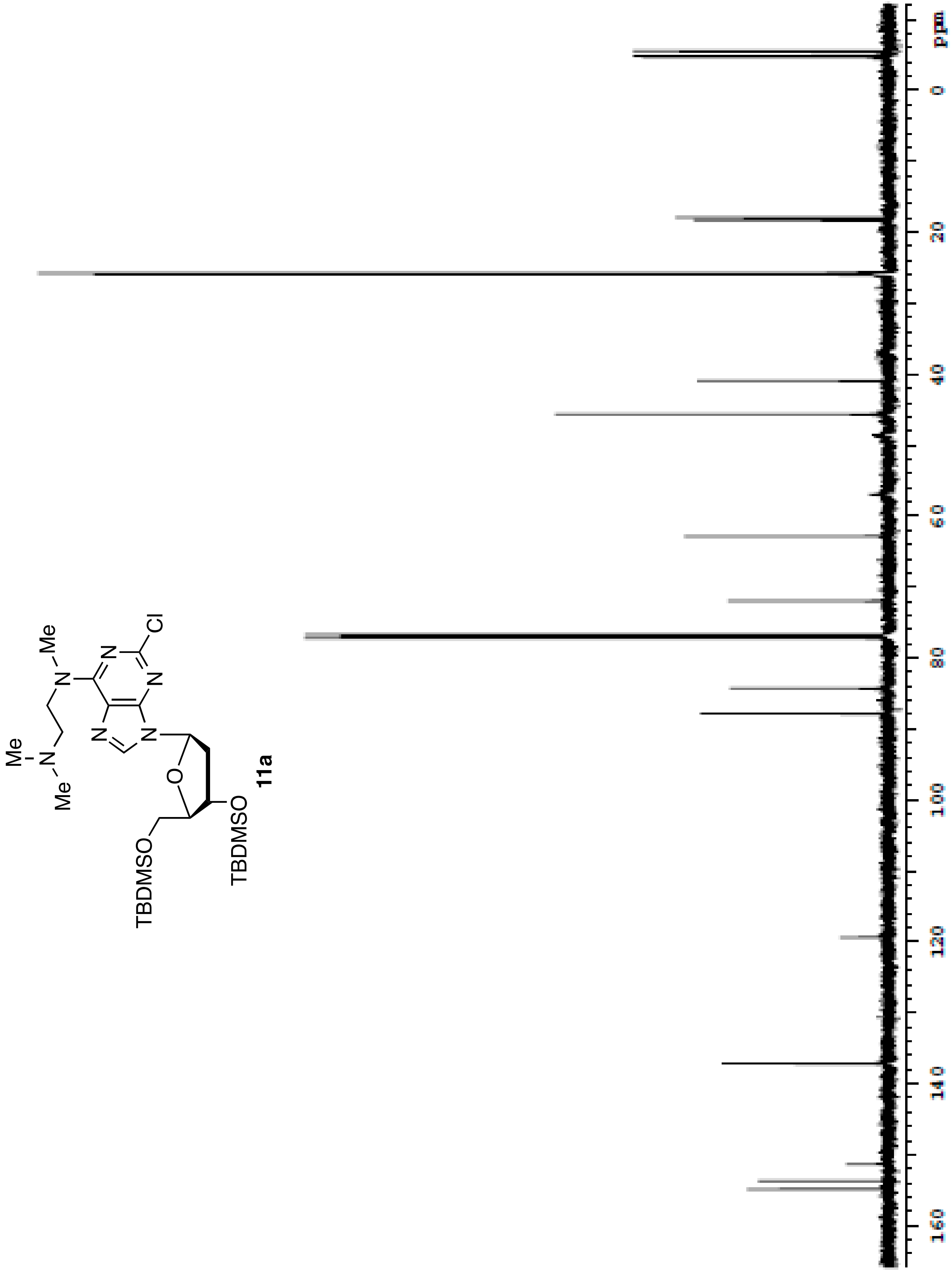
Me
N
Cl
TBDMSO
O
11a
ppm
0
20
40
60
80
100
120
140
160

Sample Name :
GVK-DD0397-CHLORO

Solvent: cdcl3
Date: Mar 22 2015
400 VNMRS/NRM-1/NMR-002

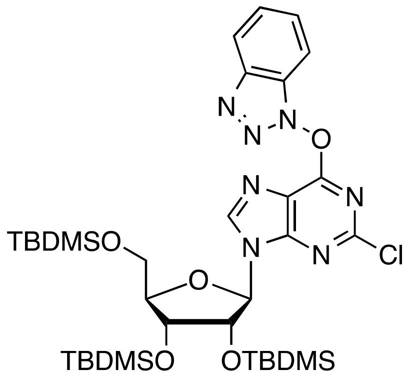

4b

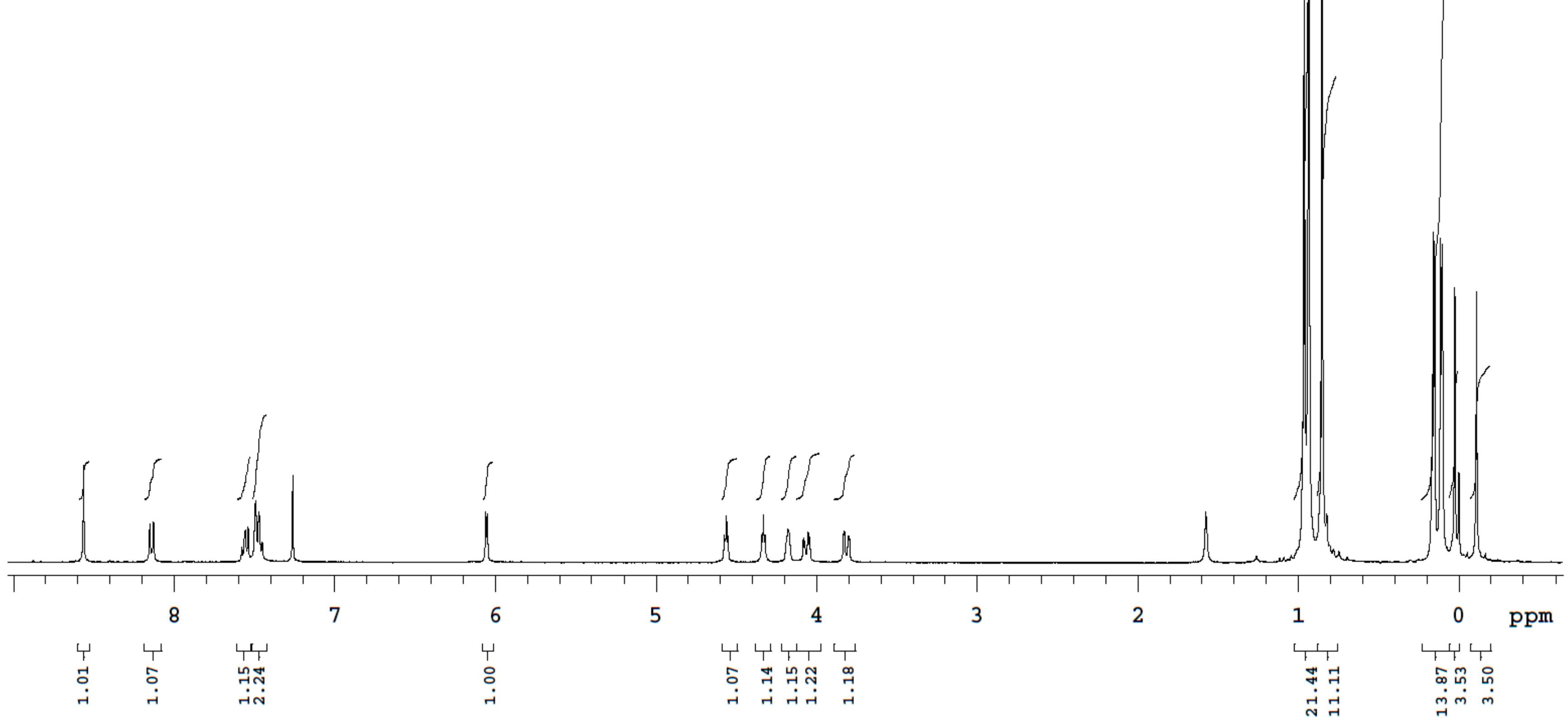

Plotname: 021503C284_PROTON_01_plot02

Sample Name :
GVK-DD0397-CHLORO

Solvent: cdcl3
Date: Mar 22 2015
400 VNMRS/NRM-1/NMR-002

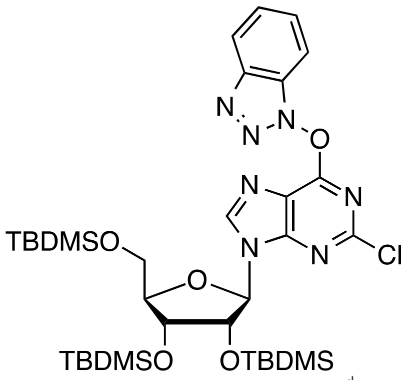

4b

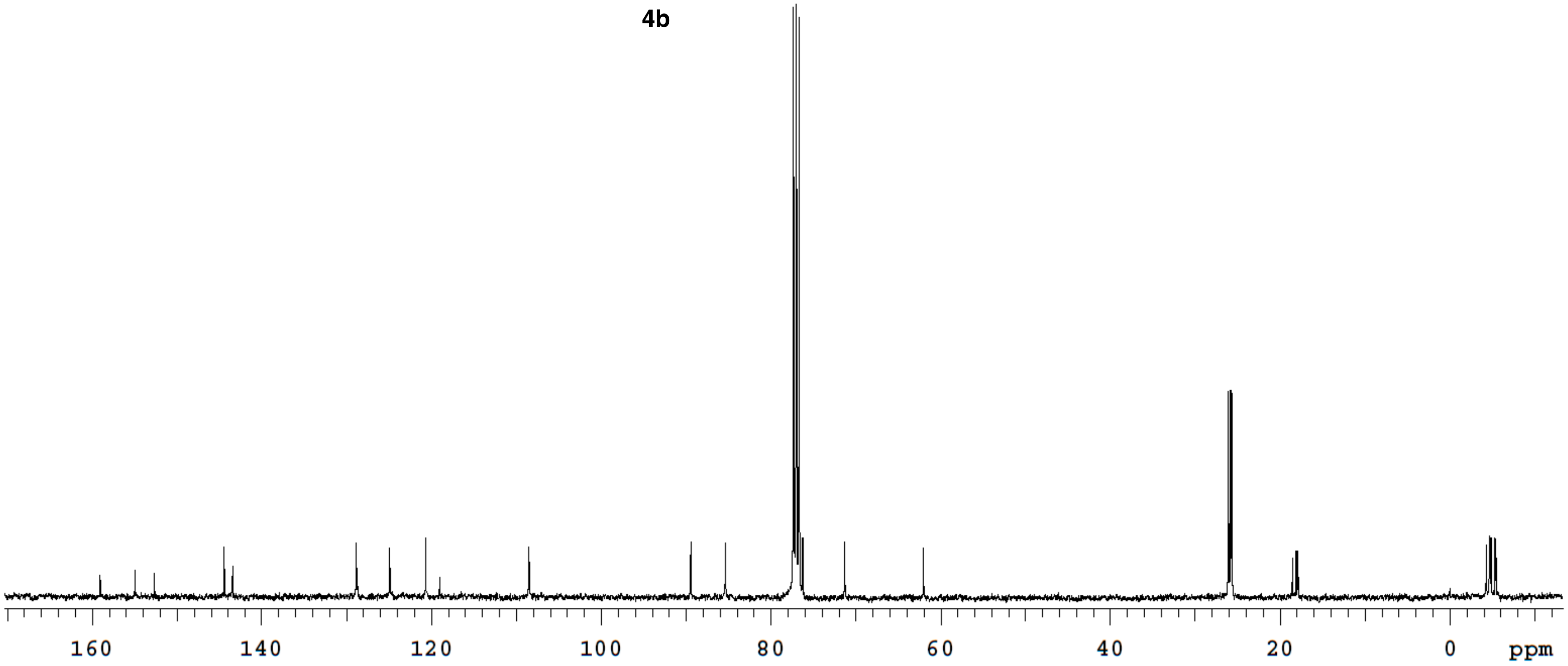

Plotname: 021503C2184_CARBON_01_plot01

Sample Name :
GVK-DD0397-E109832-A
Solvent: cdcl3
400-MR/NRM-1/NMR-001
Date: Feb 27 2015

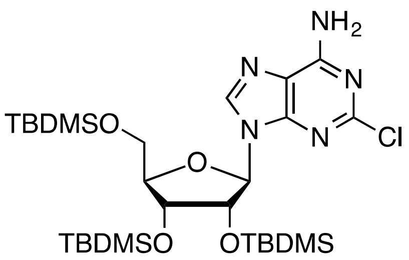

5b

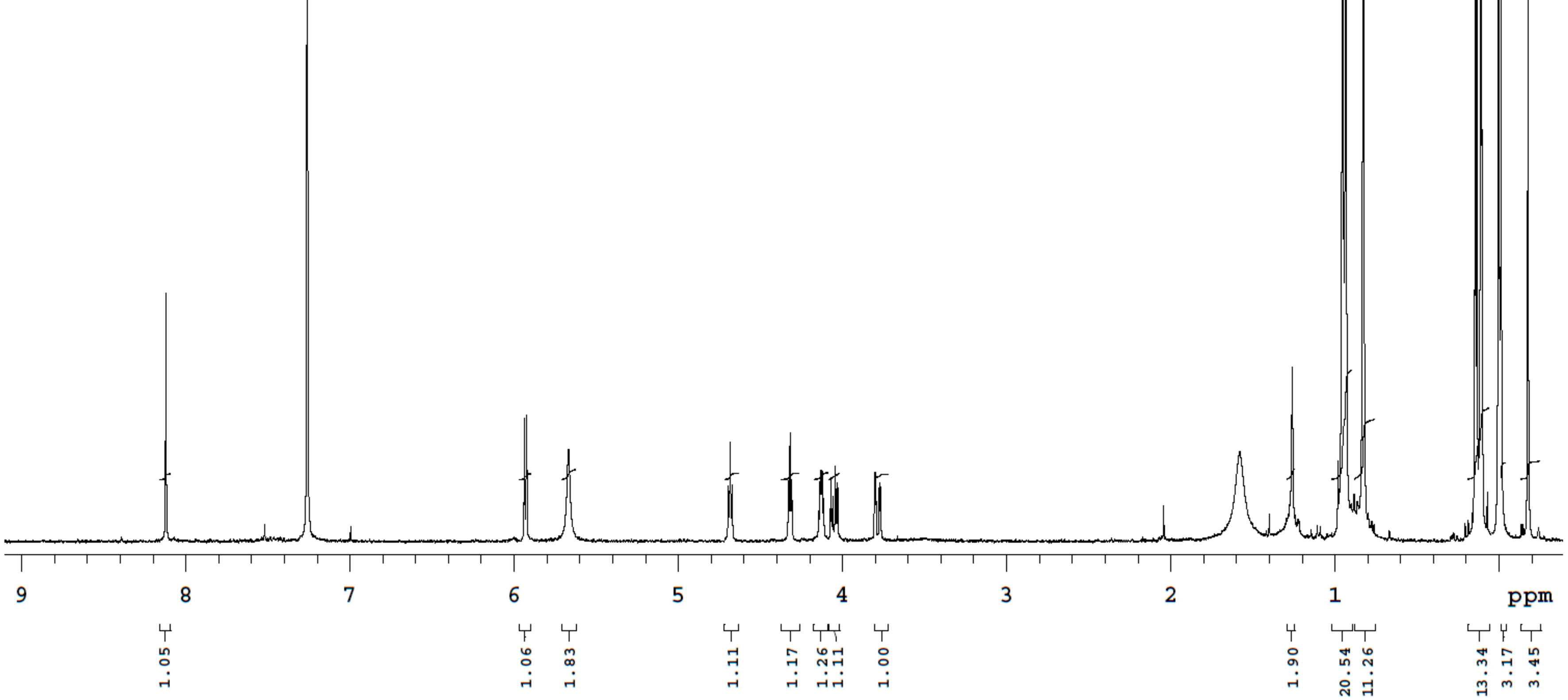

Plotname: 021502D3602_PROTON_01_plot08

Sample Name :
GVK-DD0-397-AMM-T

Solvent: cdcl3
Date: Mar 28 2015
400 VNMRS/NRM-1/NMR-002

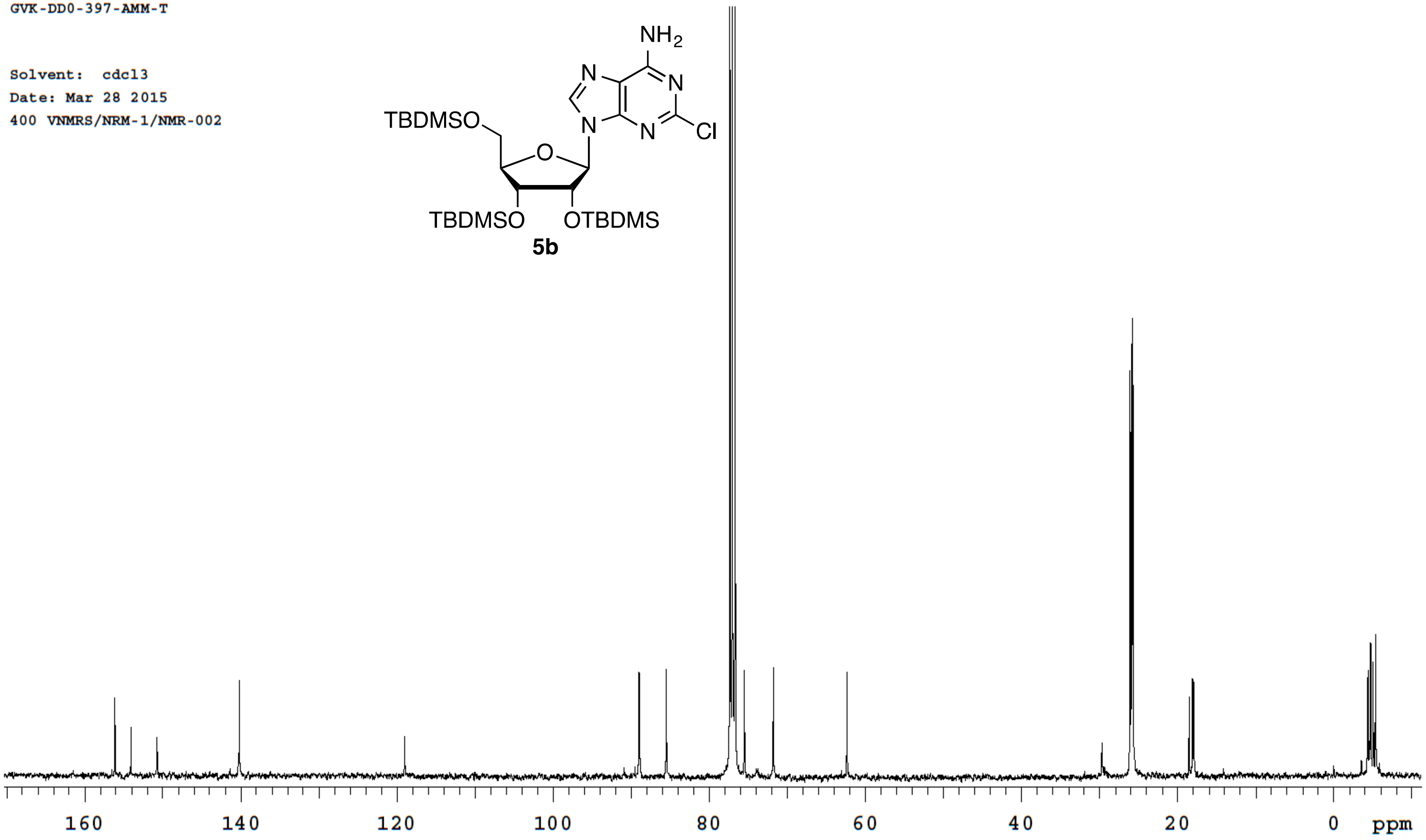

Plotname: 021503D2572_CARBON_01_plot01

Sample Name :
GVK-DD0397-ME-T
Solvent: cdcl3
400-MR/NRM-1/NMR-001
Date: Mar 29 2015

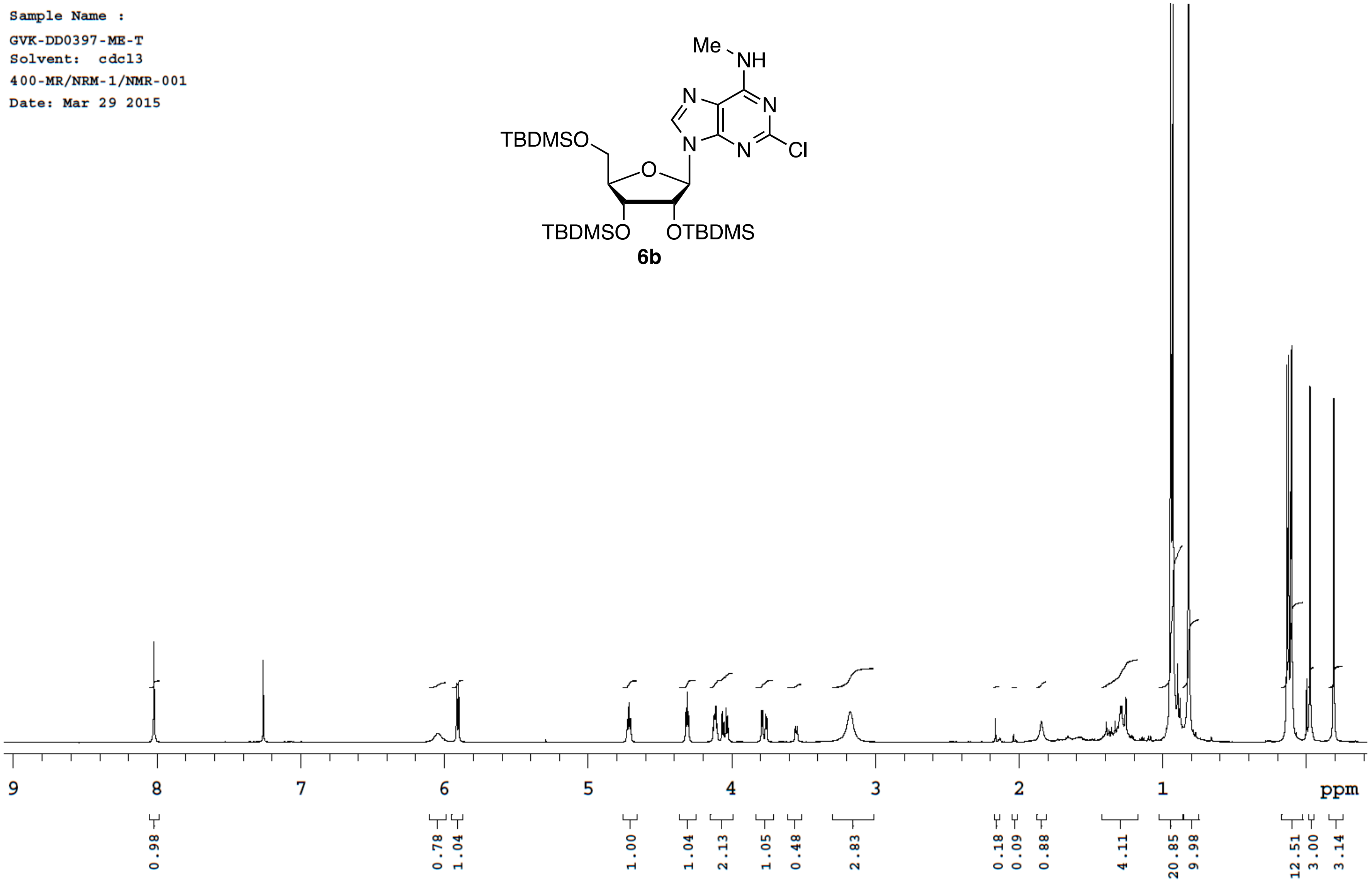

6b

Plotname: 021503D2577_PROTON_01_plot07

Sample Name :
GVK-DD0397-ME-T
Solvent: cdcl3
400-MR/NRM-1/NMR-001
Date: Mar 29 2015

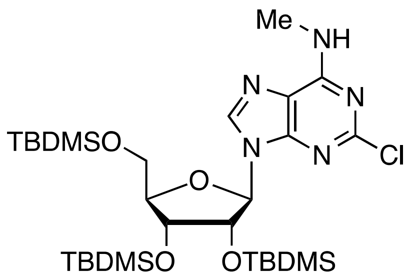

6b

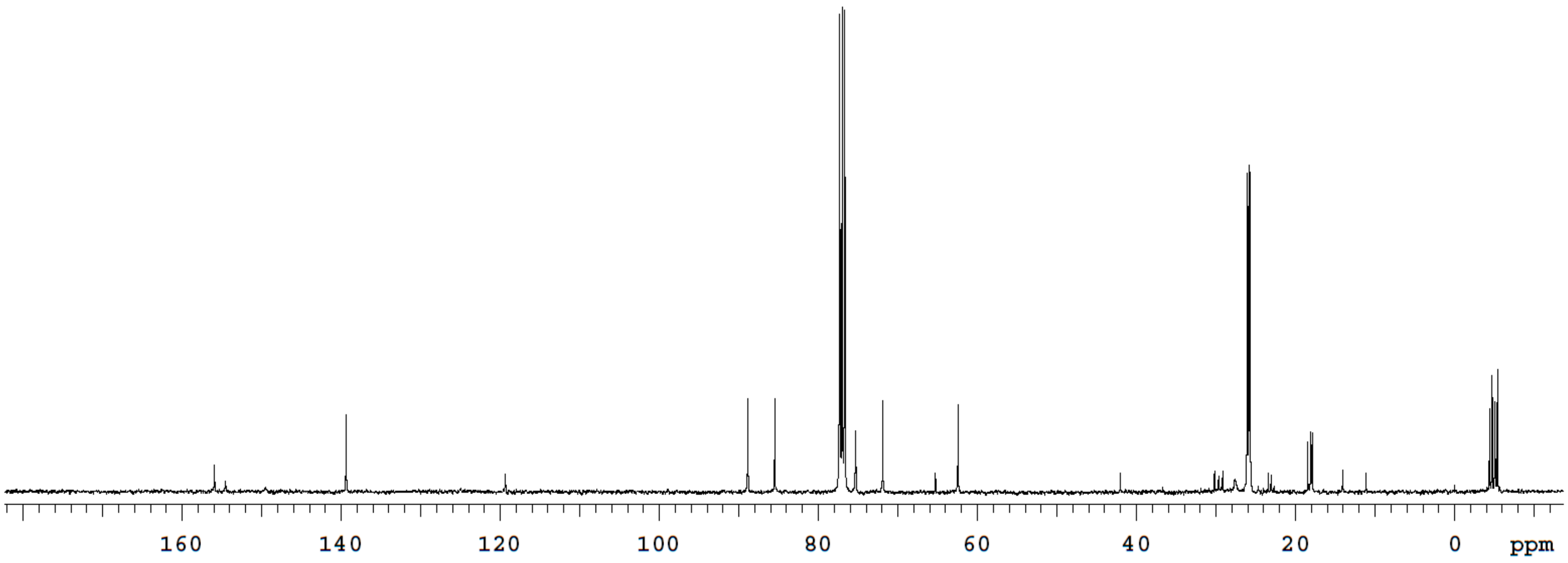

Plotname: 021503D2577_CARBON_01_plot01

Sample Name :
GVK-DD0-397-DME-T

Solvent: cdcl3
Date: Mar 28 2015
400 VNMRS/NRM-1/NMR-002

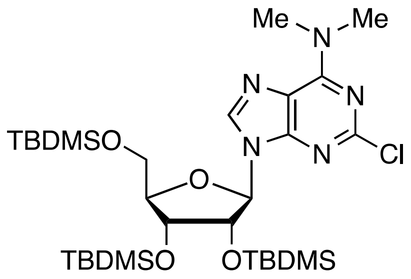

7b

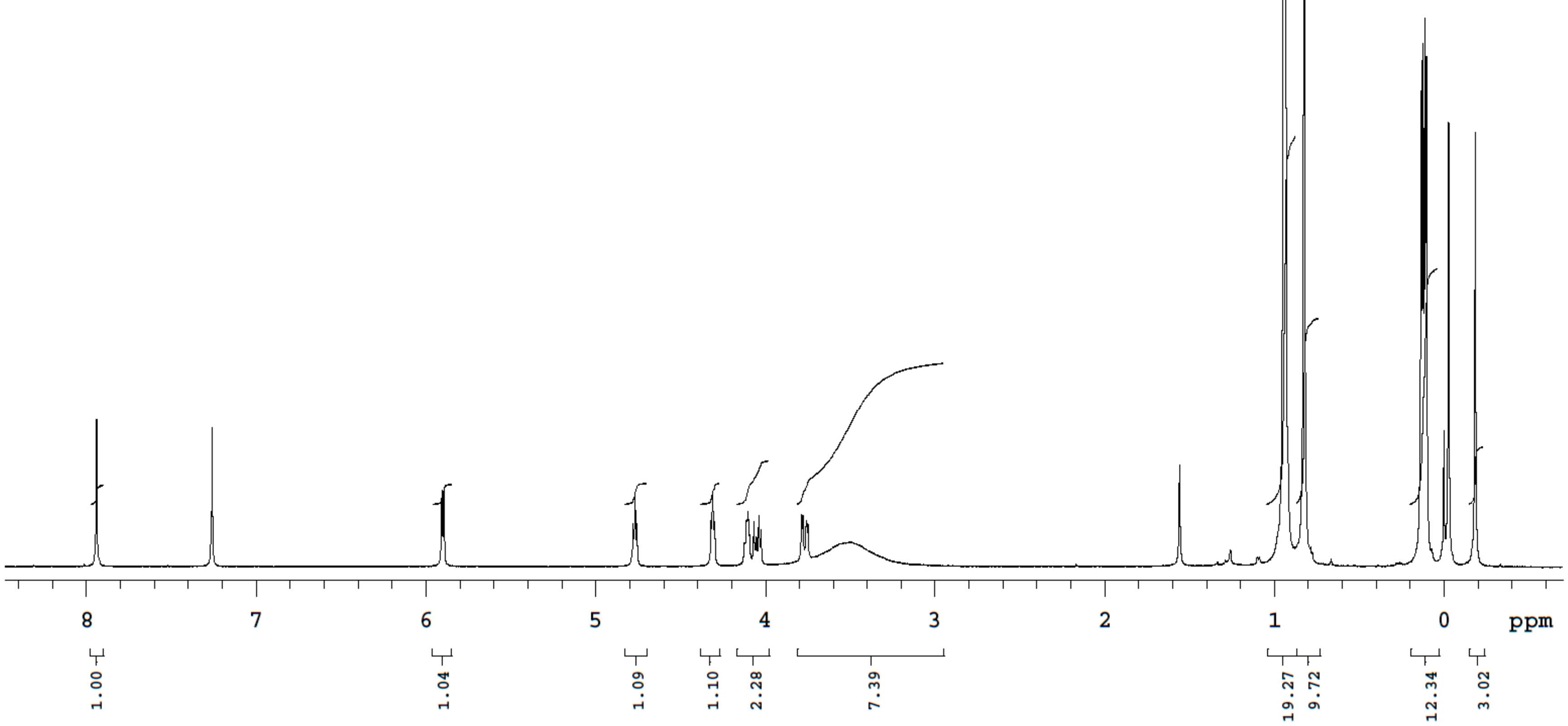

Plotname: 021503D2595_PROTON_01_plot08

Sample Name :
GVK-DD0-397-DME-T

Solvent: cdcl3
Date: Mar 28 2015
400 VNMRS/NRM-1/NMR-002

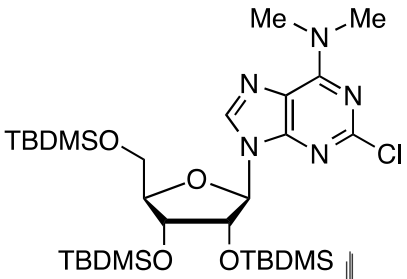

7b

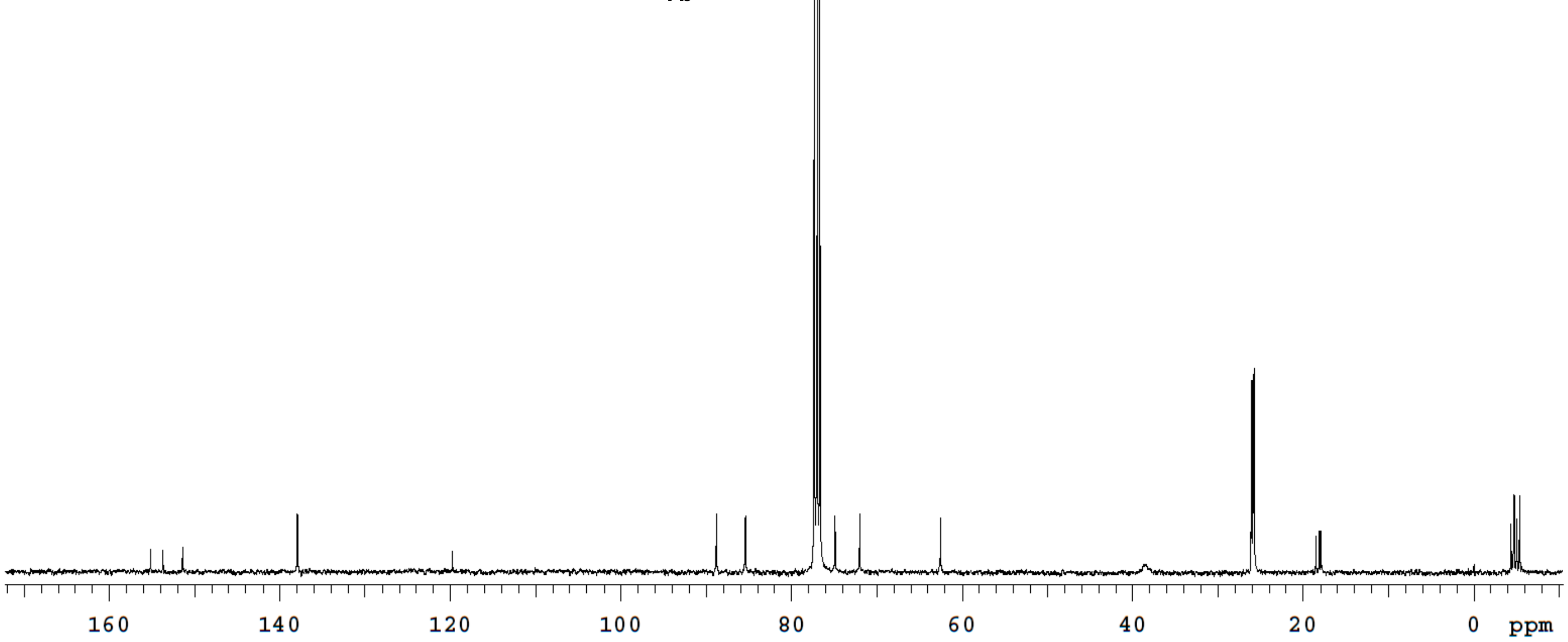

Plotname: 021503D2595_CARBON_01_plot01

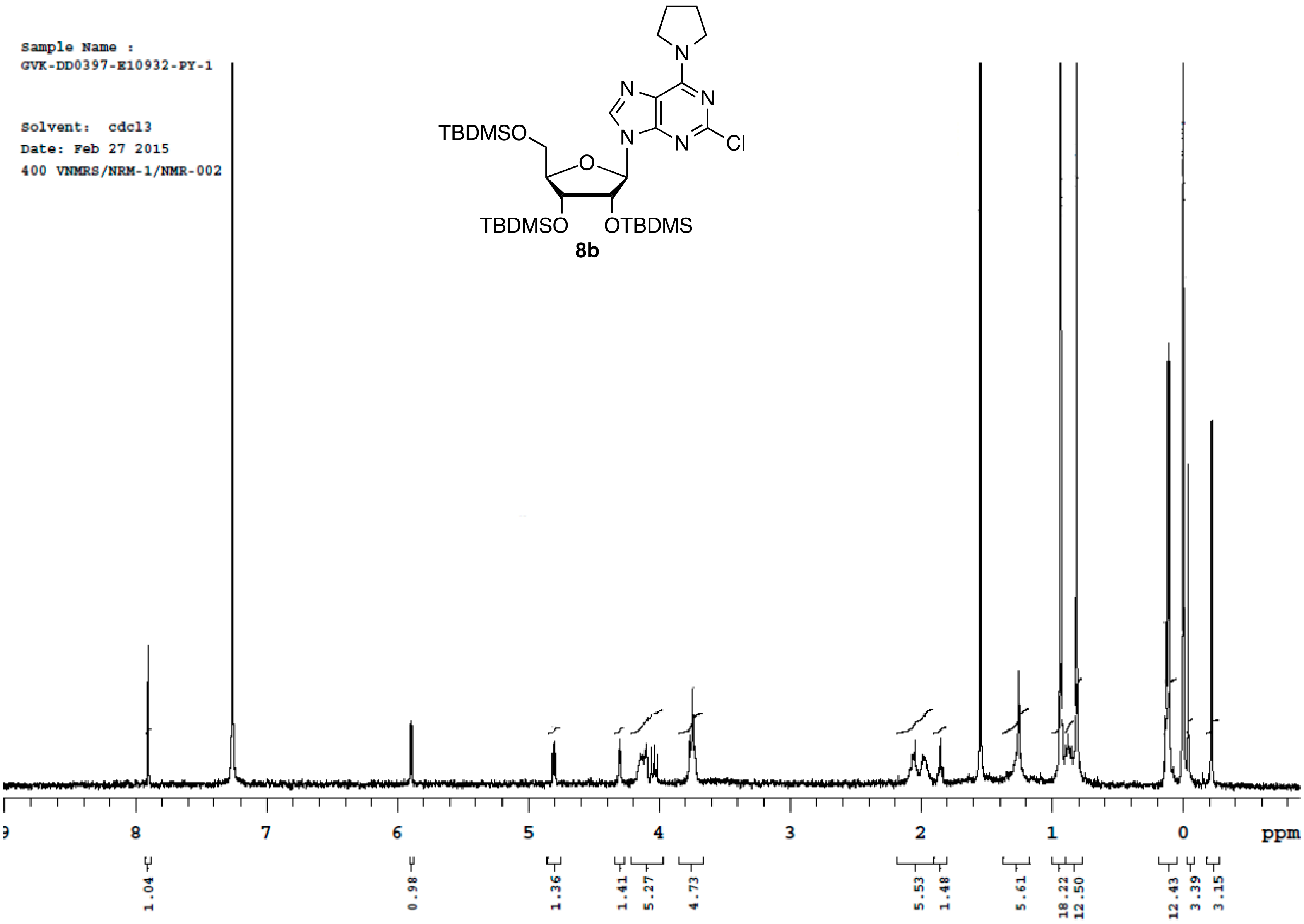
Sample Name :
GVK-DD0397-E10932-PY-1
Solvent: cdcl3
Date: Feb 27 2015
400 VNMRS/NRM-1/NMR-002
TBDMSO
N
N
N
N
N
Cl
O
TBDMSO
OTBDMS
8b
9
8
7
6
5
4
3
2
1
0
ppm
1.04
0.98
1.36
1.41
5.27
4.73
5.53
1.48
5.61
18.22
12.50
12.43
3.39
3.15

Sample Name :
GVK-DD0397-PYRROLIDINE
Solvent: cdcl3
400-MR/NRM-1/NMR-001
Date: Apr 25 2015

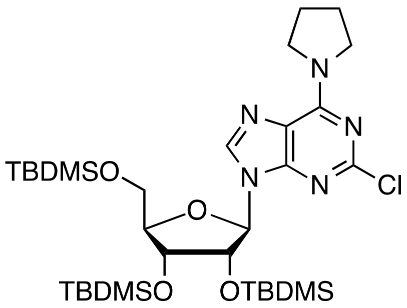

8b

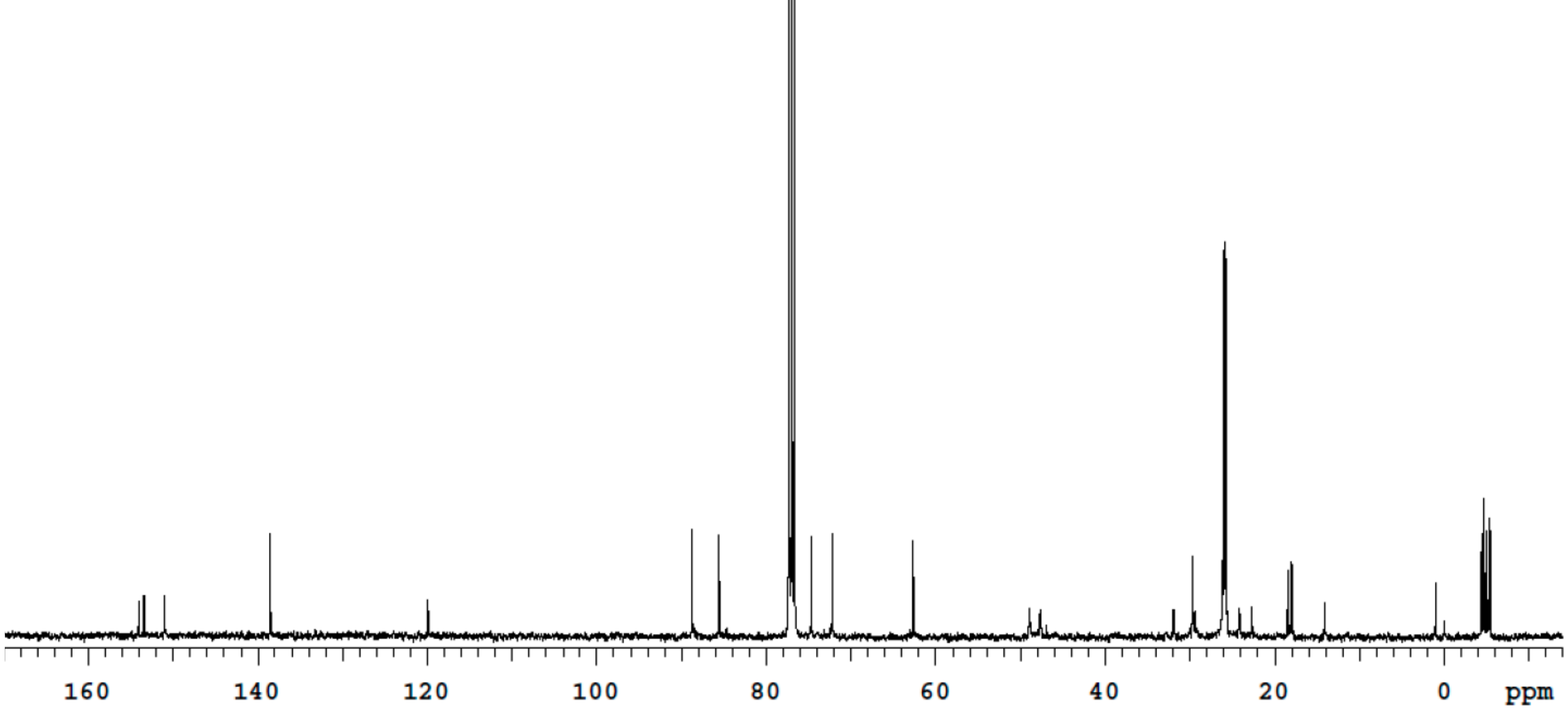

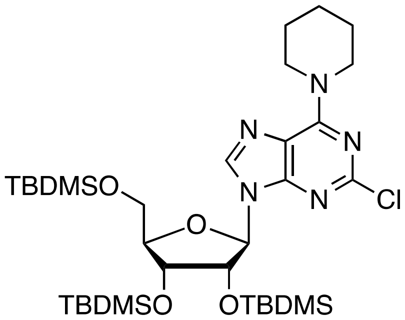
TBDMSO
N
N
N
N
N
Cl
O
TBDMSO
OTBDMS

9b

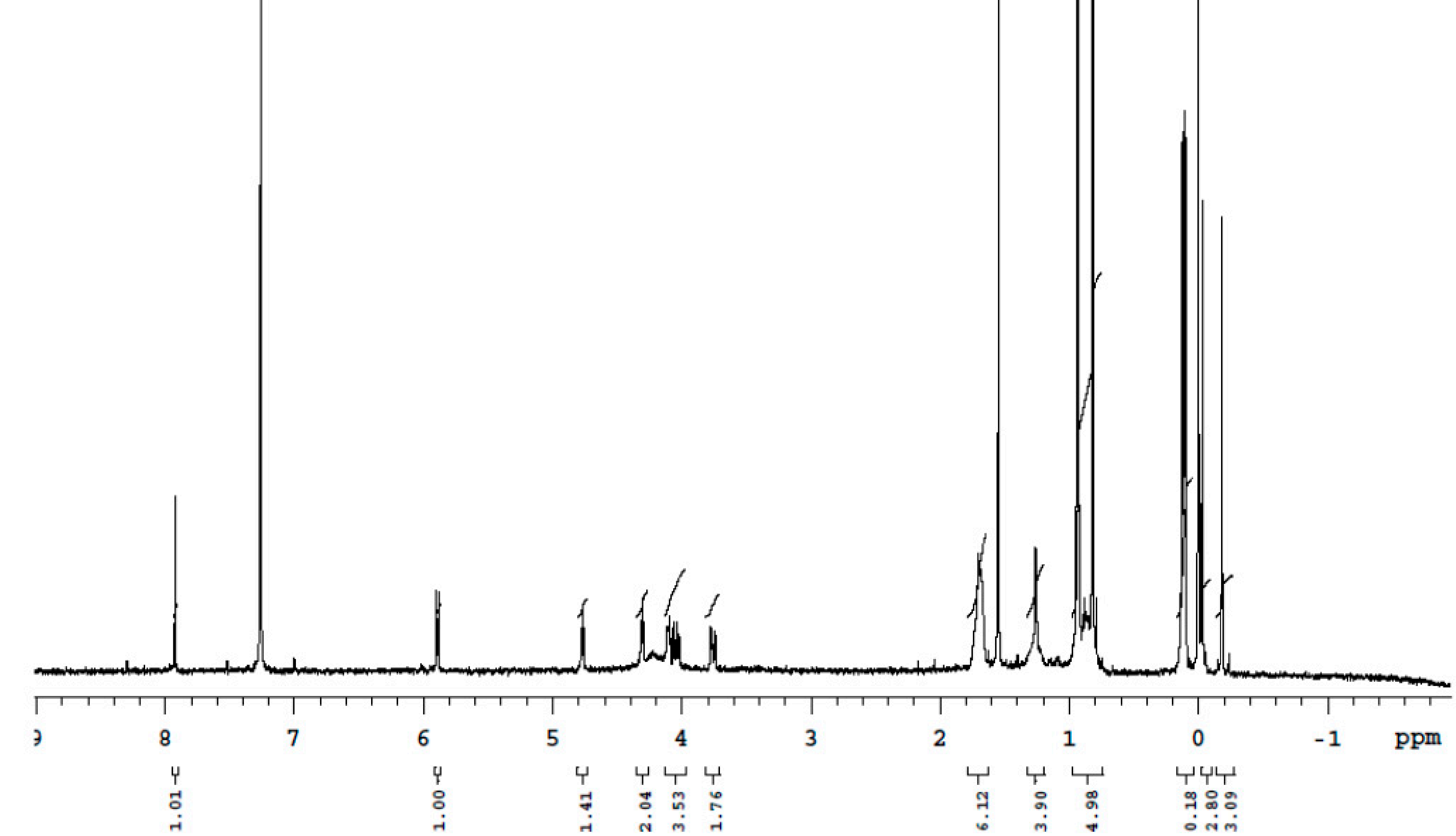
Sample Name :
GVK-DD0397-E10932-PI-1
Solvent: cdcl3
Date: Feb 27 2015
400 VNMRS/NRM-1/NMR-002
9
8
7
6
5
4
3
2
1
0
-1
ppm
1.01
1.00
1.41
2.04
3.53
1.76
6.12
3.90
4.98
0.18
2.80
3.09

Sample Name :
GVK-DD0-397-PIP-T

Solvent: cdcl3
Date: Mar 29 2015
400 VNMRS/NRM-1/NMR-002

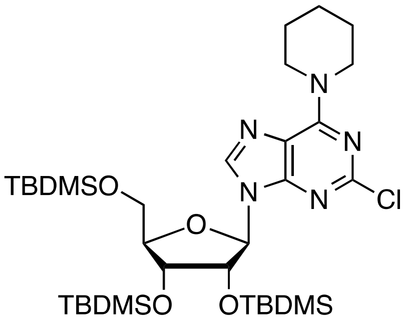

9b

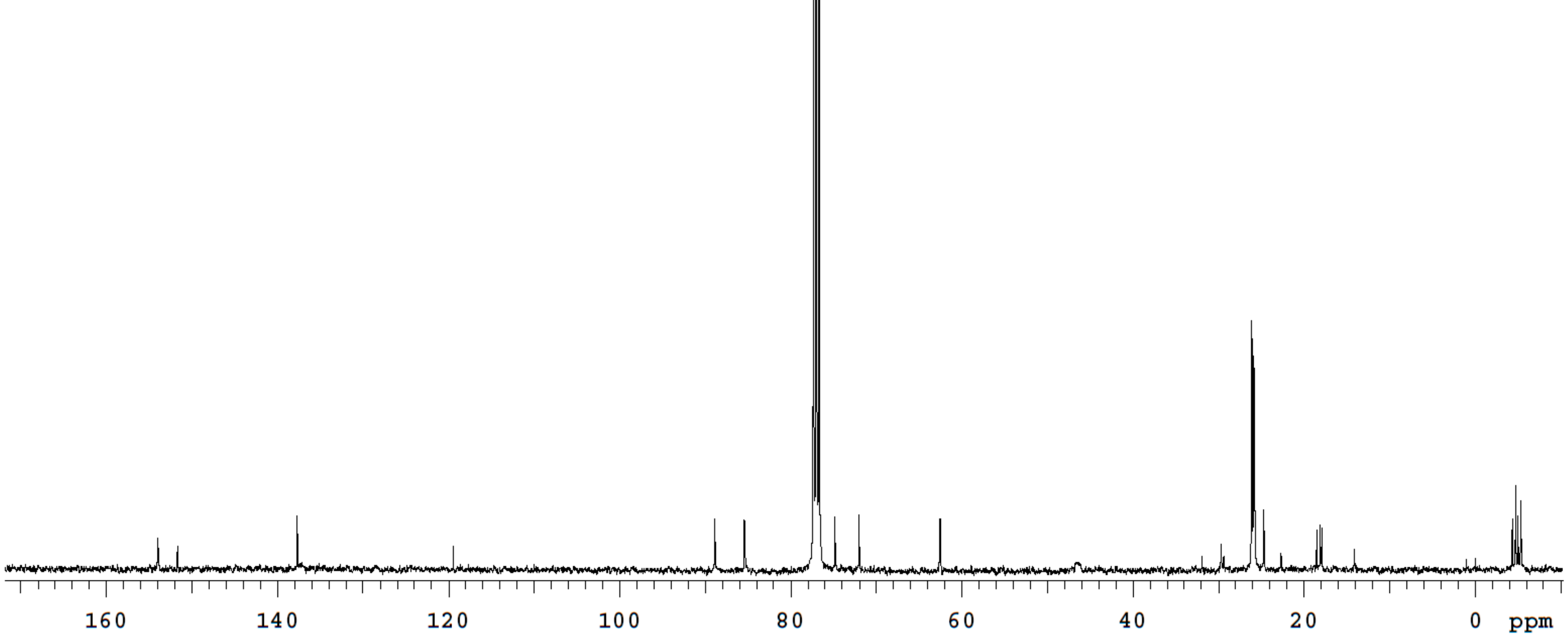

Plotname: 021503D2568_CARBON_01_plot01

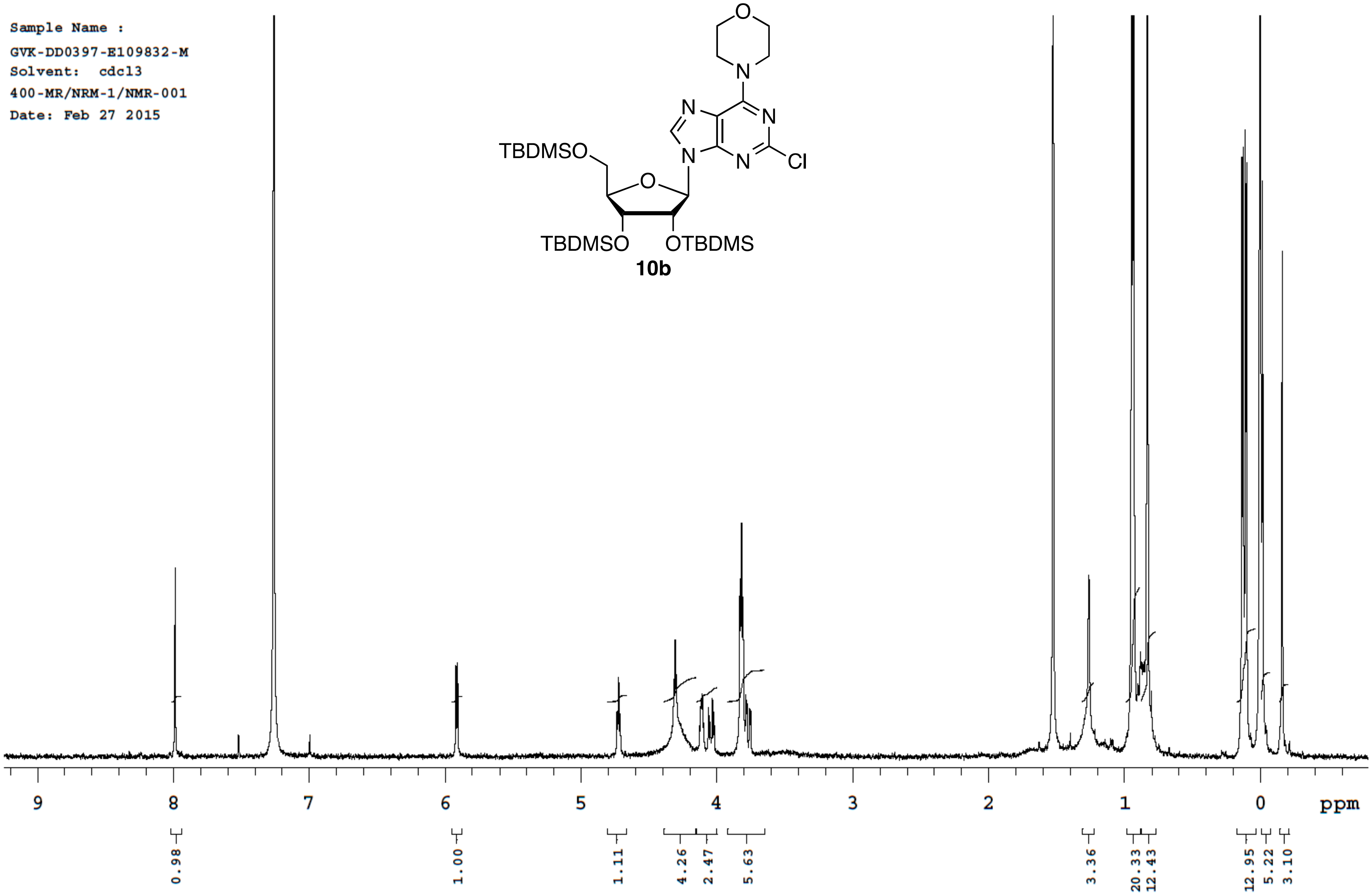

Plotname: 021502D3581_PROTON_01_plot06

Sample Name :
GVK-DD0397-MOR-T

Solvent: cdcl3
Date: May 4 2015
400 VNMRS/NRM-1/NMR-002

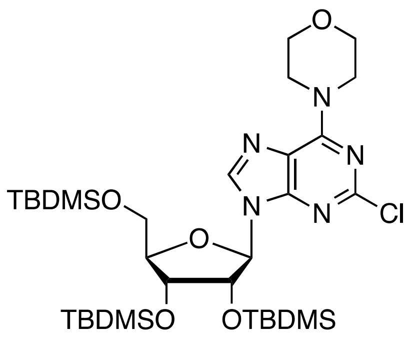

10b

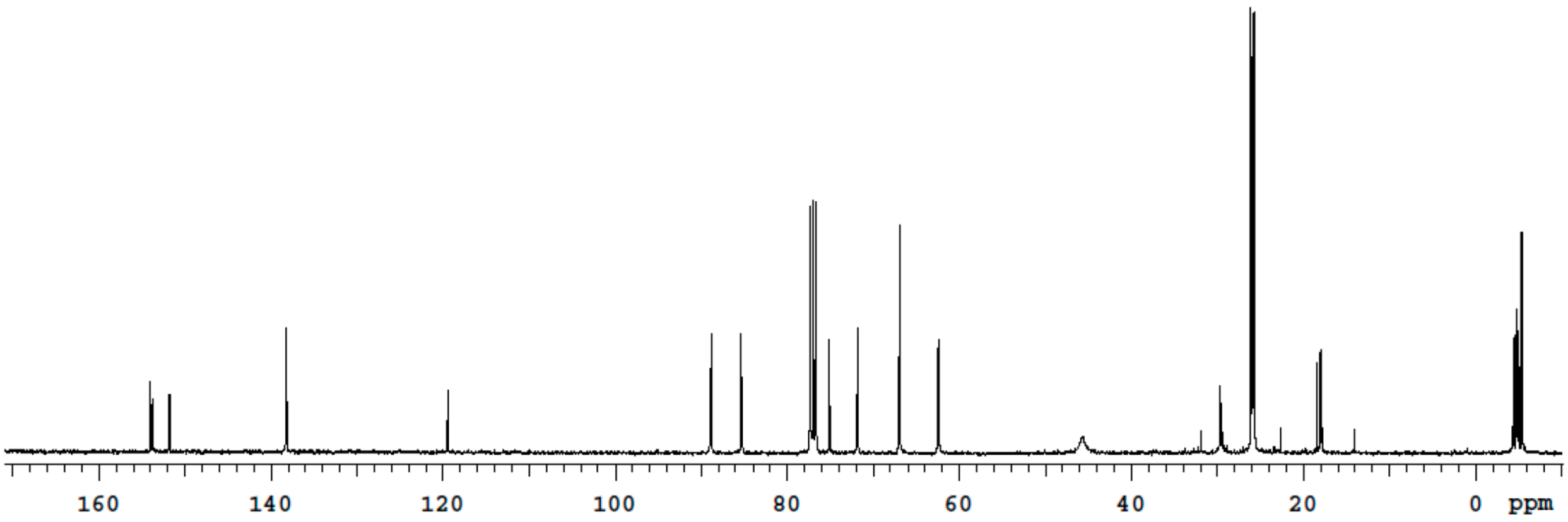

Sample Name:
GVK-DD0-397-TME-T
Solvent: cdcl3
400 VNMRS/NRM-1/NMR-002
Temp. 30.0 C / 303.1 K
Relax. delay 1.000 sec
Acq. time 1.000 sec
Pulse 30.0 degrees
Line broadening 0.5 Hz
Date: Mar 29 2015

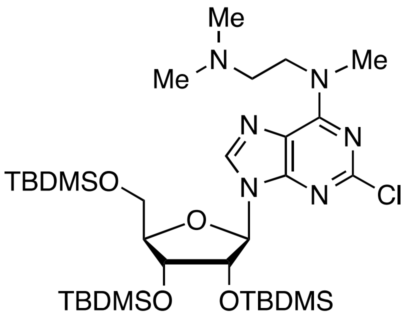

11b

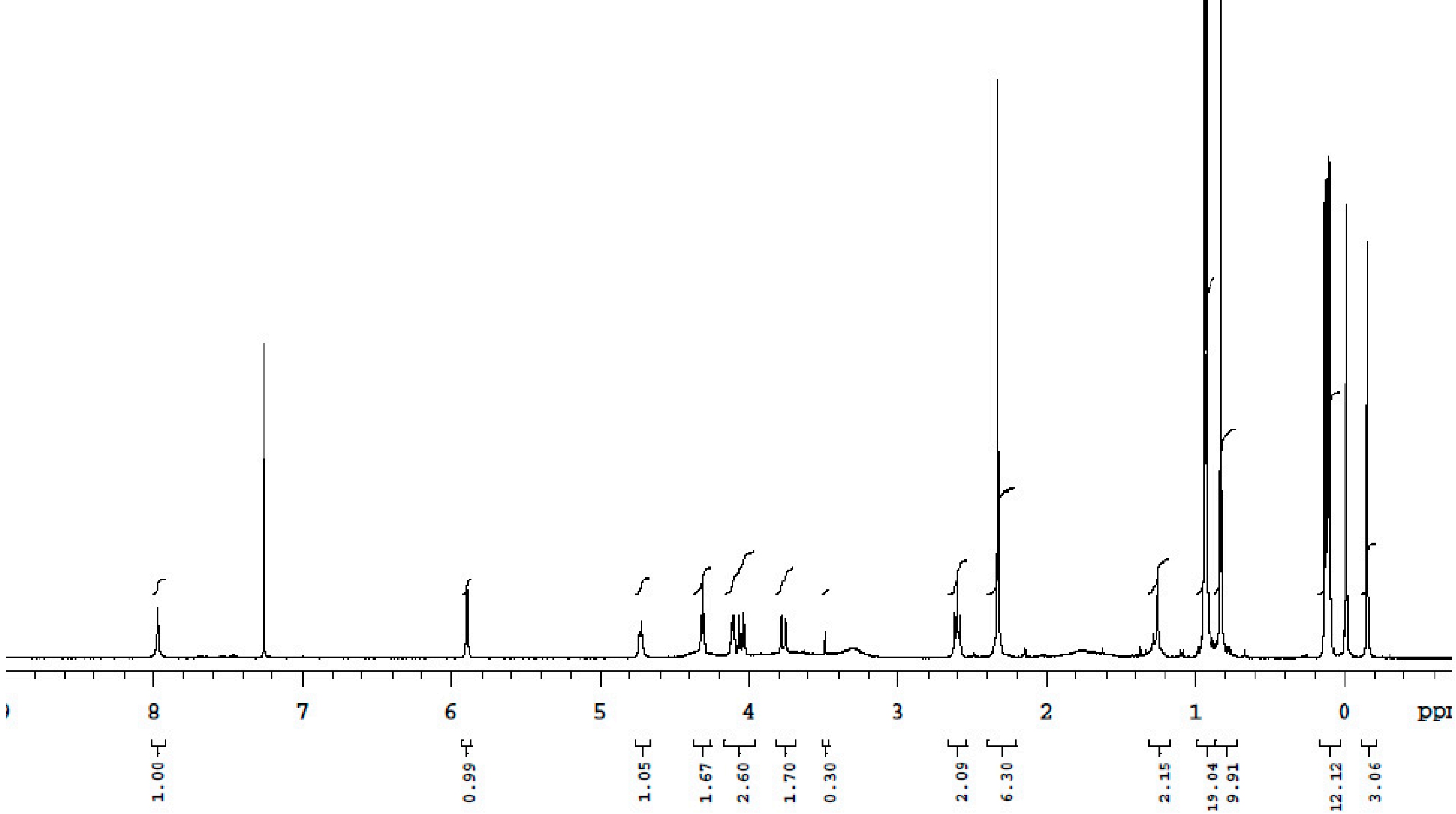

Sample Name :
GVK-DD0-397-TME-T

Solvent: cdcl3
Date: Mar 29 2015
400 VNMRS/NRM-1/NMR-002

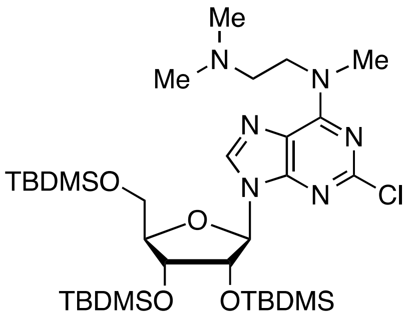

11b

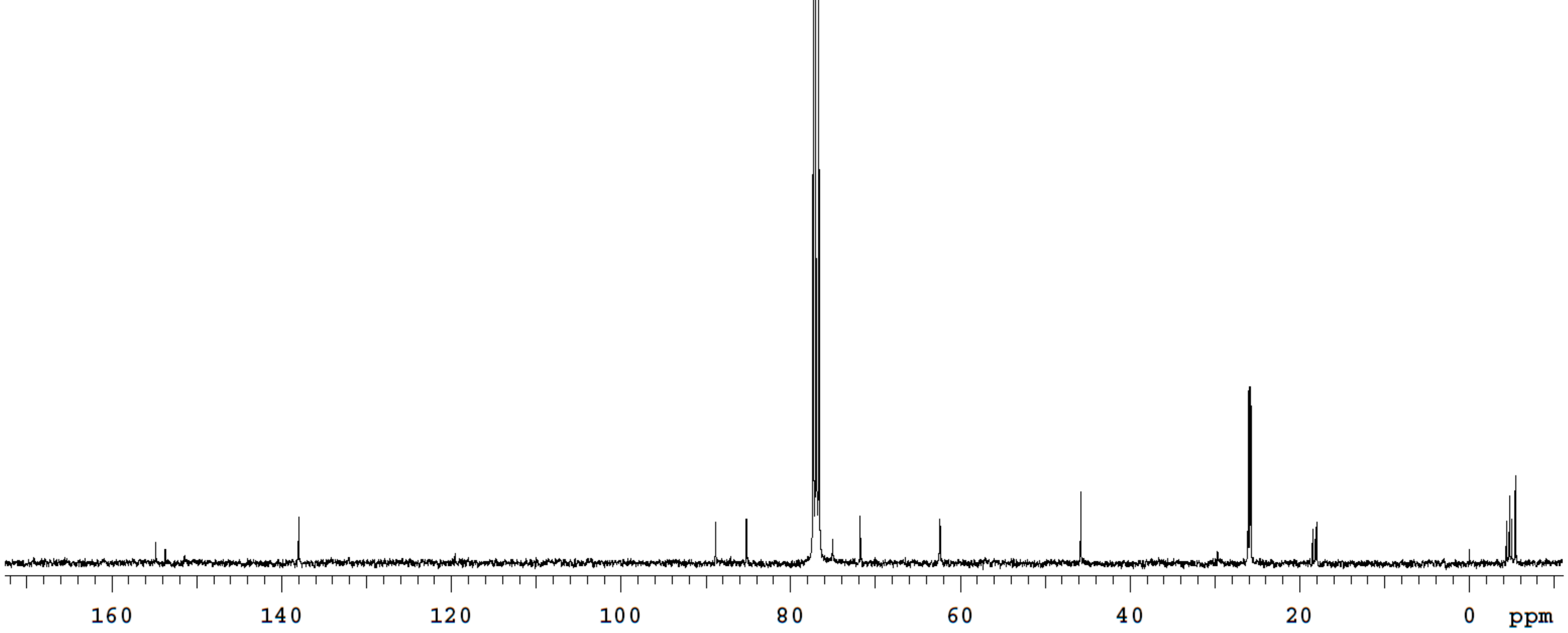

Plotname: 021503D2561_CARBON_01_plot01

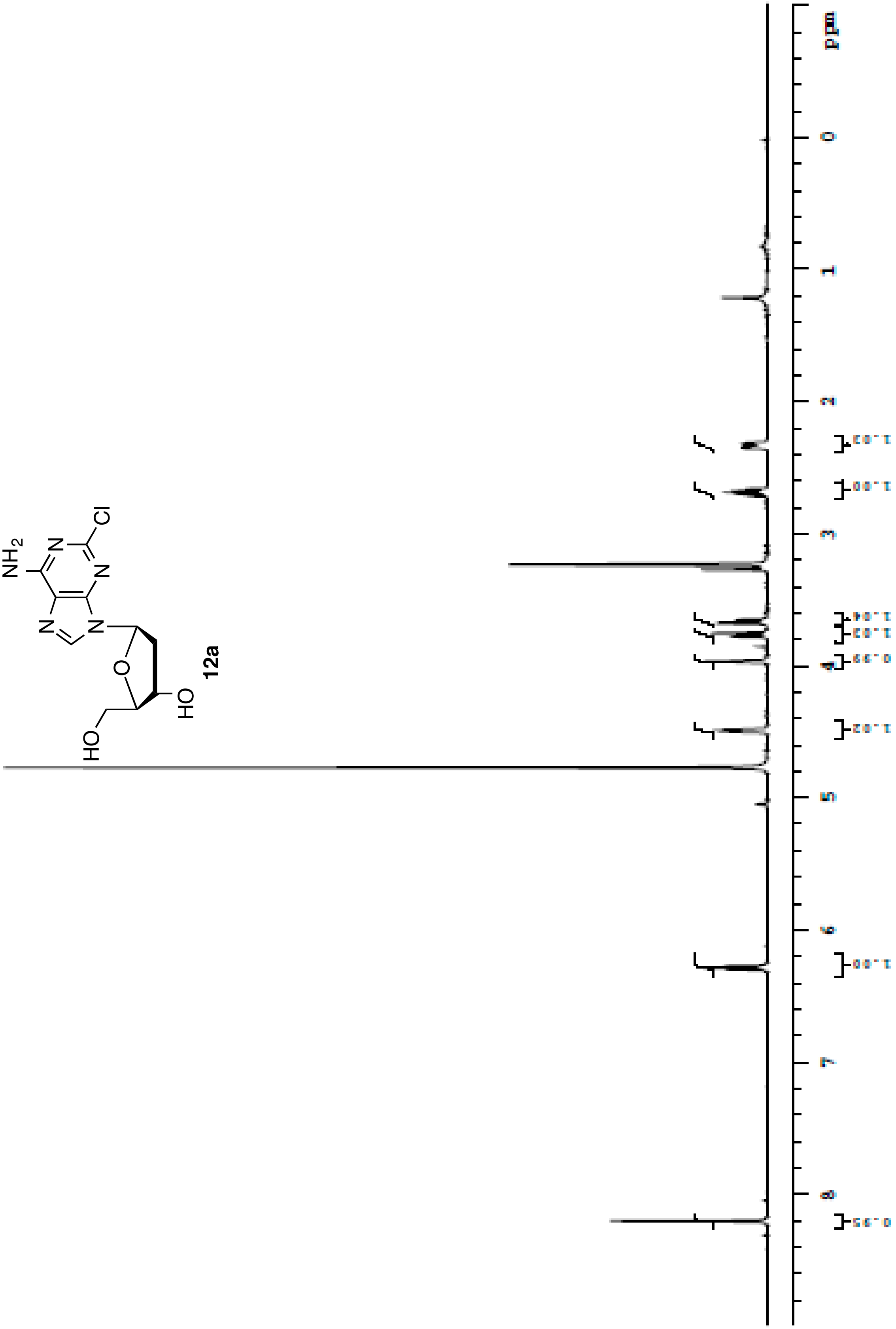

12a

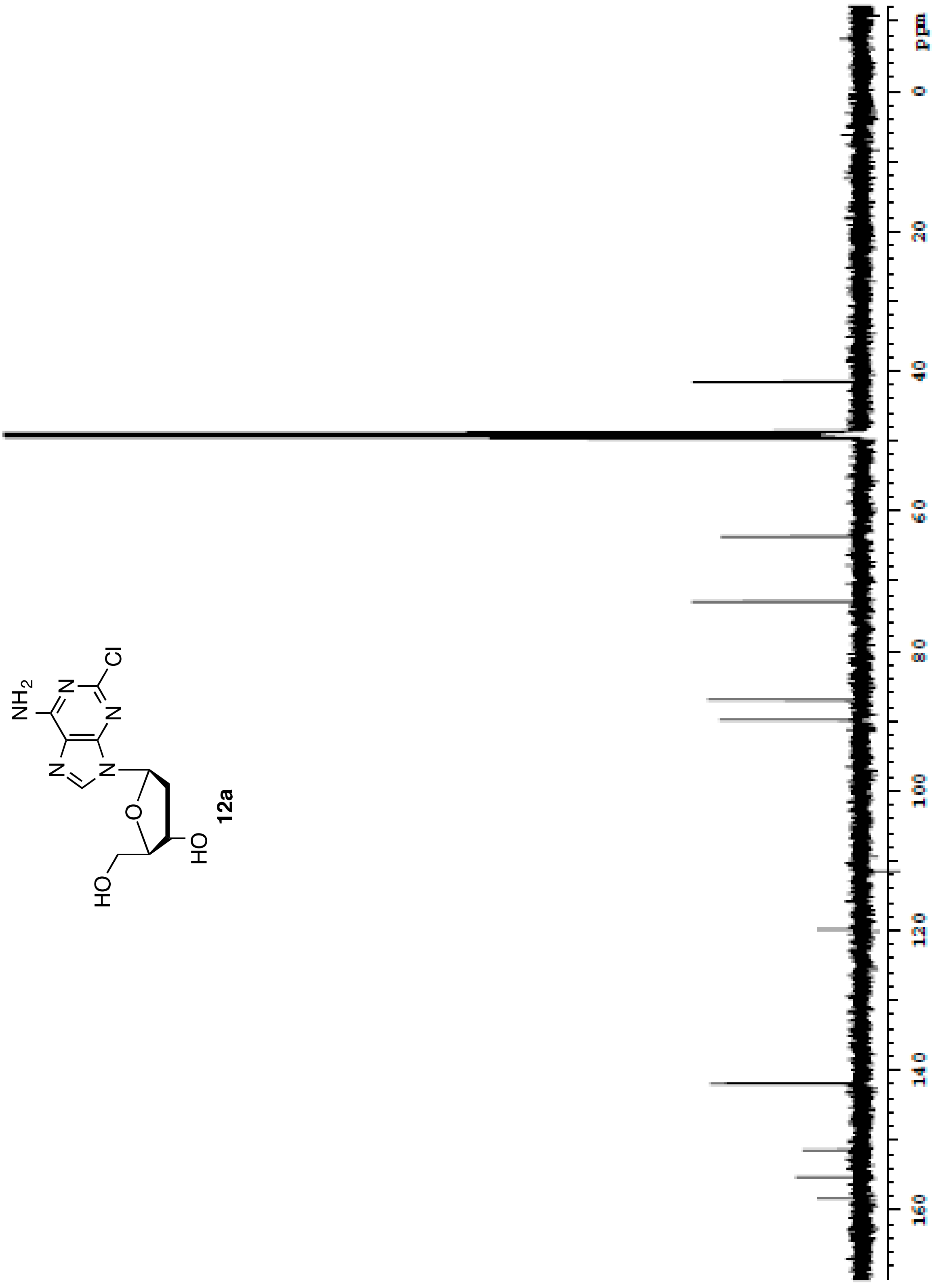
NH2
N
N
Cl
N
N
N
O
HO
HO
12a
ppm
0
20
40
60
80
100
120
140
160

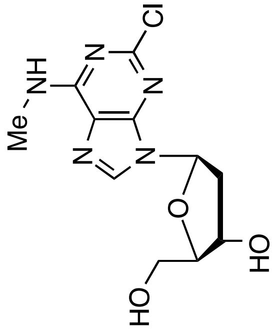
Me
NH
N
N
Cl
N
N
O
HO
HO

13a

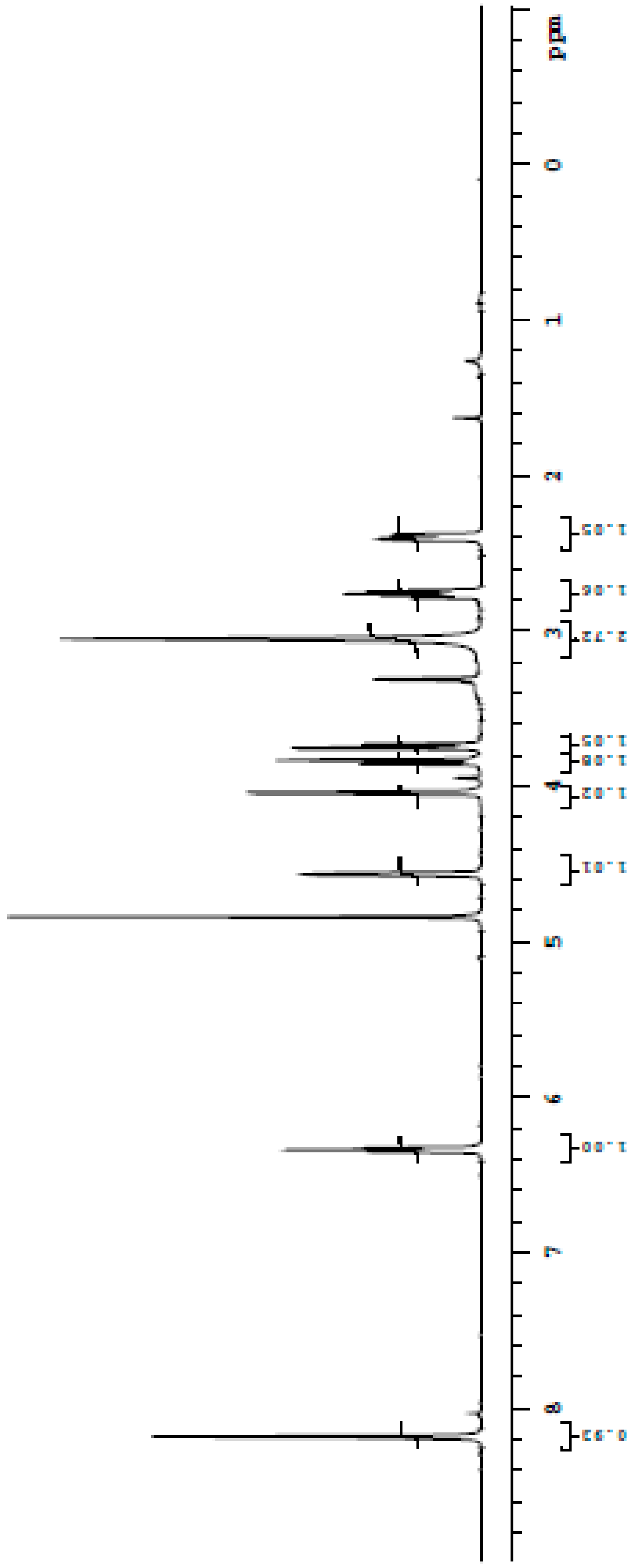
ppm
0
1
2
3
4
5
6
7
8
1.05
1.06
2.72
1.05
1.08
1.02
1.01
1.00
0.93

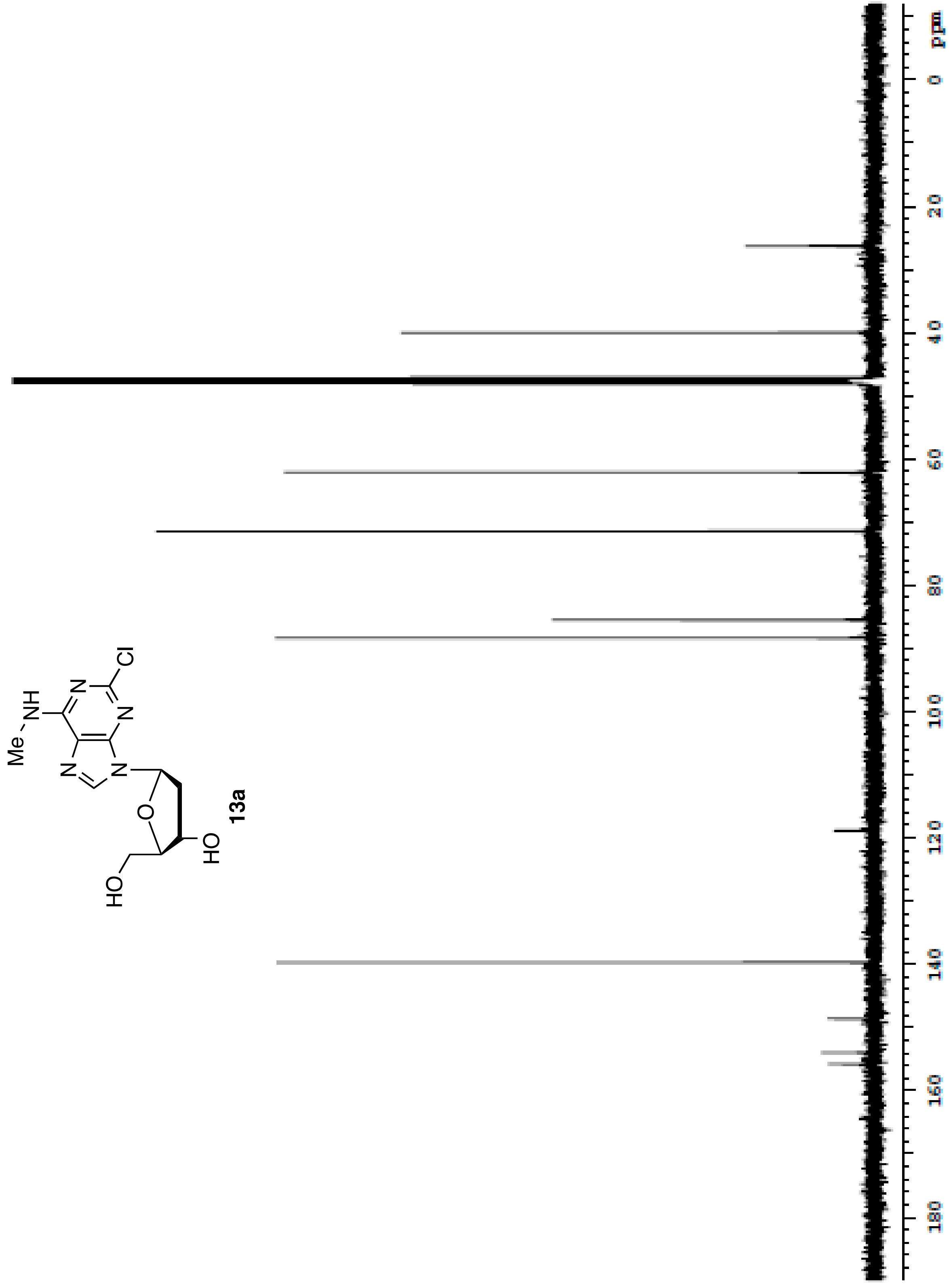

Me
NH
N
N
Cl
O
HO
HO
13a
ppm
0
20
40
60
80
100
120
140
160
180

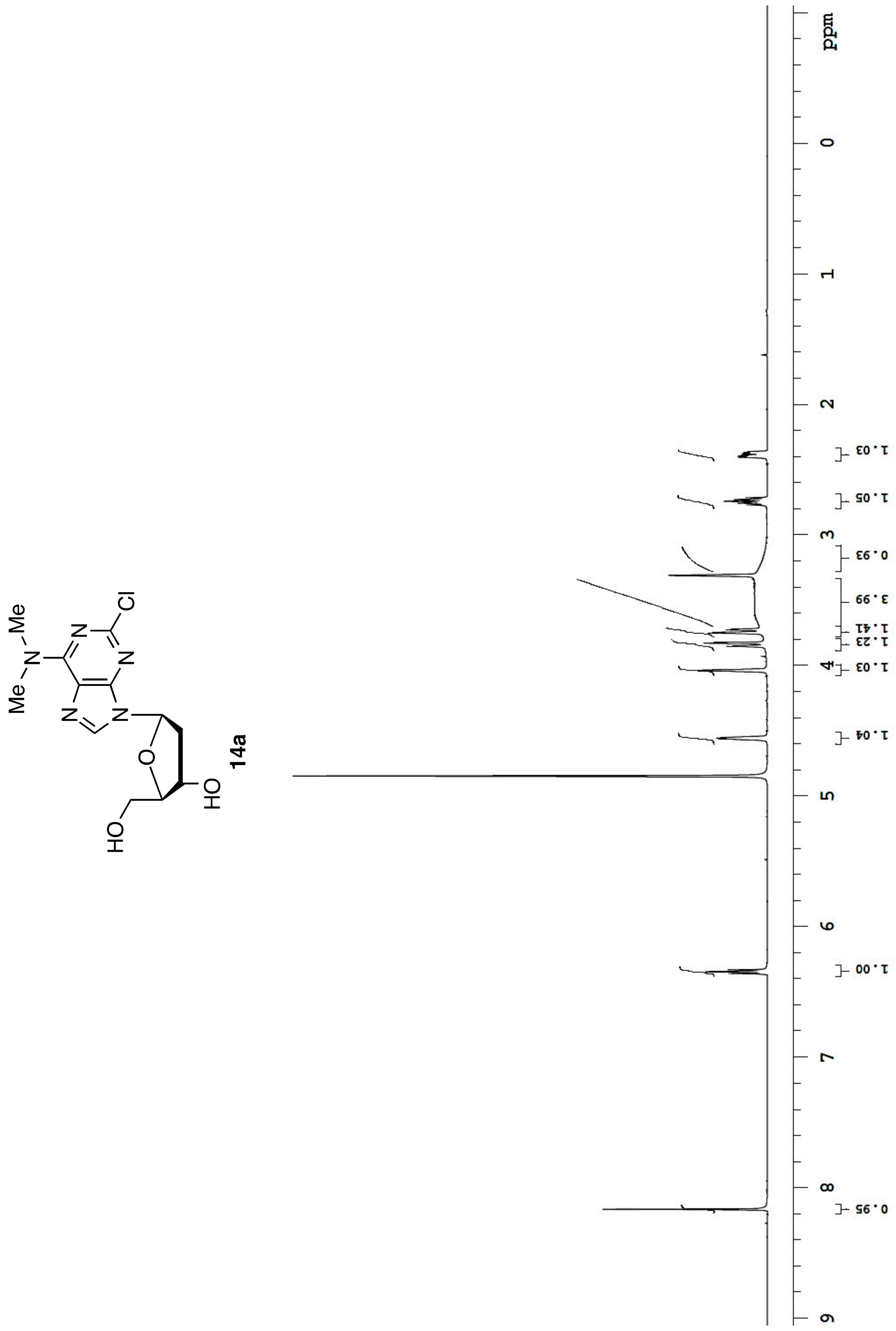

Me
N
Me
N
N
Cl
N
N
O
HO
HO
14a
ppm
0
1
2
3
4
5
6
7
8
9
1.03
1.05
0.93
3.99
1.41
1.23
1.03
1.04
1.00
0.95

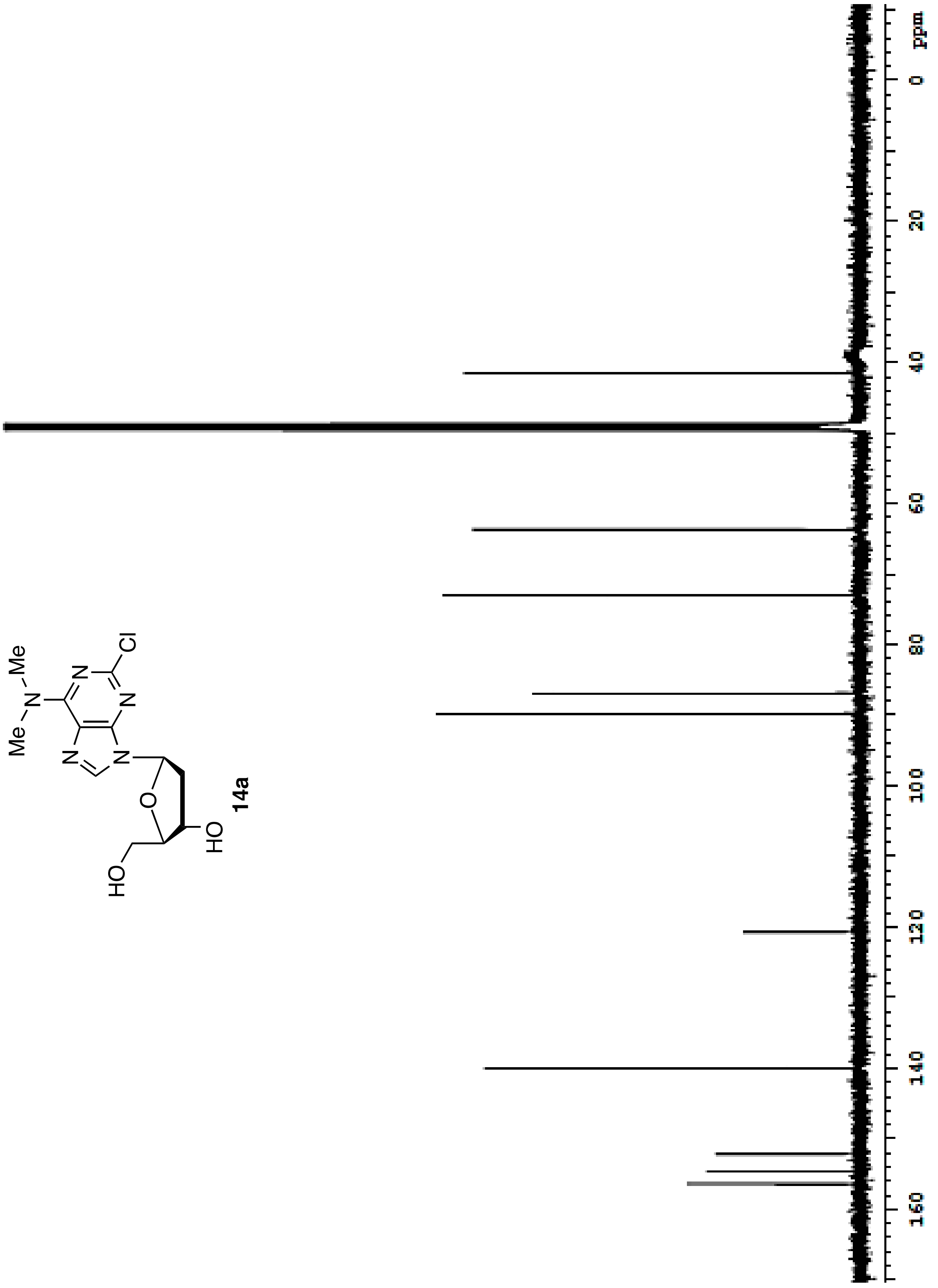

Me
N
Me
N
N
Cl
N
N
O
HO
HO
14a
ppm
0
20
40
60
80
100
120
140
160

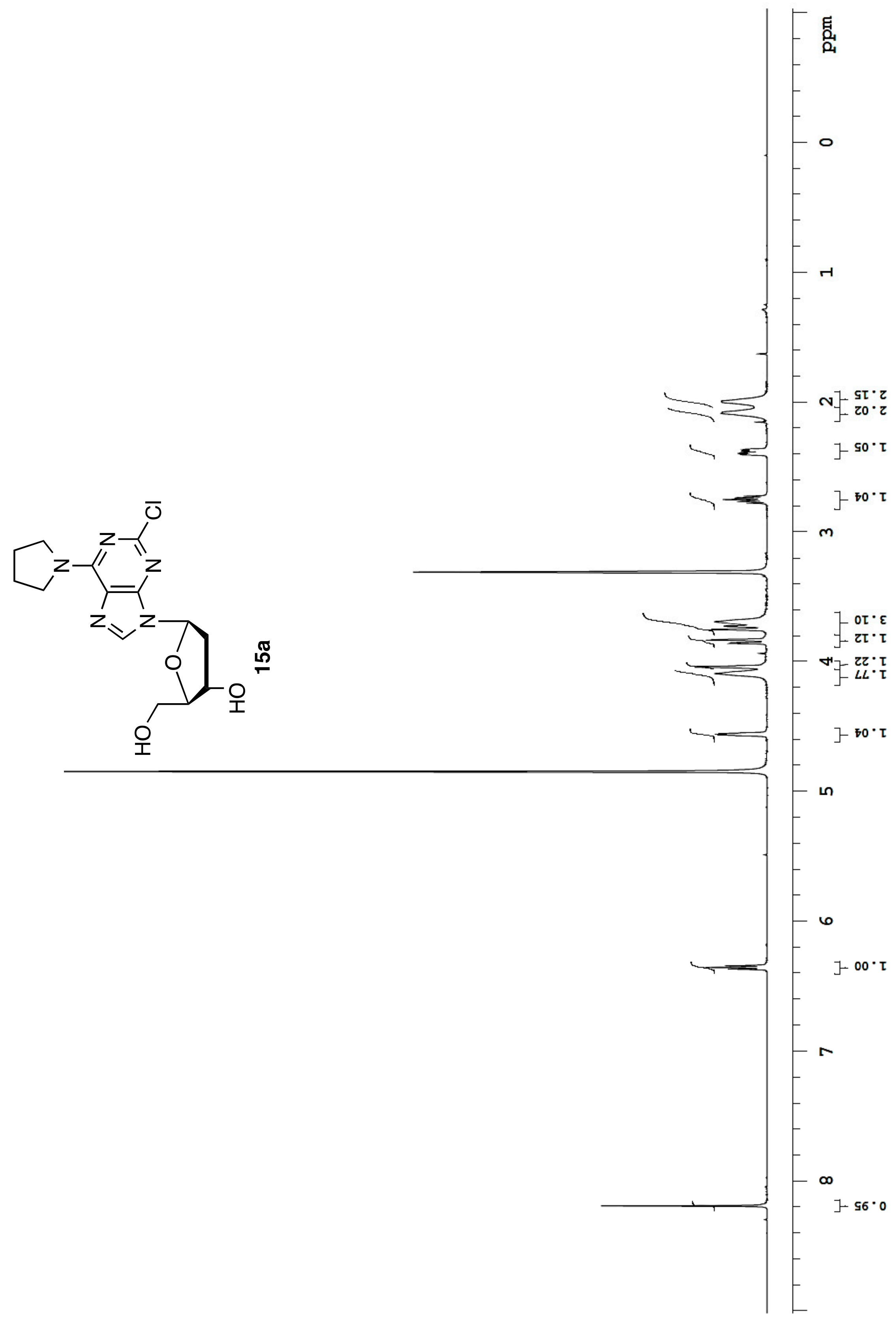

Cl
N
N
N
N
N
O
HO
HO
15a
ppm
0
1
2
3
4
5
6
7
8
2.15
2.02
1.05
1.04
3.10
1.12
1.22
1.77
1.04
1.00
0.95

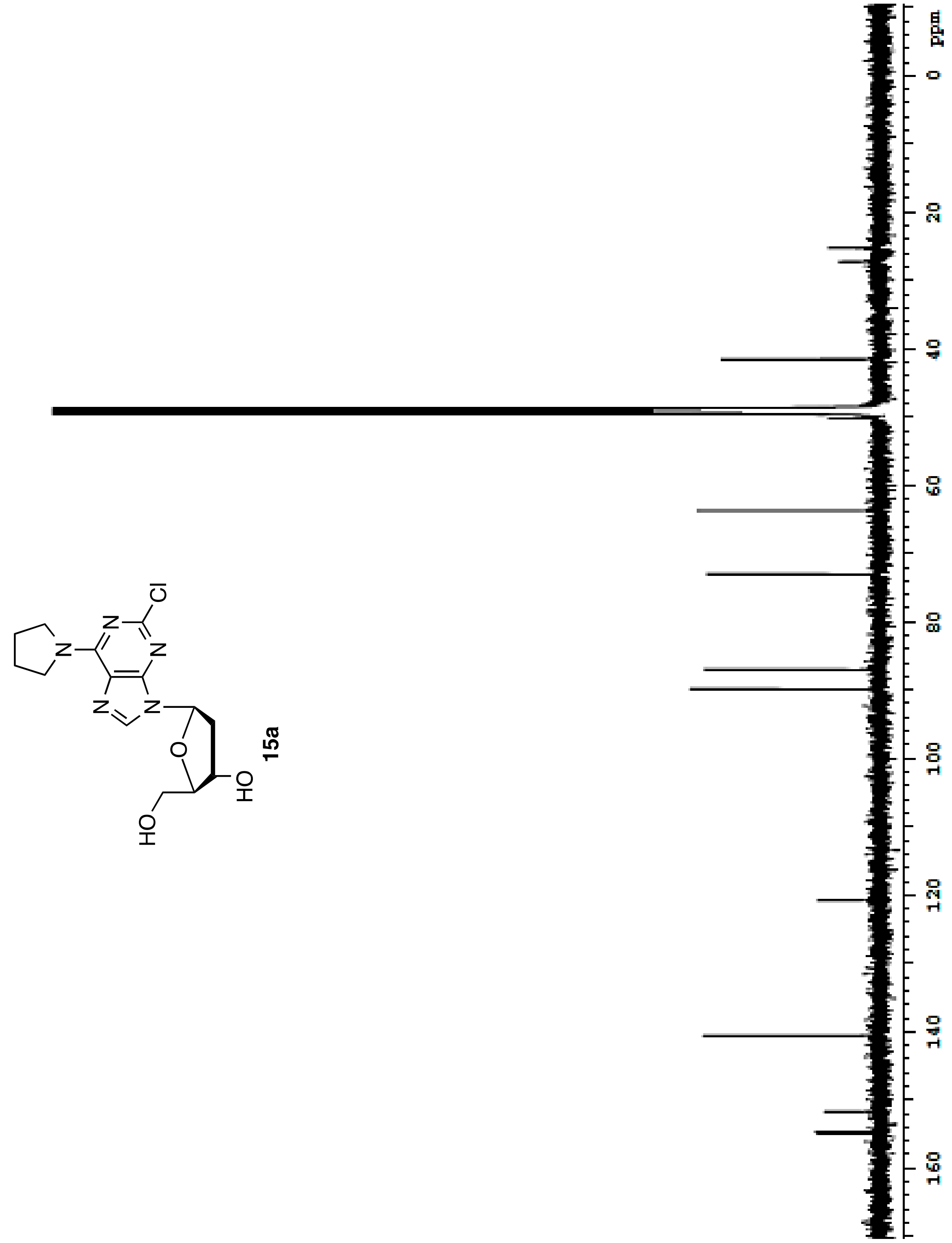

15a

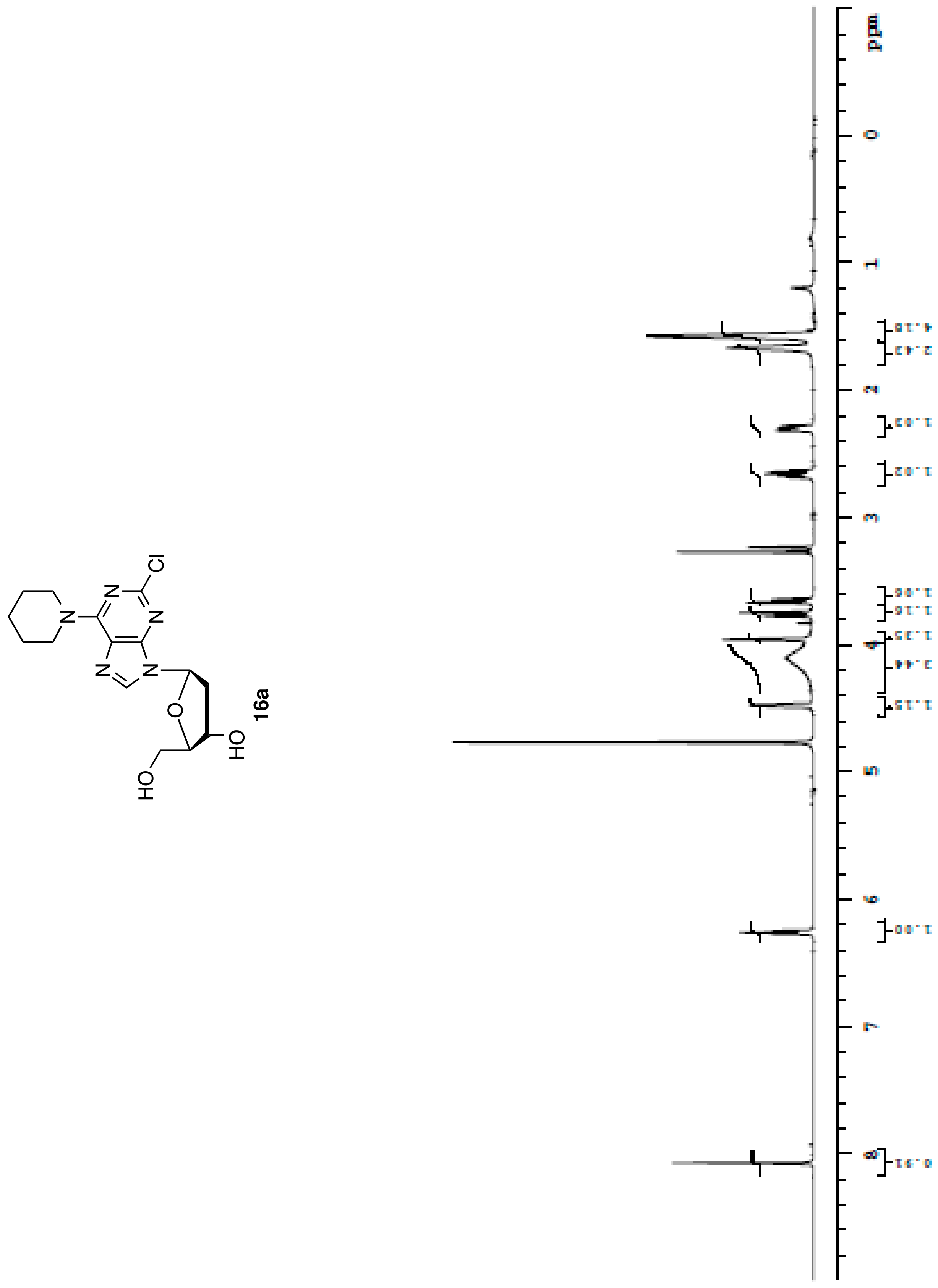
16a
ppm

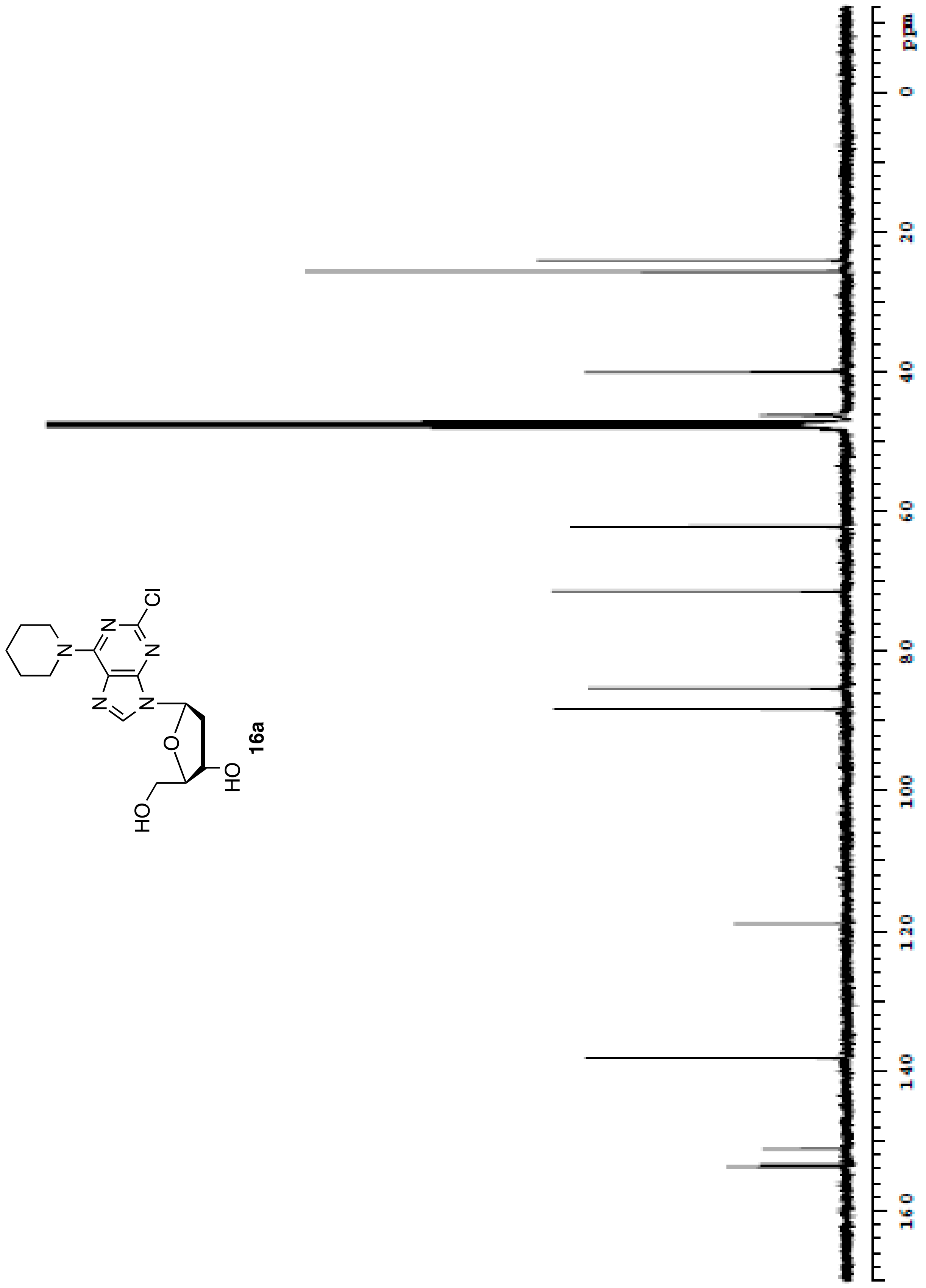

ppm
0
20
40
60
80
100
120
140
160
Cl
N
N
N
N
N
O
HO
HO
16a

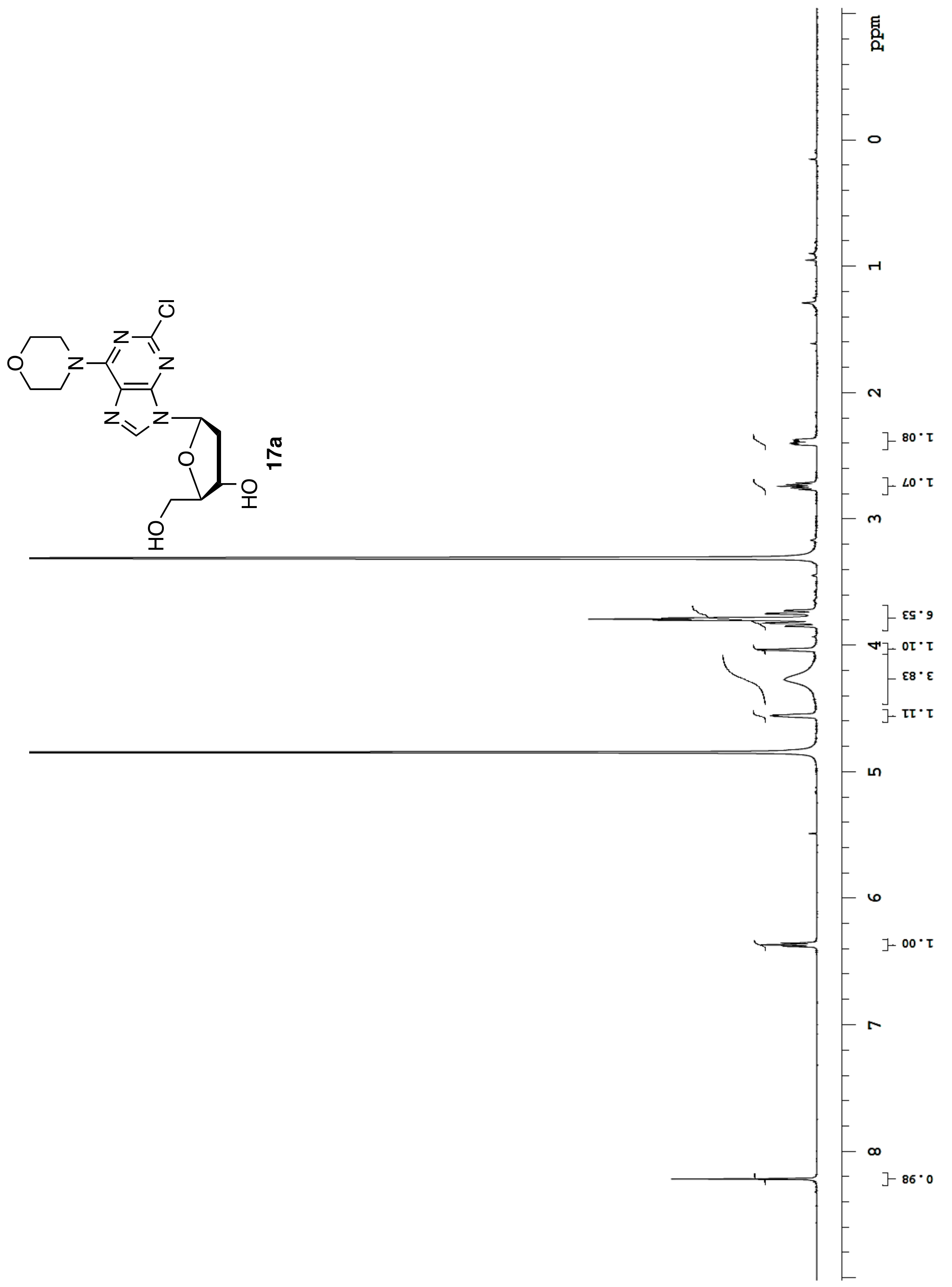

17a

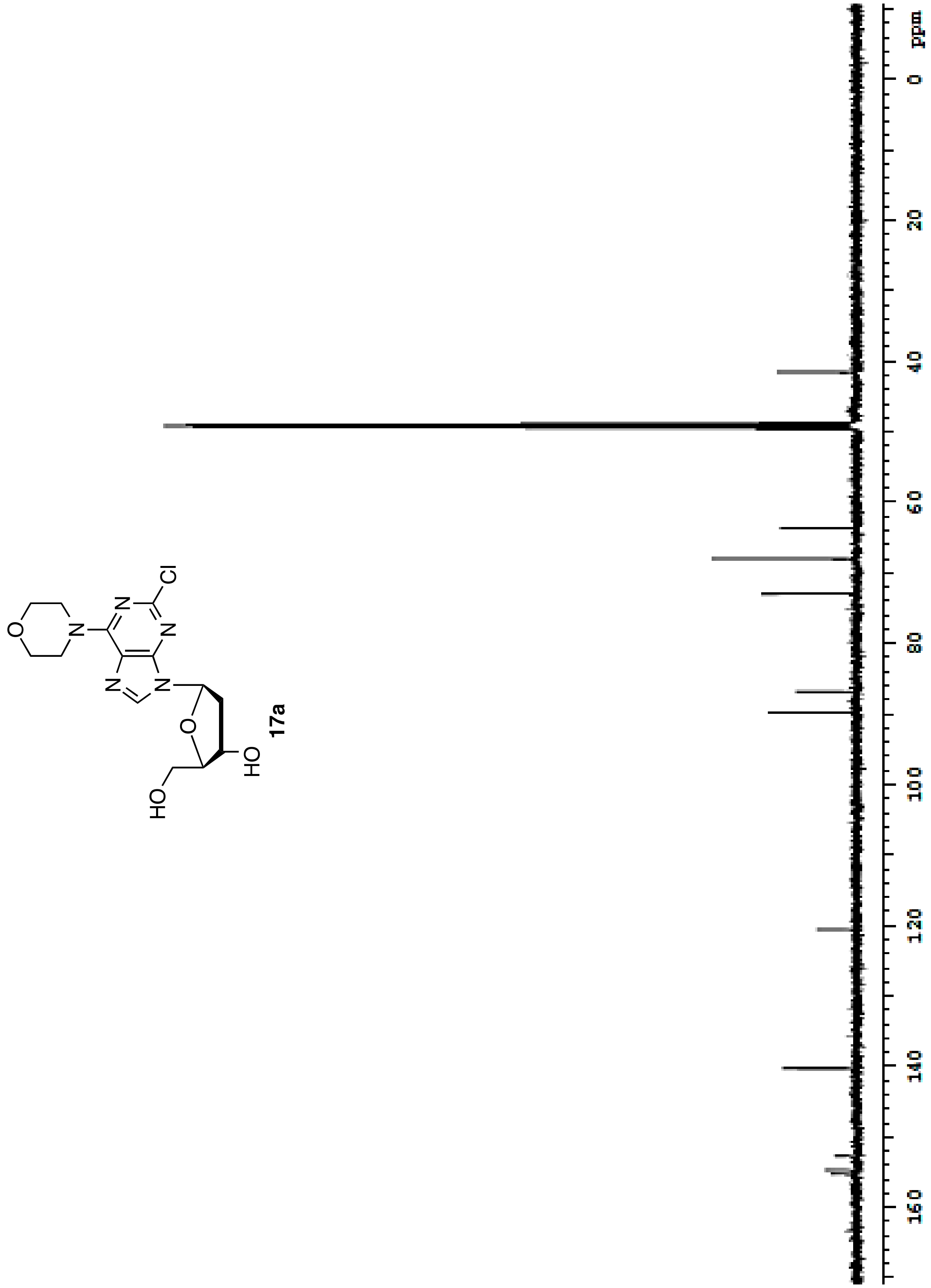

17a

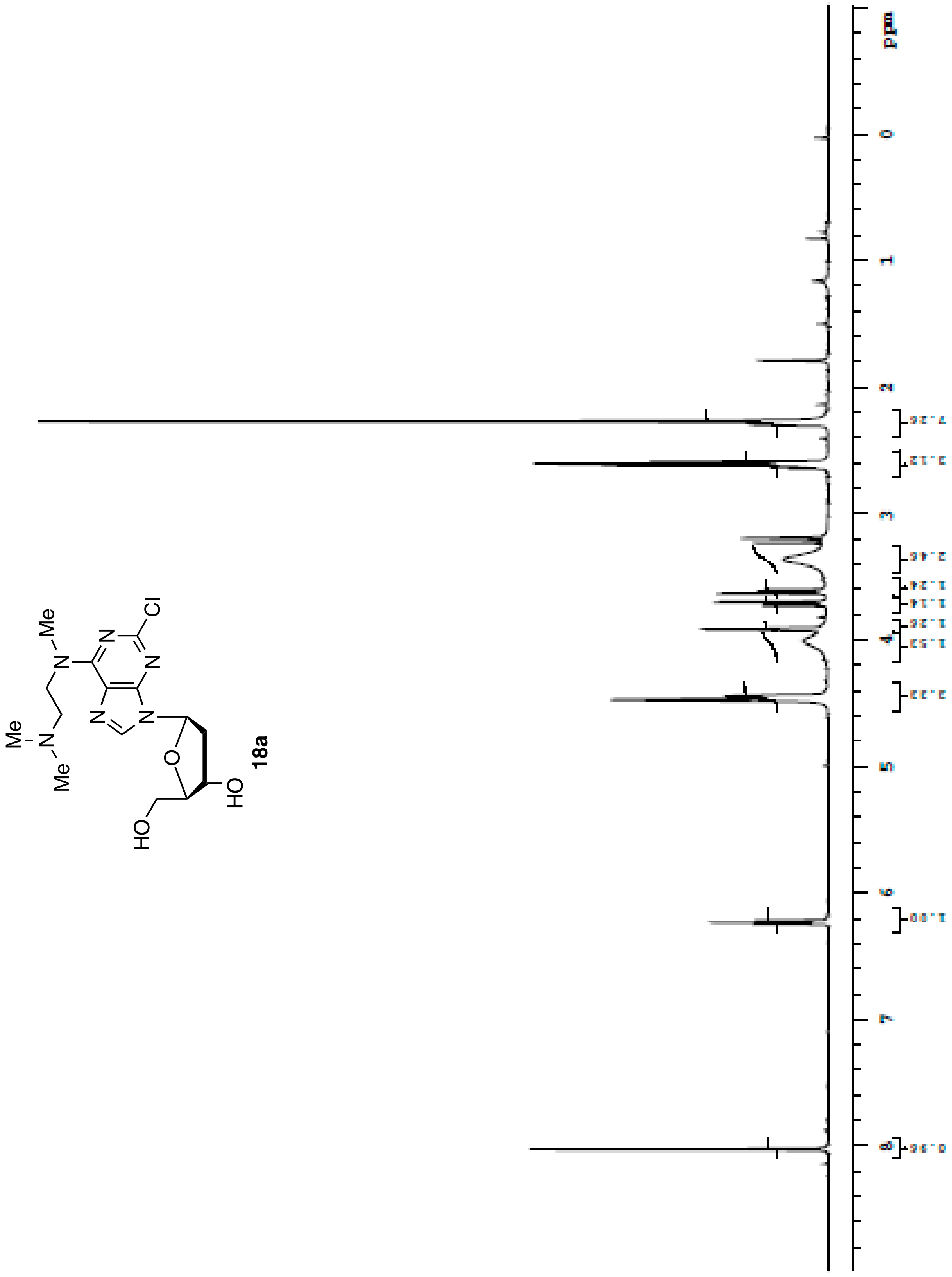
18a

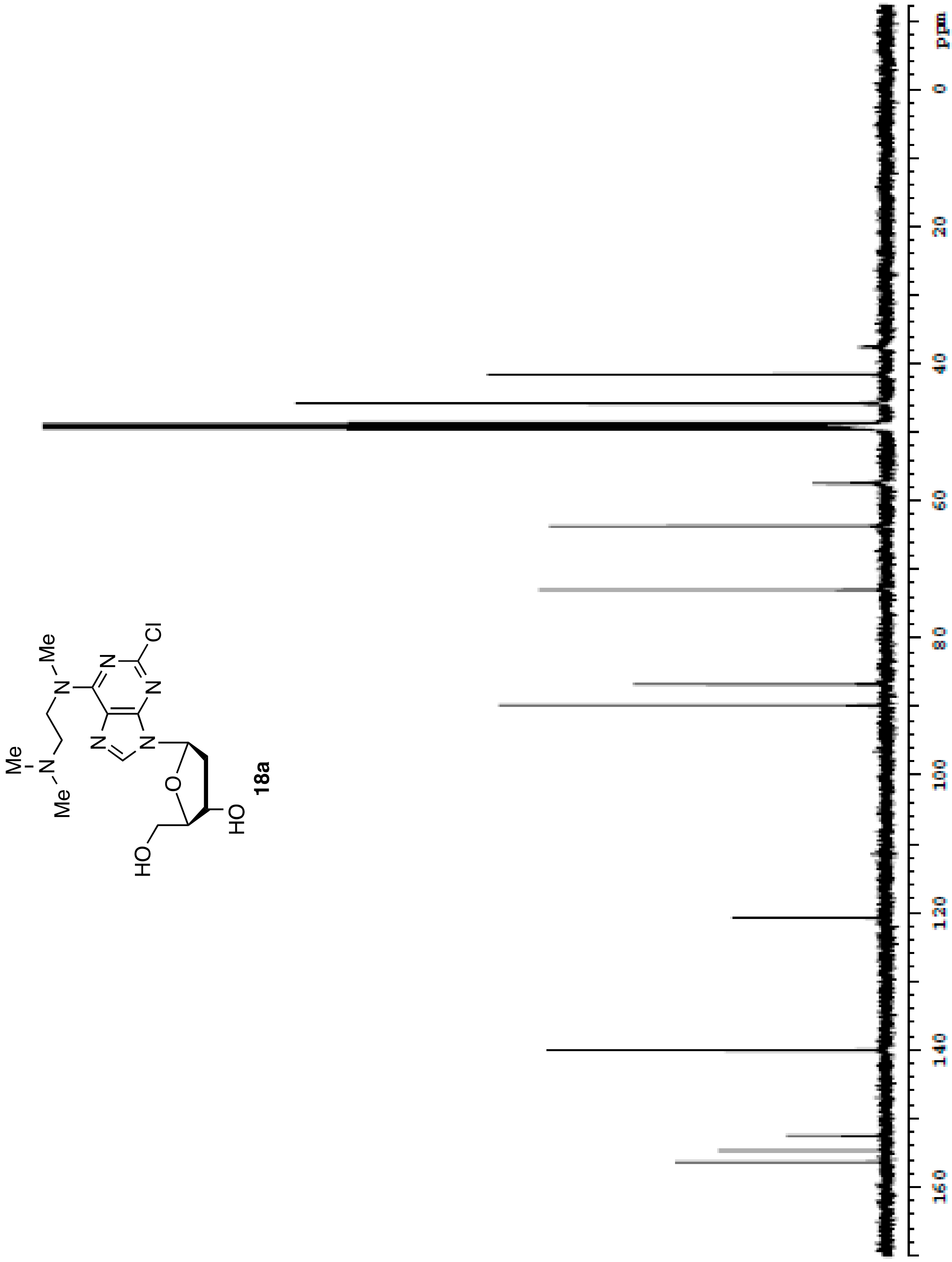

Me
N
Me
N
Me
N
N
Cl
N
N
N
O
HO
HO
18a
ppm
0
20
40
60
80
100
120
140
160

Sample Name :
GVK-DD0397-AMM-F
Solvent: cd3od
400-MR/NRM-1/NMR-001
Date: Apr 2 2015

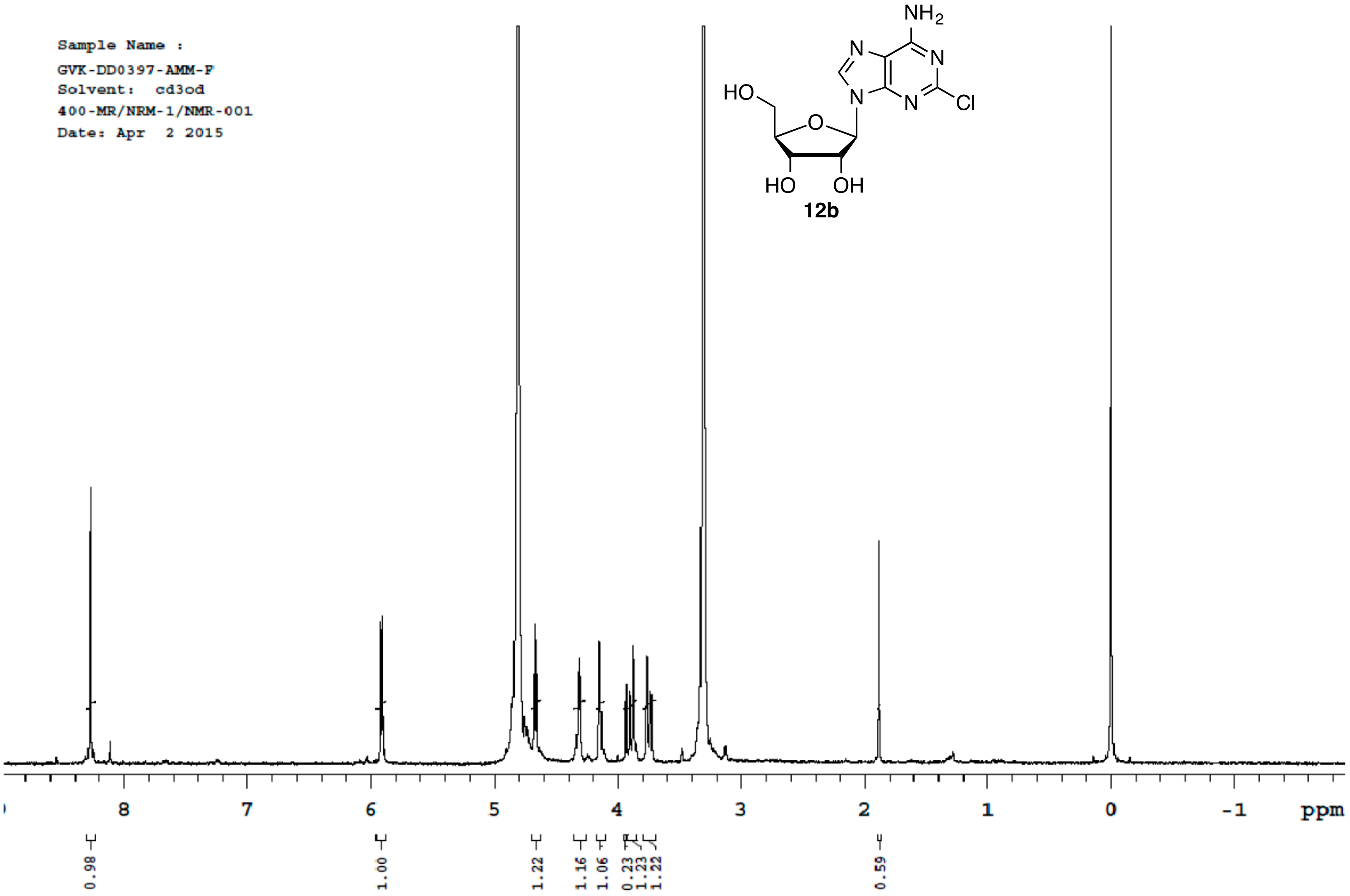

GVK-DD0397-Amm-F sol problem
Reference Code: 021504B2827
Solvent: dmso
Archive directory:
/home/gvkbio/data/2015/Apr
Agilent 400-MRDD2
Data collected on: Apr 11 2015
Experiment: CARBON

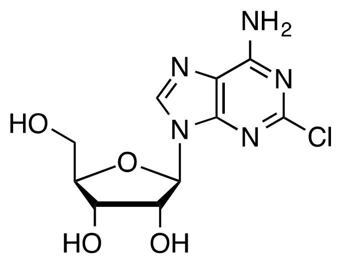

12b

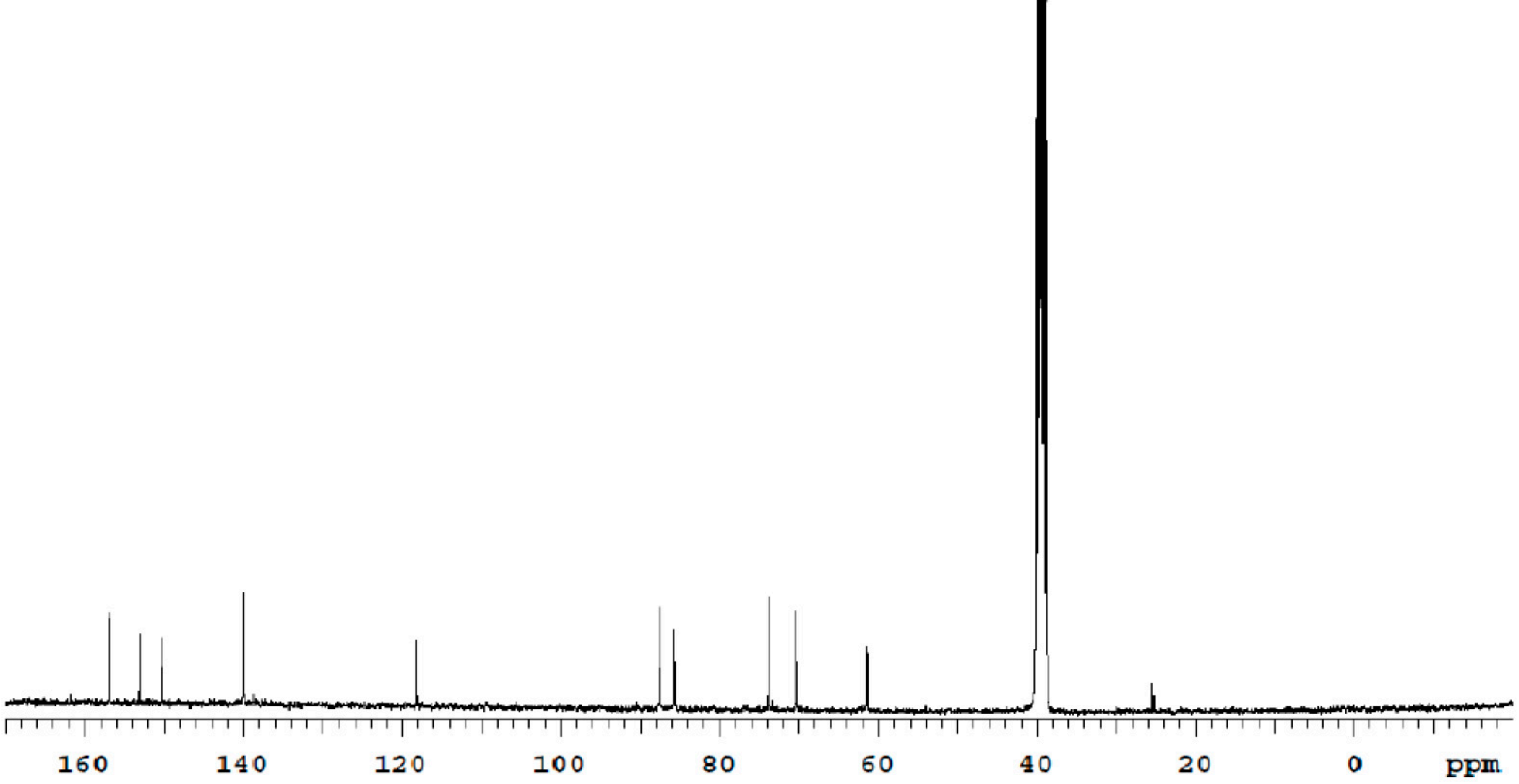

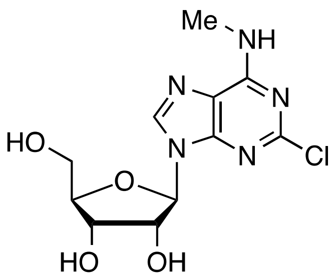

13b

Sample Name :
VK-DD0397-METHYL-F
Solvent: cd3od
400-MR/NRM-1/NMR-001
Date: Apr 2 2015

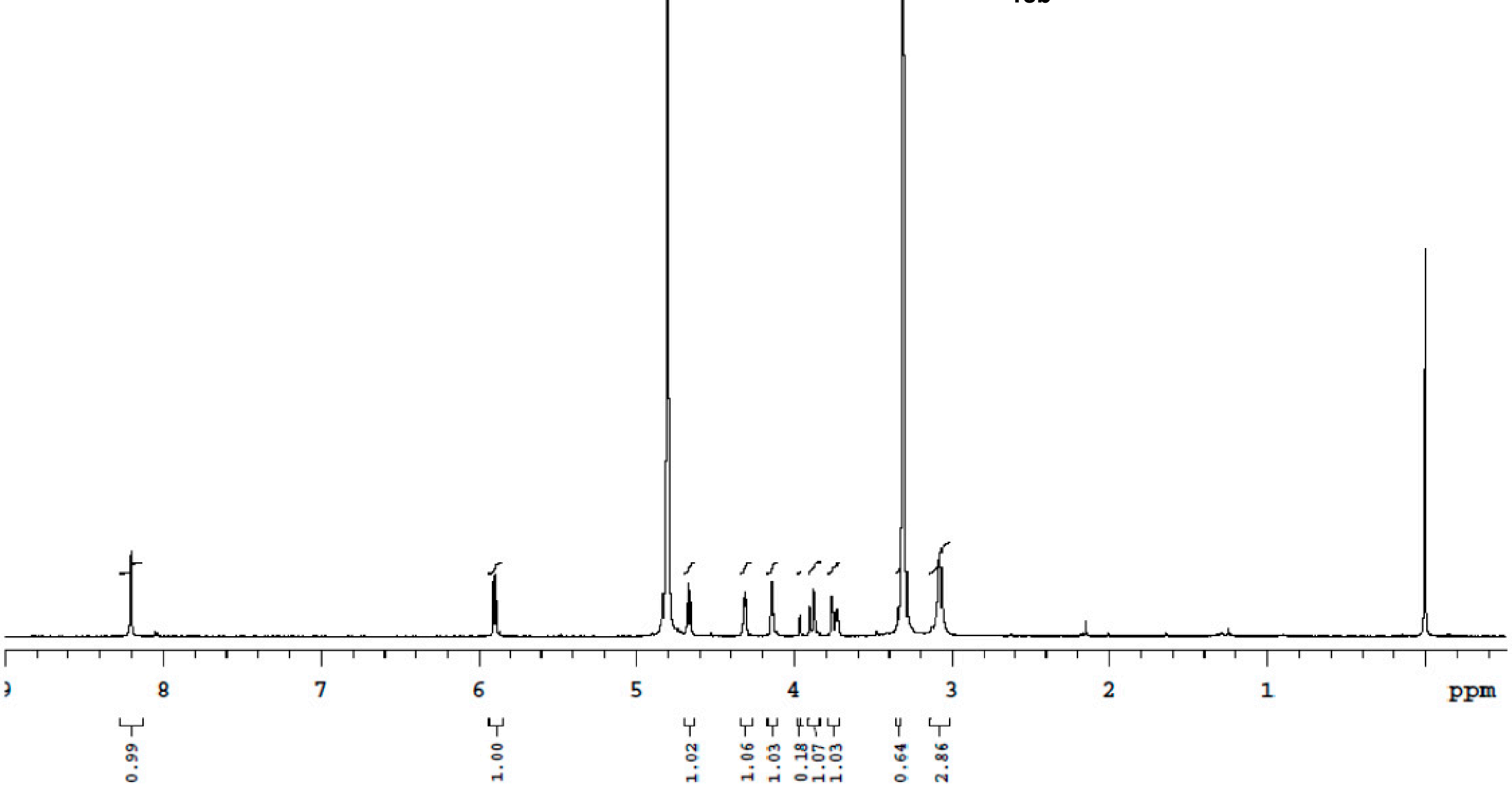

Reference Code: 021504B2796
Solvent: dmso
Archive directory:
/home/gvkbio/data/2015/Apr
Agilent 400-MRDD2
Data collected on: Apr 13 2015
Experiment: CARBON

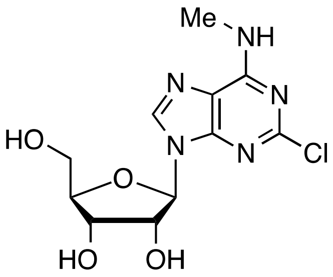

13b

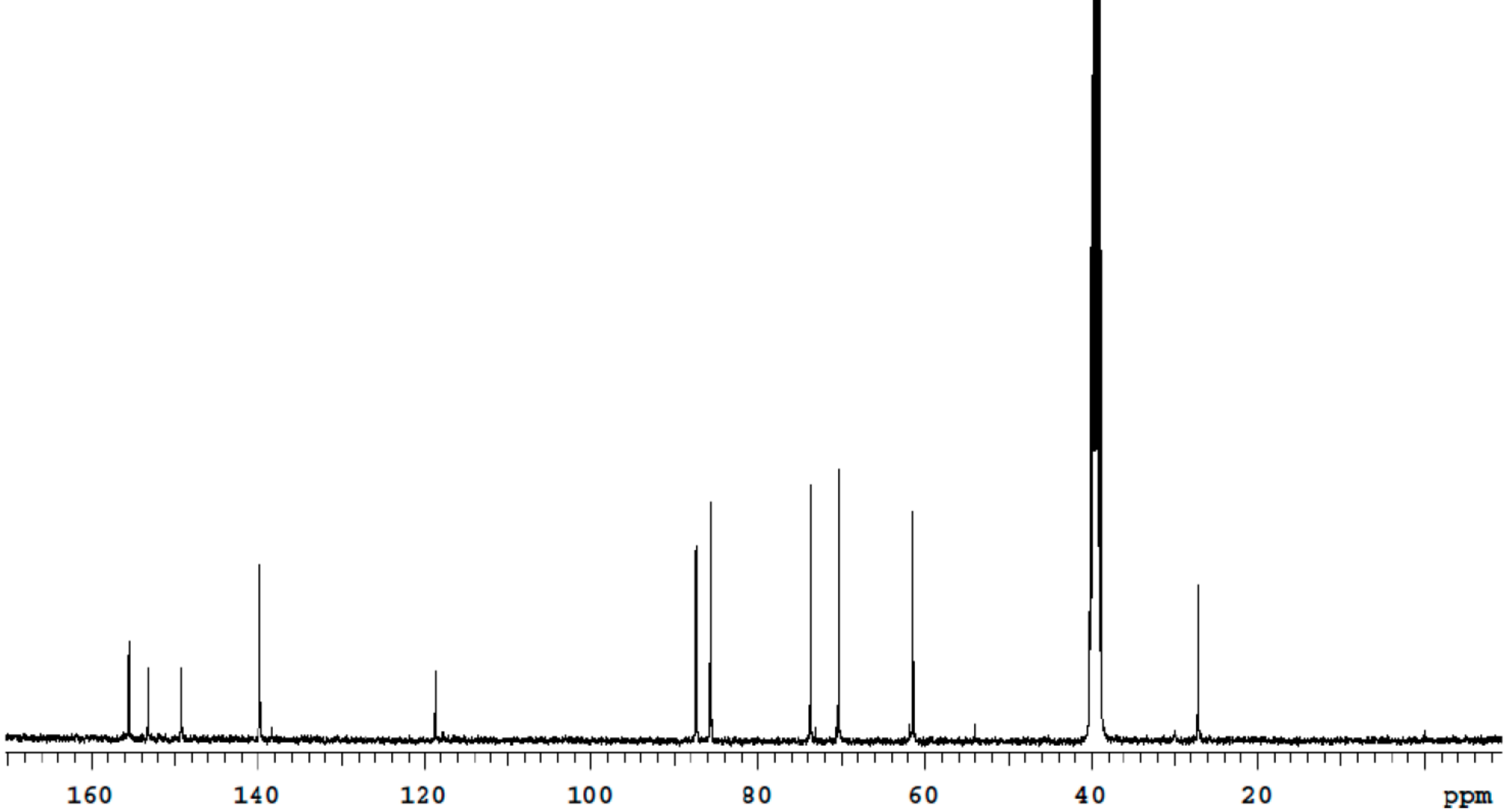

b

Sample Name :
GVK-DD0397-DIMETHYL-F
Solvent: cd3od
400-MR/NRM-1/NMR-001
Date: Apr 2 2015

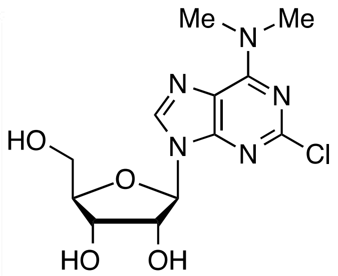

14b

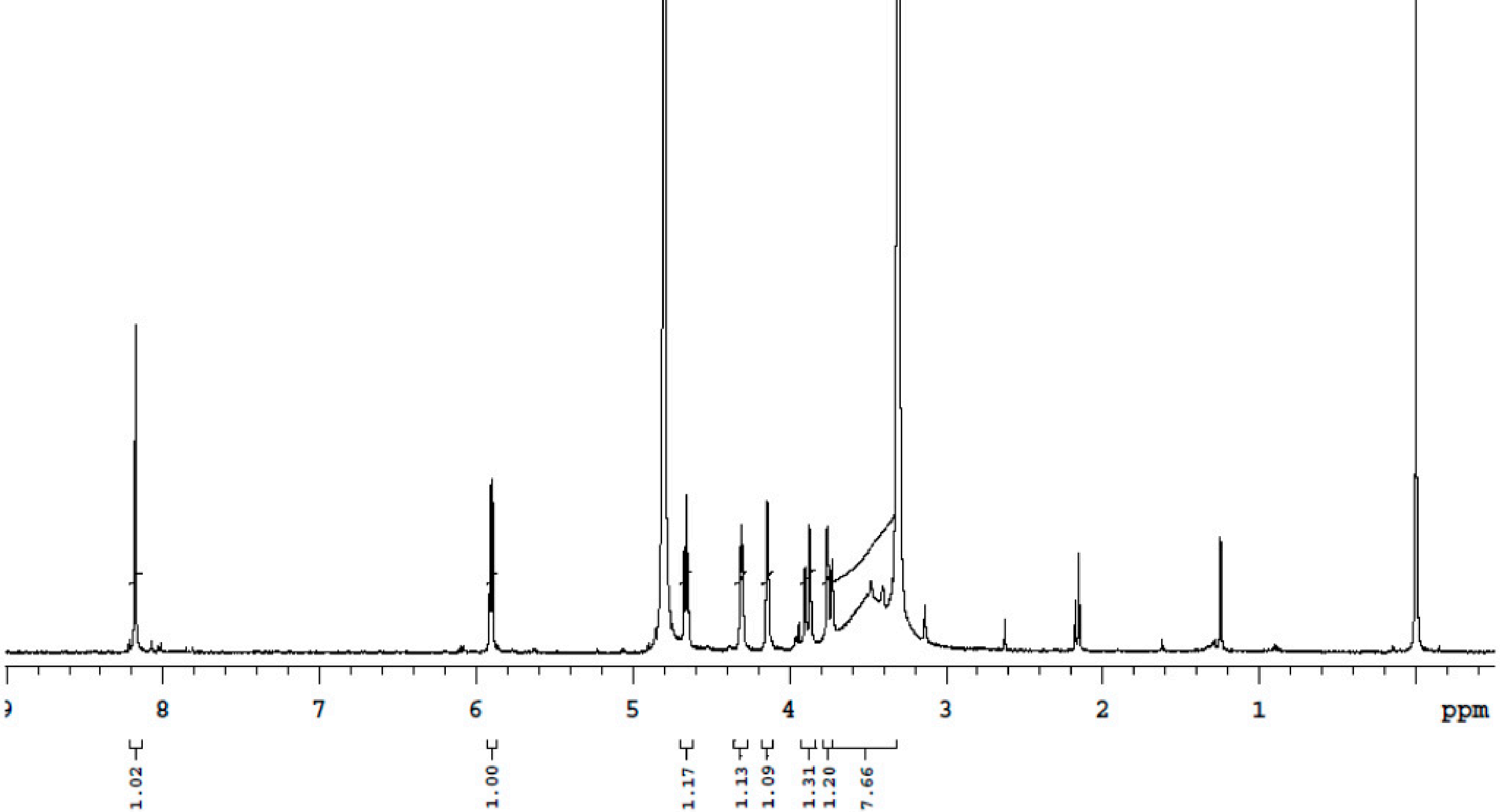

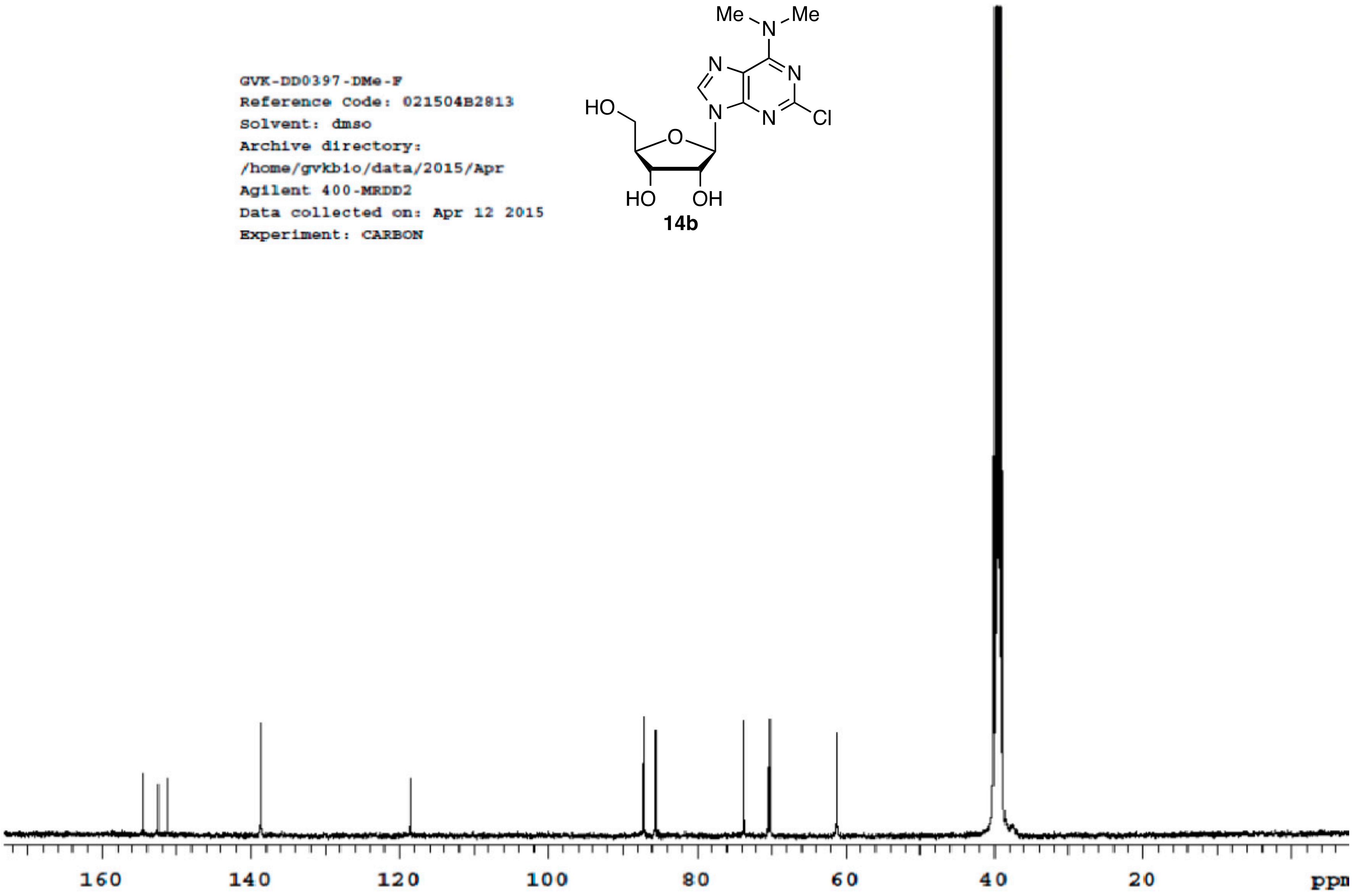
GVK-DD0397-DMe-F
Reference Code: 021504B2813
Solvent: dmso
Archive directory:
/home/gvkbio/data/2015/Apr
Agilent 400-MRDD2
Data collected on: Apr 12 2015
Experiment: CARBON
Me
N
Me
N
N
N
N
Cl
HO
O
HO
OH
14b
160
140
120
100
80
60
40
20
ppm

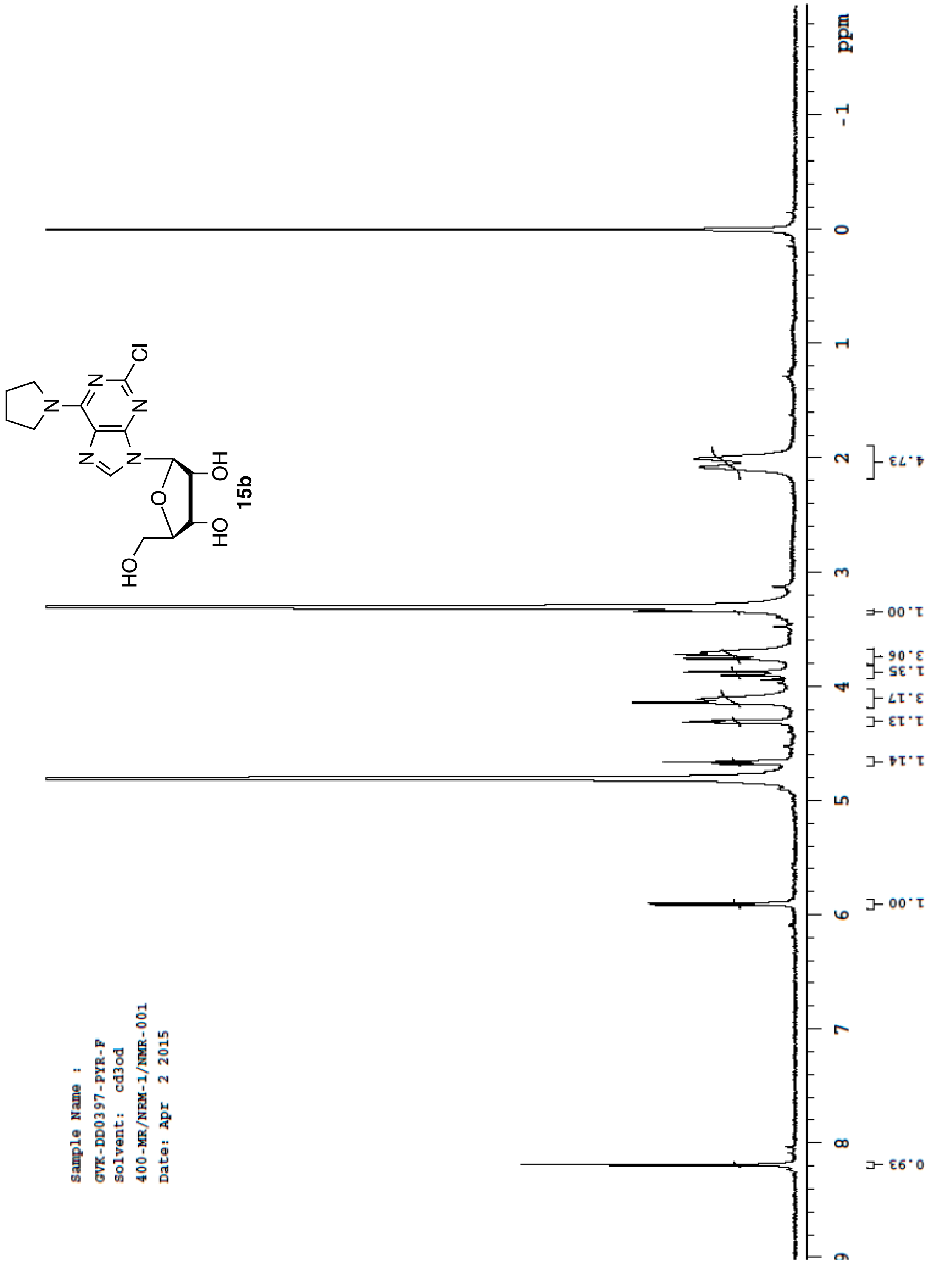
Sample Name :
GVK-DD0397-PYR-F
Solvent: cd3od
400-MR/NRM-1/NMR-001
Date: Apr 2 2015
15b
ppm

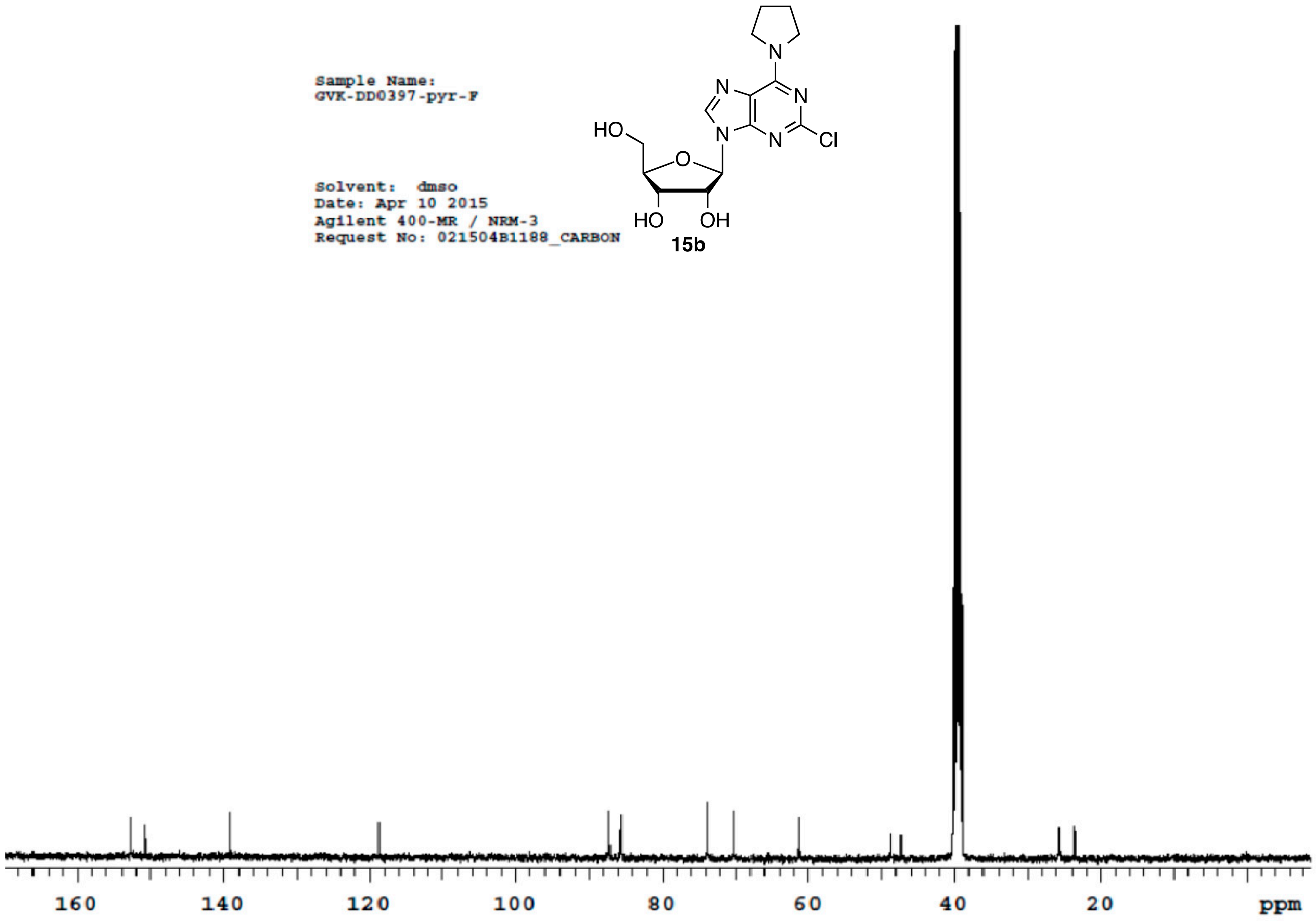
Sample Name:
GVK-DD0397-pyr-F
Solvent: dmso
Date: Apr 10 2015
Agilent 400-MR / NRM-3
Request No: 021504B1188_CARBON
15b
160
140
120
100
80
60
40
20
ppm

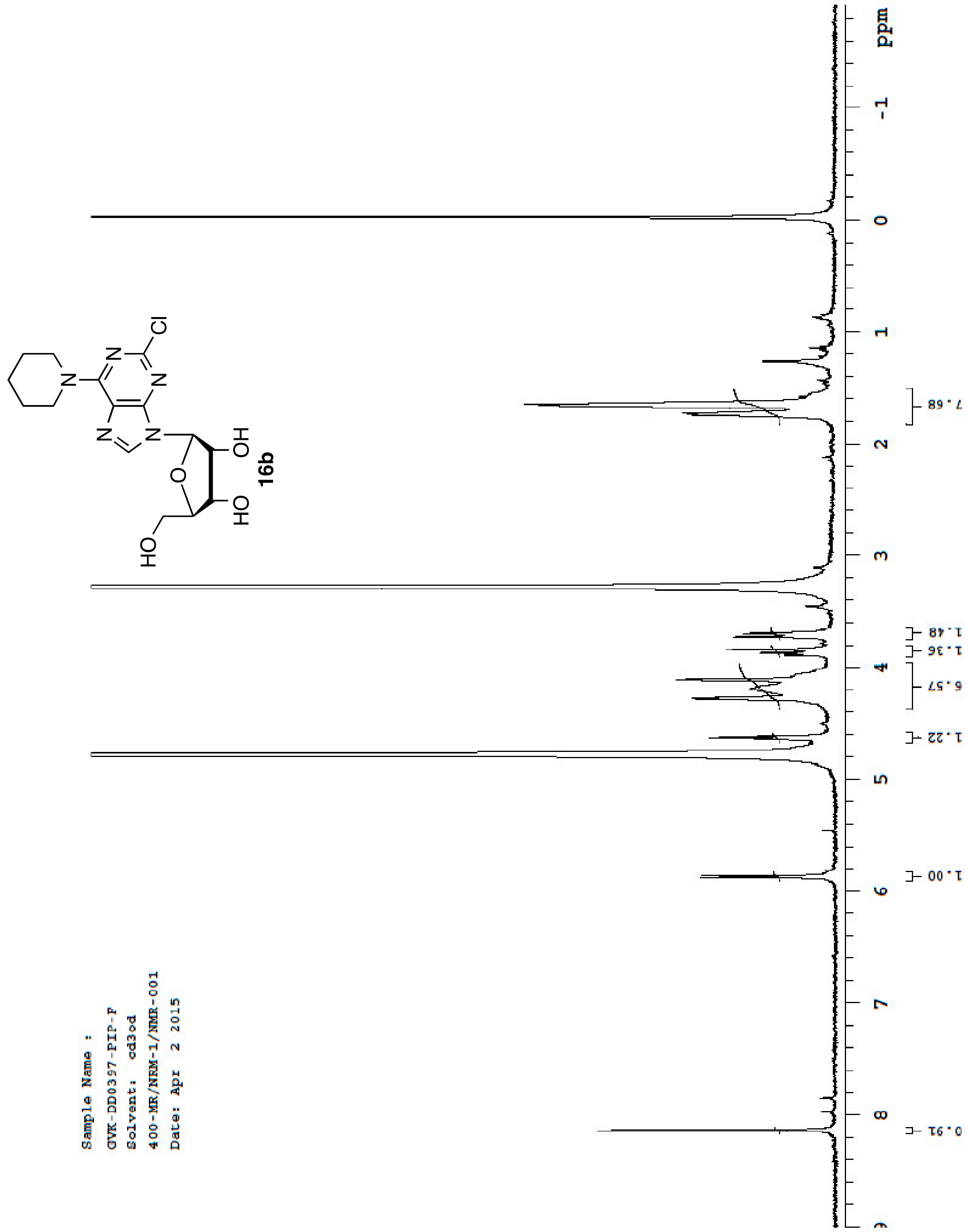
Sample Name :
GVK-DD0397-PIP-F
Solvent: cd3od
400-MR/NRM-1/NMR-001
Date: Apr 2 2015
16b
7.68
1.48
1.36
6.57
1.22
1.00
0.91
ppm
-1
0
1
2
3
4
5
6
7
8

GVK-DD0397-PIP-F
Reference Code: 021504B2820
Solvent: dmso
Archive directory:
/home/gvkbio/data/2015/Apr
Agilent 400-MRDD2
Data collected on: Apr 11 2015
Experiment: CARBON

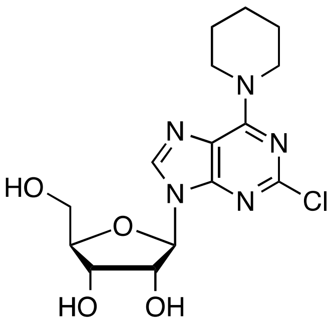

16b

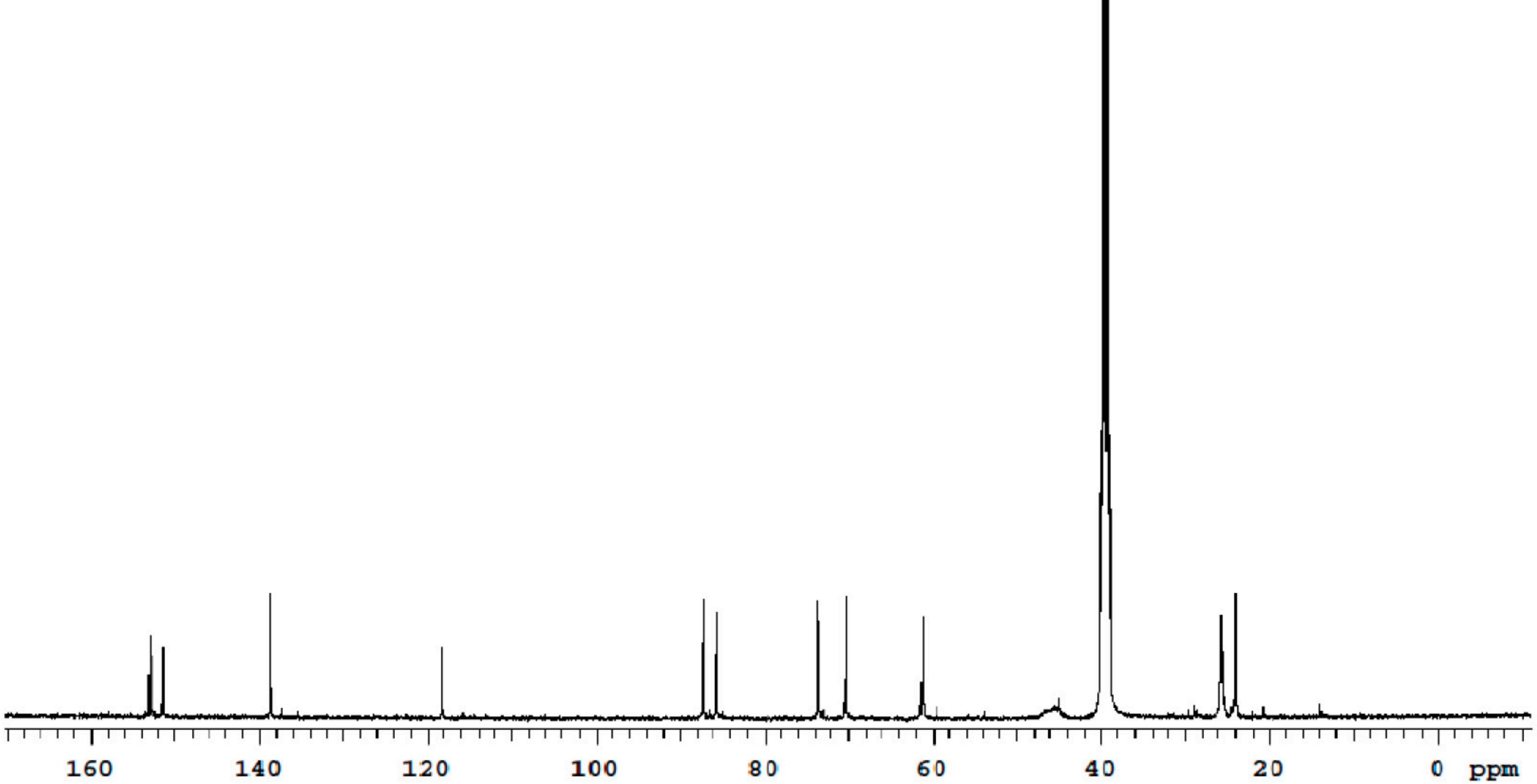

Sample Name :
GVK-DD0397-MOY-F
Solvent: cd3od
400-MR/NRM-1/NMR-001
Date: Apr 2 2015

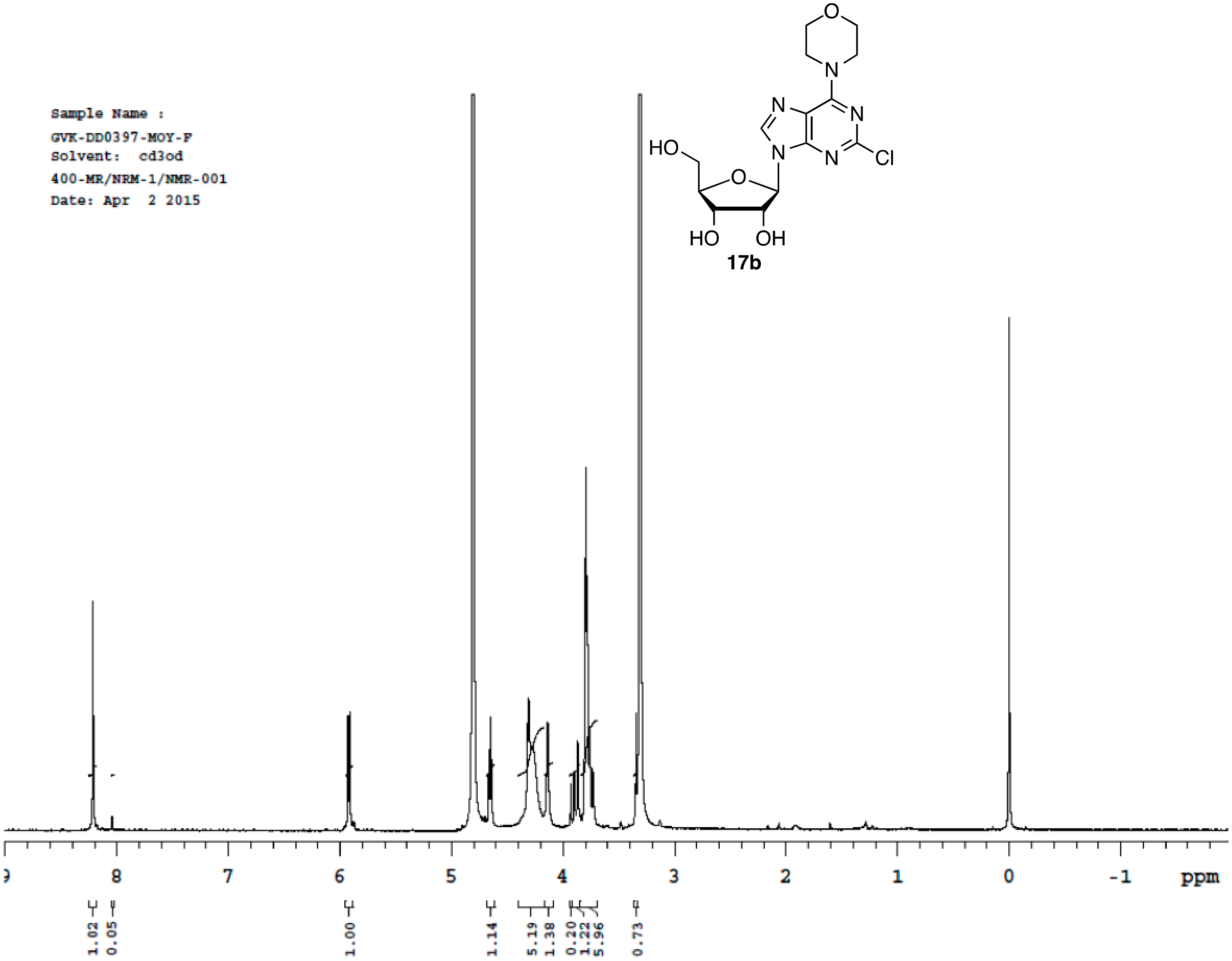

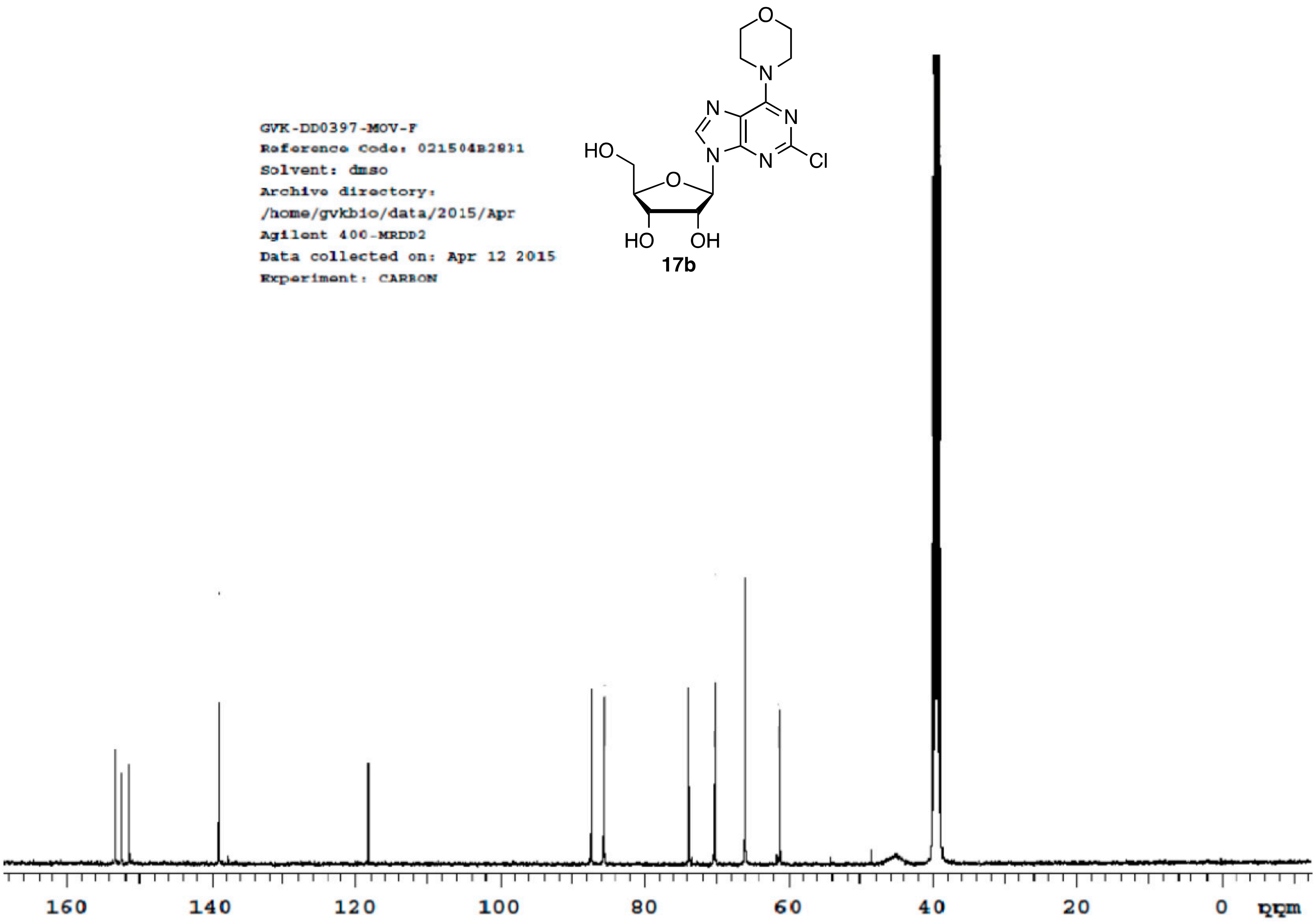
GVK-DD0397-MOV-F
Reference Code: 021504B2831
Solvent: dmso
Archive directory:
/home/gvkbio/data/2015/Apr
Agilent 400-MRDD2
Data collected on: Apr 12 2015
Experiment: CARBON
17b
160
140
120
100
80
60
40
20
0
ppm

Sample Name :
GVK-DD0397-TME-F
Solvent: cd3od
400-MR/NRM-1/NMR-001
Date: May 10 2015

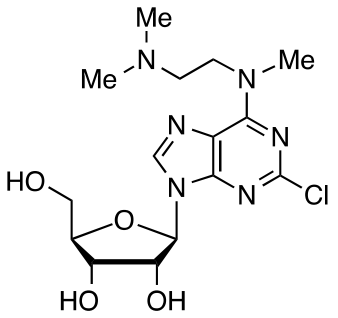

18b

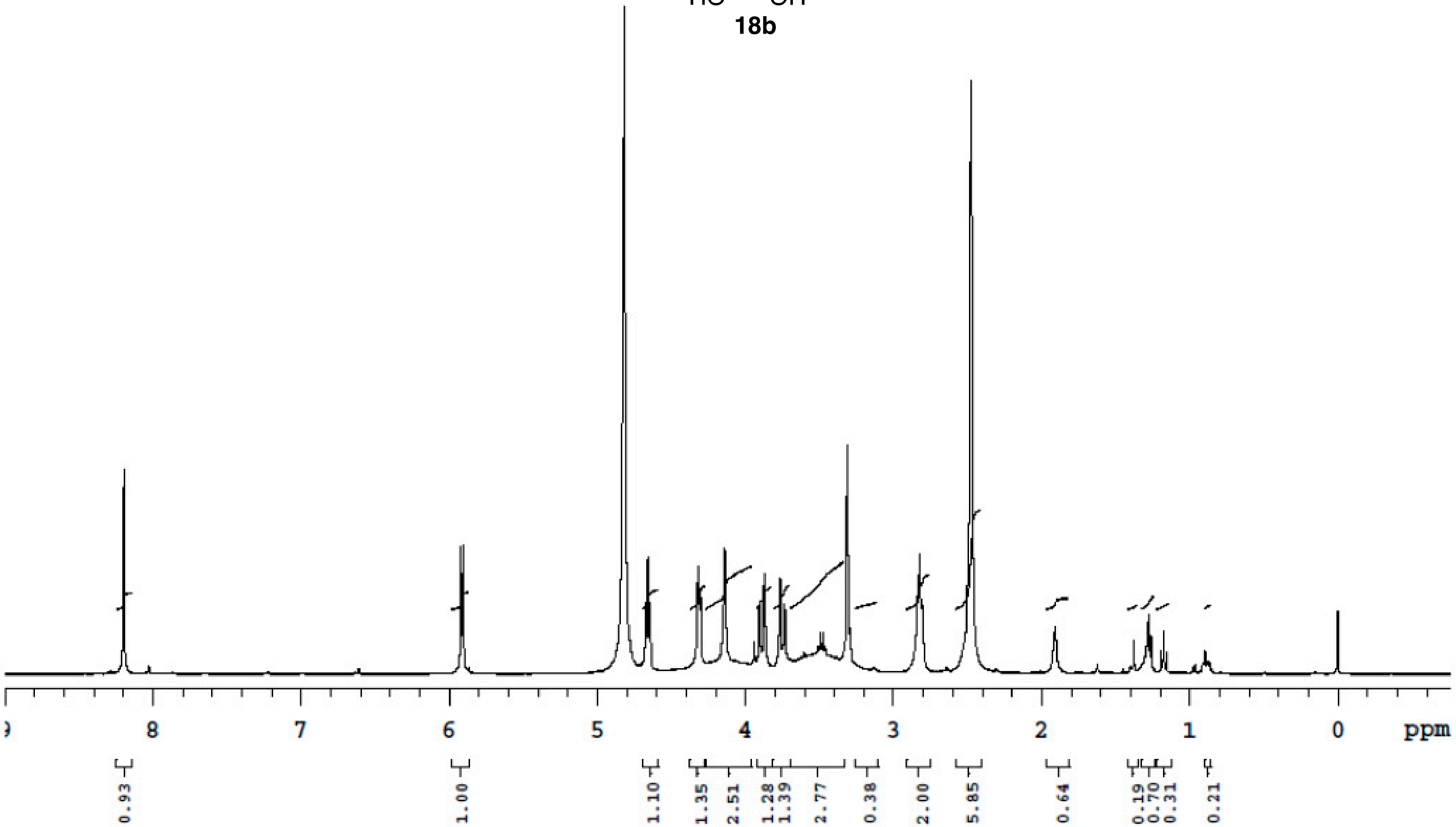

Sample Name :
GVK-DD0397-TME-F
Solvent: cd3od
400-MR/NRM-1/NMR-001
Date: May 10 2015

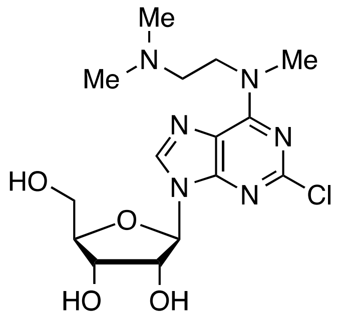

18b

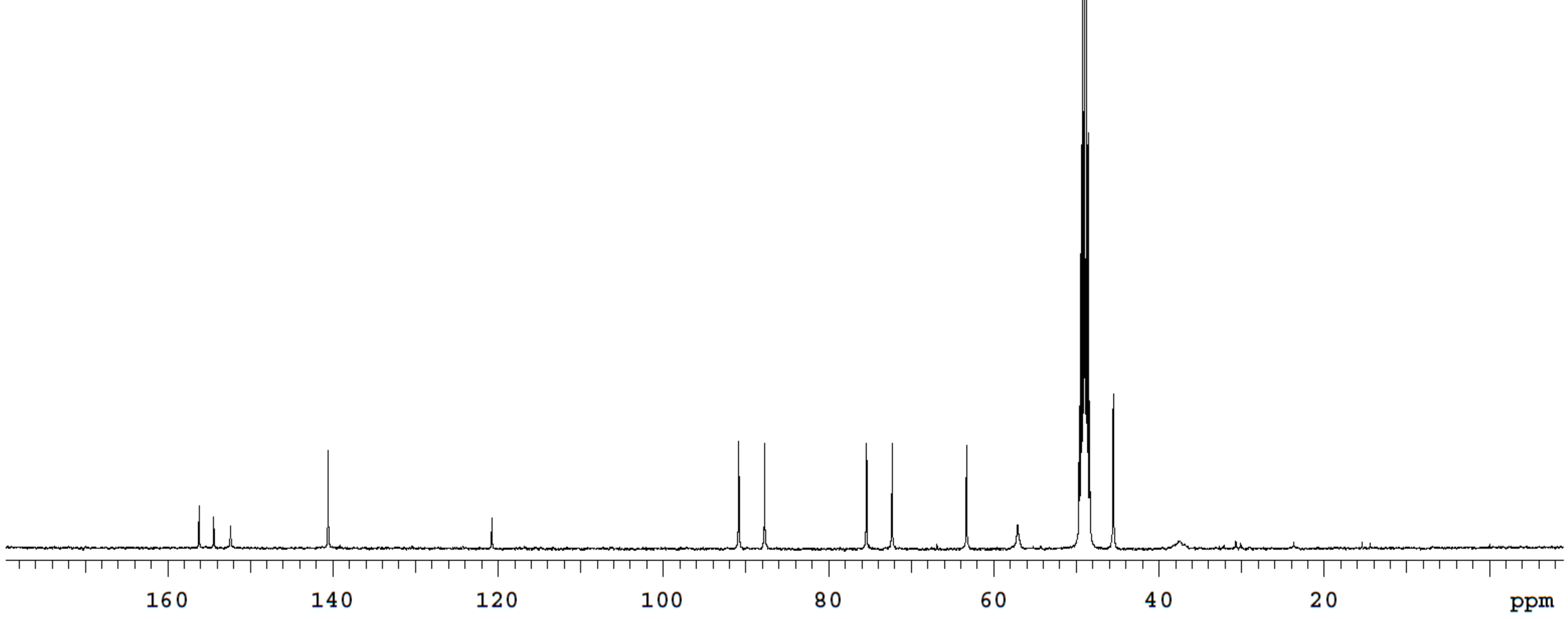

Plotname: 021505A7314_CARBON_01_plot04

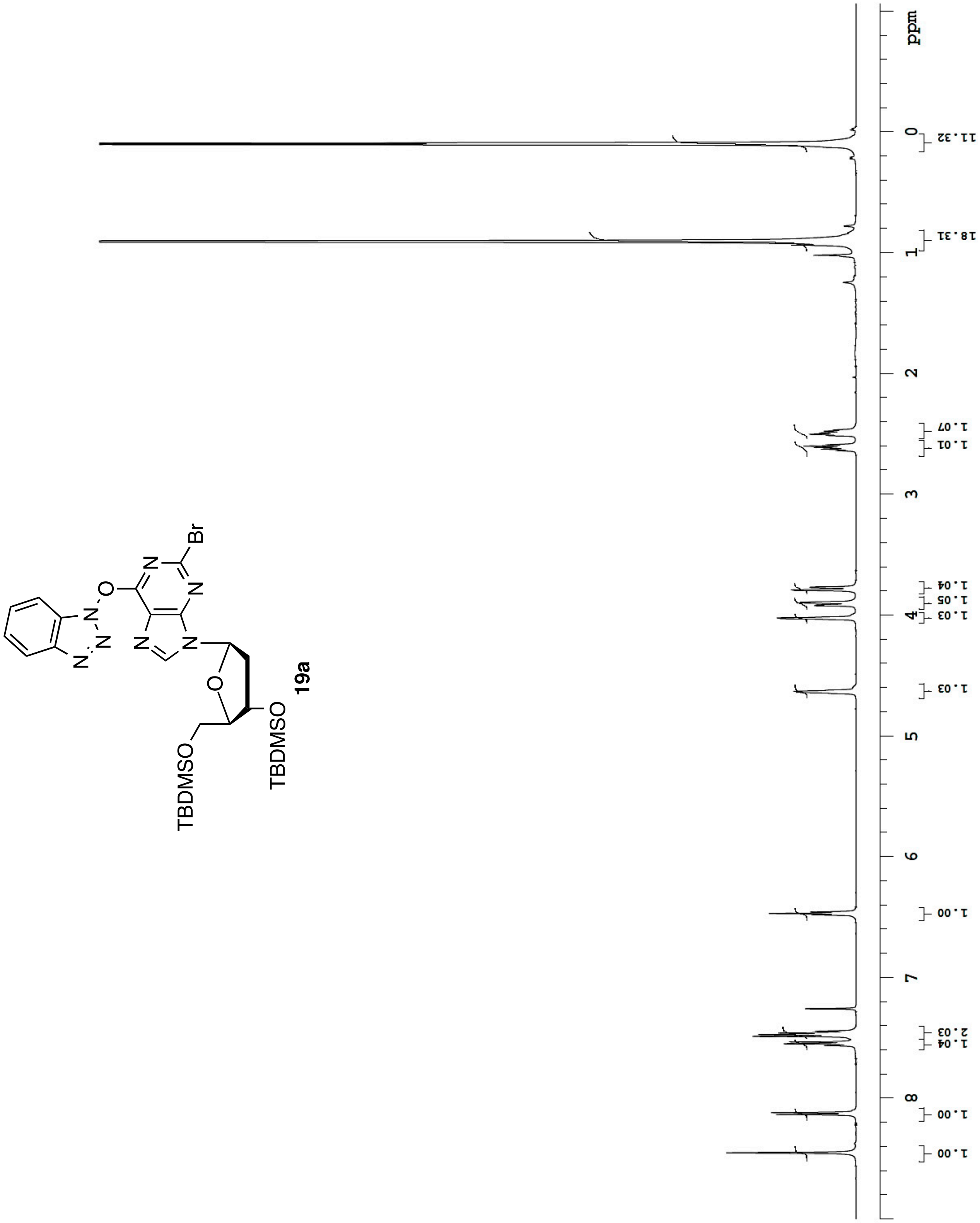

19a

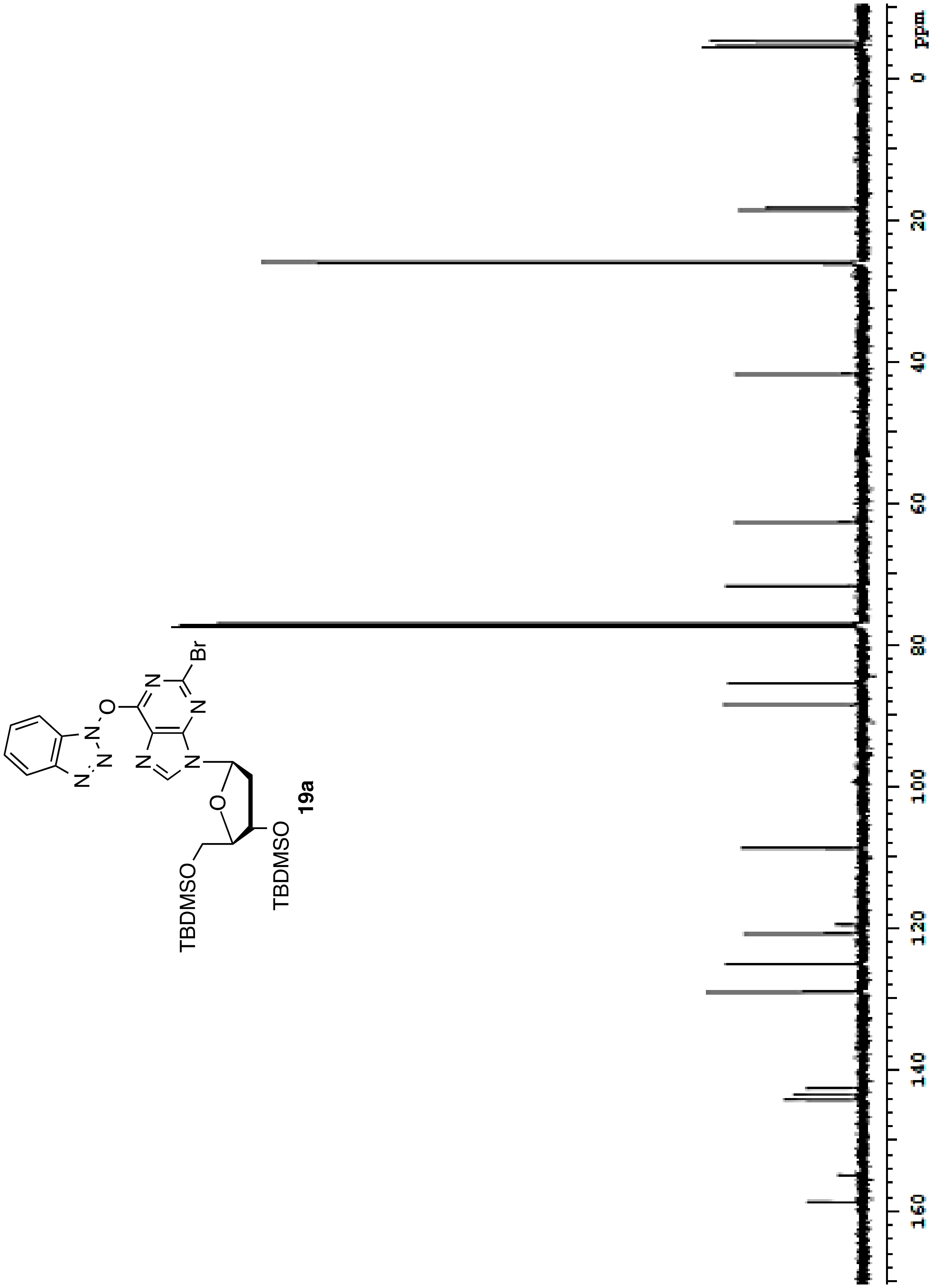
TBDMSO
TBDMSO
Br
19a
ppm
0
20
40
60
80
100
120
140
160

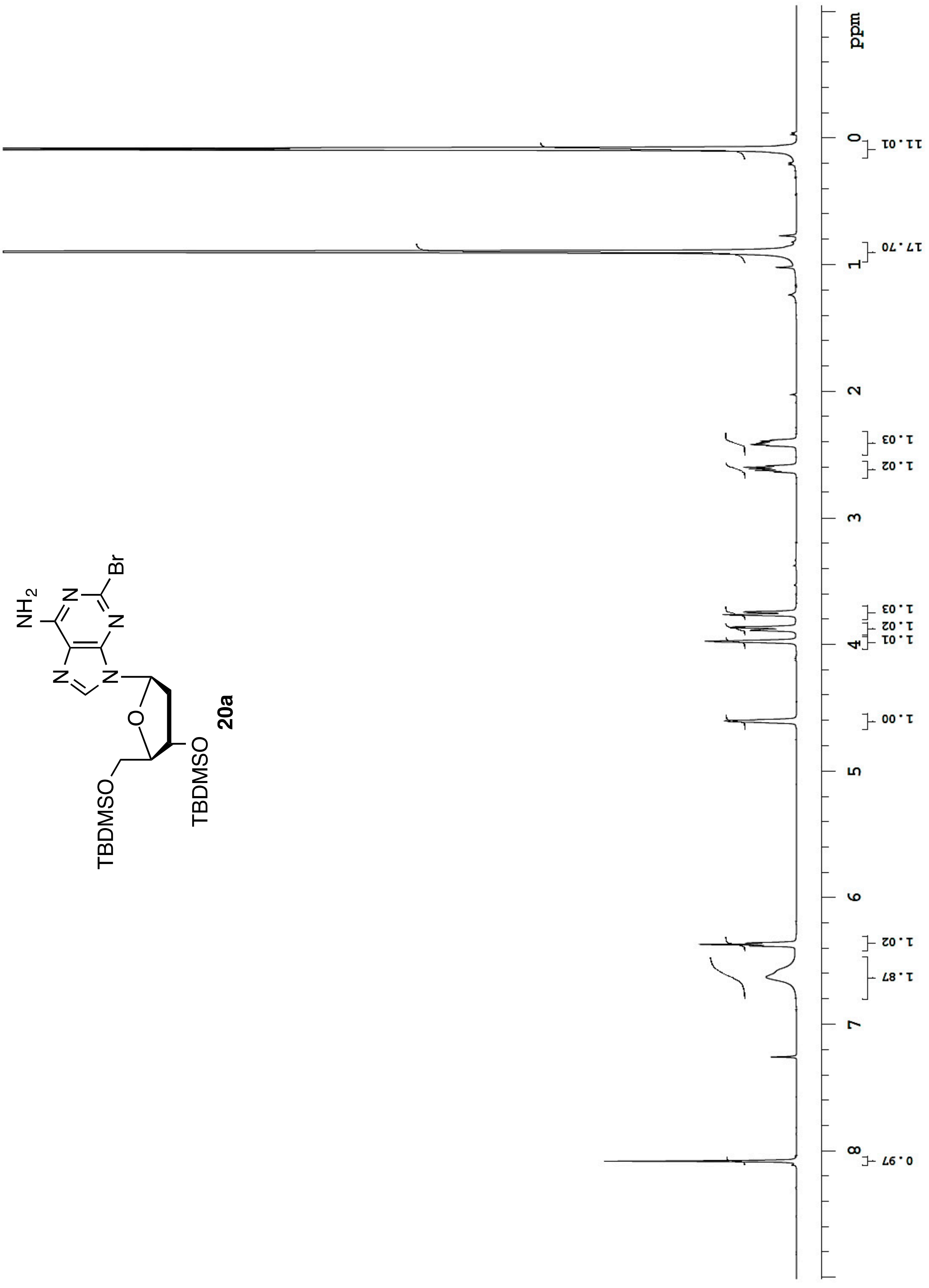
ppm
11.01
17.70
1.03
1.02
1.03
1.02
1.01
1.00
1.02
1.87
0.97
NH2
Br
TBDMSO
TBDMSO

20a

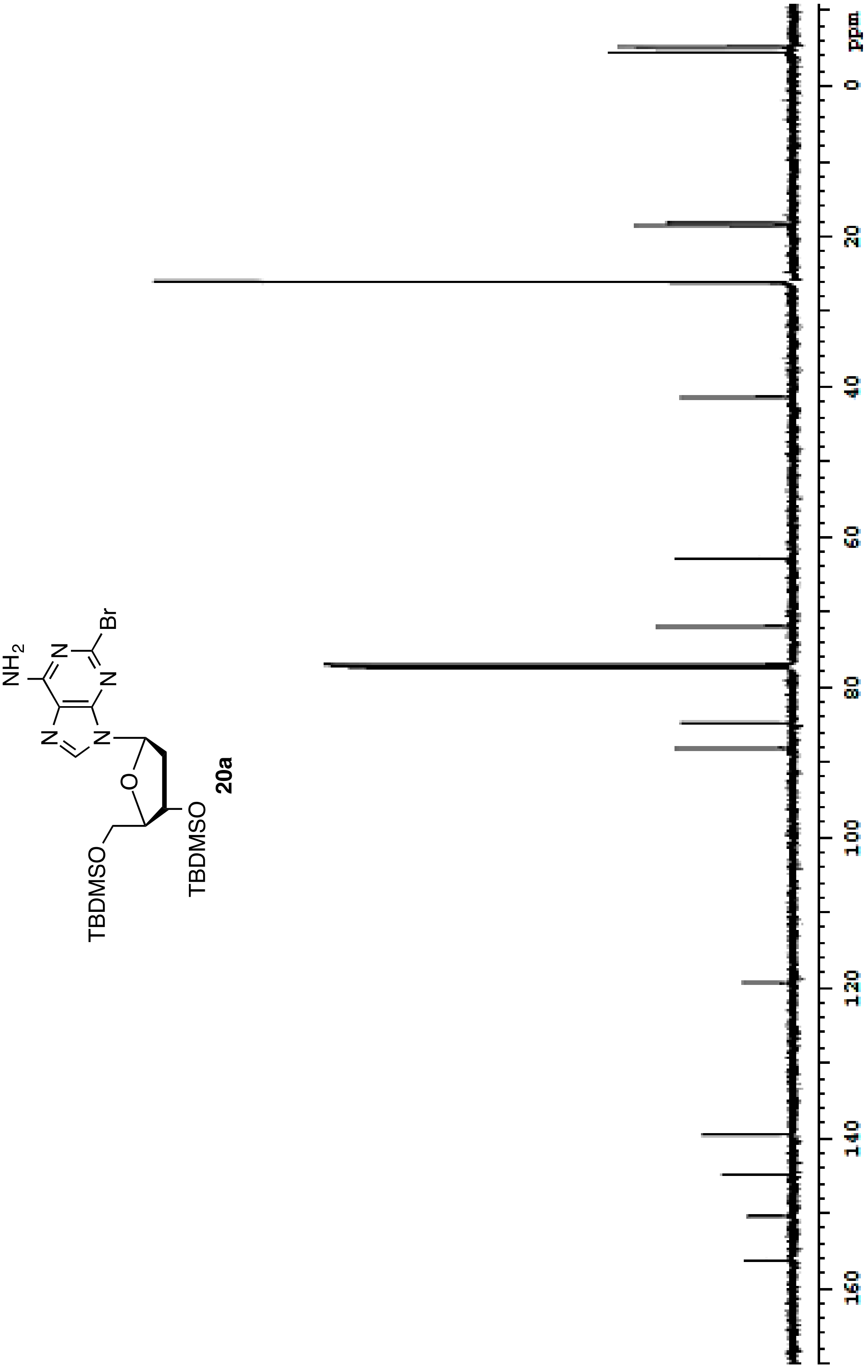
NH2
Br
N
TBDMSO
TBDMSO
20a
ppm
0
20
40
60
80
100
120
140
160

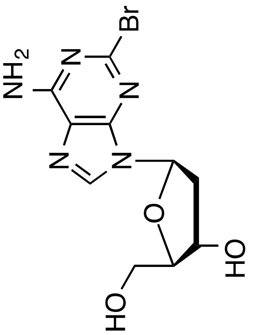
NH2
N
N
Br
N
N
O
HO
HO

21a

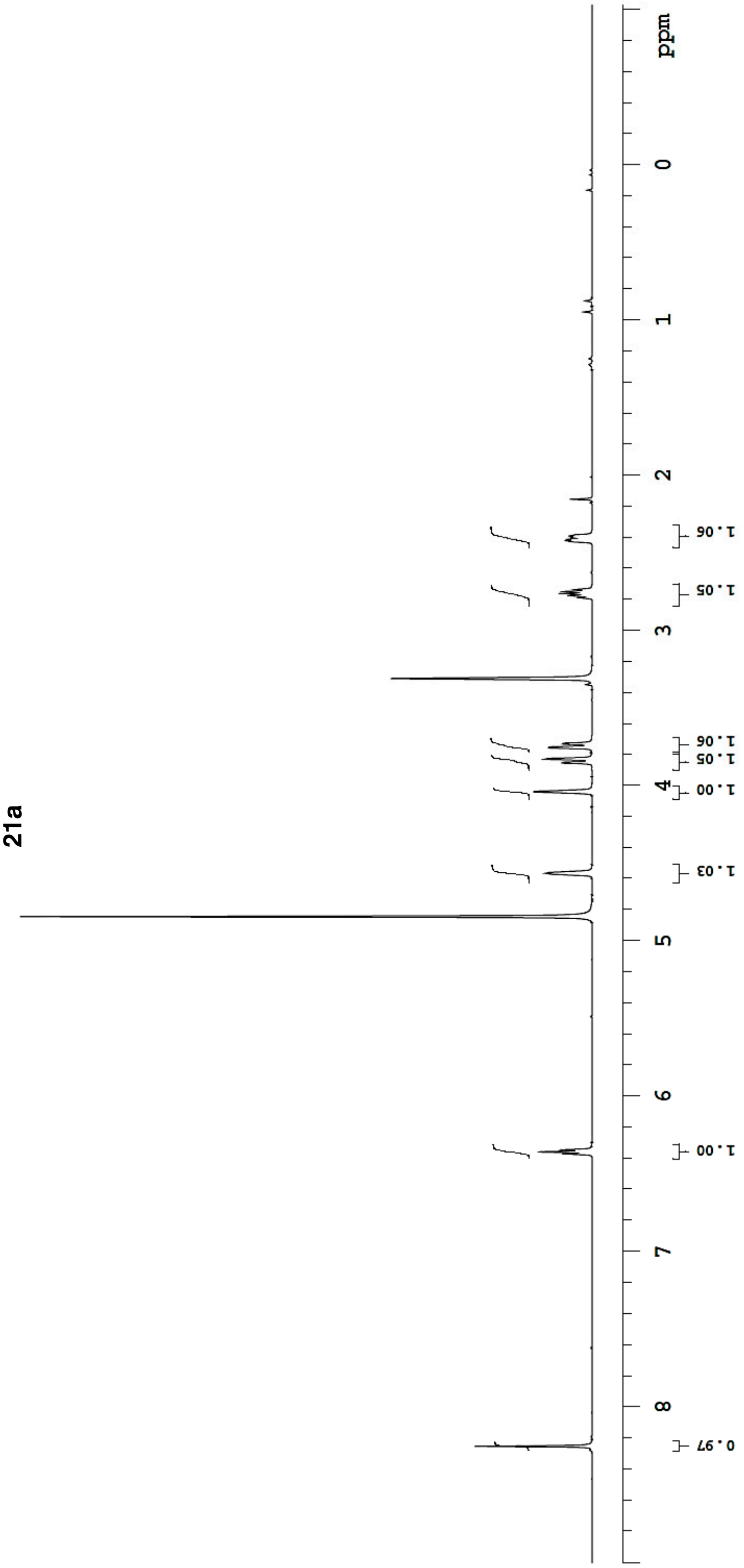
ppm
0
1
2
3
4
5
6
7
8
1.06
1.05
1.06
1.05
1.00
1.03
1.00
0.97

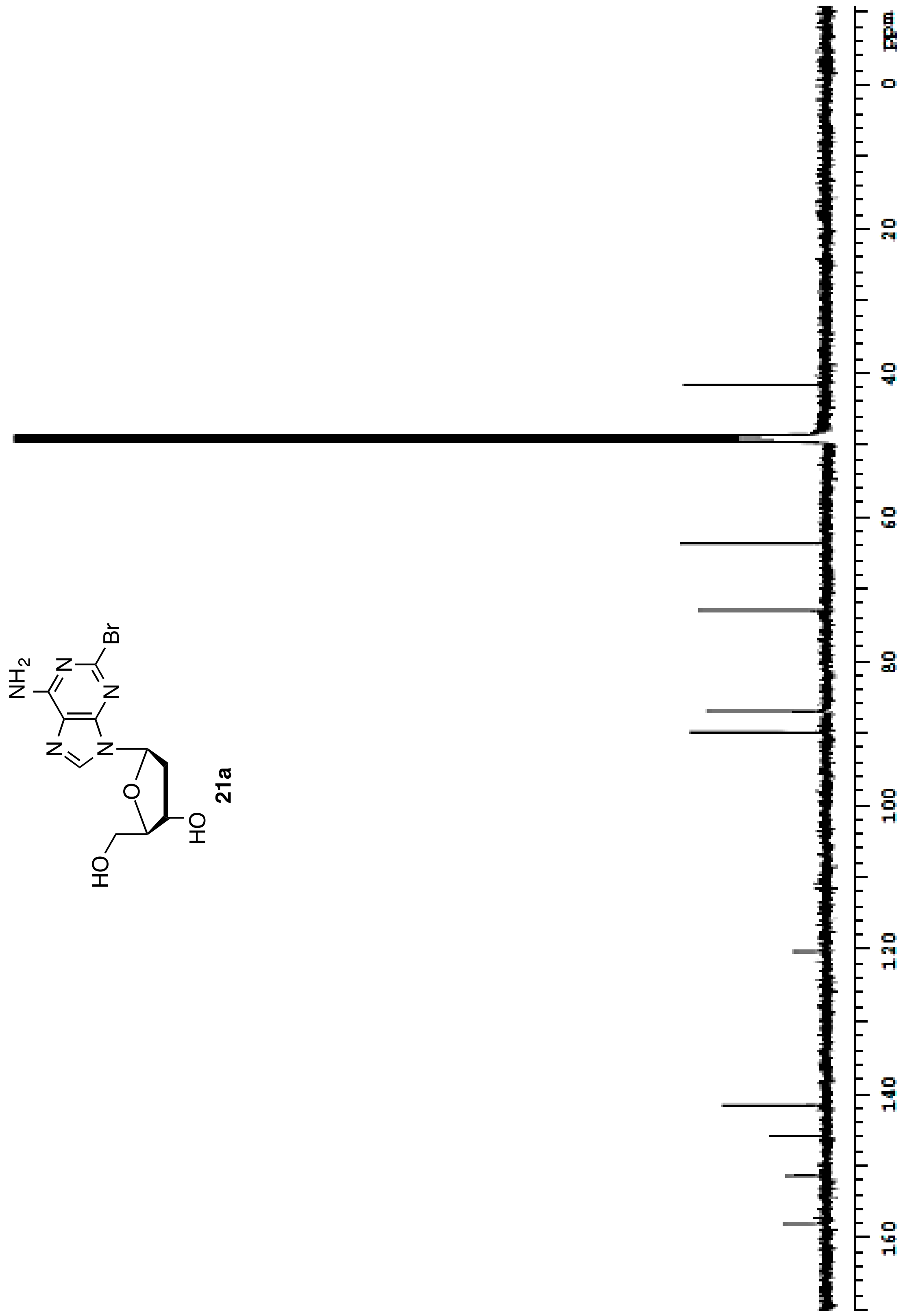

21a

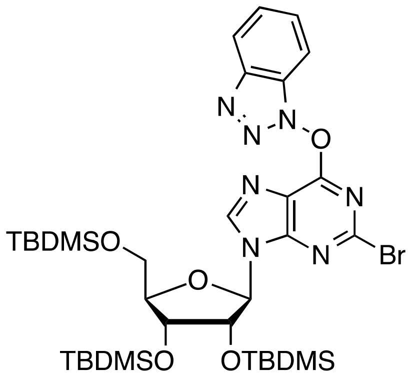
TBDMSO
TBDMSO
OTBDMS
Br
N
O

19b

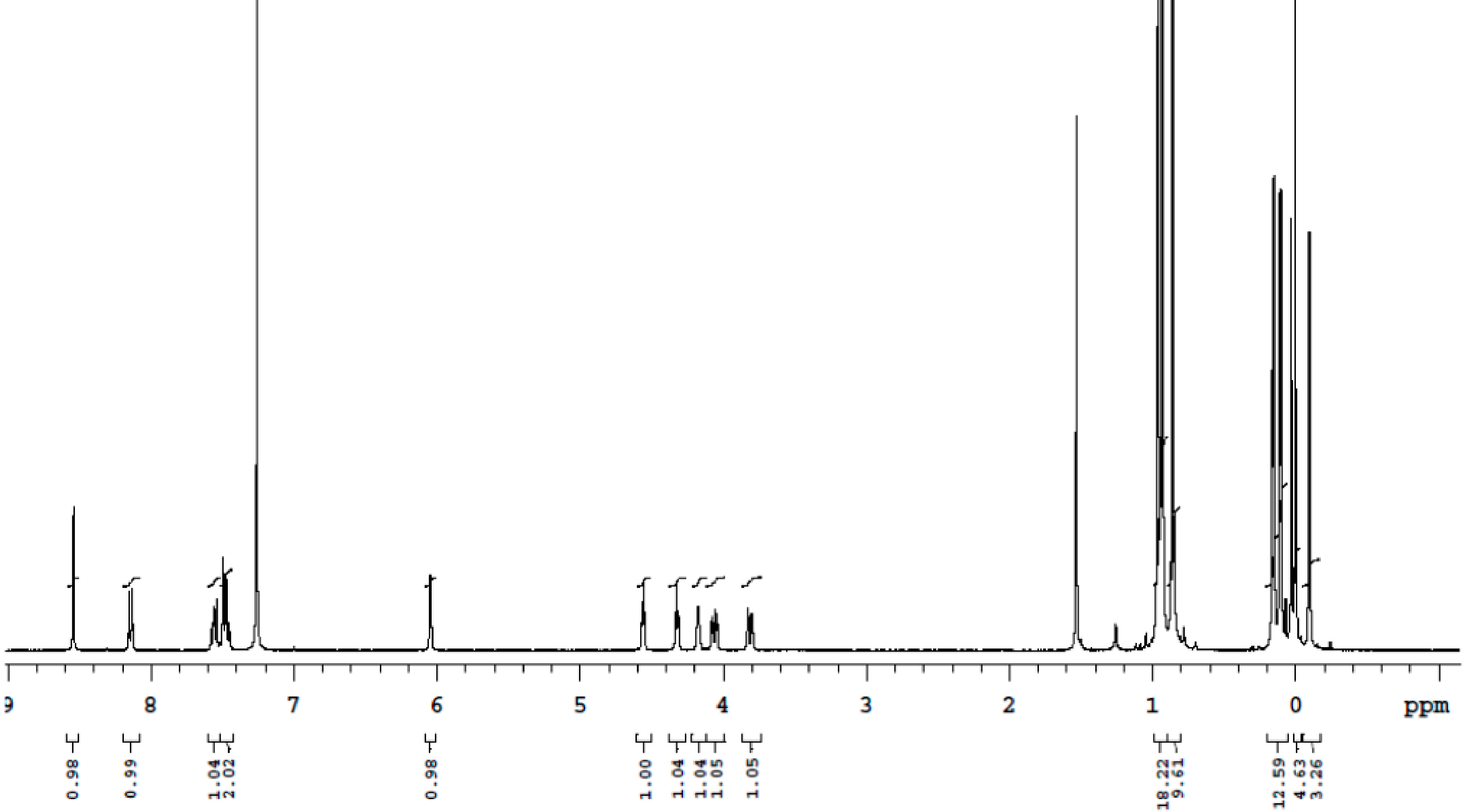
Sample Name :
GVK-DD0397-BR-T
Solvent: cdcl3
400-MR/NRM-1/NMR-001
Date: Apr 17 2015
ppm
9
8
7
6
5
4
3
2
1
0
0.98
0.99
1.04
2.02
0.98
1.00
1.04
1.04
1.05
1.05
18.22
9.61
12.59
4.63
3.26

Sample Name :
GVK-DD0397-BR-OBT-OTB

Solvent: cdcl3
Date: Apr 22 2015
400 VNMRS/NRM-1/NMR-002

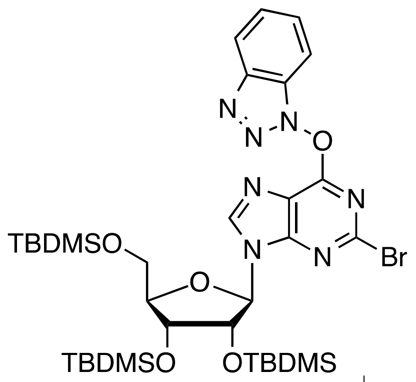

19b

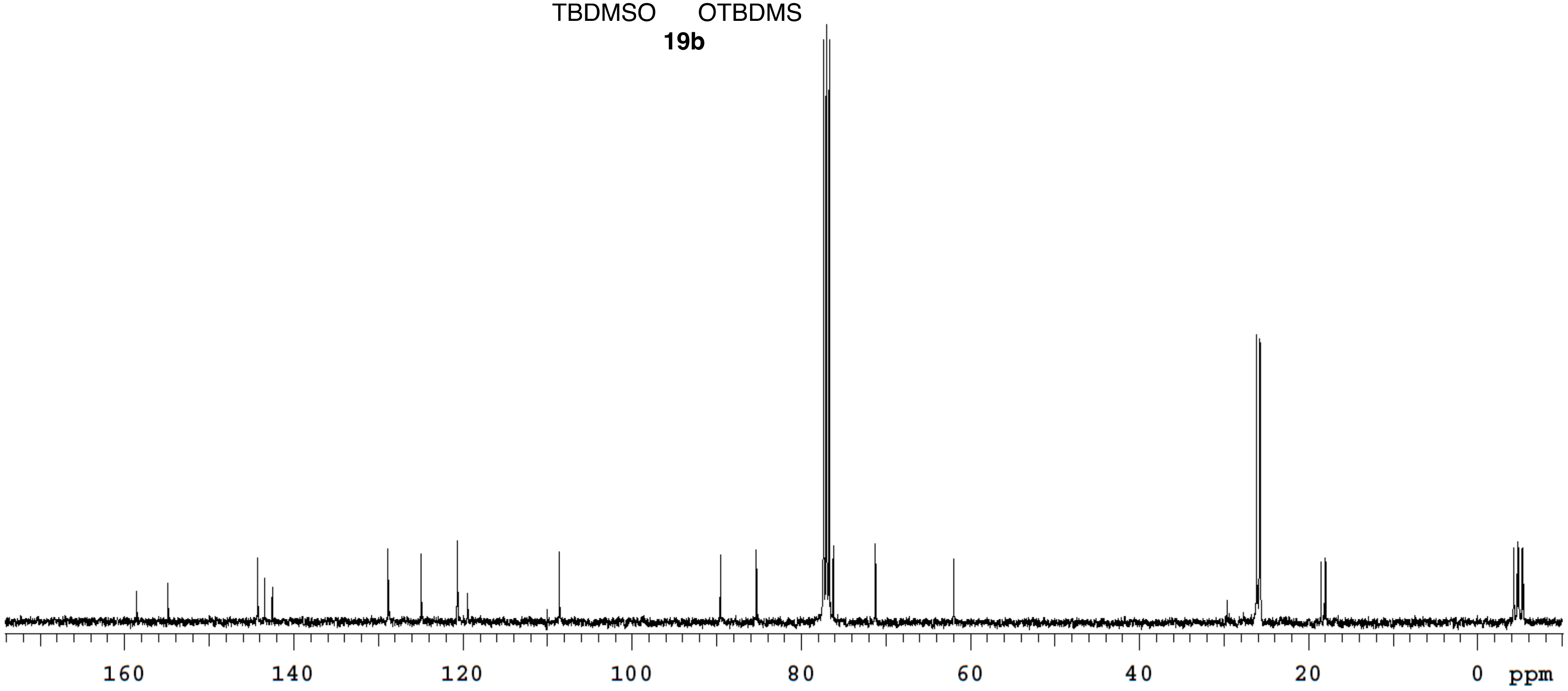

Plotname: 021504C5846_CARBON_01_plot05

Sample Name :
GVK-DD0397-BR-NH2-TBS

Solvent: cdcl3
Date: Apr 22 2015
400 VNMRS/NRM-1/NMR-002

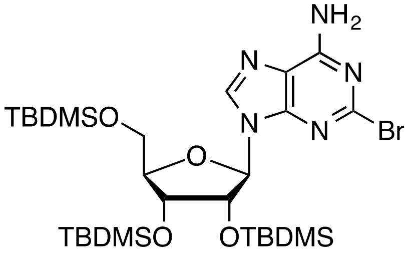

20b

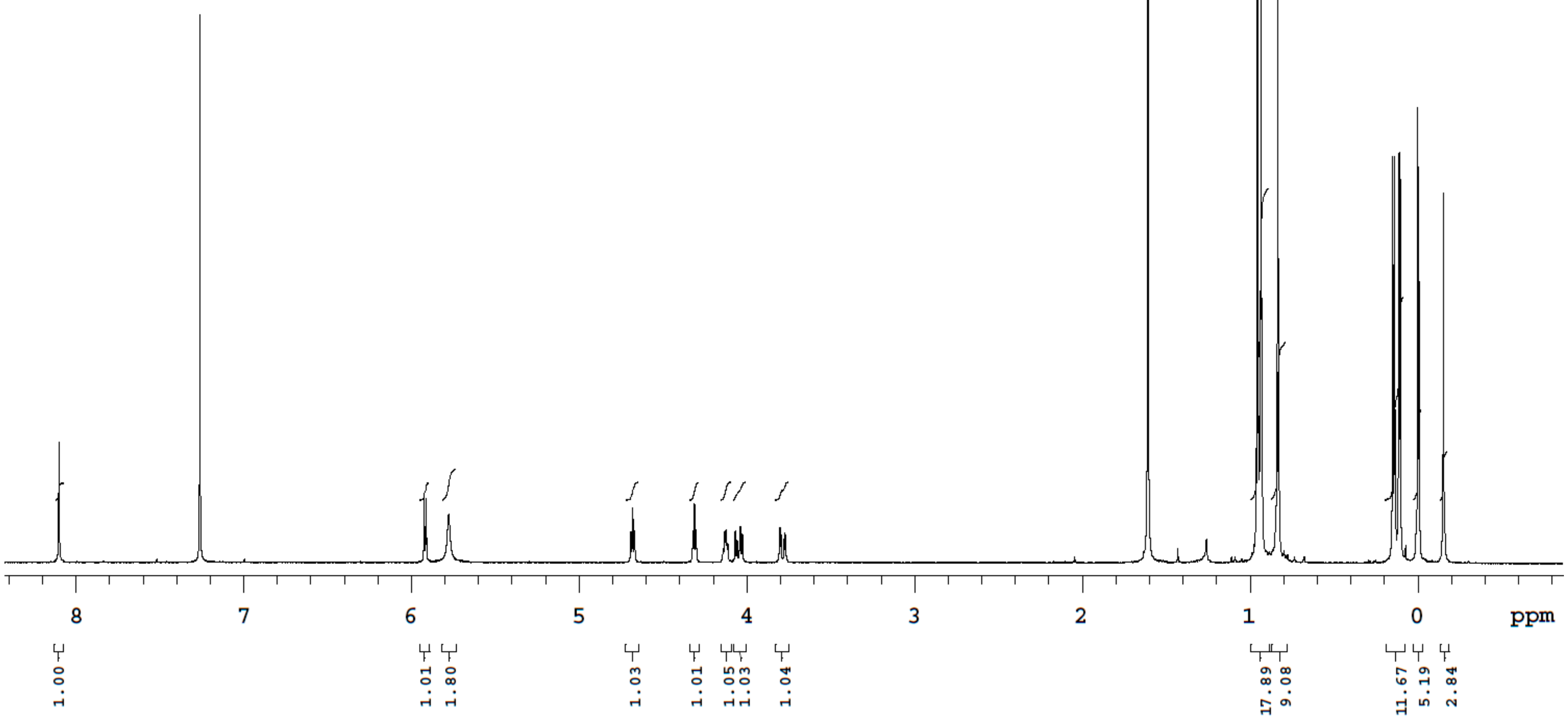

Plotname: 021504C5838_PROTON_01_plot07

Sample Name :
GVK-DD0397-BR-NH2-CTB

Solvent: cdcl3
VNMRS-400/NRM-1/NMR-002
Date: Apr 22 2015

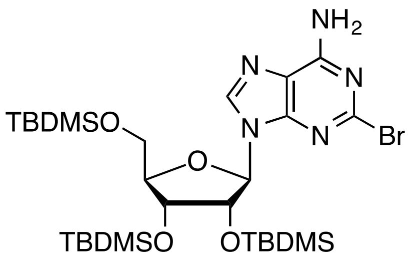

20b

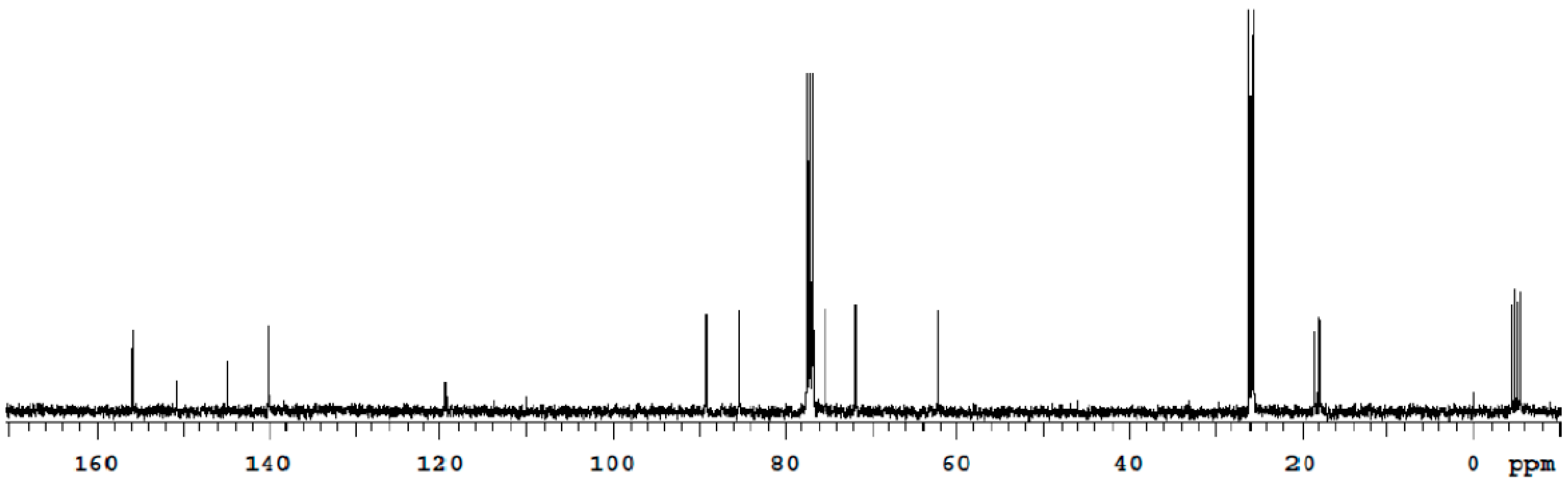

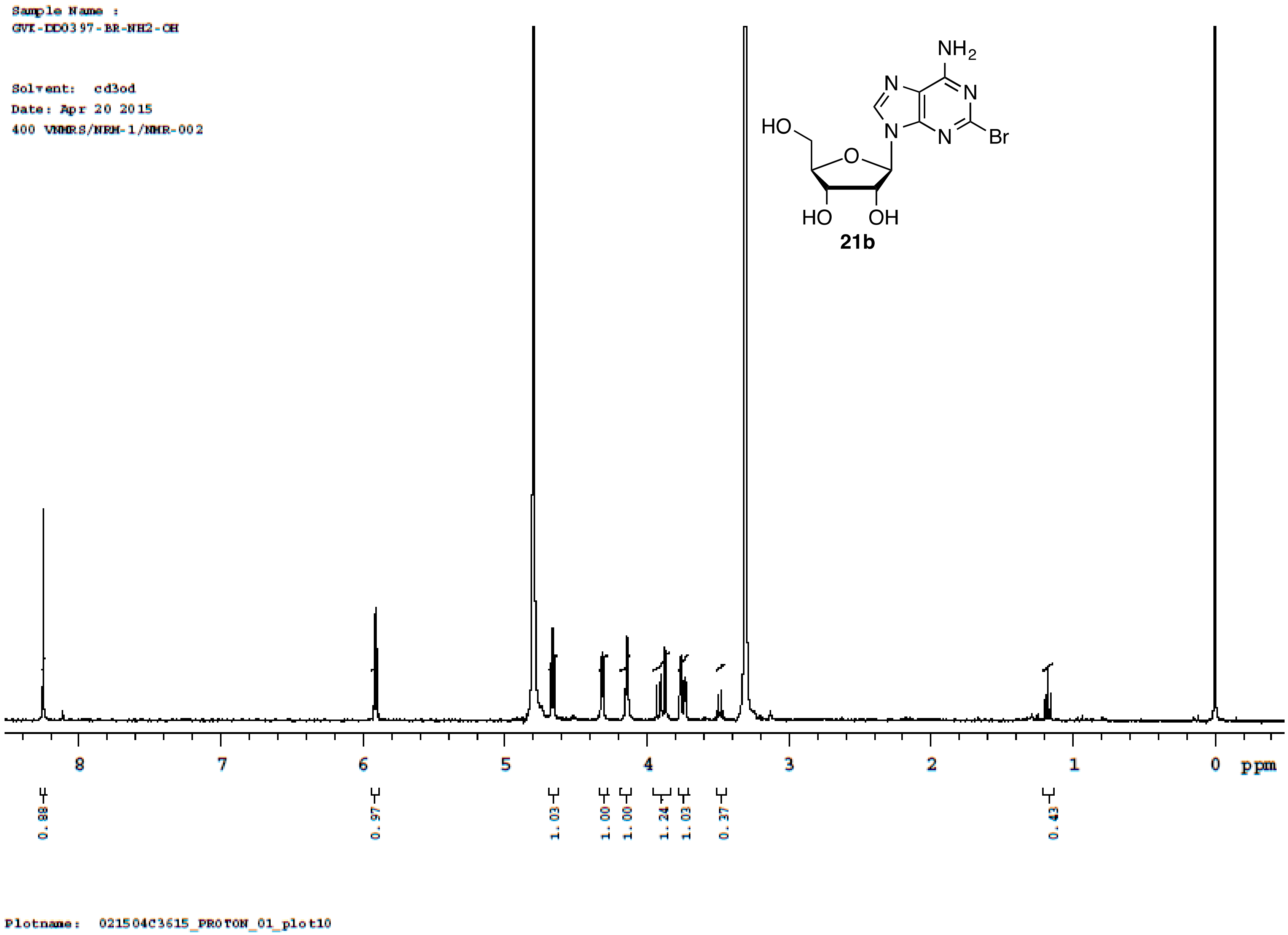

Sample Name :
GVI-DD0397-BR-NH2-OH
Solvent: cd3od
Date: Apr 20 2015
400 VNMRS/NRM-1/NMR-002
8
7
6
5
4
3
2
1
0
ppm
0.88
0.97
1.03
1.00
1.00
1.24
1.03
0.37
0.43
Plotname: 021504C3615_PROTON_01_plot10
NH2
N
N
N
N
Br
HO
O
HO
OH

21b

Sample Name :
GVK-DD0397-BR-NH2-OH

Solvent: dmso
VNMRS-400/NRM-1/NMR-002
Date: Apr 22 2015

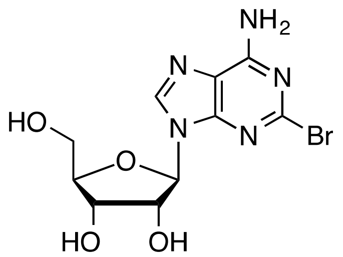

21b

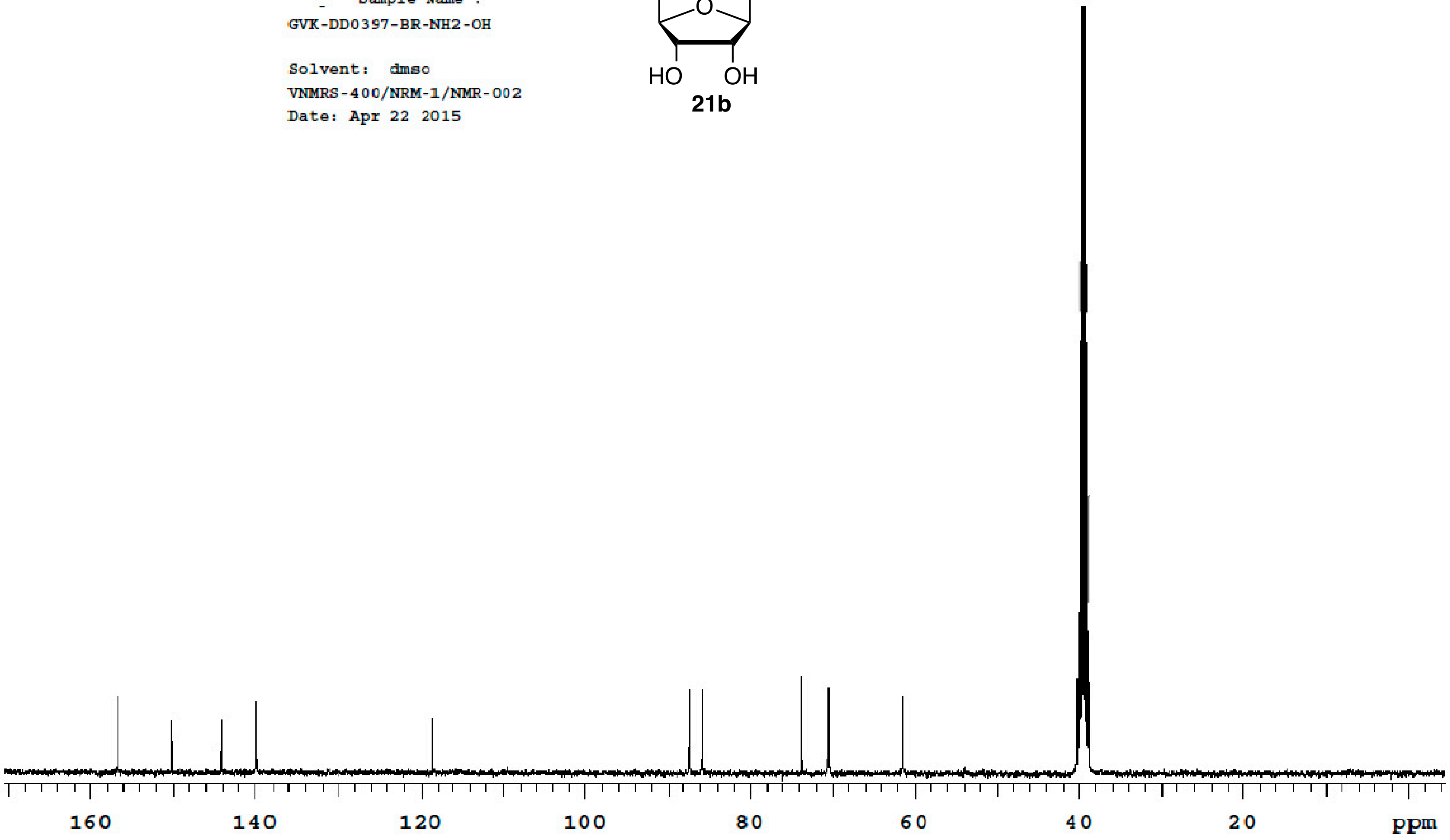

Supplement: Supplementary file 1 [file molecules-20-18437-s001.pdf]
