# Supplementary material for: Cladribine Analogues via O6-(Benzotriazolyl) Derivatives of Guanine Nucleosides
Source: Molecules. Author manuscript; Available in PMC 2016 Apr 22. (PMC4841790; doi:10.3390/molecules201018437)

Cladribine Analogues via O^6-(Benzotriazolyl) Derivatives of Guanine Nucleosides

Sakilam Satishkumar, Prasanna K. Vuram, Siva Subrahmanyam Relangi, Venkateshwarlu Gurram, Hong Zhou, Robert J. Kreitman, Michelle M. Martínez Montemayor, Lijia Yang, Muralidharan Kaliyaperumal, Somesh Sharma, Narender Pottabathini, and Mahesh K. Lakshman*

Table of Contents

Information	Page
500 MHz ^{1}H NMR spectrum of compound **4a** in $CDCl_3$	S-3
125 MHz ^{13}C NMR spectrum of compound **4a** in $CDCl_3$	S-4
500 MHz ^{1}H NMR spectrum of compound **5a** in $CDCl_3$	S-5
125 MHz ^{13}C NMR spectrum of compound **5a** in $CDCl_3$	S-6
500 MHz ^{1}H NMR spectrum of compound **6a** in $CDCl_3$	S-7
125 MHz ^{13}C NMR spectrum of compound **6a** in $CDCl_3$	S-8
500 MHz ^{1}H NMR spectrum of compound **7a** in $CDCl_3$	S-9
125 MHz ^{13}C NMR spectrum of compound **7a** in $CDCl_3$	S-10
500 MHz ^{1}H NMR spectrum of compound **8a** in $CDCl_3$	S-11
125 MHz ^{13}C NMR spectrum of compound **8a** in $CDCl_3$	S-12
500 MHz ^{1}H NMR spectrum of compound **9a** in $CDCl_3$	S-13
125 MHz ^{13}C NMR spectrum of compound **9a** in $CDCl_3$	S-14
500 MHz ^{1}H NMR spectrum of compound **10a** in $CDCl_3$	S-15
125 MHz ^{13}C NMR spectrum of compound **10a** in $CDCl_3$	S-16
500 MHz ^{1}H NMR spectrum of compound **11a** in $CDCl_3$	S-17
125 MHz ^{13}C NMR spectrum of compound **11a** in $CDCl_3$	S-18
400 MHz ^{1}H NMR spectrum of compound **4b** in $CDCl_3$	S-19
100 MHz ^{13}C NMR spectrum of compound **4b** in $CDCl_3$	S-20
400 MHz ^{1}H NMR spectrum of compound **5b** in $CDCl_3$	S-21
100 MHz ^{13}C NMR spectrum of compound **5b** in $CDCl_3$	S-22
400 MHz ^{1}H NMR spectrum of compound **6b** in $CDCl_3$	S-23
100 MHz ^{13}C NMR spectrum of compound **6b** in $CDCl_3$	S-24
400 MHz ^{1}H NMR spectrum of compound **7b** in $CDCl_3$	S-25
100 MHz ^{13}C NMR spectrum of compound **7b** in $CDCl_3$	S-26
400 MHz ^{1}H NMR spectrum of compound **8b** in $CDCl_3$	S-27
100 MHz ^{13}C NMR spectrum of compound **8b** in $CDCl_3$	S-28
400 MHz ^{1}H NMR spectrum of compound **9b** in $CDCl_3$	S-29
100 MHz ^{13}C NMR spectrum of compound **9b** in $CDCl_3$	S-30
400 MHz ^{1}H NMR spectrum of compound **10b** in $CDCl_3$	S-31
100 MHz ^{13}C NMR spectrum of compound **10b** in $CDCl_3$	S-32
400 MHz ^{1}H NMR spectrum of compound **11b** in $CDCl_3$	S-33
100 MHz ^{13}C NMR spectrum of compound **11b** in $CDCl_3$	S-34

500 MHz ^{1}H NMR spectrum of compound **12a** in CD_3OD	S-35
125 MHz ^{13}C NMR spectrum of compound **12a** in CD_3OD	S-36
500 MHz ^{1}H NMR spectrum of compound **13a** in CD_3OD	S-37
125 MHz ^{13}C NMR spectrum of compound **13a** in CD_3OD	S-38
500 MHz ^{1}H NMR spectrum of compound **14a** in CD_3OD	S-39
125 MHz ^{13}C NMR spectrum of compound **14a** in CD_3OD	S-40
500 MHz ^{1}H NMR spectrum of compound **15a** in CD_3OD	S-41
125 MHz ^{13}C NMR spectrum of compound **15a** in CD_3OD	S-42
500 MHz ^{1}H NMR spectrum of compound **16a** in CD_3OD	S-43
125 MHz ^{13}C NMR spectrum of compound **16a** in CD_3OD	S-44
500 MHz ^{1}H NMR spectrum of compound **17a** in CD_3OD	S-45
125 MHz ^{13}C NMR spectrum of compound **17a** in CD_3OD	S-46
500 MHz ^{1}H NMR spectrum of compound **18a** in CD_3OD	S-47
125 MHz ^{13}C NMR spectrum of compound **18a** in CD_3OD	S-48
400 MHz ^{1}H NMR spectrum of compound **12b** in CD_3OD	S-49
100 MHz ^{13}C NMR spectrum of compound **12b** in DMSO-d_6	S-50
400 MHz ^{1}H NMR spectrum of compound **13b** in CD_3OD	S-51
100 MHz ^{13}C NMR spectrum of compound **13b** in DMSO-d_6	S-52
400 MHz ^{1}H NMR spectrum of compound **14b** in CD_3OD	S-53
100 MHz ^{13}C NMR spectrum of compound **14b** in DMSO-d_6	S-54
400 MHz ^{1}H NMR spectrum of compound **15b** in CD_3OD	S-55
100 MHz ^{13}C NMR spectrum of compound **15b** in DMSO-d_6	S-56
400 MHz ^{1}H NMR spectrum of compound **16b** in CD_3OD	S-57
100 MHz ^{13}C NMR spectrum of compound **16b** in DMSO-d_6	S-58
400 MHz ^{1}H NMR spectrum of compound **17b** in CD_3OD	S-59
100 MHz ^{13}C NMR spectrum of compound **17b** in DMSO-d_6	S-60
400 MHz ^{1}H NMR spectrum of compound **18b** in CD_3OD	S-61
100 MHz ^{13}C NMR spectrum of compound **18b** in CD_3OD	S-62
500 MHz ^{1}H NMR spectrum of compound **19a** in $CDCl_3$	S-63
125 MHz ^{13}C NMR spectrum of compound **19a** in $CDCl_3$	S-64
500 MHz ^{1}H NMR spectrum of compound **20a** in $CDCl_3$	S-65
125 MHz ^{13}C NMR spectrum of compound **20a** in $CDCl_3$	S-66
500 MHz ^{1}H NMR spectrum of compound **21a** in CD_3OD	S-67
125 MHz ^{13}C NMR spectrum of compound **21a** in CD_3OD	S-68
400 MHz ^{1}H NMR spectrum of compound **19b** in $CDCl_3$	S-69
100 MHz ^{13}C NMR spectrum of compound **19b** in $CDCl_3$	S-70
400 MHz ^{1}H NMR spectrum of compound **20b** in $CDCl_3$	S-71
100 MHz ^{13}C NMR spectrum of compound **20b** in $CDCl_3$	S-72
400 MHz ^{1}H NMR spectrum of compound **21b** in CD_3OD	S-73
100 MHz ^{13}C NMR spectrum of compound **21b** in DMSO-d_6	S-74

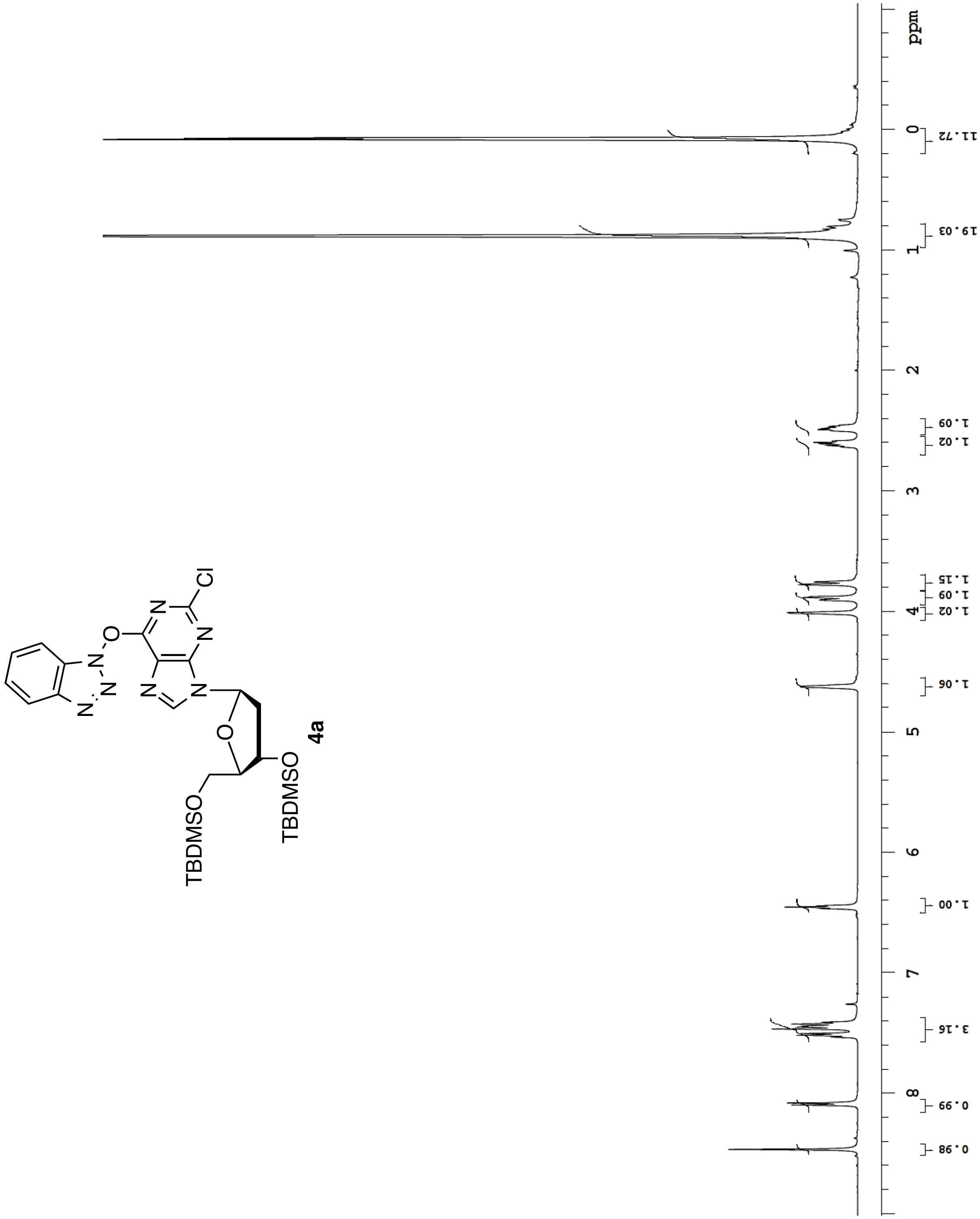
ppm
0
1
2
3
4
5
6
7
8
11.72
19.03
1.09
1.02
1.15
1.09
1.02
1.06
1.00
3.16
0.99
0.98
TBDMSO
TBDMSO
Cl

4a

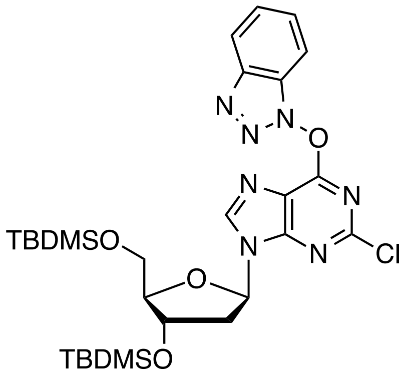

4a

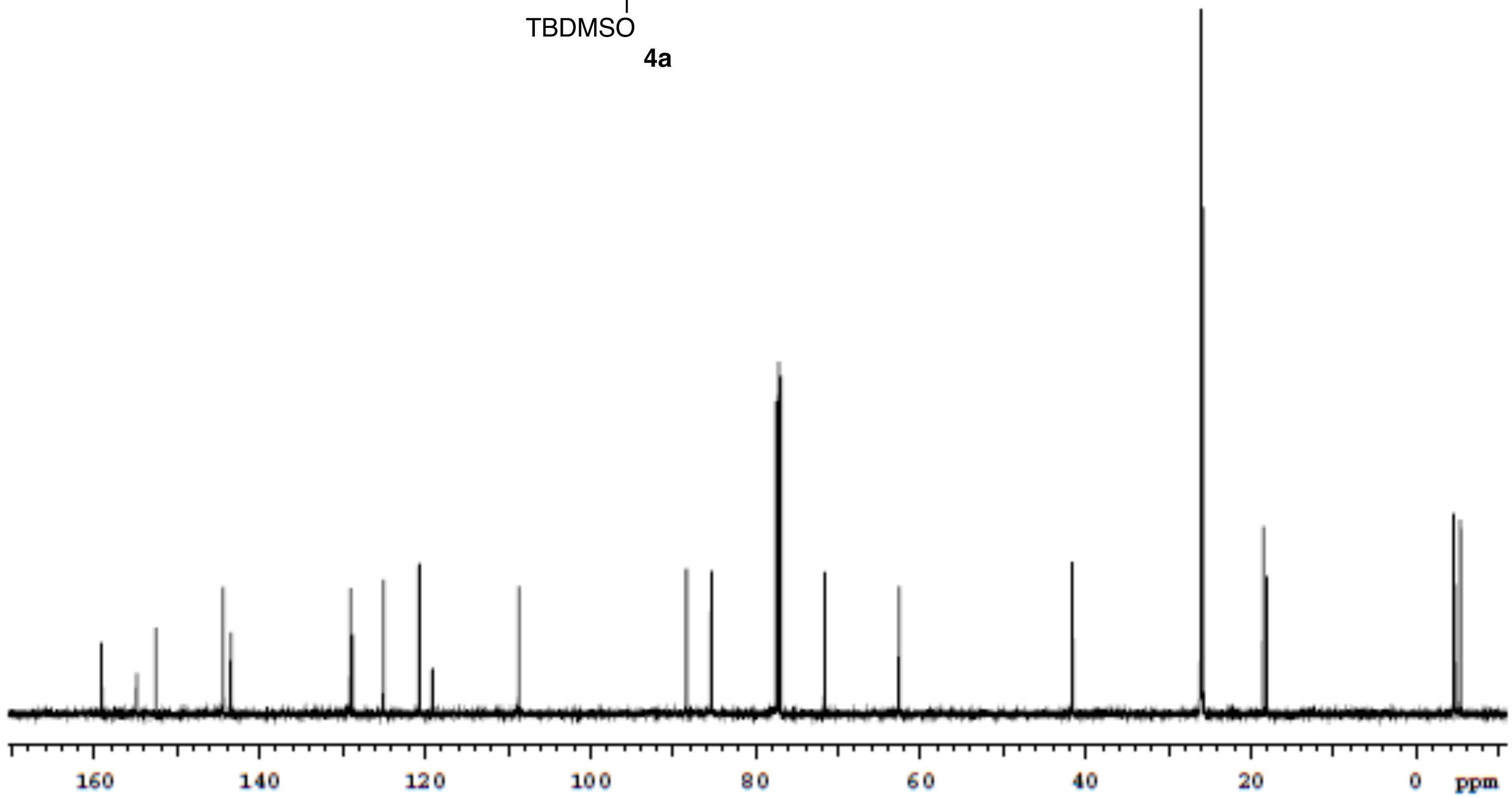

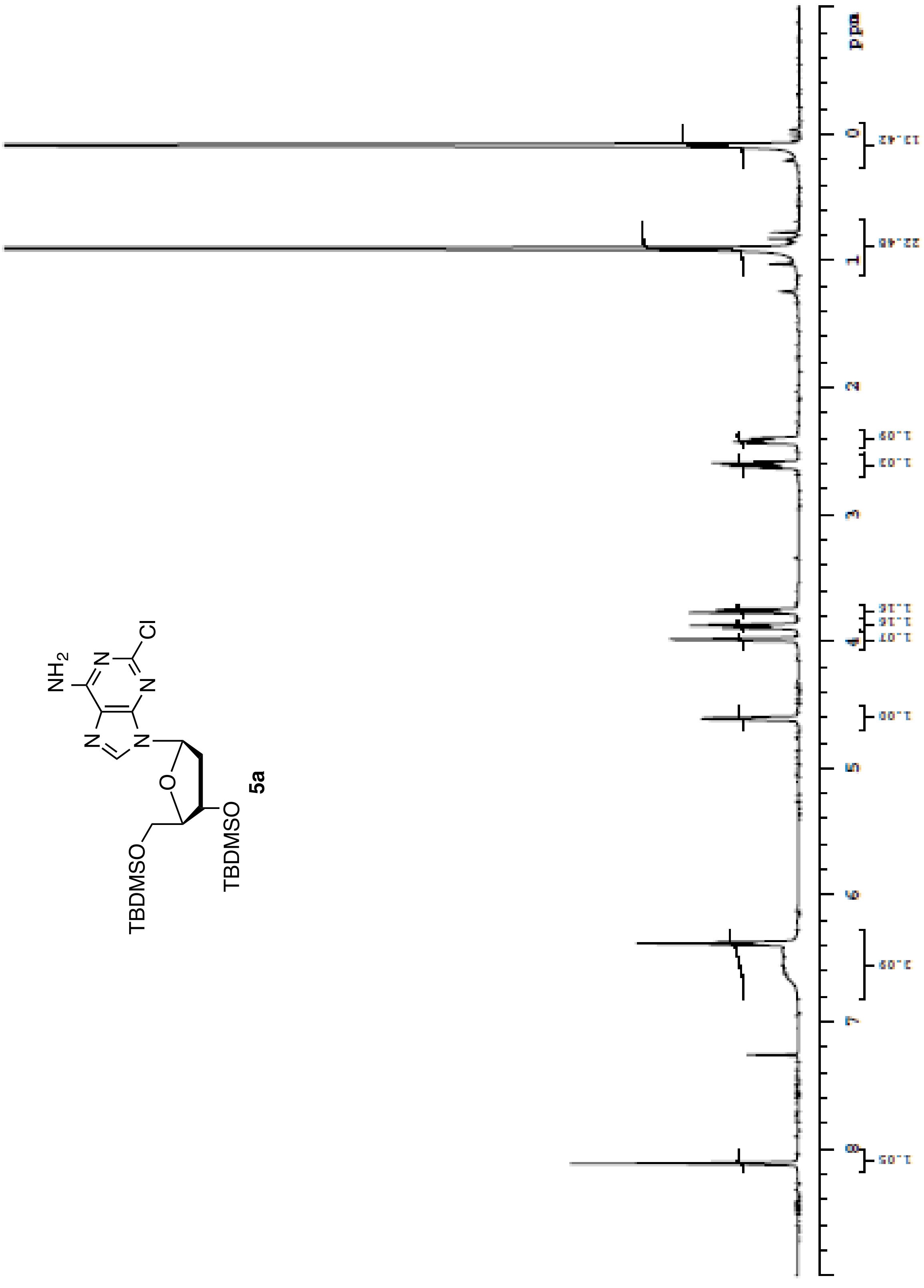
NH2
N
N
Cl
N
N
O
TBDMSO
TBDMSO
5a
ppm

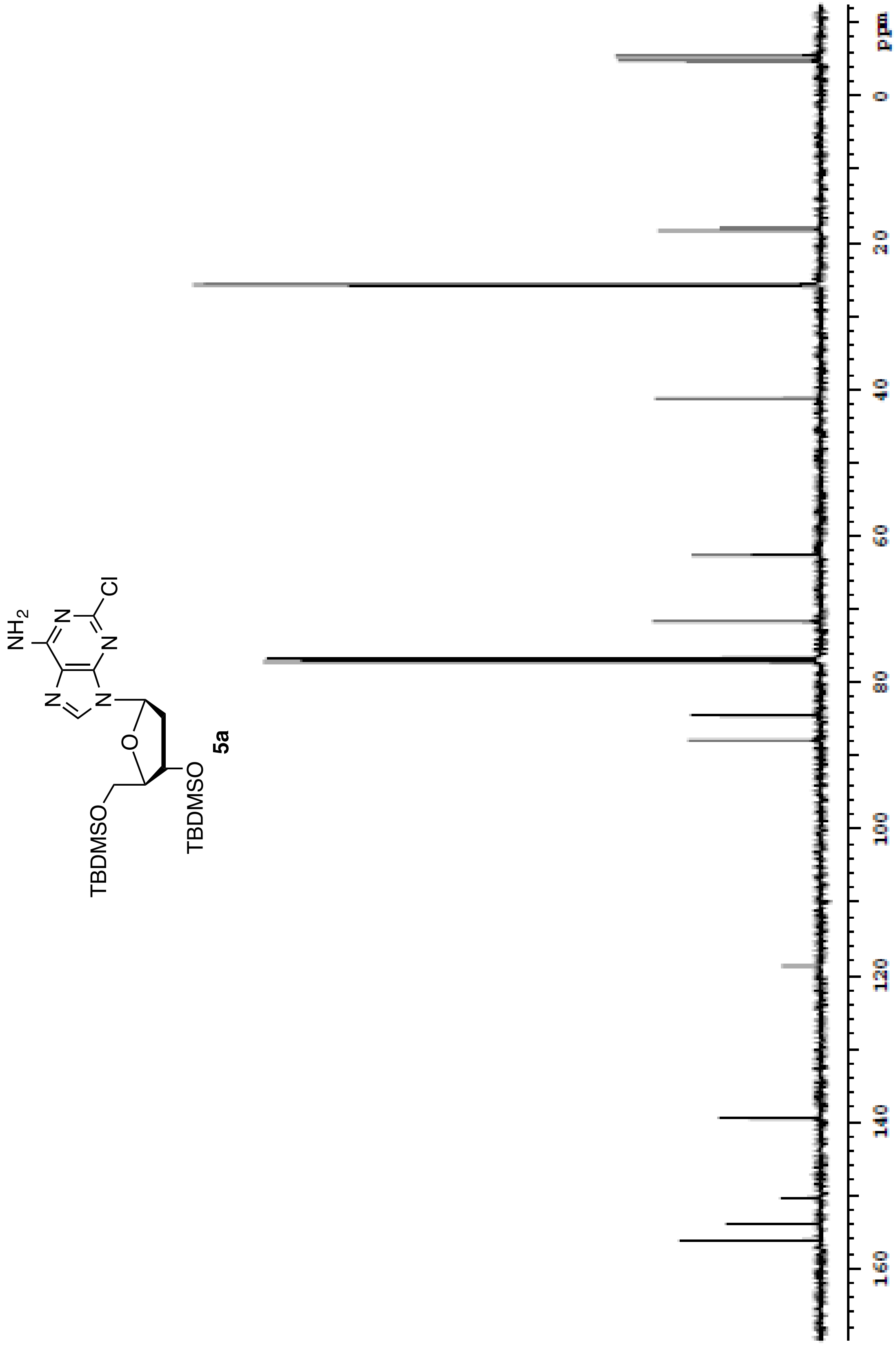
NH2
N
N
N
N
Cl
O
TBDMSO
TBDMSO
5a
ppm
0
20
40
60
80
100
120
140
160

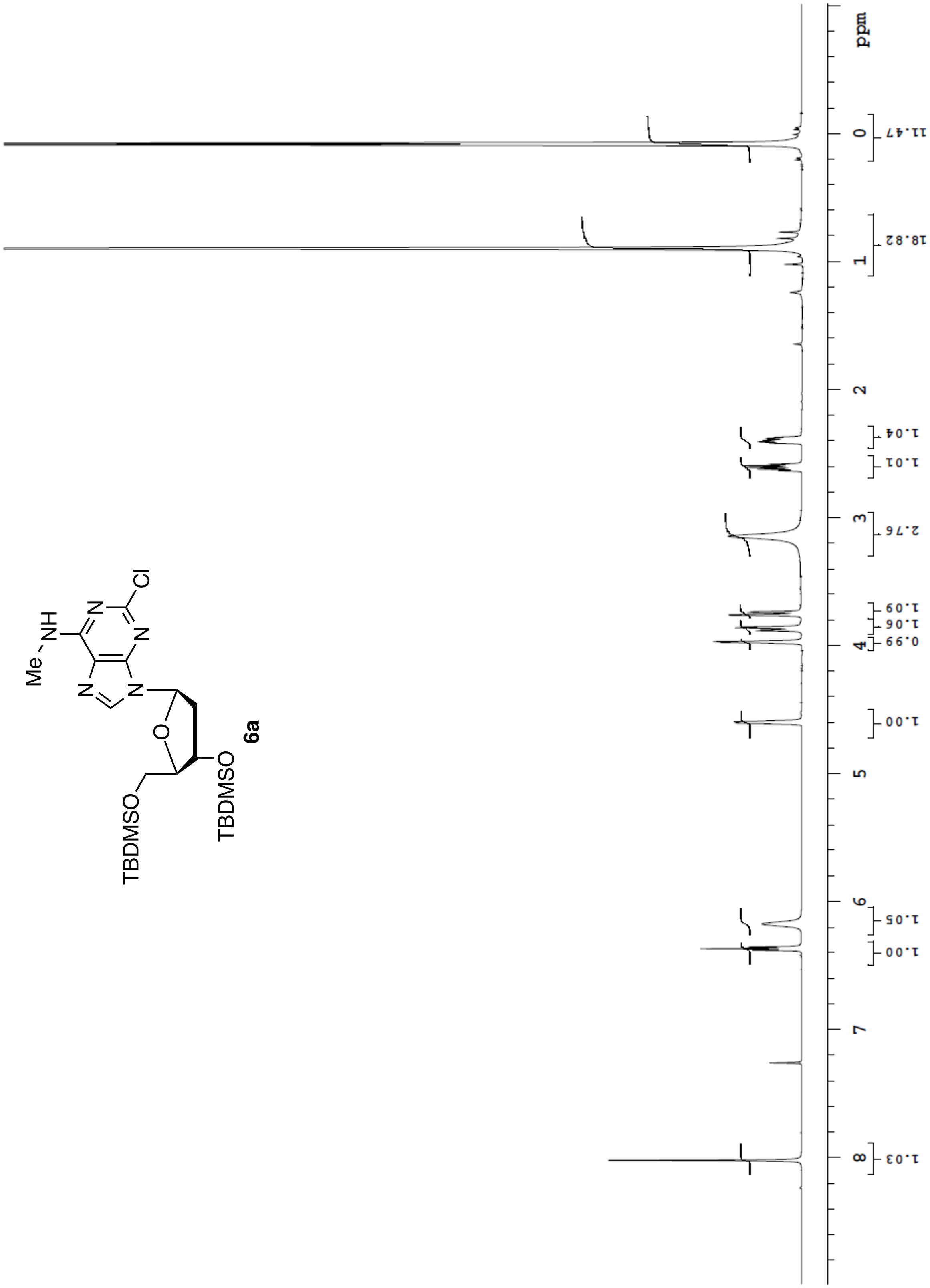
Me
NH
N
Cl
O
TBDMSO
TBDMSO
6a
ppm
0
1
2
3
4
5
6
7
8
11.47
18.82
1.04
1.01
2.76
1.09
1.06
0.99
1.00
1.05
1.00
1.03

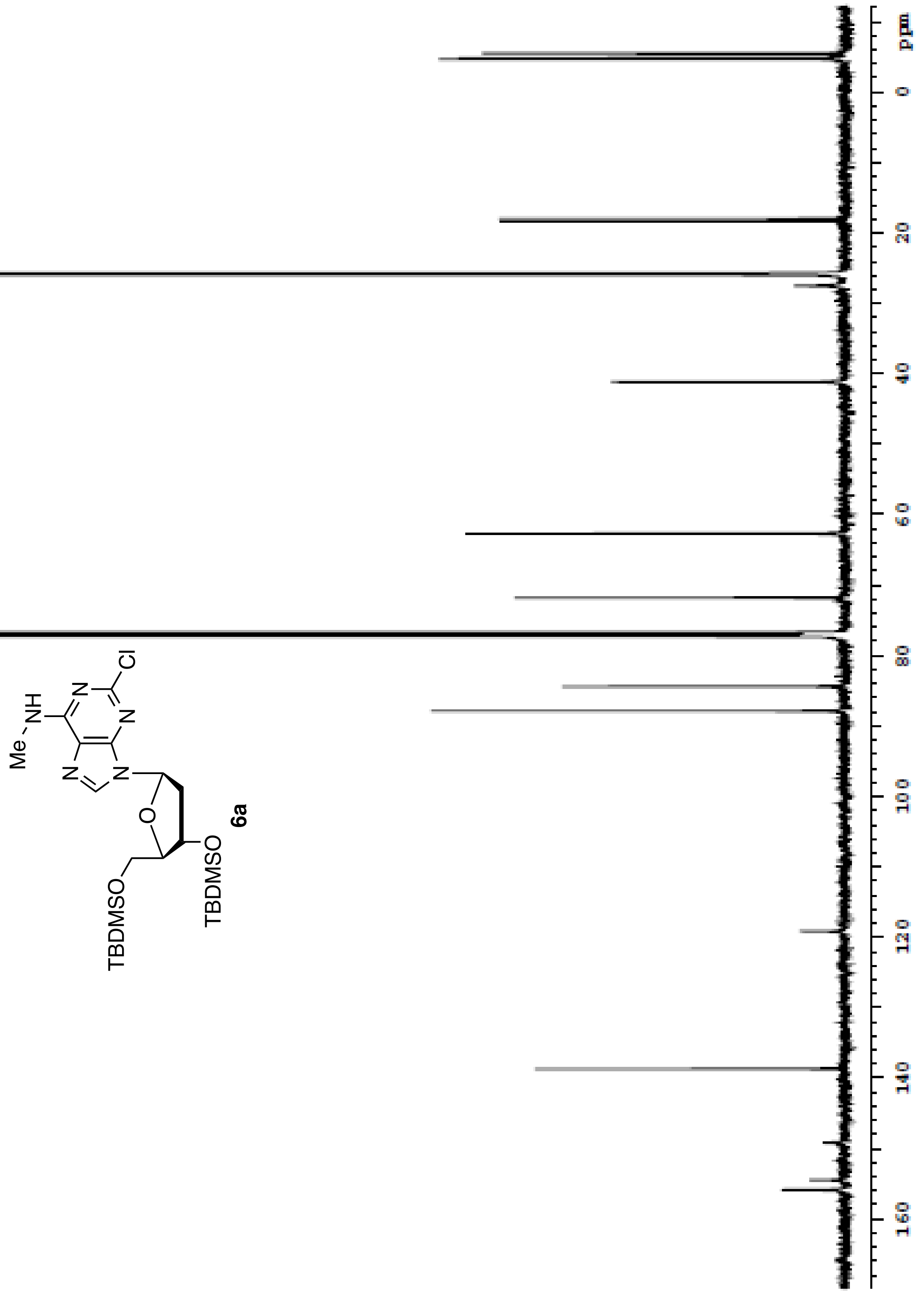

6a

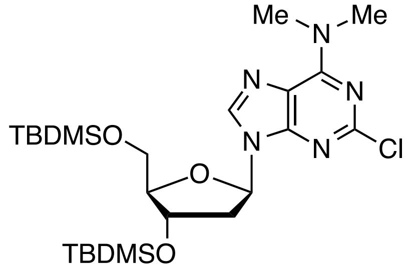
Me
N
Me
N
N
N
N
Cl
TBDMSO
O
TBDMSO

7a

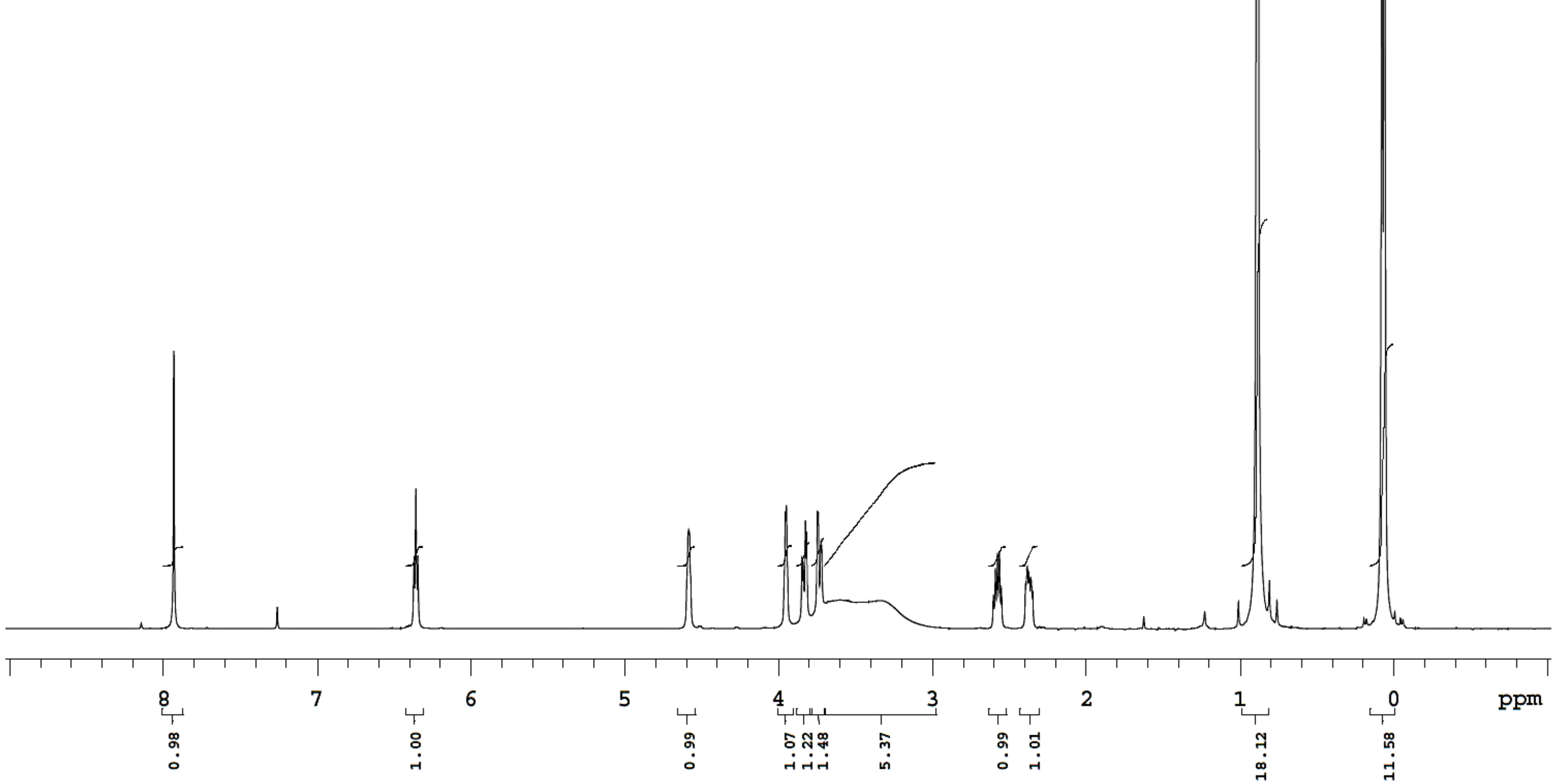
8
7
6
5
4
3
2
1
0
ppm
0.98
1.00
0.99
1.07
1.22
1.48
5.37
0.99
1.01
18.12
11.58

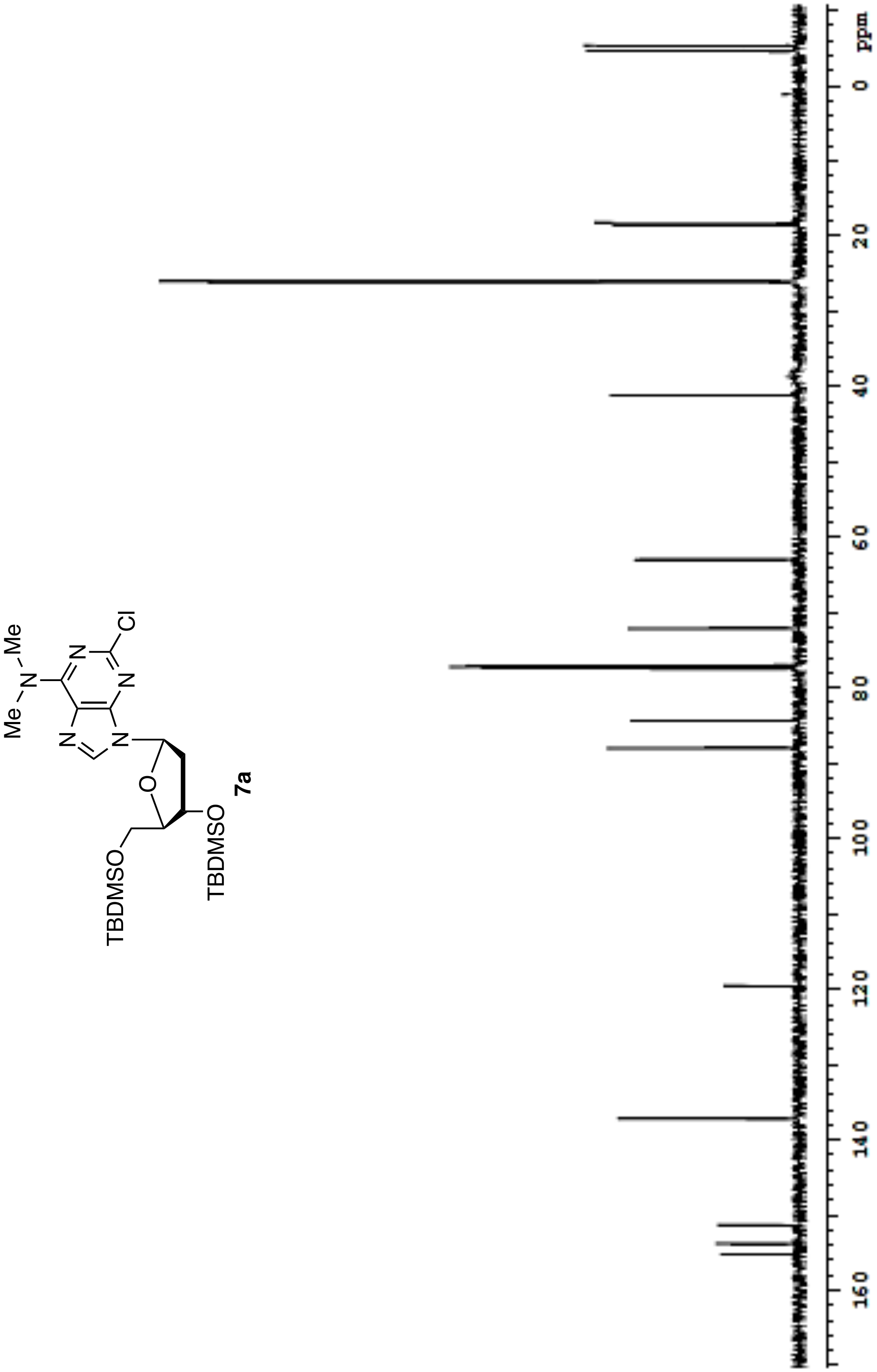
Me
N
Me
N
N
Cl
N
N
TBDMSO
O
TBDMSO
7a
ppm
0
20
40
60
80
100
120
140
160

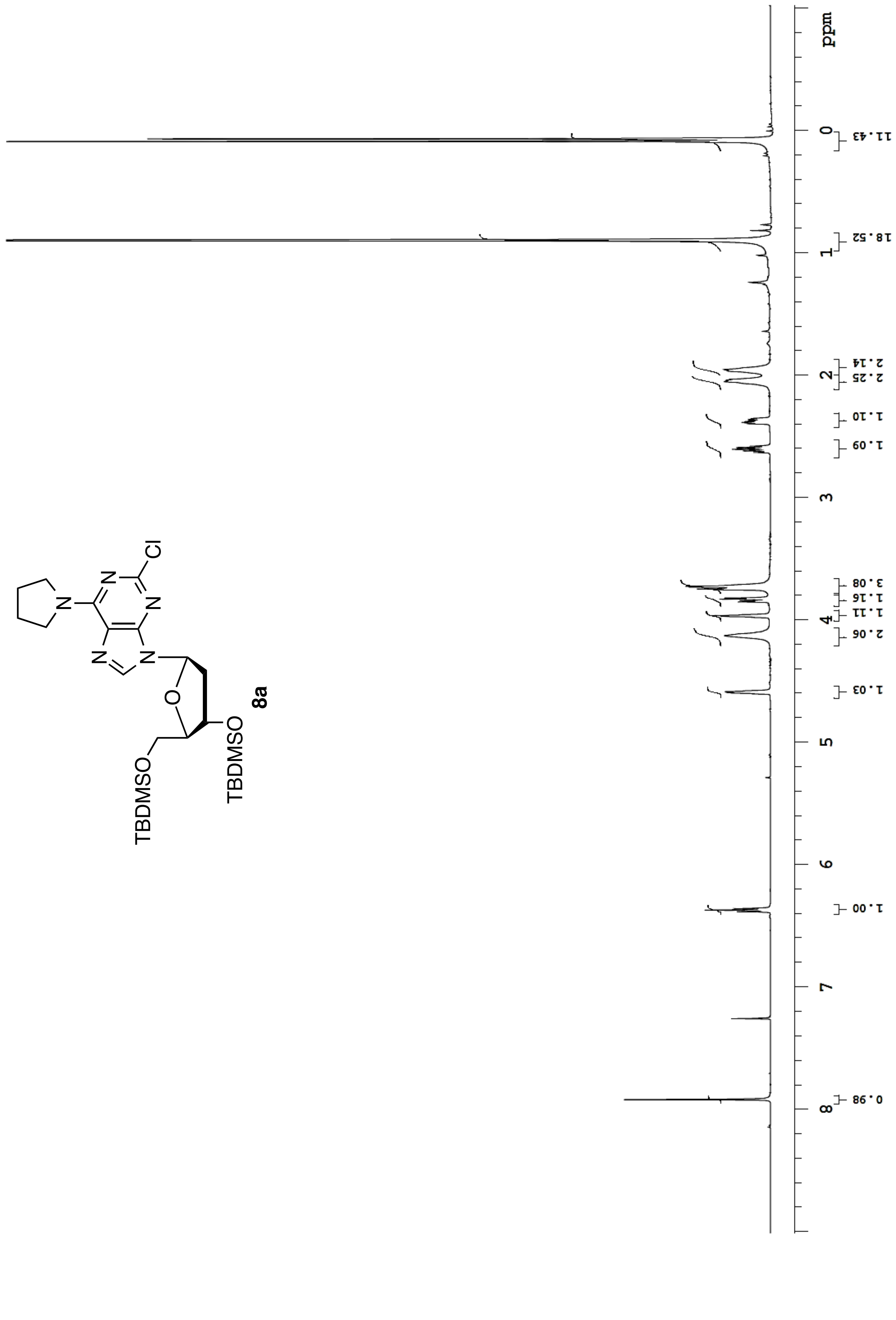
TBDMSO
TBDMSO
8a
Cl
N
O
ppm
0
1
2
3
4
5
6
7
8
11.43
18.52
2.14
2.25
1.10
1.09
3.08
1.16
1.11
2.06
1.03
1.00
0.98

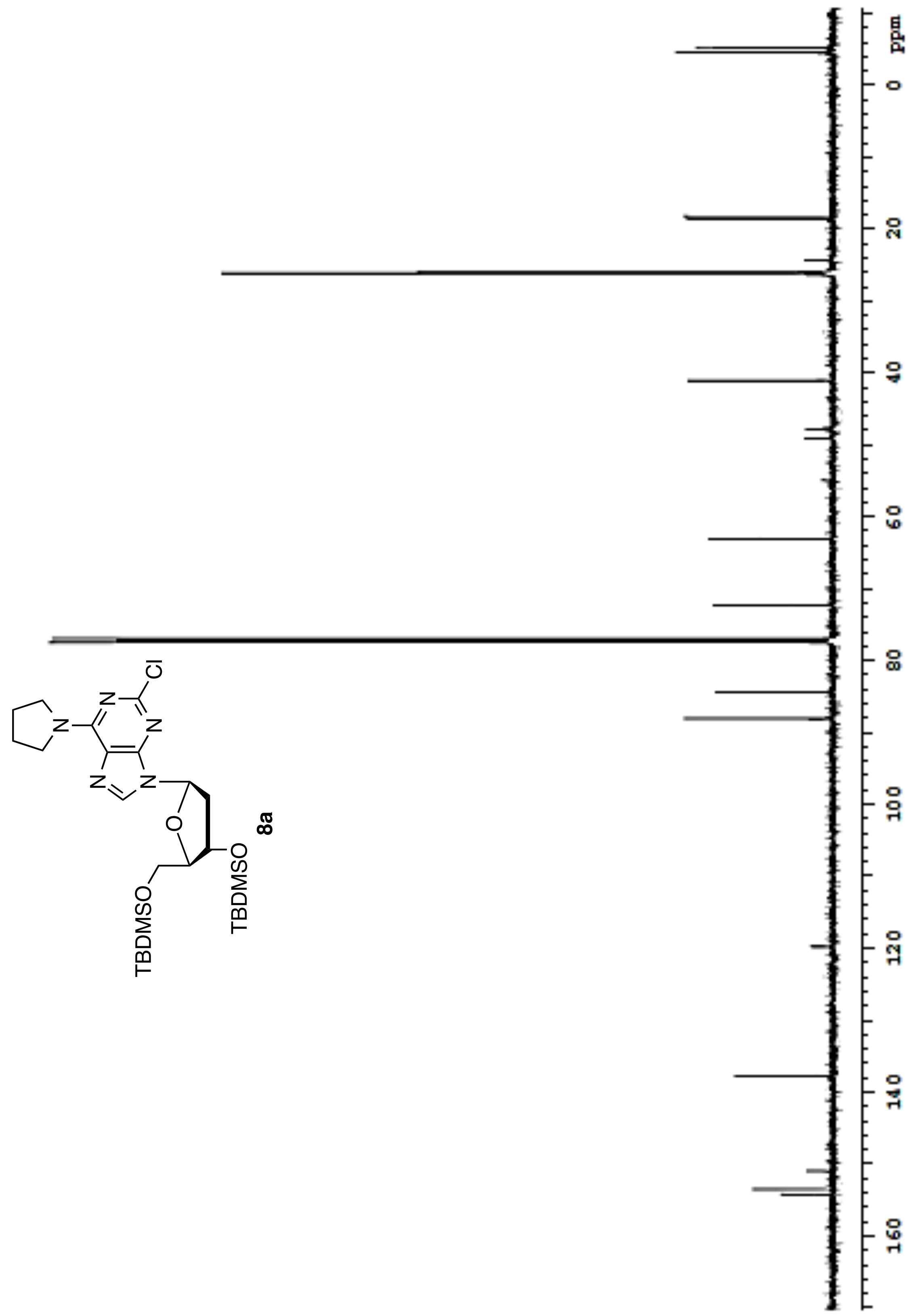
TBDMSO
TBDMSO
N
N
N
N
N
Cl
O
8a
ppm
0
20
40
60
80
100
120
140
160

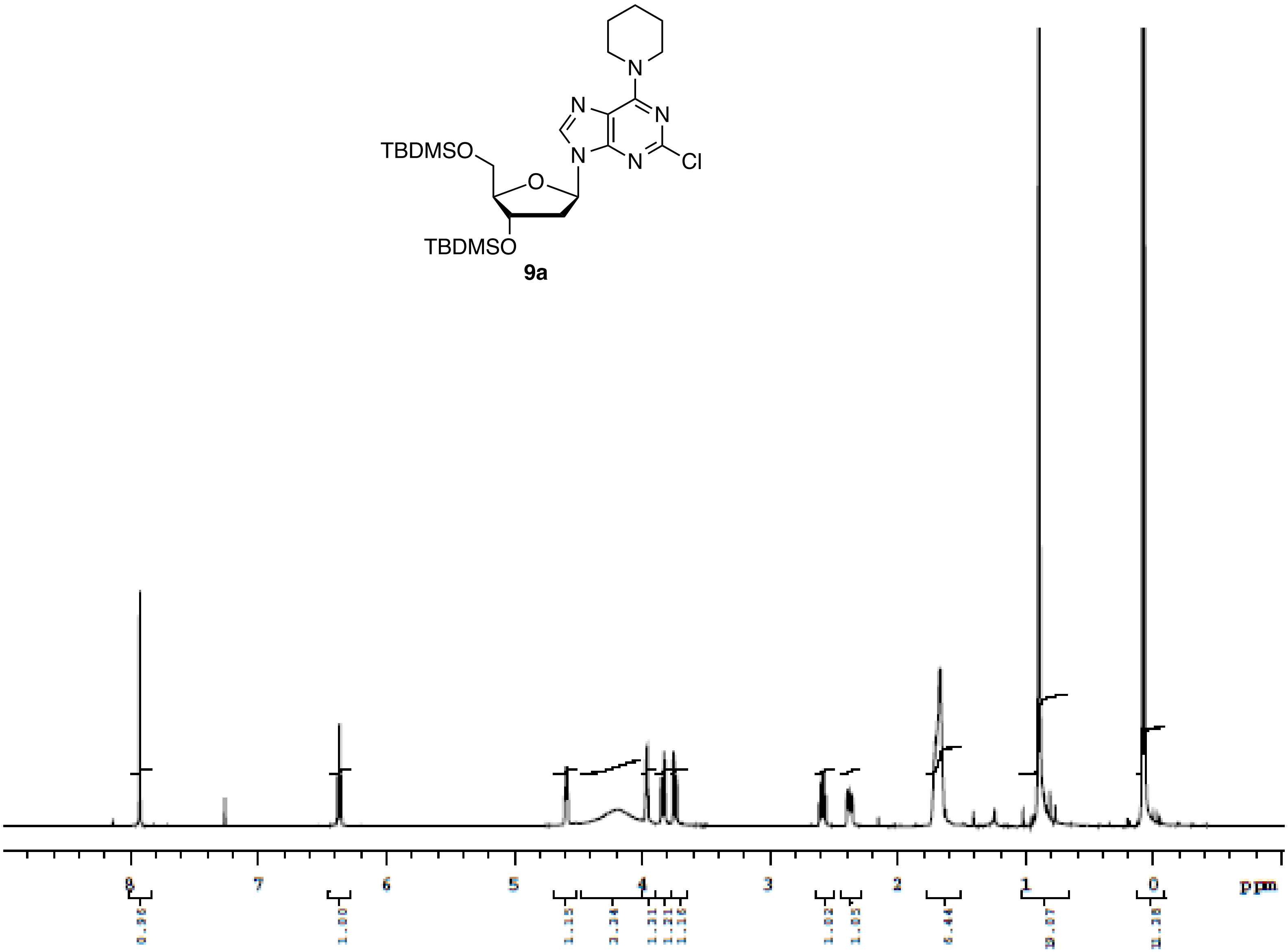
TBDMSO
TBDMSO
N
N
N
N
N
O
Cl
9a
8
7
6
5
4
3
2
1
0
ppm

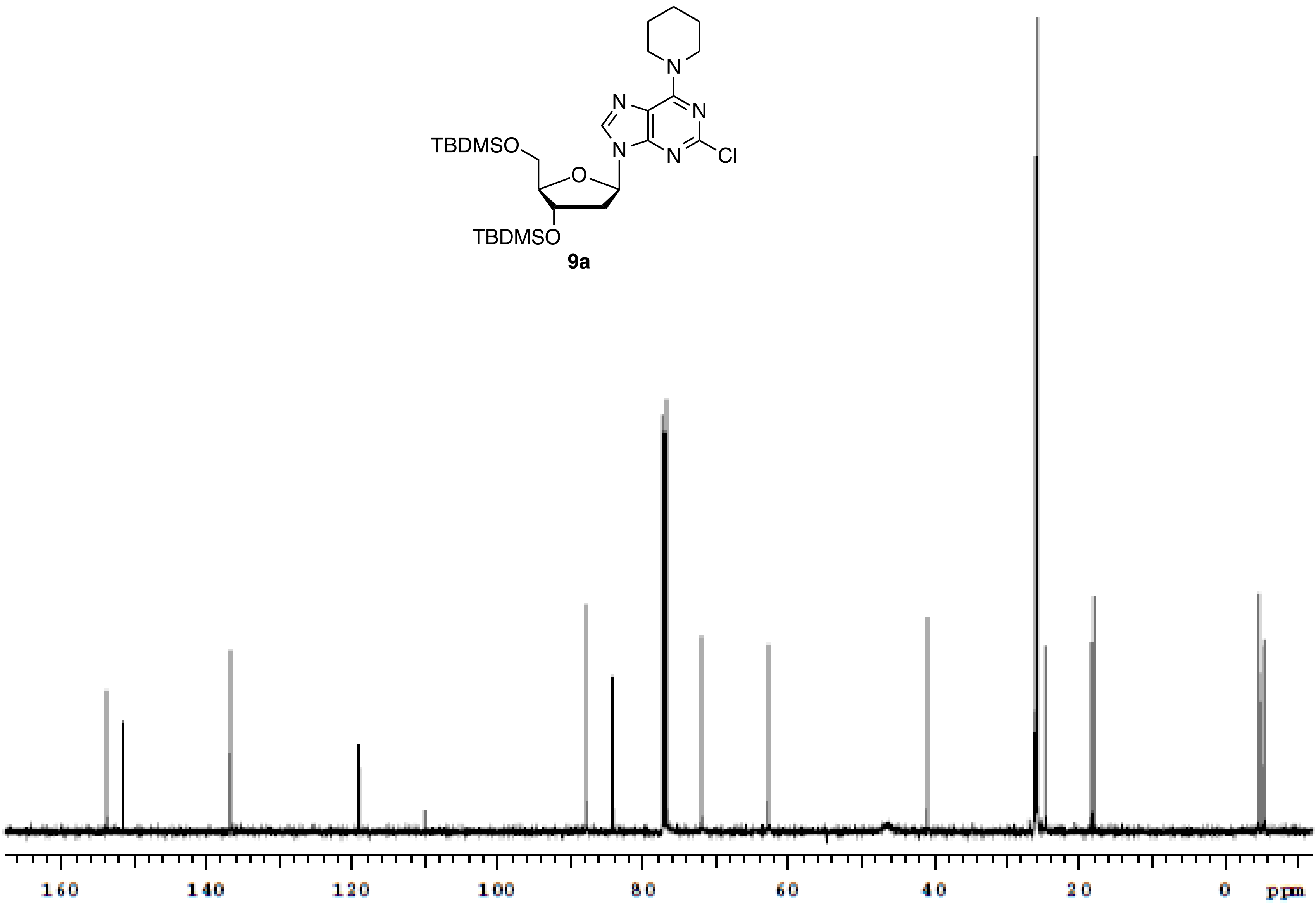

9a

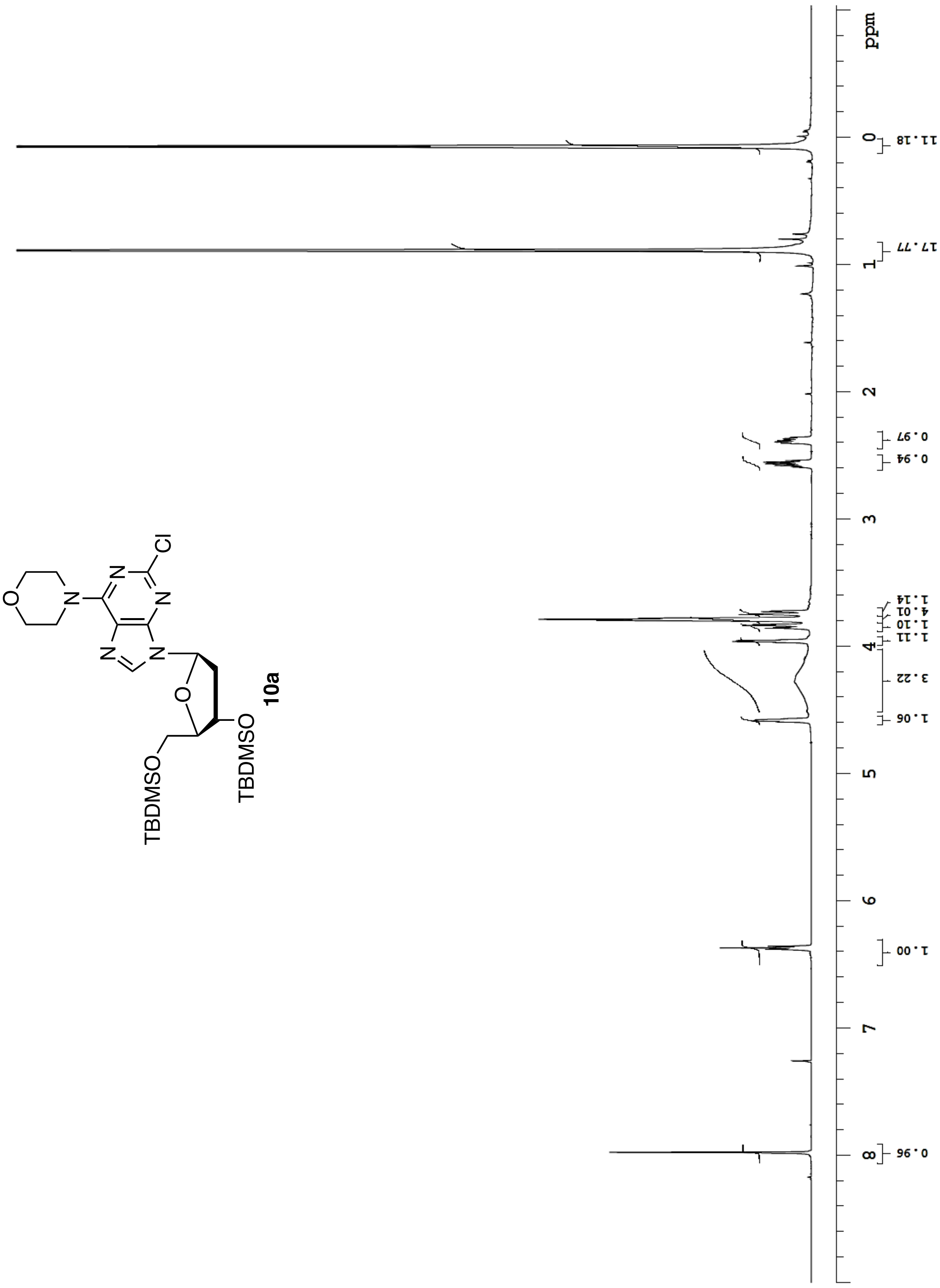

10a
TBDMSO
TBDMSO
Cl
ppm
11.18
17.77
0.97
0.94
1.14
4.01
1.10
1.11
3.22
1.06
1.00
0.96

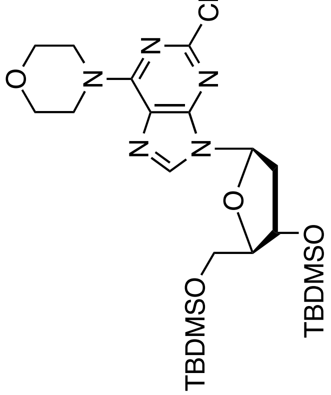

10a

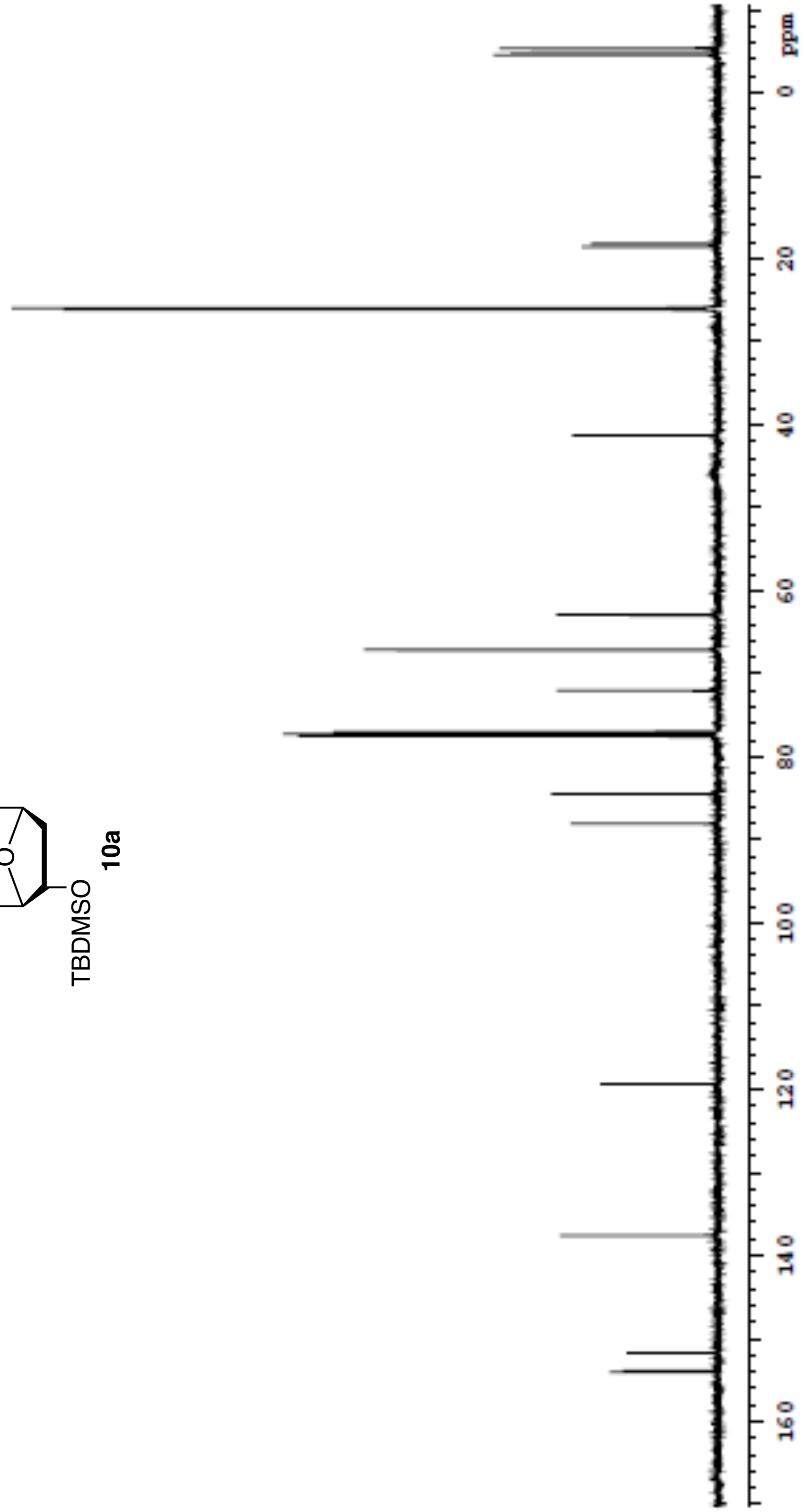

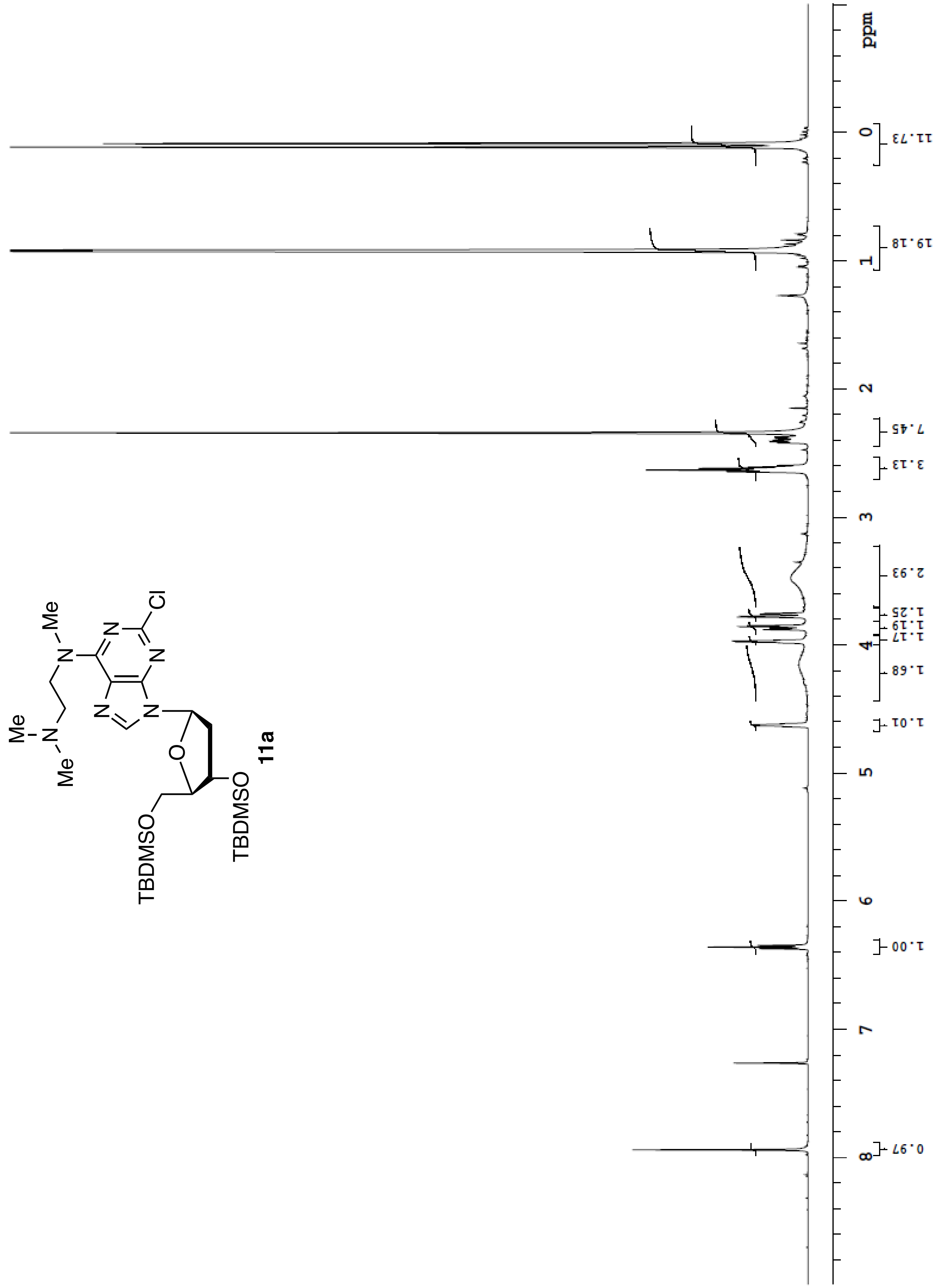

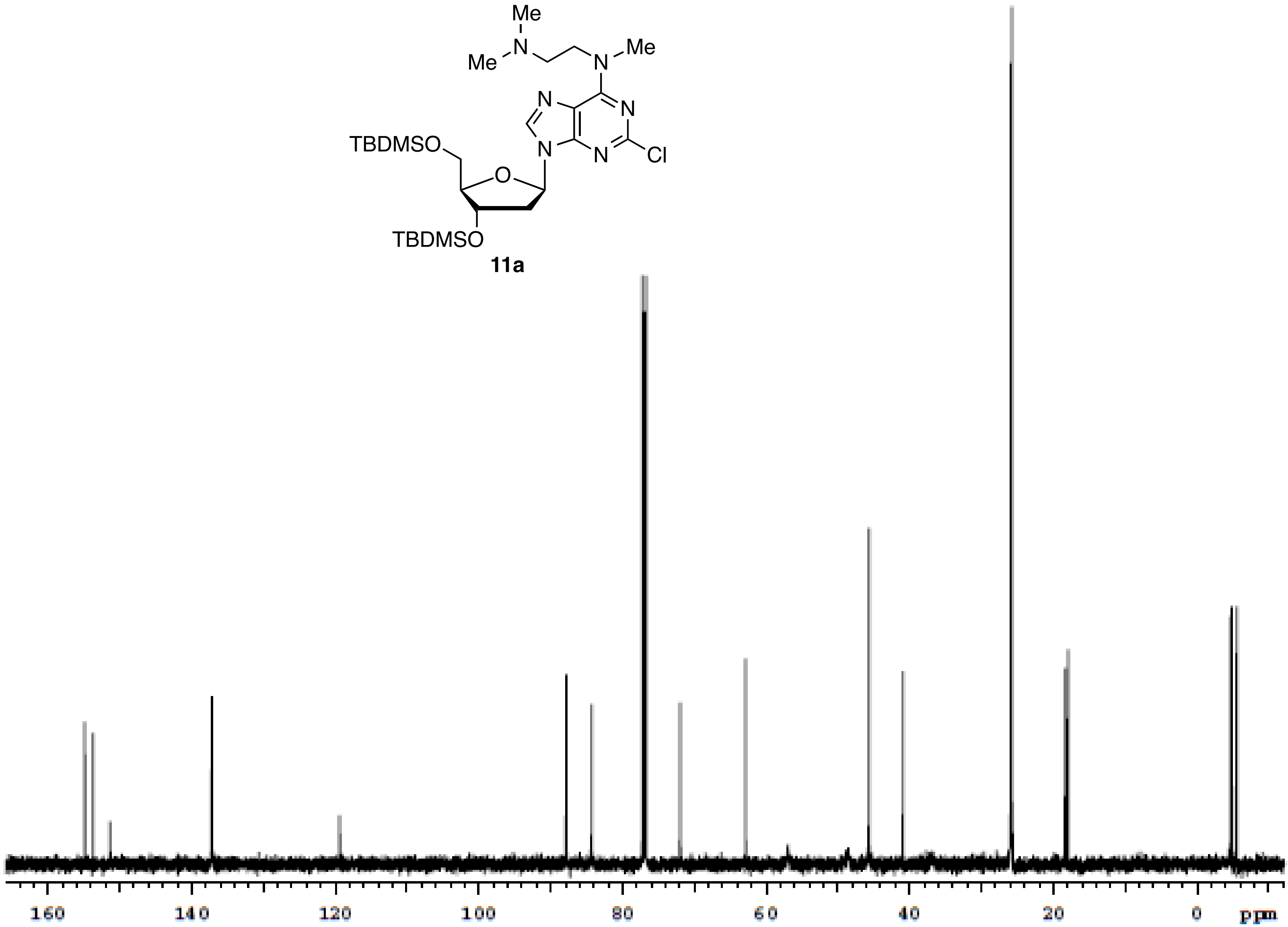
Me
Me
N
N
Me
N
N
N
N
TBDMSO
O
Cl
TBDMSO
11a
160
140
120
100
80
60
40
20
0
ppm

Sample Name :
GVK-DD0397-CHLORO

Solvent: cdcl3
Date: Mar 22 2015
400 VNMRS/NRM-1/NMR-002

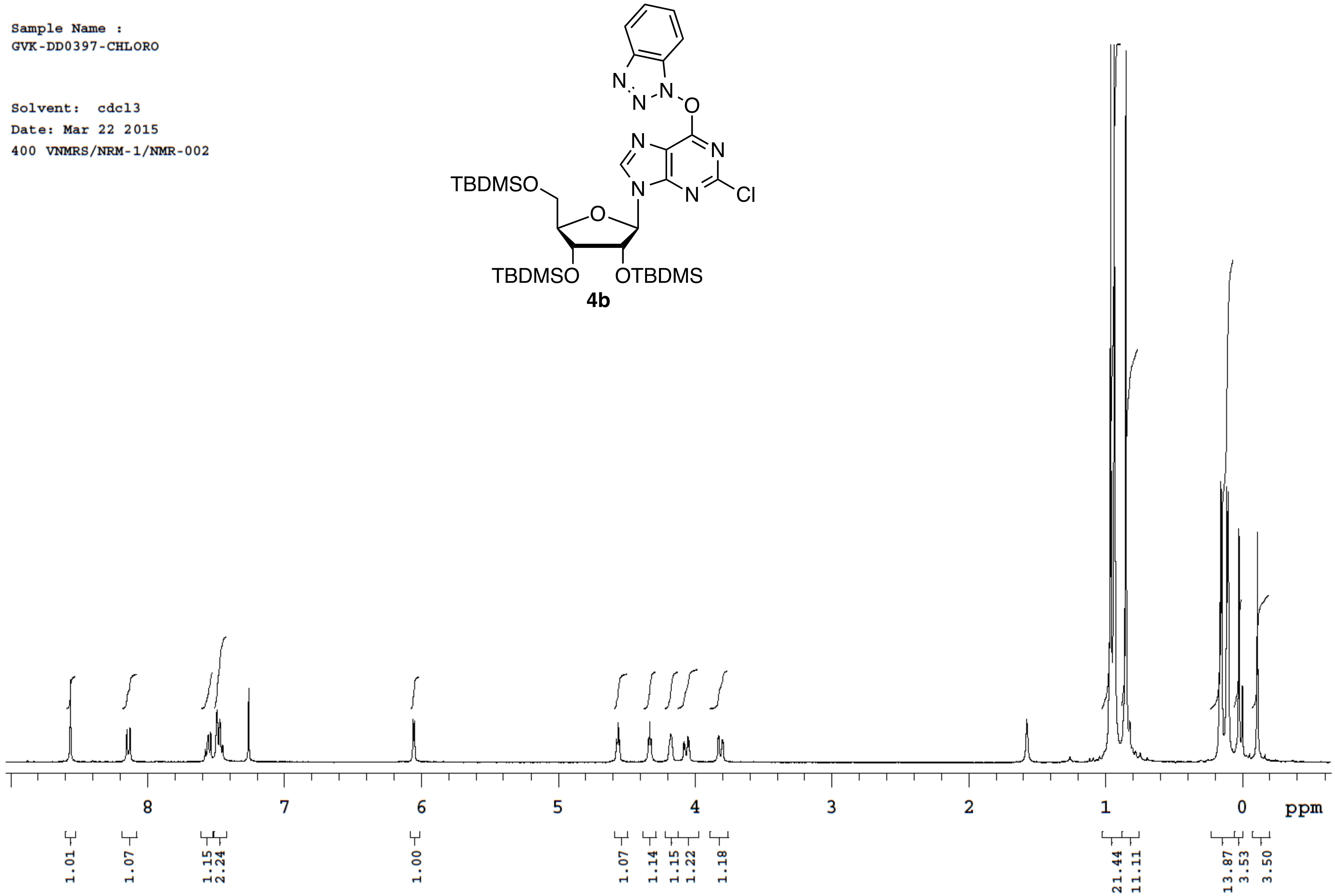

4b

Plotname: 021503C284_PROTON_01_plot02

Sample Name :
GVK-DD0397-CHLORO

Solvent: cdcl3
Date: Mar 22 2015
400 VNMRS/NRM-1/NMR-002

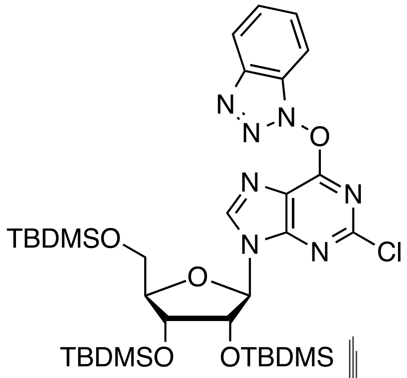

4b

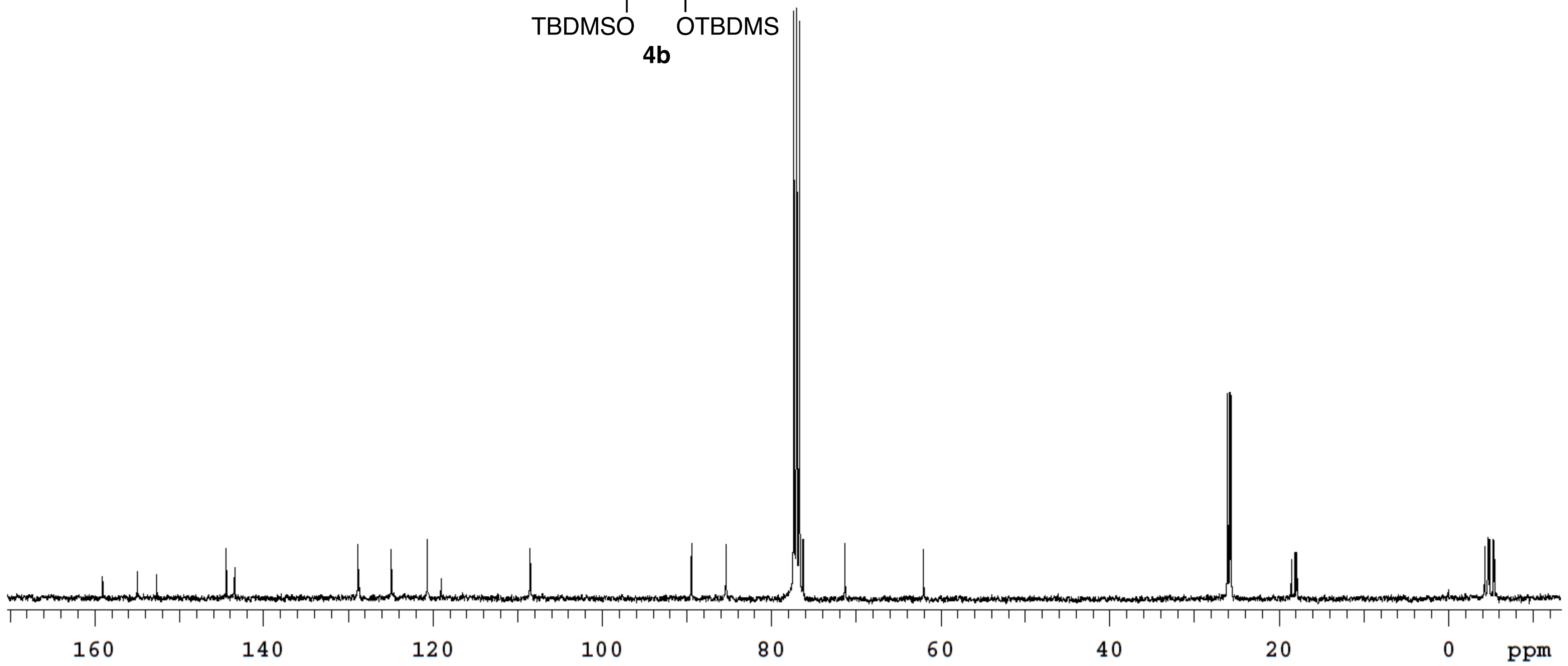

Plotname: 021503C2184_CARBON_01_plot01

Sample Name :
GVK-DD0397-E109832-A
Solvent: cdcl3
400-MR/NRM-1/NMR-001
Date: Feb 27 2015

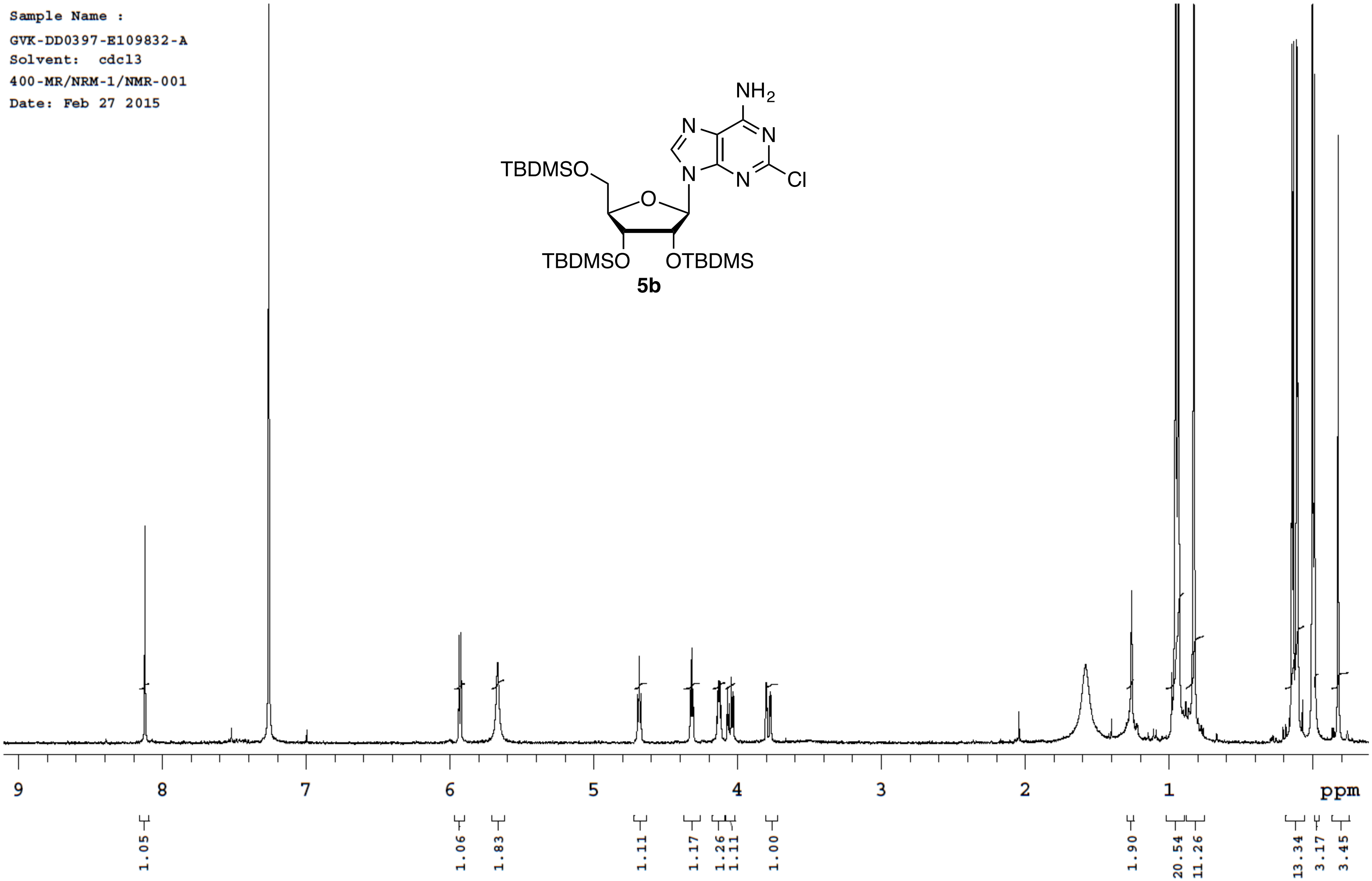

Plotname: 021502D3602_PROTON_01_plot08

Sample Name :
GVK-DD0-397-AMM-T

Solvent: cdcl3
Date: Mar 28 2015
400 VNMRS/NRM-1/NMR-002

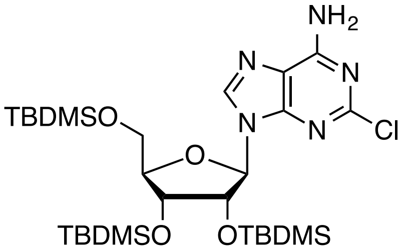

5b

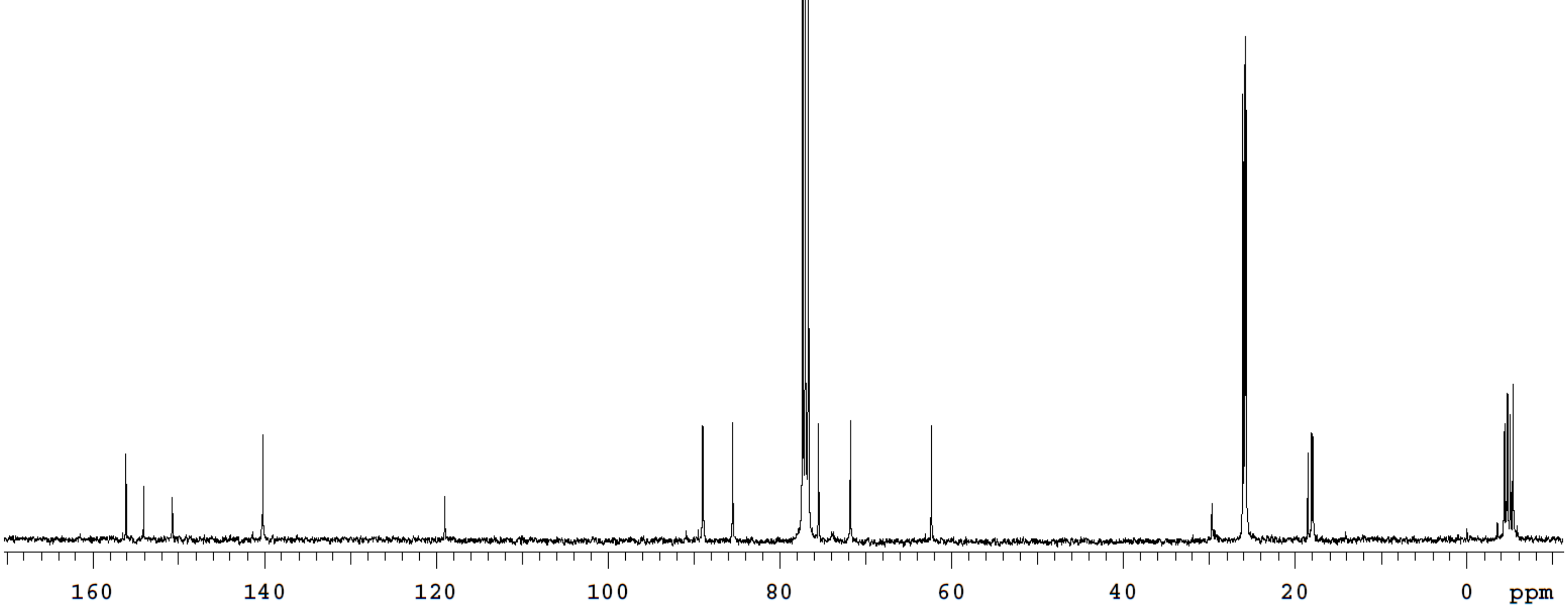

Plotname: 021503D2572_CARBON_01_plot01

Sample Name :
GVK-DD0397-ME-T
Solvent: cdcl3
400-MR/NRM-1/NMR-001
Date: Mar 29 2015

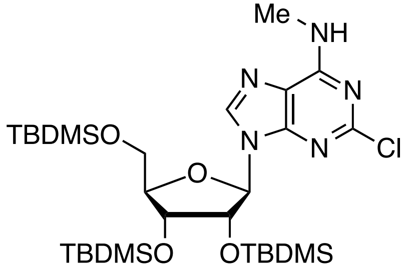

6b

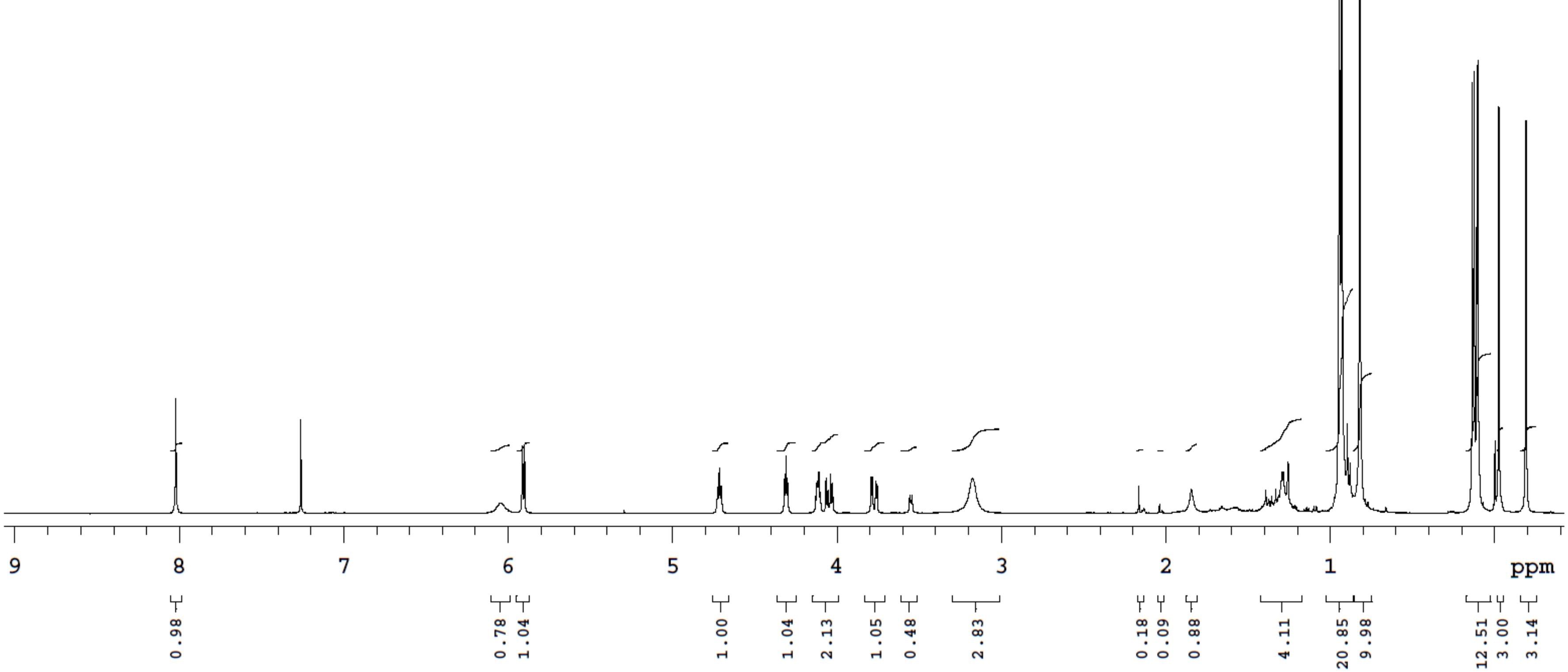

Plotname: 021503D2577_PROTON_01_plot07

Sample Name :
GVK-DD0397-ME-T
Solvent: cdcl3
400-MR/NRM-1/NMR-001
Date: Mar 29 2015

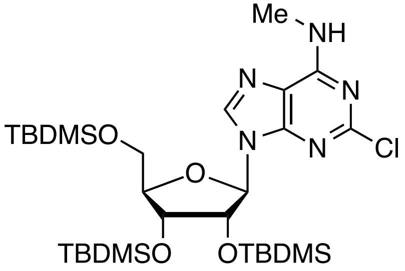

6b

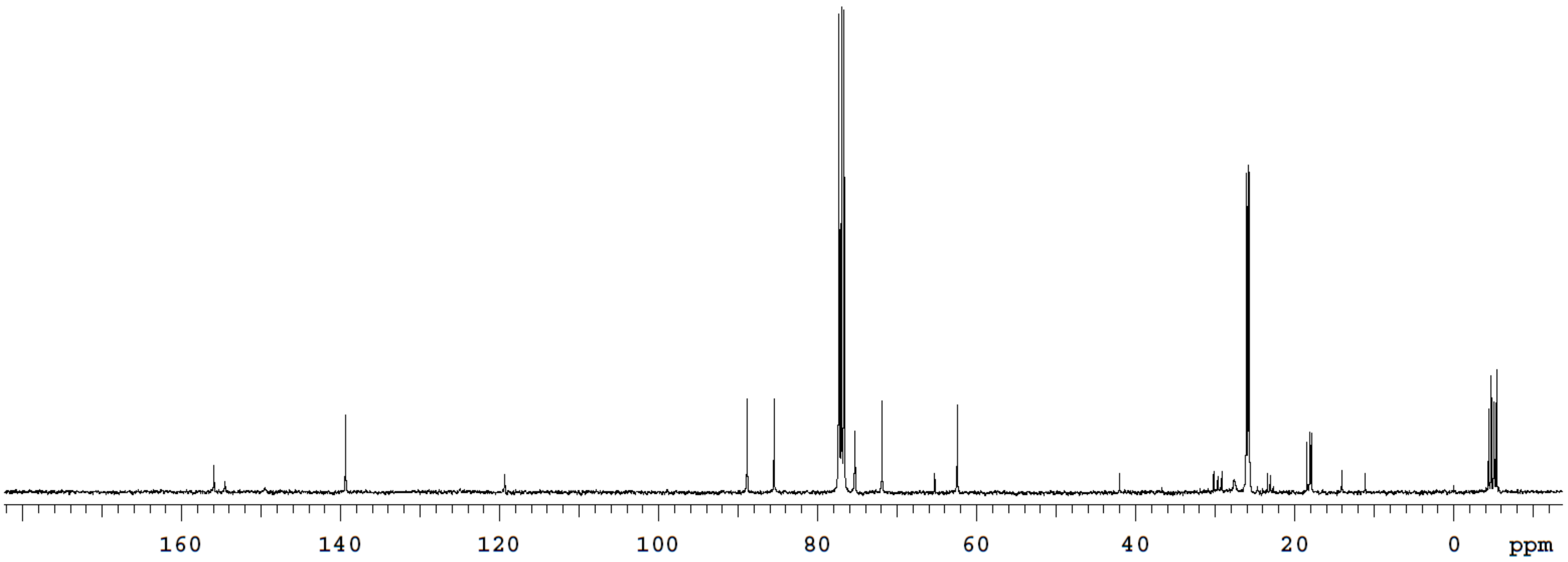

Plotname: 021503D2577_CARBON_01_plot01

Sample Name :
GVK-DD0-397-DME-T

Solvent: cdcl3
Date: Mar 28 2015
400 VNMRS/NRM-1/NMR-002

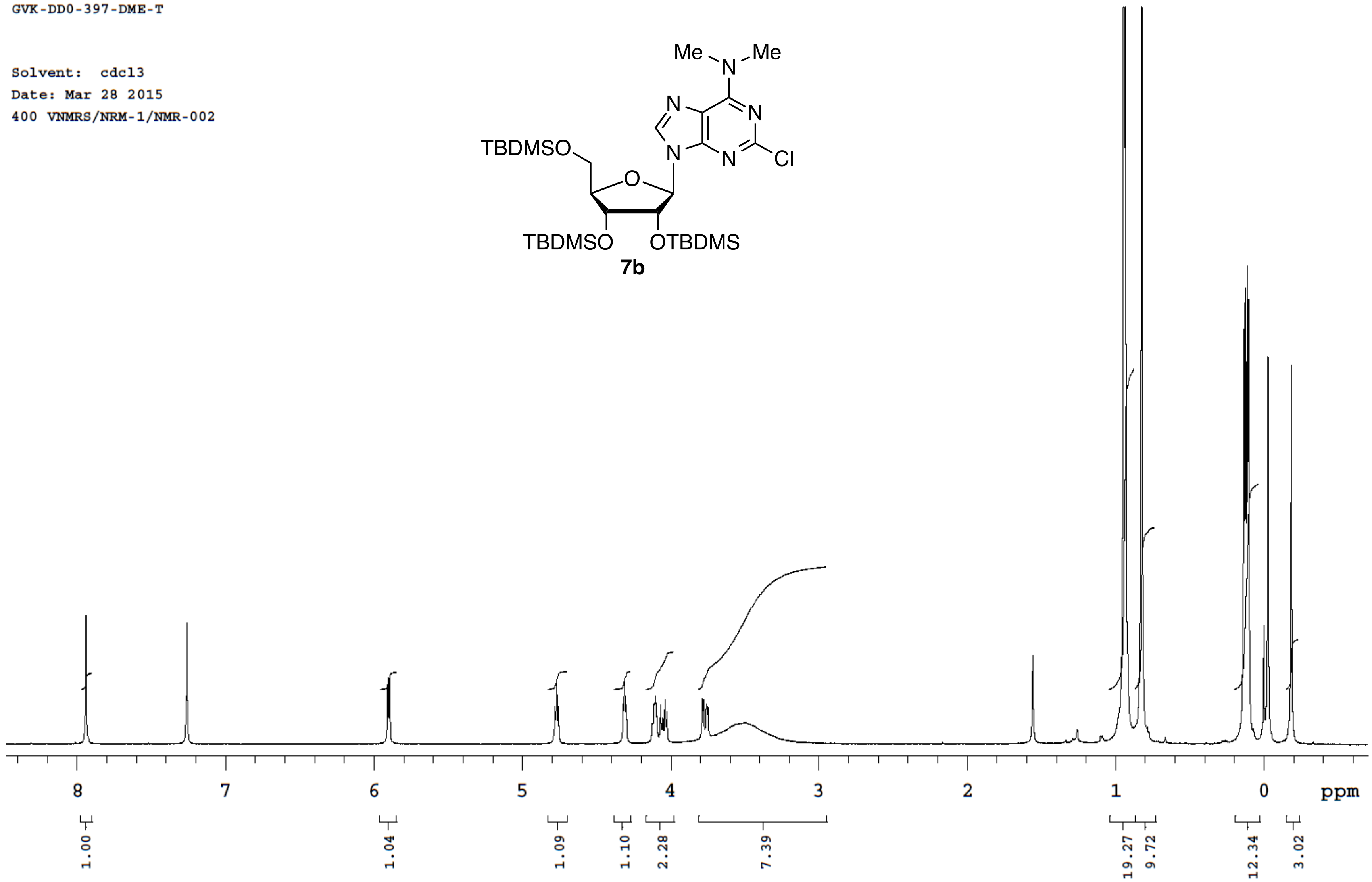

Plotname: 021503D2595_PROTON_01_plot08

Sample Name :
GVK-DD0-397-DME-T

Solvent: cdcl3
Date: Mar 28 2015
400 VNMRS/NRM-1/NMR-002

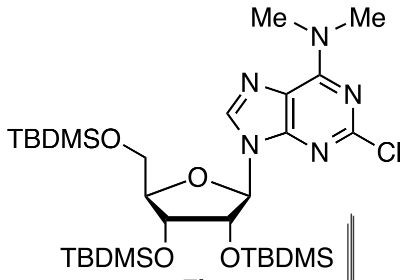

7b

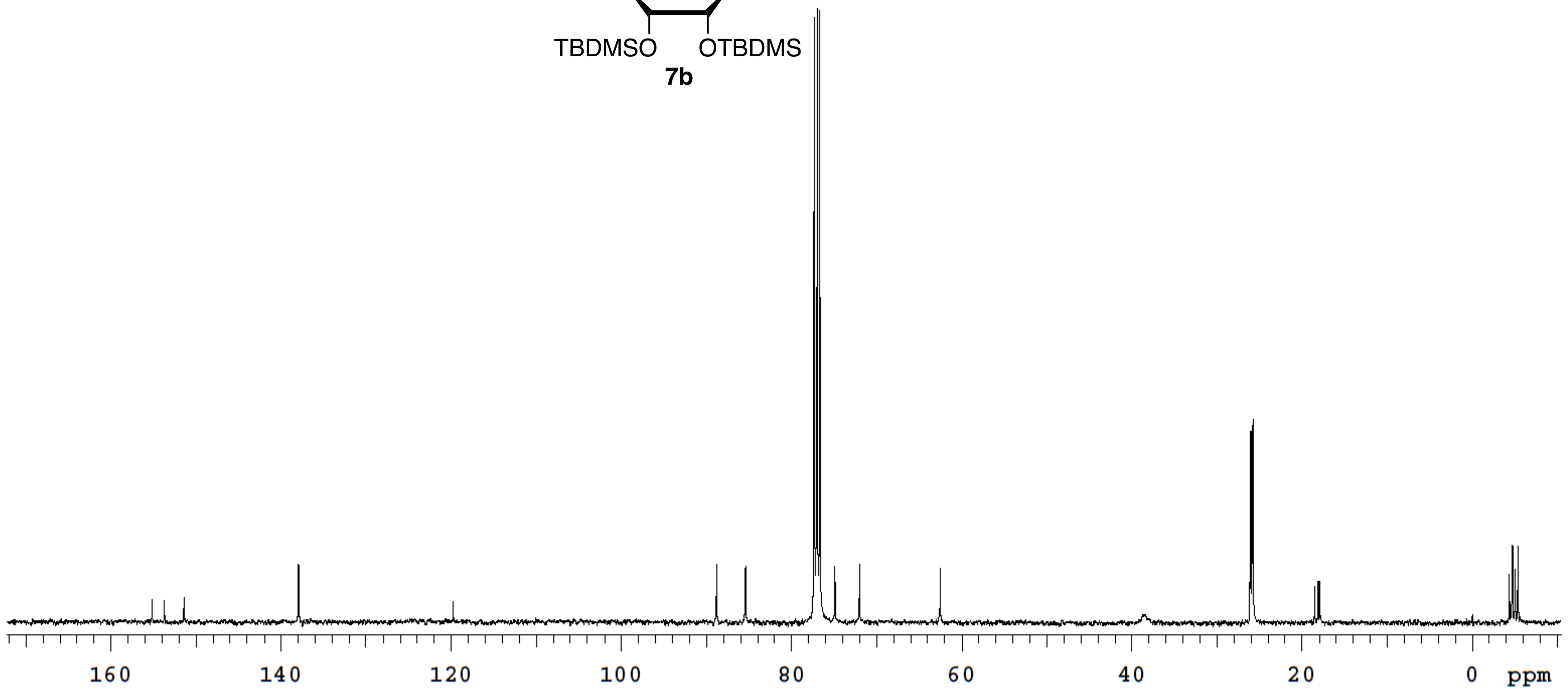

Plotname: 021503D2595_CARBON_01_plot01

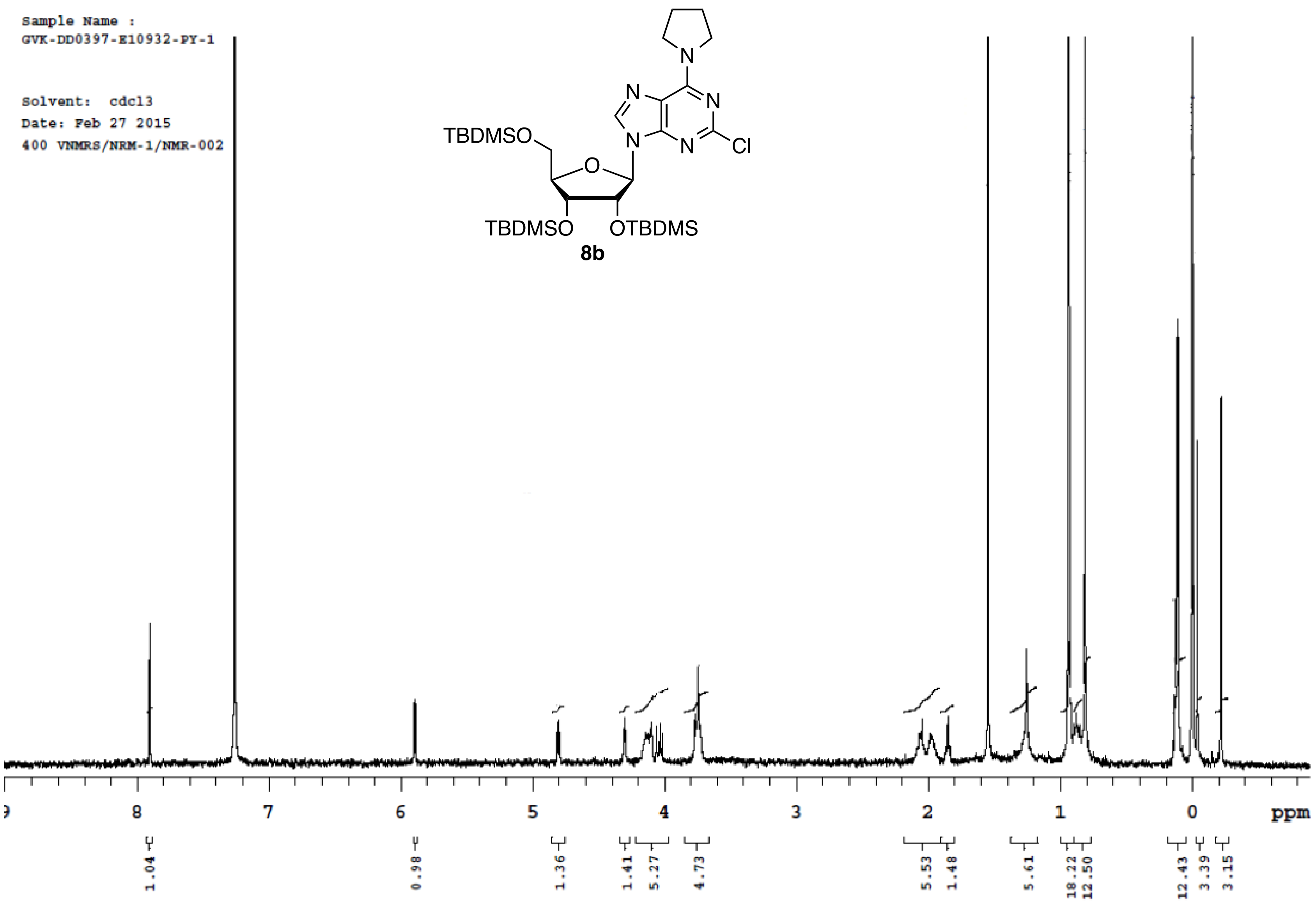

Sample Name :
GVK-DD0397-E10932-PY-1
Solvent: cdcl3
Date: Feb 27 2015
400 VNMRS/NRM-1/NMR-002
TBDMSO
TBDMSO
OTBDMS
N
Cl
8b
ppm
1.04
0.98
1.36
1.41
5.27
4.73
5.53
1.48
5.61
18.22
12.50
12.43
3.39
3.15

Sample Name :
GVK-DD0397-PYRROLIDINE
Solvent: cdcl3
400-MR/NRM-1/NMR-001
Date: Apr 25 2015

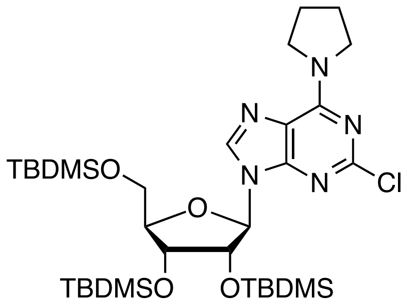

8b

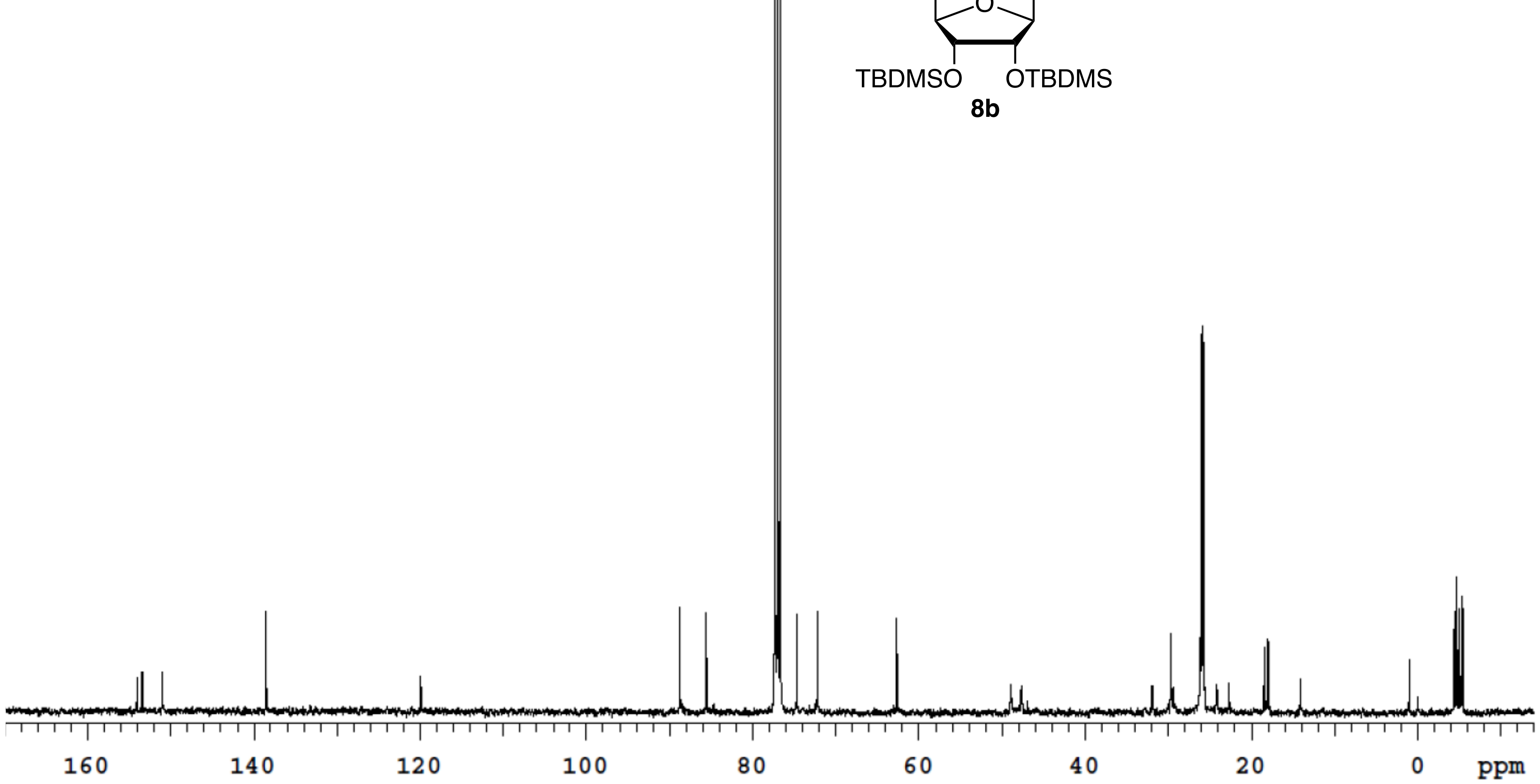

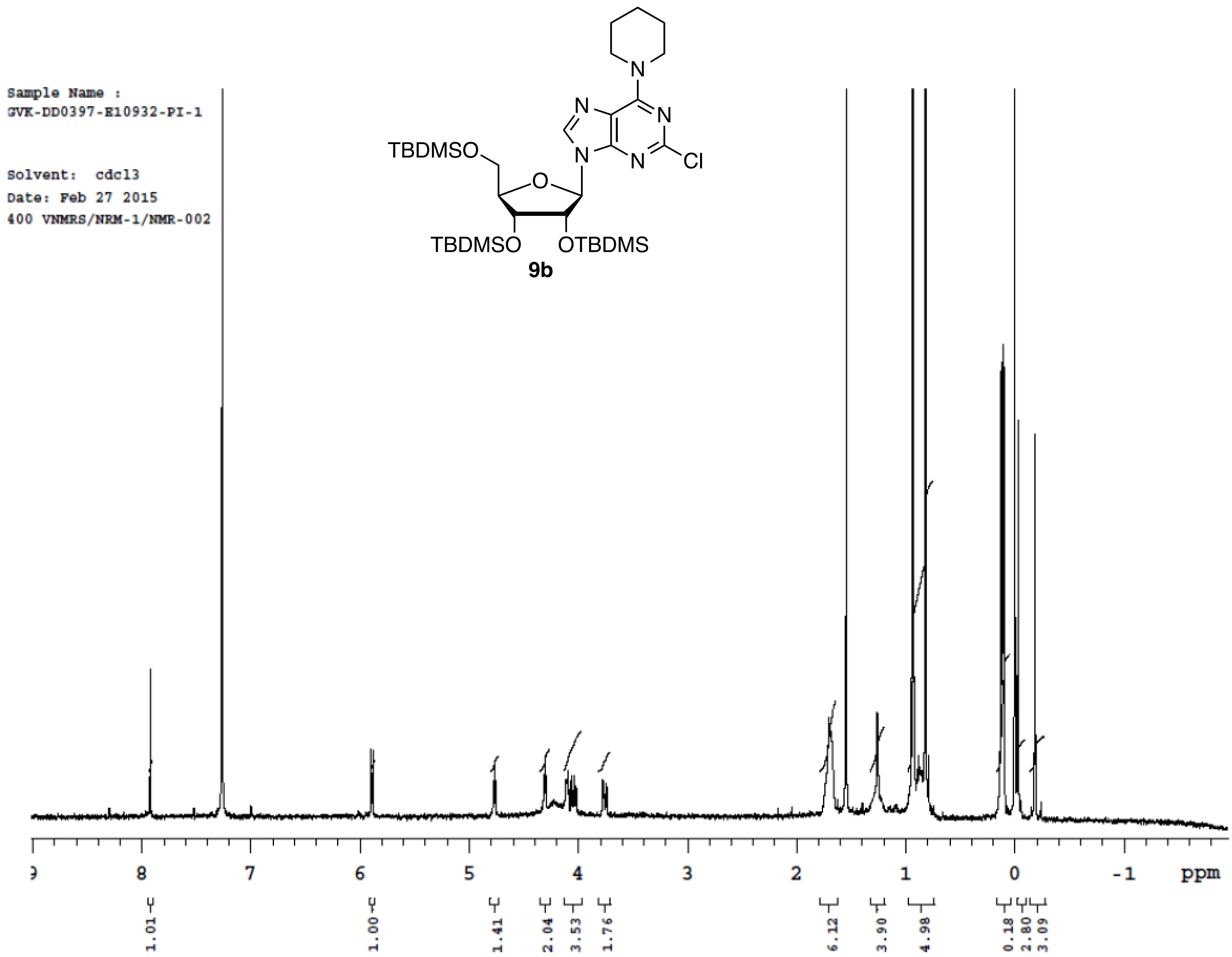
Sample Name :
GVK-DD0397-E10932-PI-1
Solvent: cdcl3
Date: Feb 27 2015
400 VNMRS/NRM-1/NMR-002
TBDMSO
TBDMSO
OTBDMS
9b
N
Cl
ppm
1.01
1.00
1.41
2.04
3.53
1.76
6.12
3.90
4.98
0.18
2.80
3.09

Sample Name :
GVK-DD0-397-PIP-T

Solvent: cdcl3
Date: Mar 29 2015
400 VNMRS/NRM-1/NMR-002

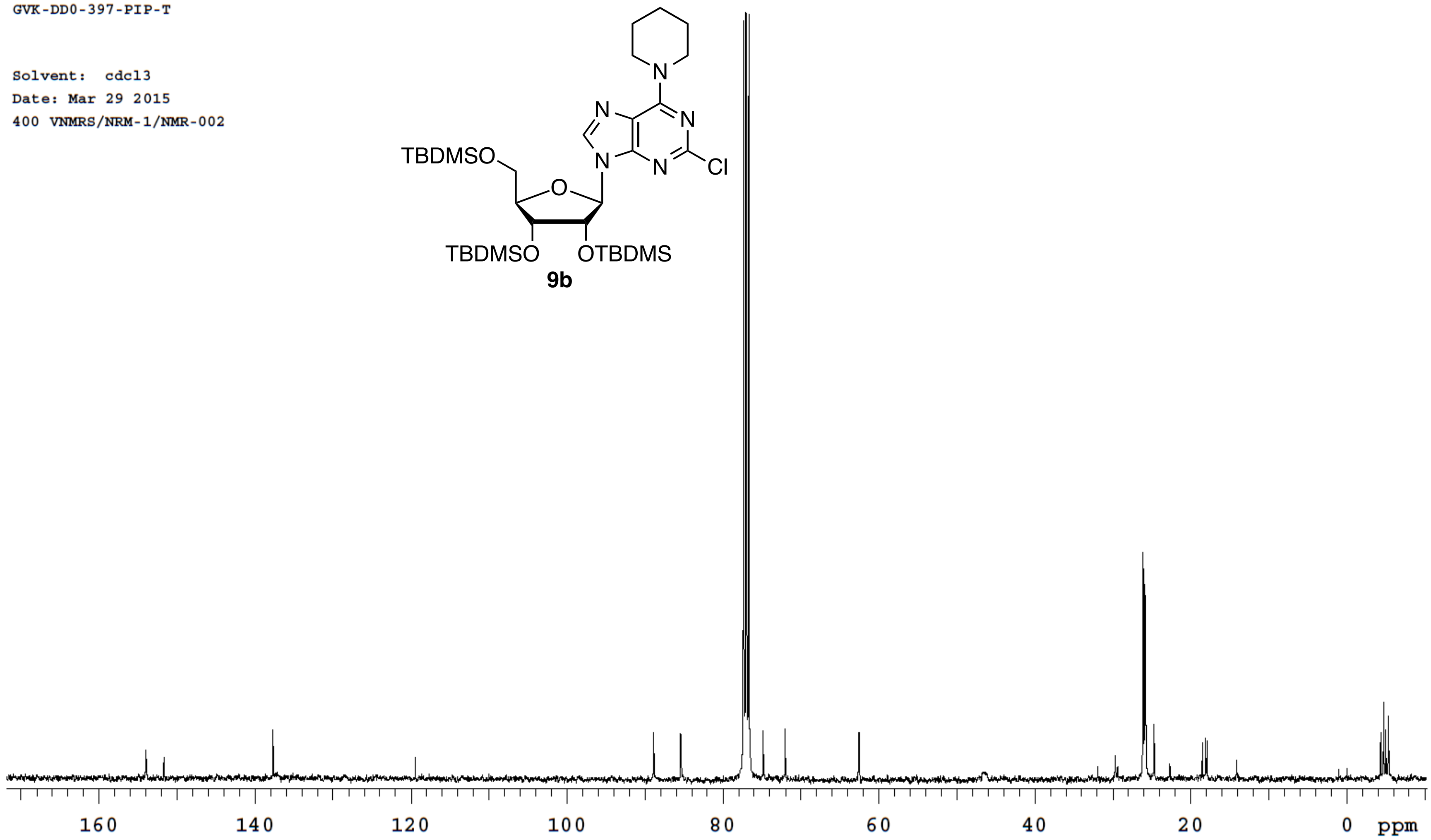

Plotname: 021503D2568_CARBON_01_plot01

Sample Name :
GVK-DD0397-E109832-M
Solvent: cdcl3
400-MR/NRM-1/NMR-001
Date: Feb 27 2015

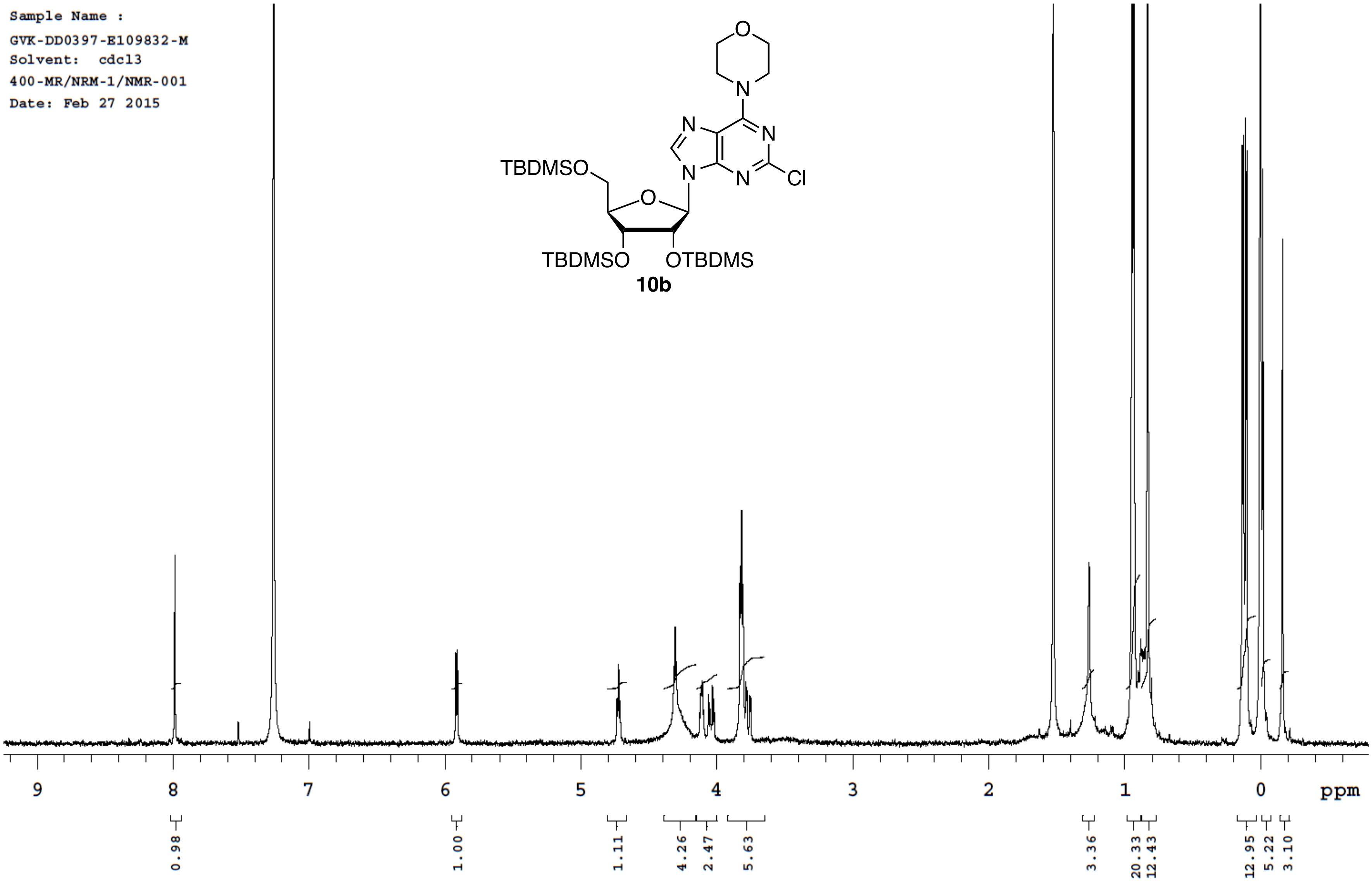

Plotname: 021502D3581_PROTON_01_plot06

Sample Name :
GVK-DD0397-MOR-T

Solvent: cdcl3
Date: May 4 2015
400 VNMRS/NRM-1/NMR-002

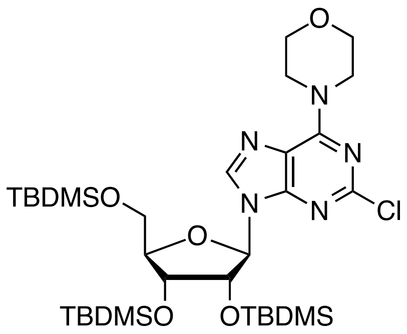

10b

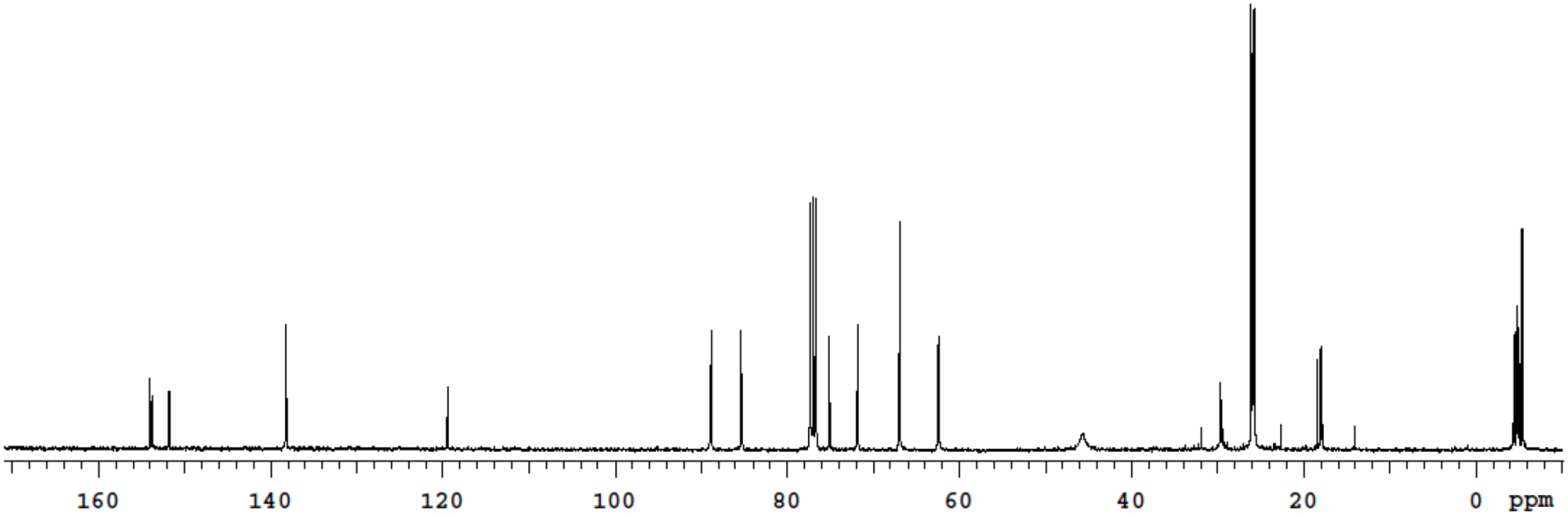

Sample Name:
GVK-DD0-397-TME-T
Solvent: cdcl3
400 VNMRS/NRM-1/NMR-002
Temp. 30.0 C / 303.1 K
Relax. delay 1.000 sec
Acq. time 1.000 sec
Pulse 30.0 degrees
Line broadening 0.5 Hz
Date: Mar 29 2015

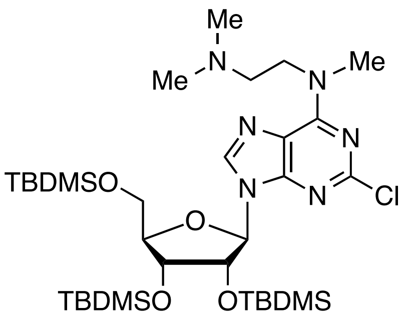

11b

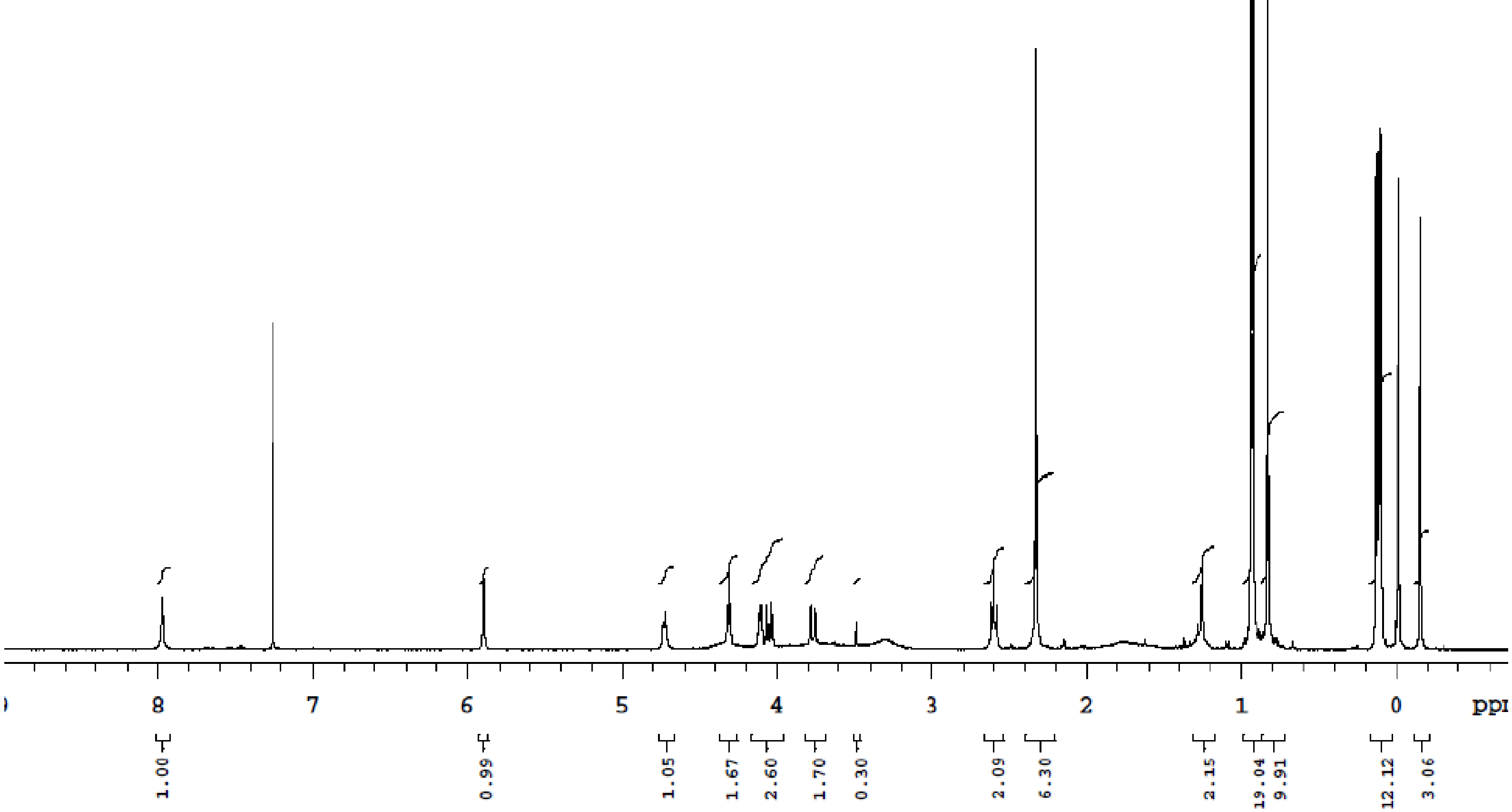

Sample Name :
GVK-DD0-397-TME-T

Solvent: cdcl3
Date: Mar 29 2015
400 VNMRS/NRM-1/NMR-002

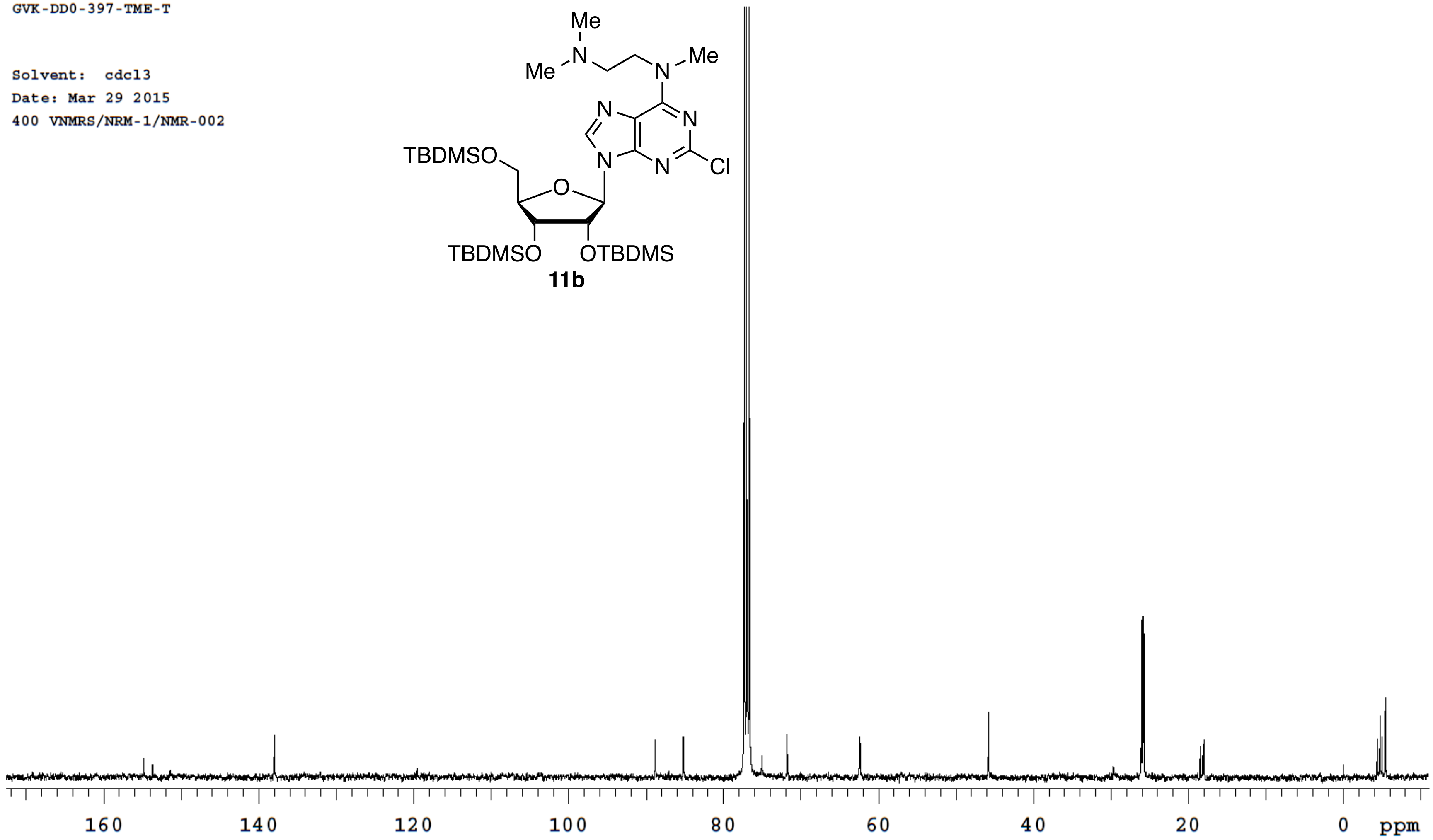

Plotname: 021503D2561_CARBON_01_plot01

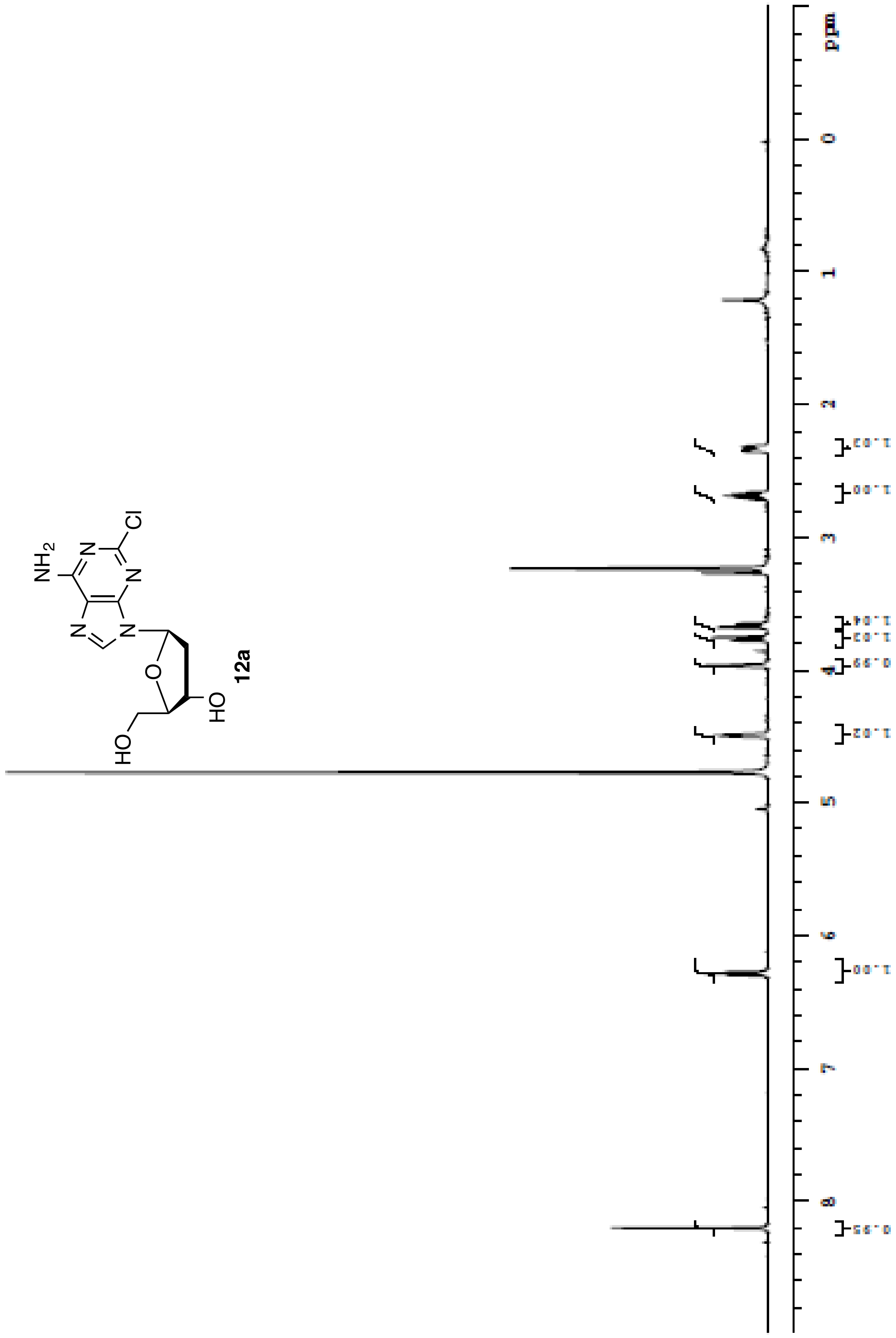

12a

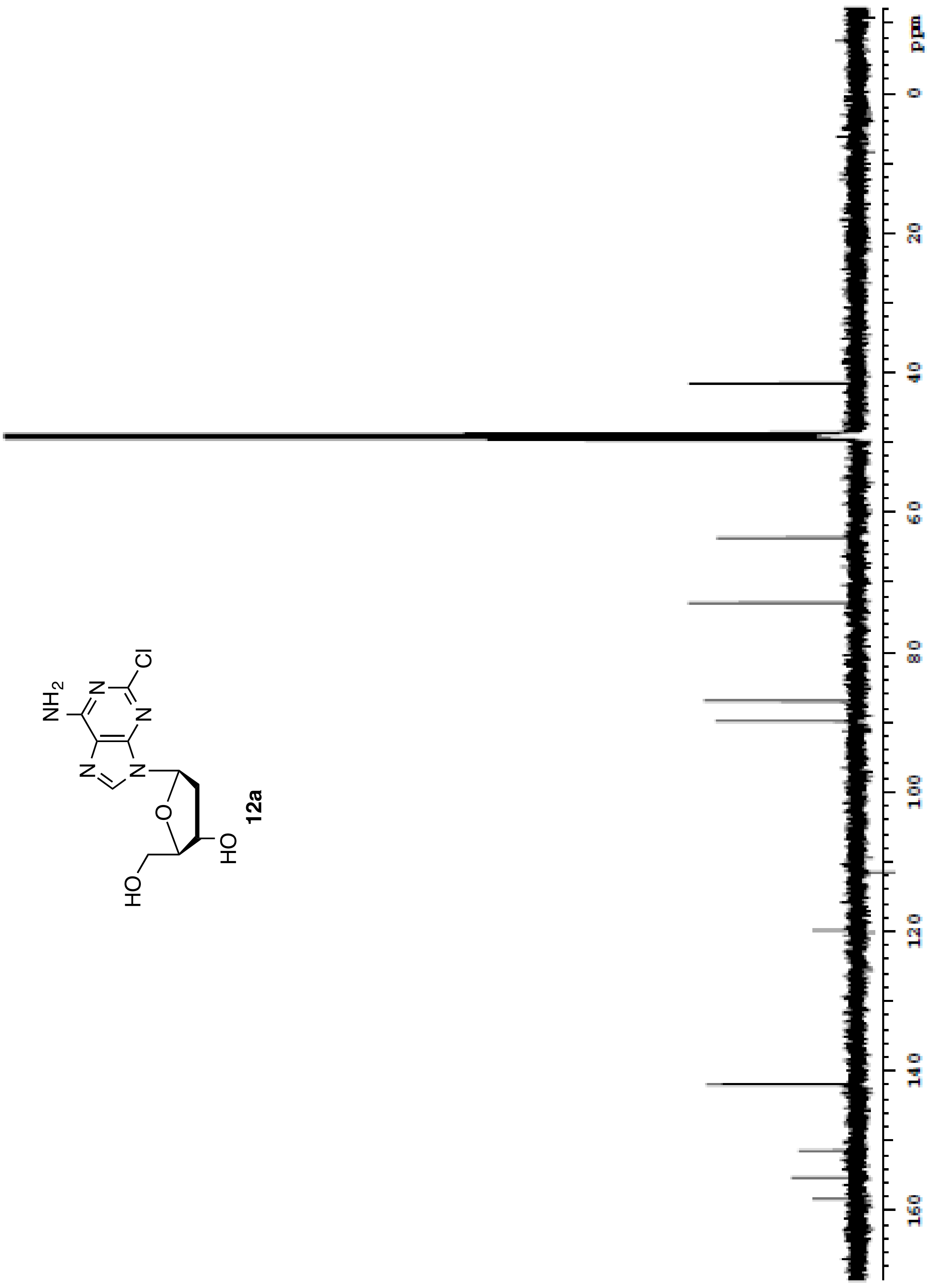

NH2
Cl
N
O
HO
HO
12a
ppm
0
20
40
60
80
100
120
140
160

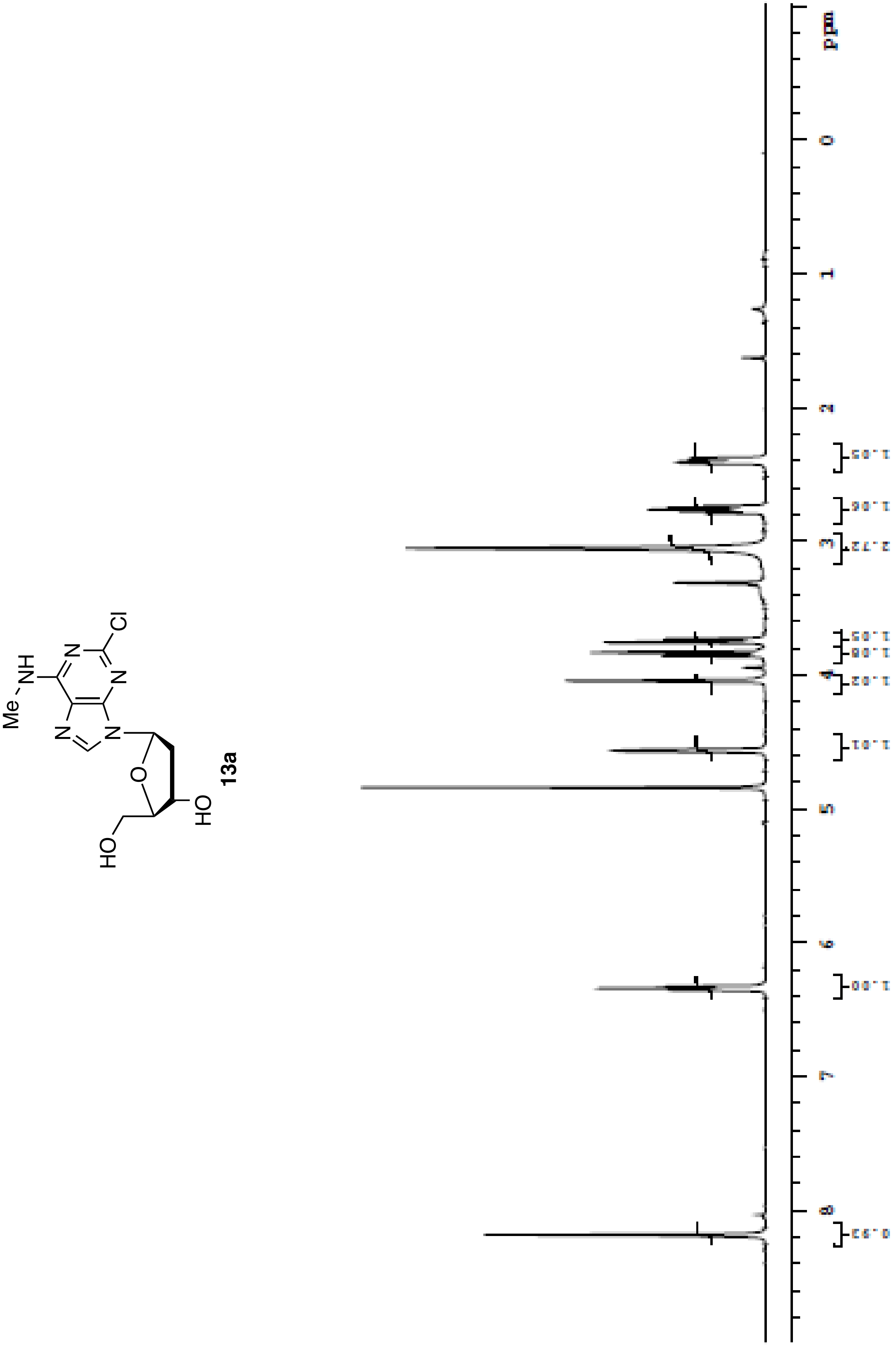

13a

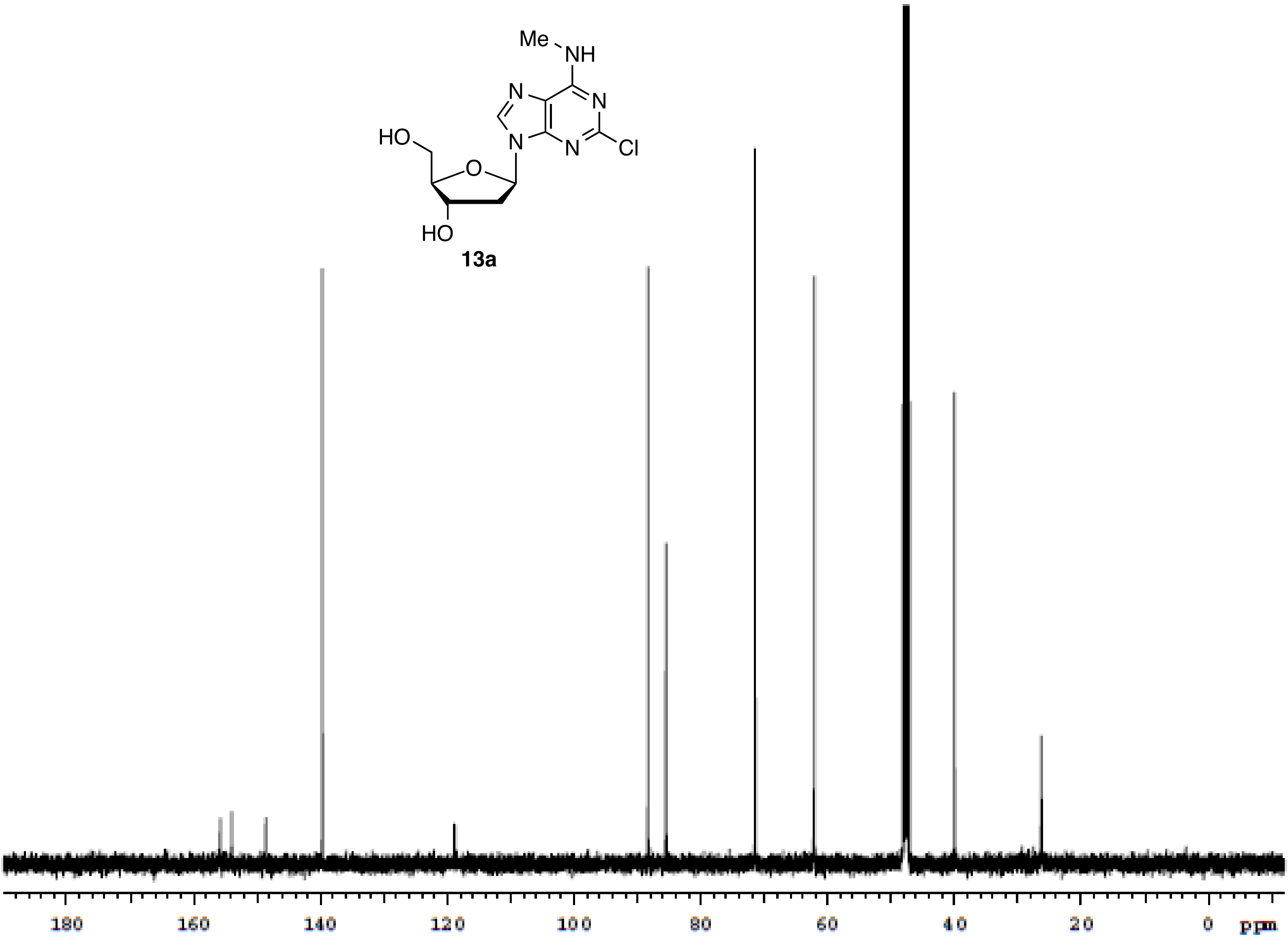

13a

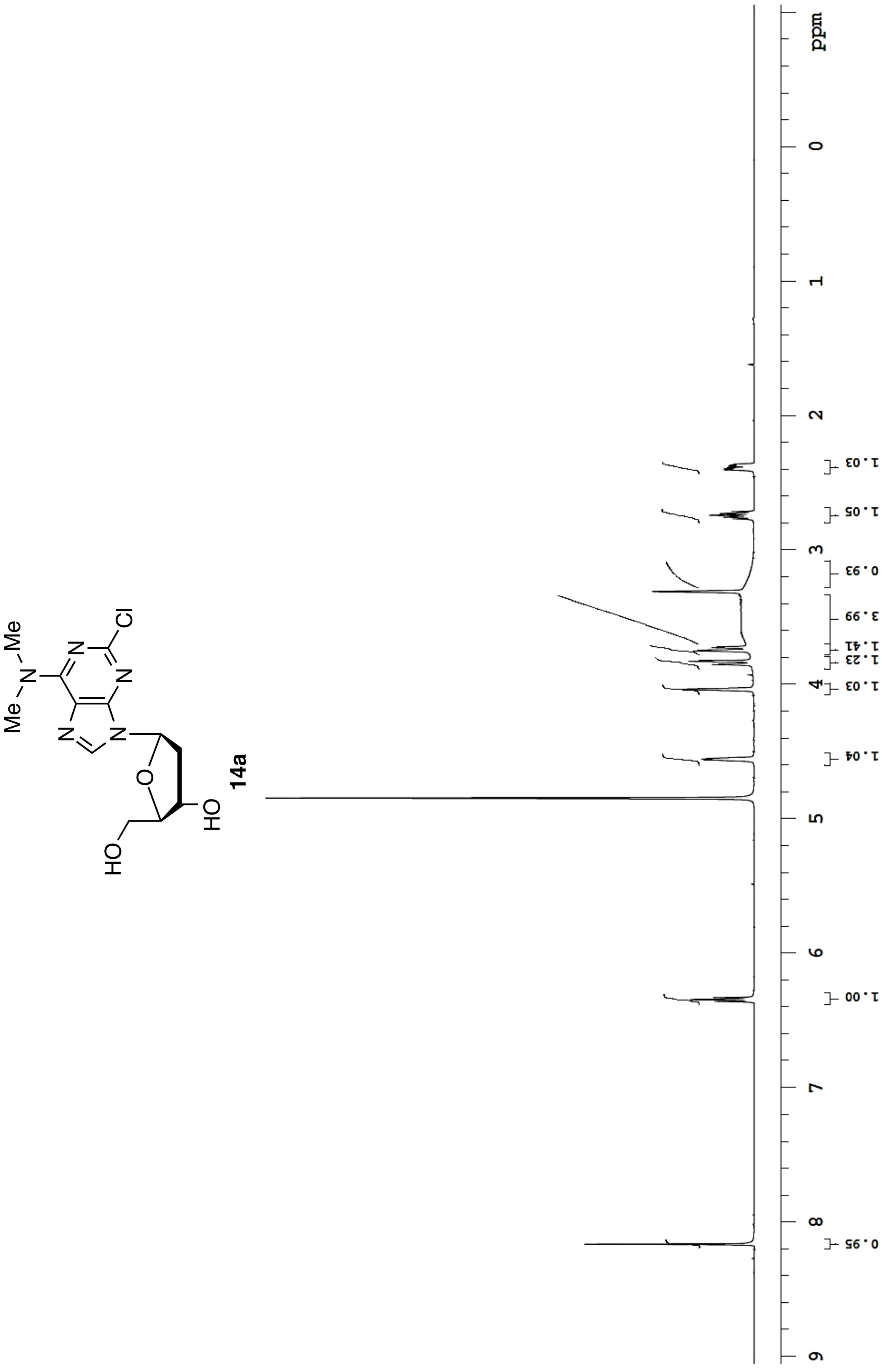
Me
N
Me
N
N
Cl
N
N
O
HO
HO
ppm
0
1
2
3
4
5
6
7
8
9
1.03
1.05
0.93
3.99
1.41
1.23
1.03
1.04
1.00
0.95

14a

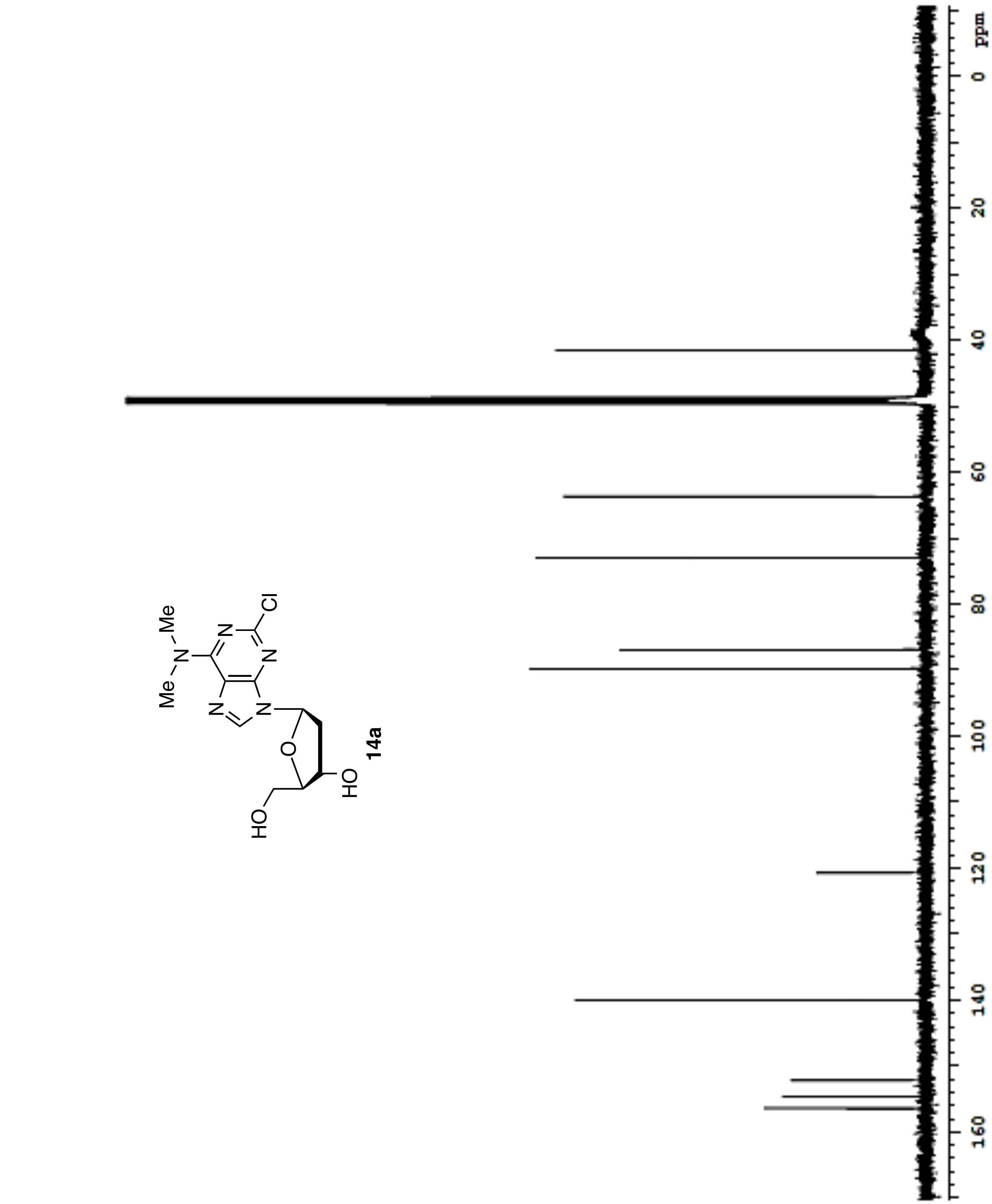
14a
ppm
0
20
40
60
80
100
120
140
160

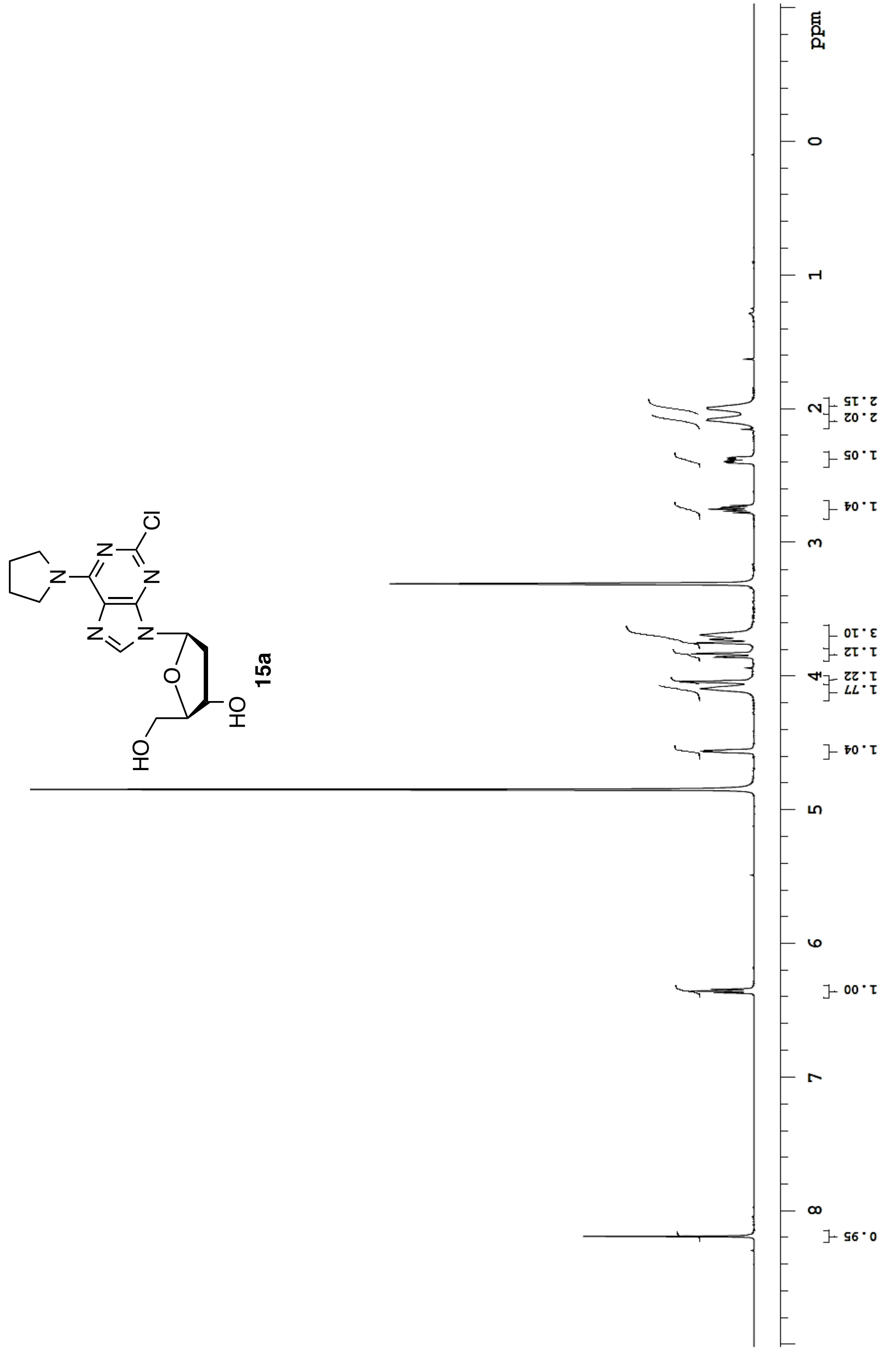

ppm
0
1
2
3
4
5
6
7
8
2.15
2.02
1.05
1.04
3.10
1.12
1.22
1.77
1.04
1.00
0.95
Cl
N
HO
O

15a

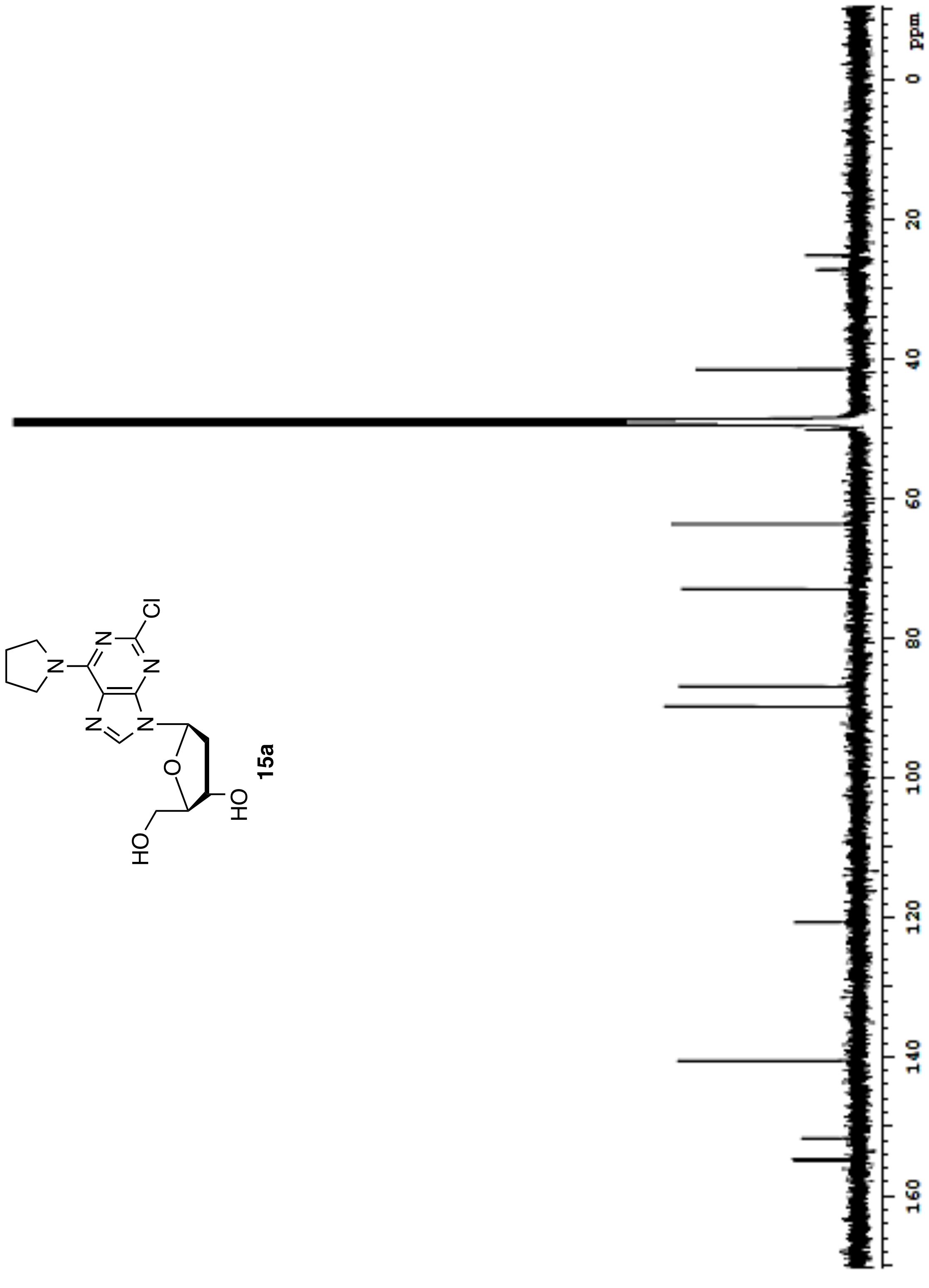
15a
ppm
0
20
40
60
80
100
120
140
160

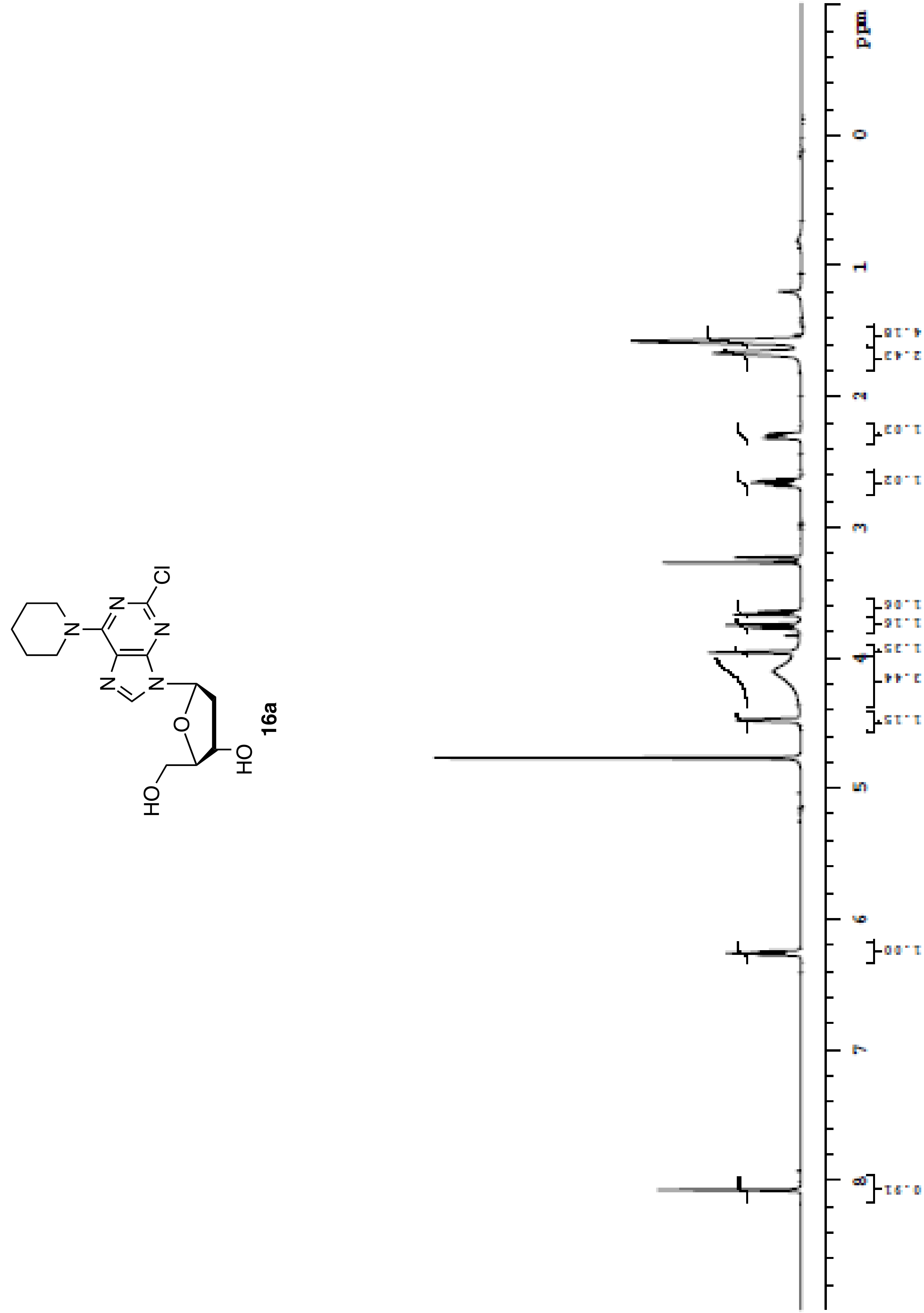

16a

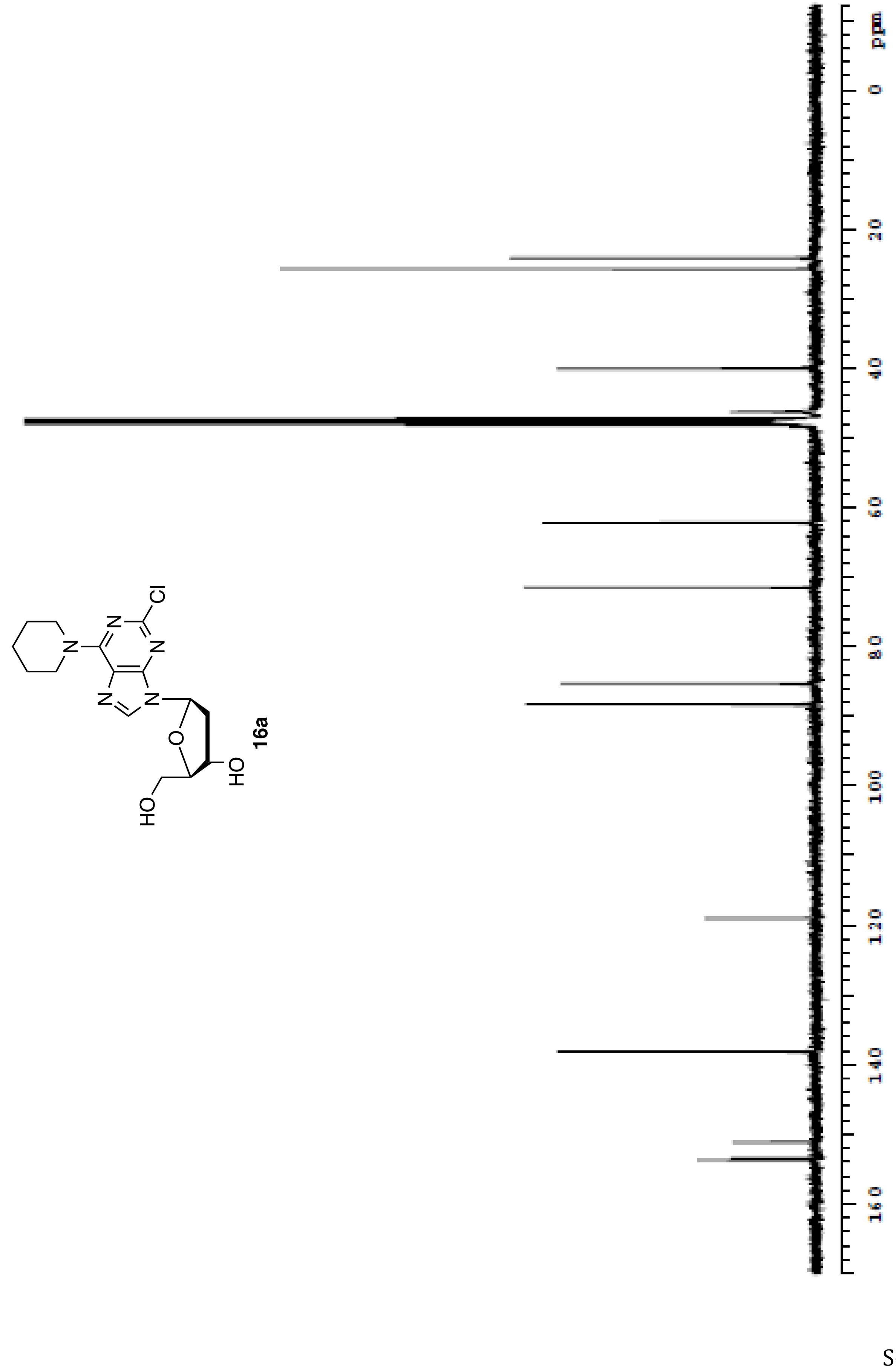
16a
ppm
0
20
40
60
80
100
120
140
160

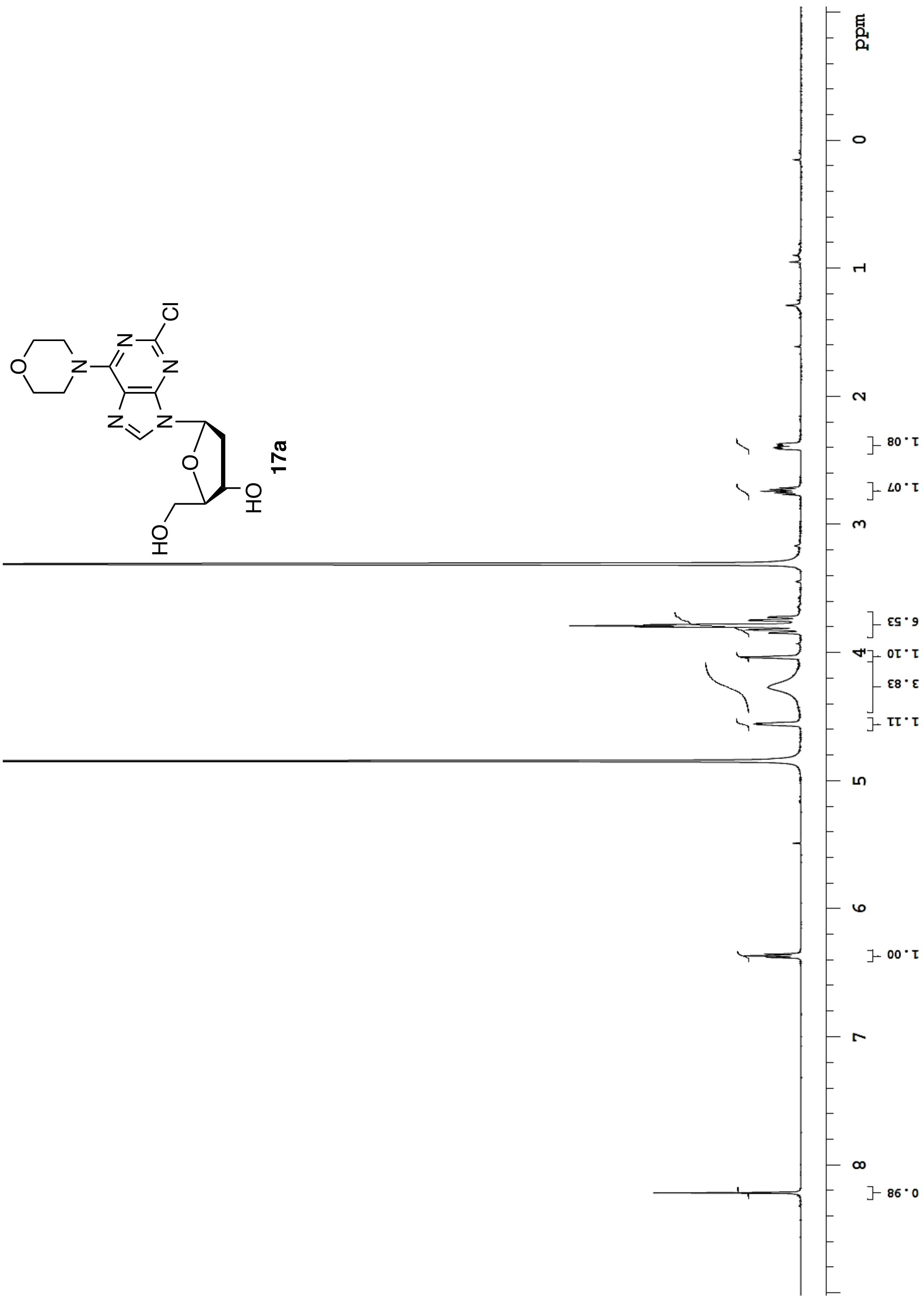

17a
ppm

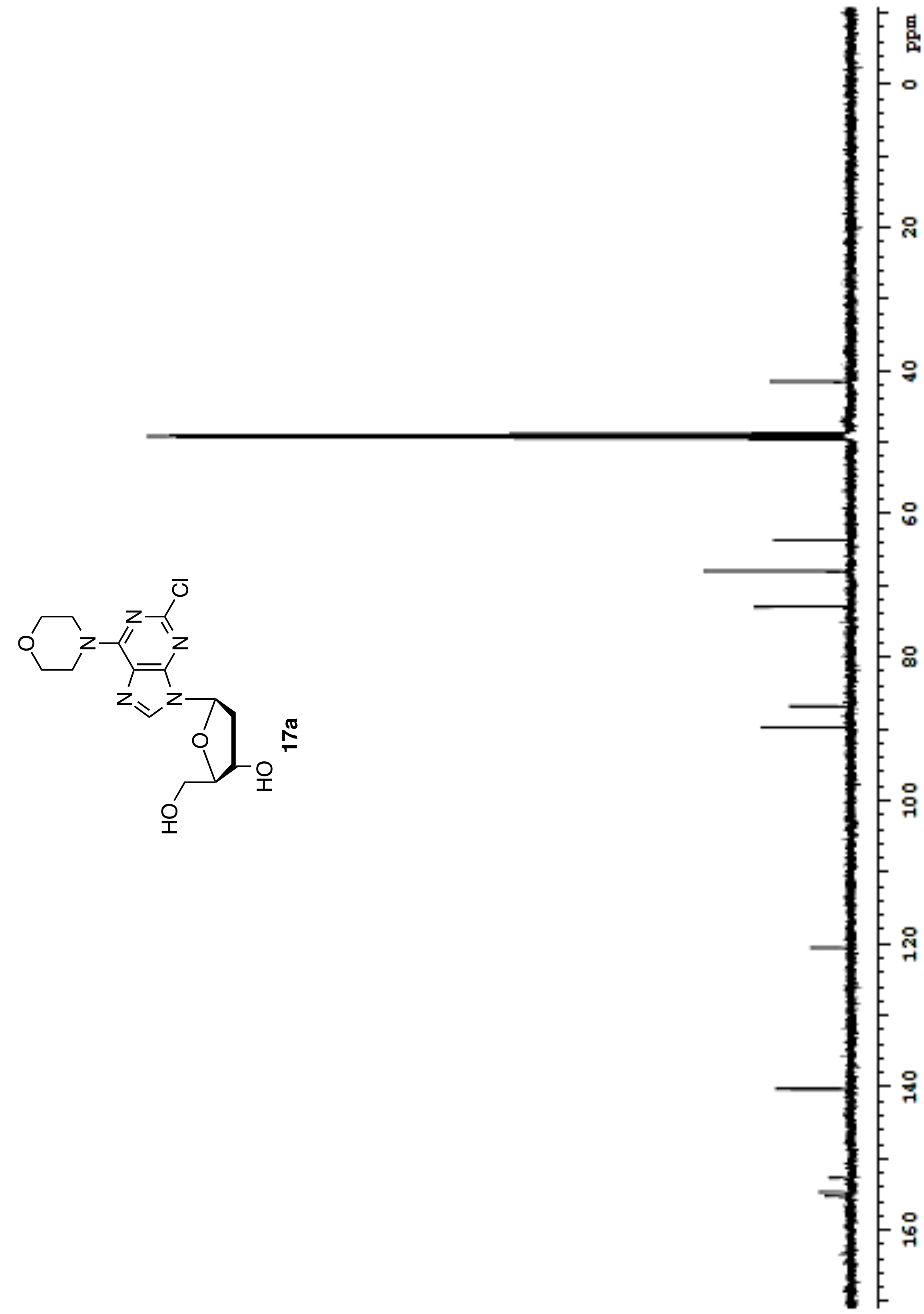

17a
ppm
0
20
40
60
80
100
120
140
160

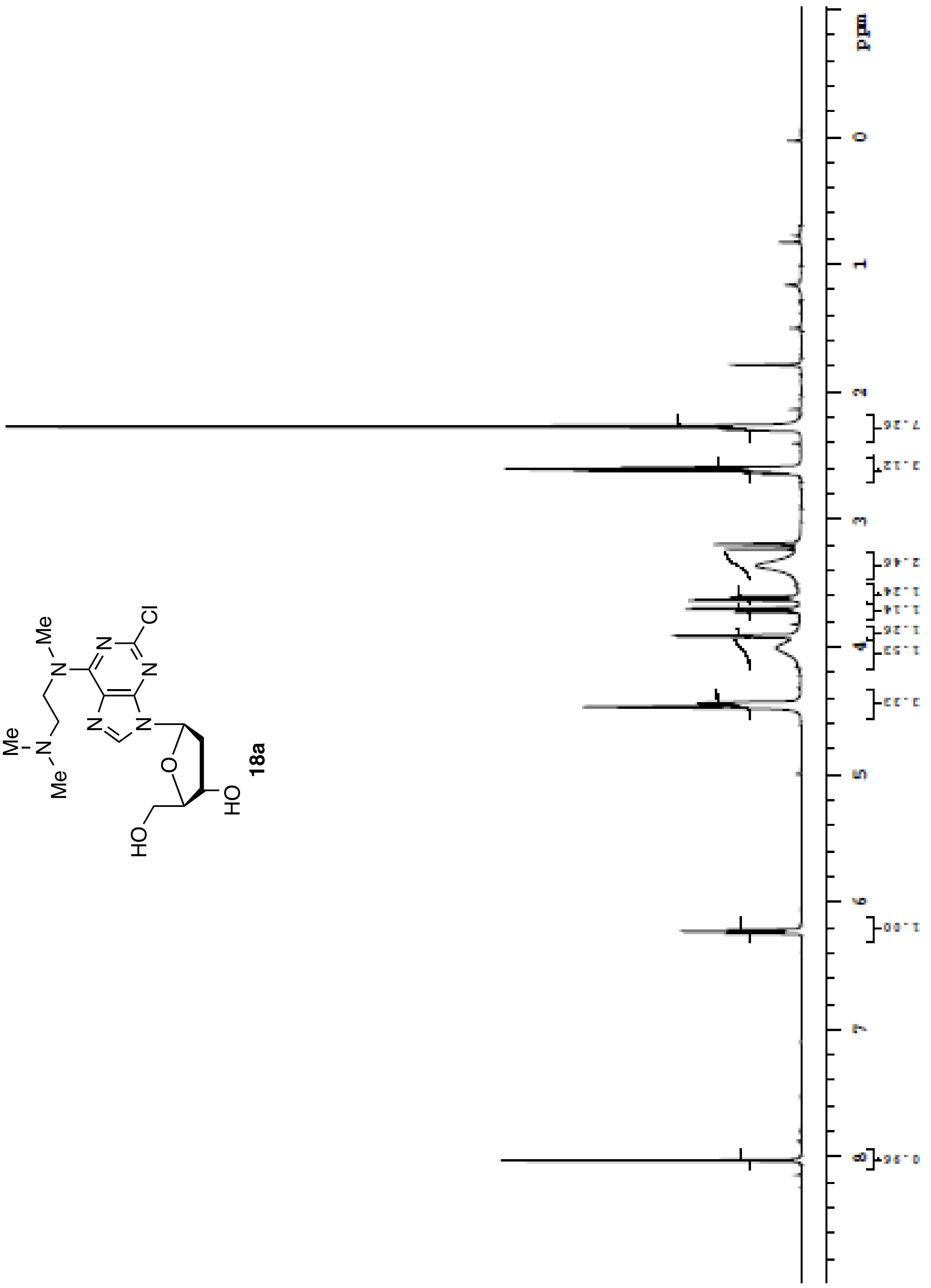
Me
N
Cl
O
HO
OH
18a
ppm
0
1
2
3
4
5
6
7
8

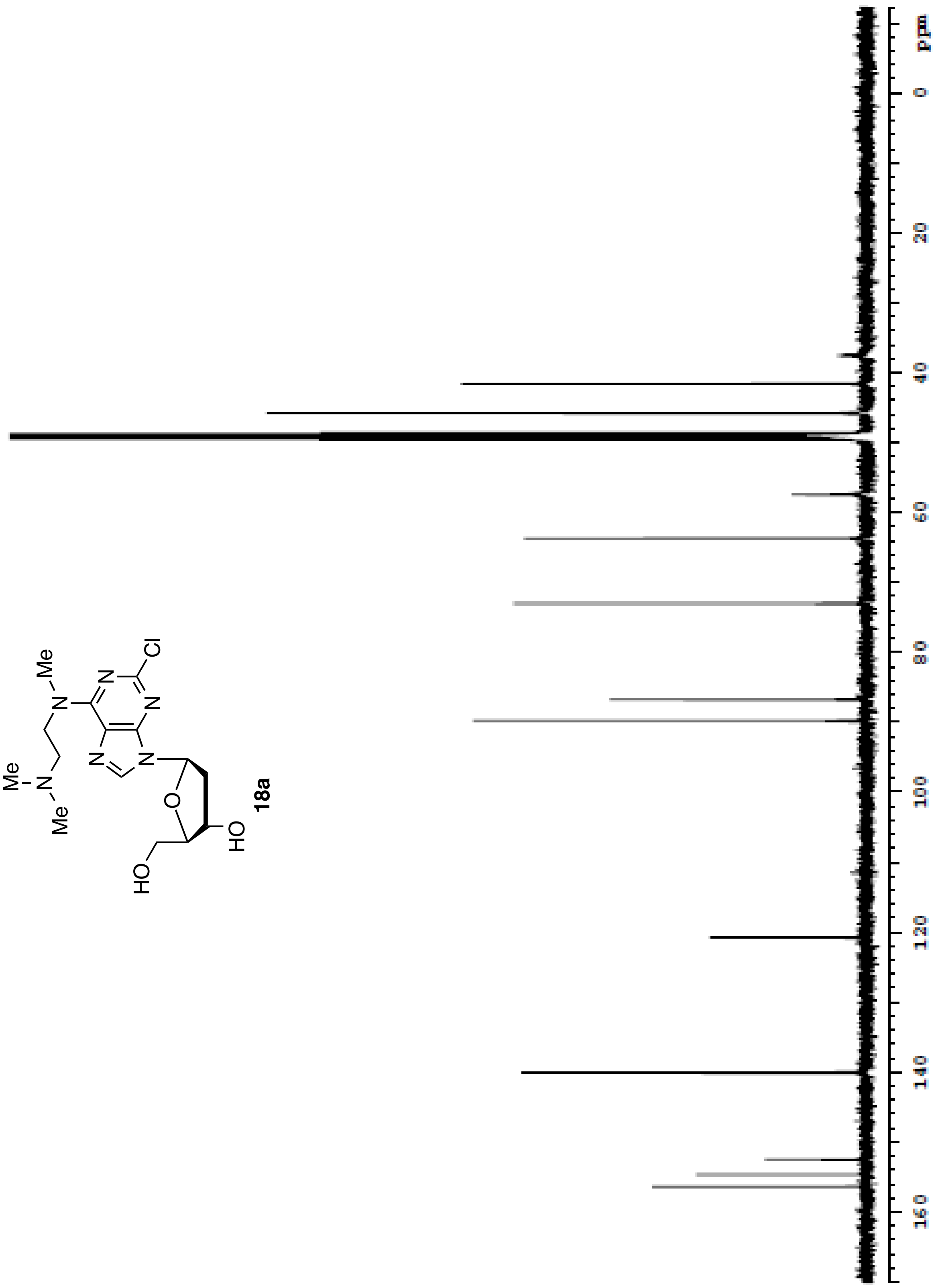
ppm
0
20
40
60
80
100
120
140
160
18a

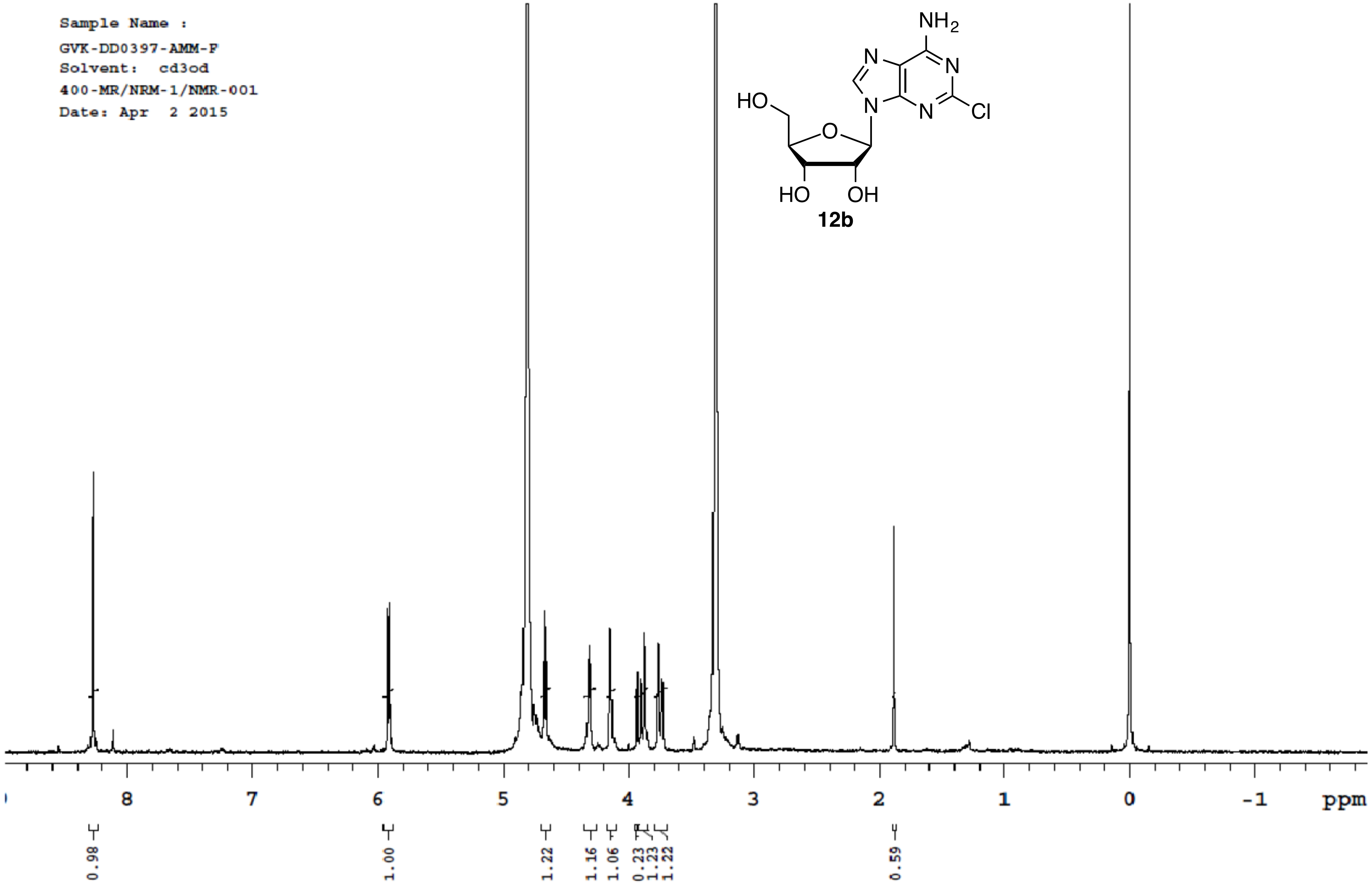

Sample Name :
GVK-DD0397-AMM-F
Solvent: cd3od
400-MR/NRM-1/NMR-001
Date: Apr 2 2015
NH2
N
N
N
N
Cl
HO
O
HO
OH
12b
8
7
6
5
4
3
2
1
0
-1
ppm
0.98
1.00
1.22
1.16
1.06
0.23
1.23
1.22
0.59

GVK-DD0397-Amm-F sol problem
Reference Code: 021504B2827
Solvent: dmso
Archive directory:
/home/gvkbio/data/2015/Apr
Agilent 400-MRDD2
Data collected on: Apr 11 2015
Experiment: CARBON

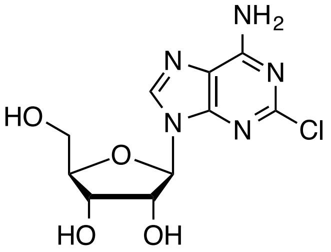

12b

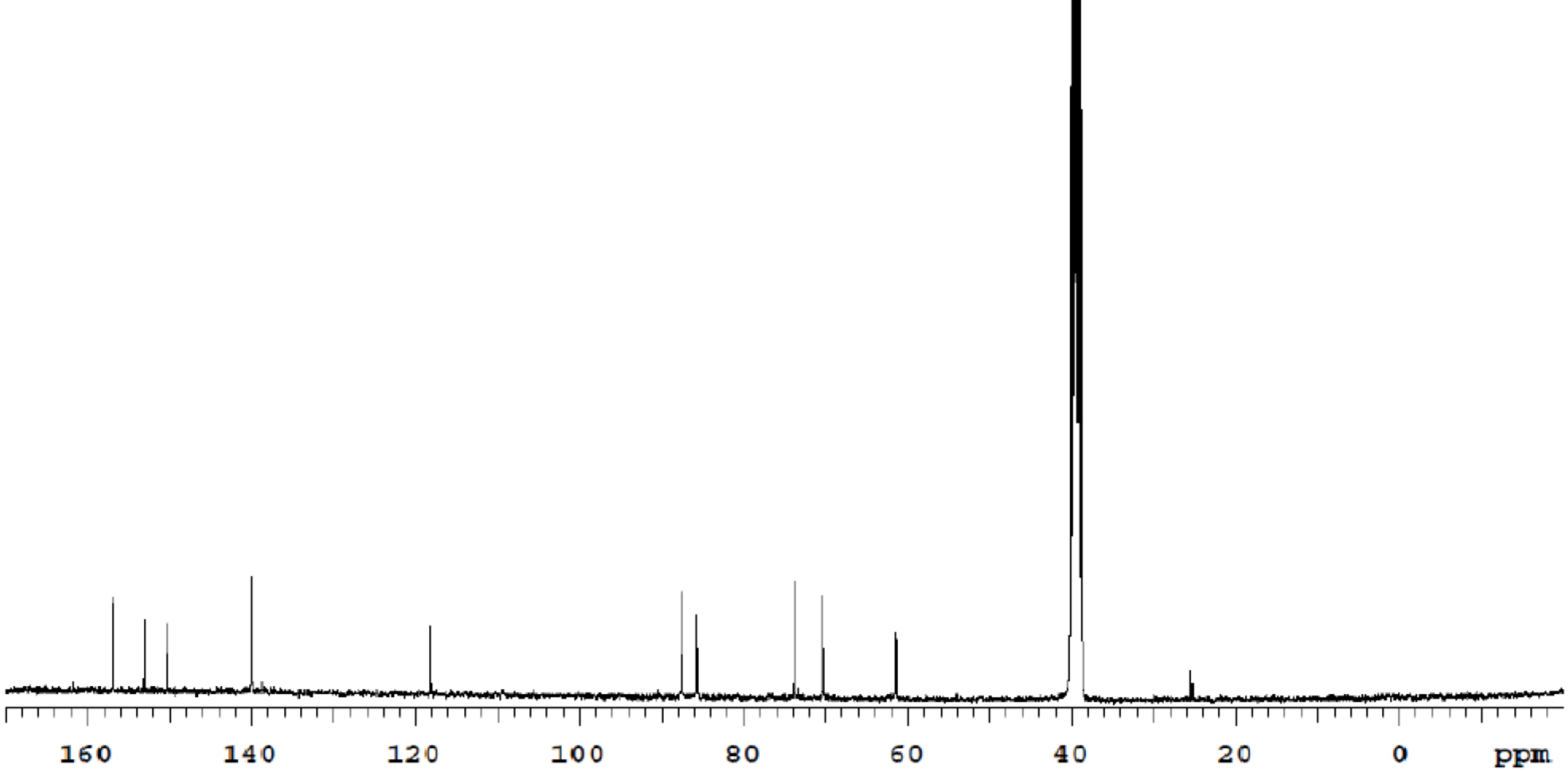

Sample Name :
GVK-DD0397-METHYL-F
Solvent: cd3od
400-MR/NRM-1/NMR-001
Date: Apr 2 2015

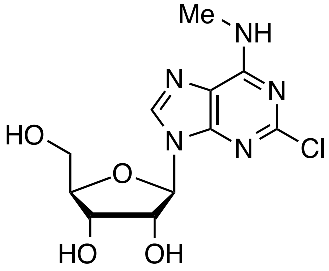

13b

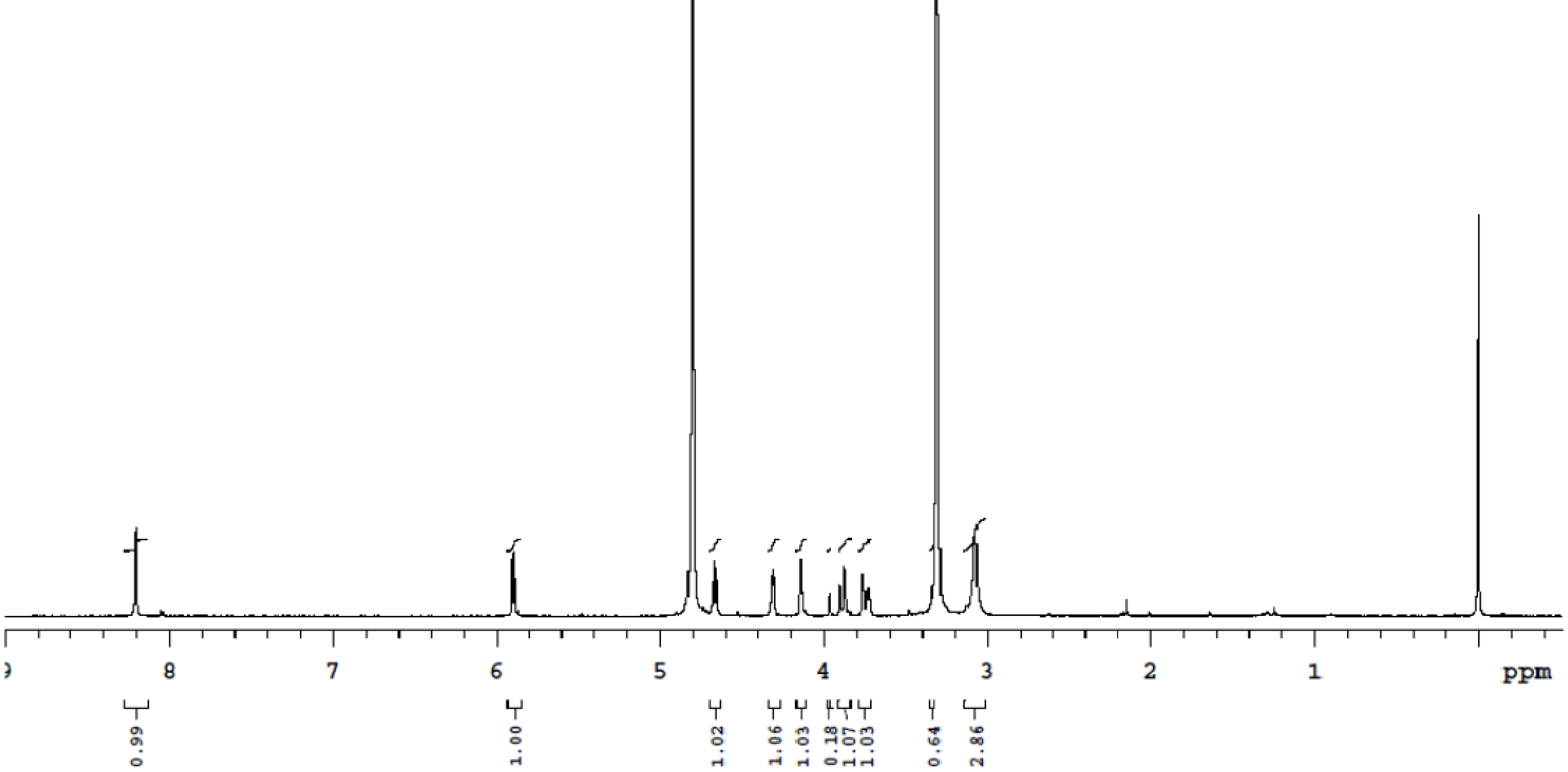

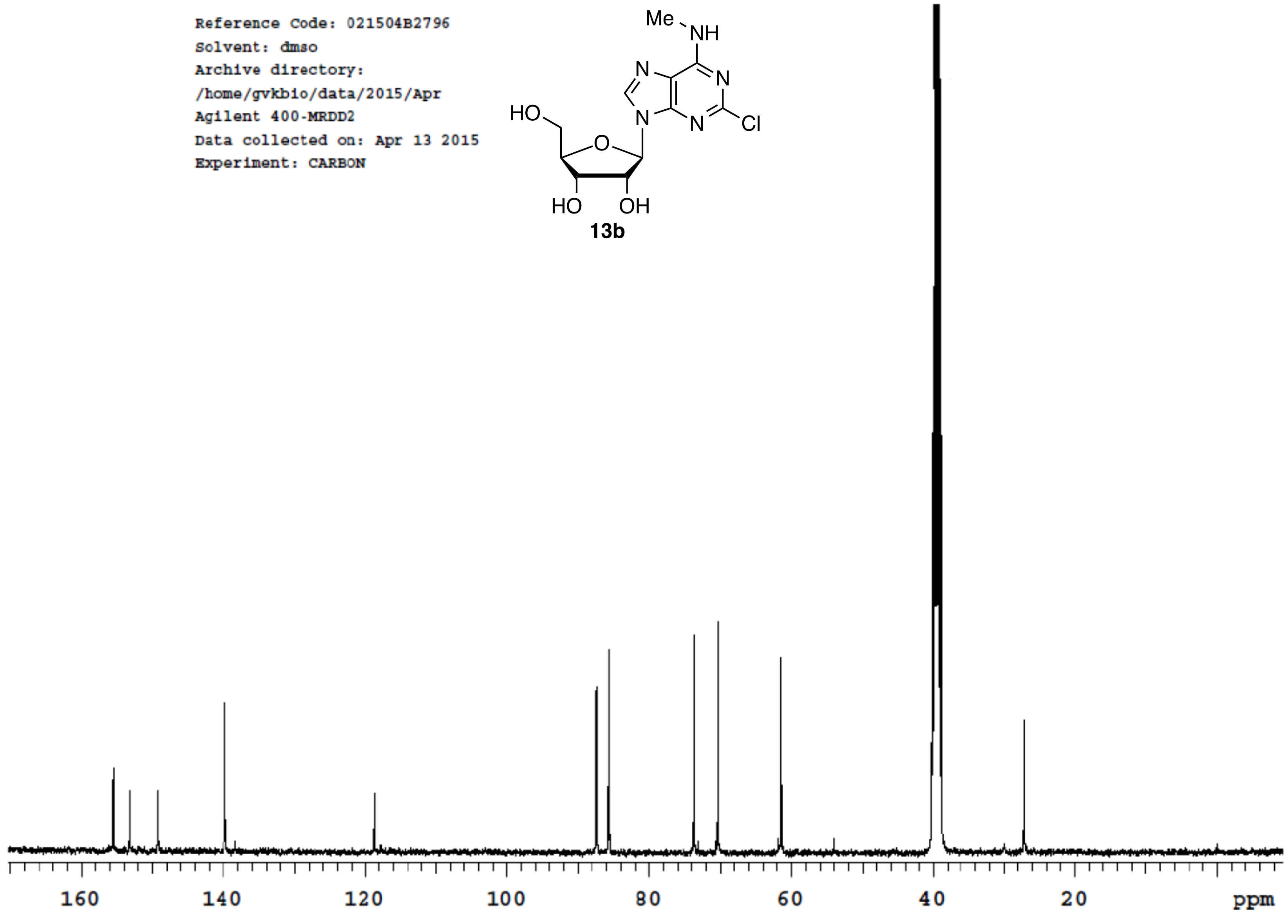
Reference Code: 021504B2796
Solvent: dmso
Archive directory:
/home/gvkbio/data/2015/Apr
Agilent 400-MRDD2
Data collected on: Apr 13 2015
Experiment: CARBON
Me
NH
N
N
N
N
Cl
HO
O
HO
OH
13b
160
140
120
100
80
60
40
20
ppm

b

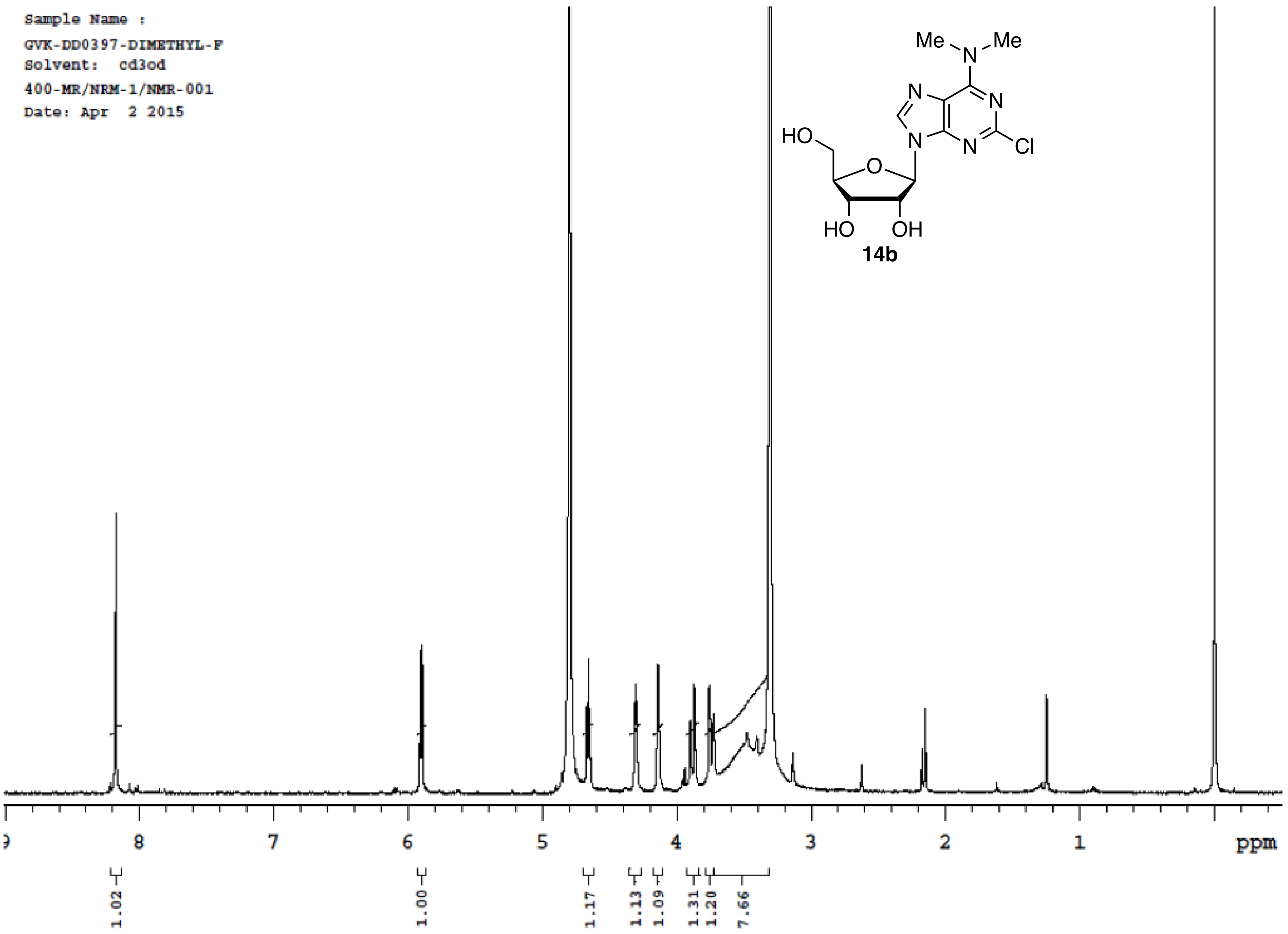
Sample Name :
GVK-DD0397-DIMETHYL-F
Solvent: cd3od
400-MR/NRM-1/NMR-001
Date: Apr 2 2015
Me
N
Me
N
N
N
N
Cl
HO
O
HO
OH
14b
9
8
7
6
5
4
3
2
1
ppm
1.02
1.00
1.17
1.13
1.09
1.31
1.20
7.66

GVK-DD0397-DMe-F
Reference Code: 021504B2813
Solvent: dmso
Archive directory:
/home/gvkbio/data/2015/Apr
Agilent 400-MRDD2
Data collected on: Apr 12 2015
Experiment: CARBON

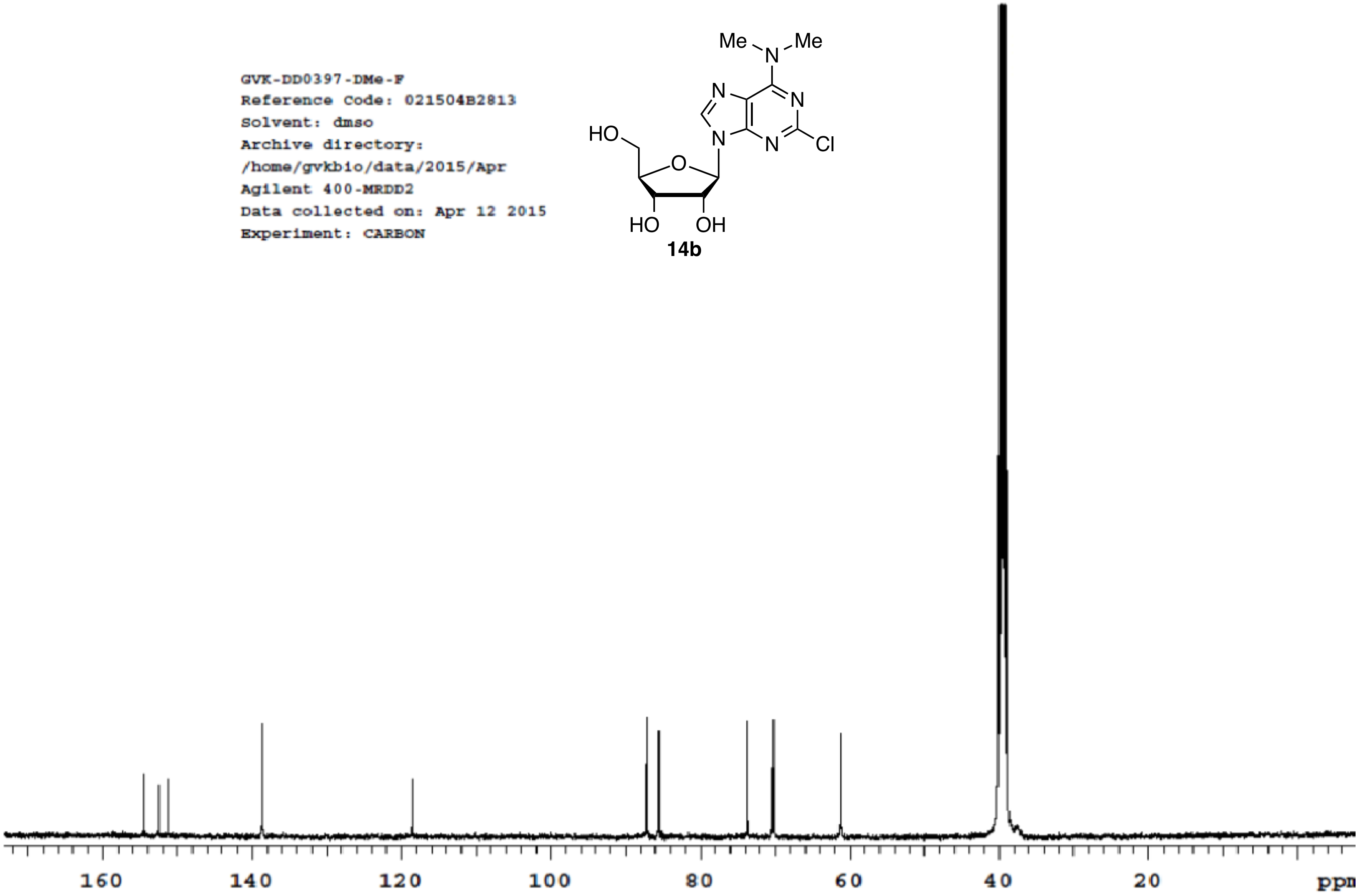

14b

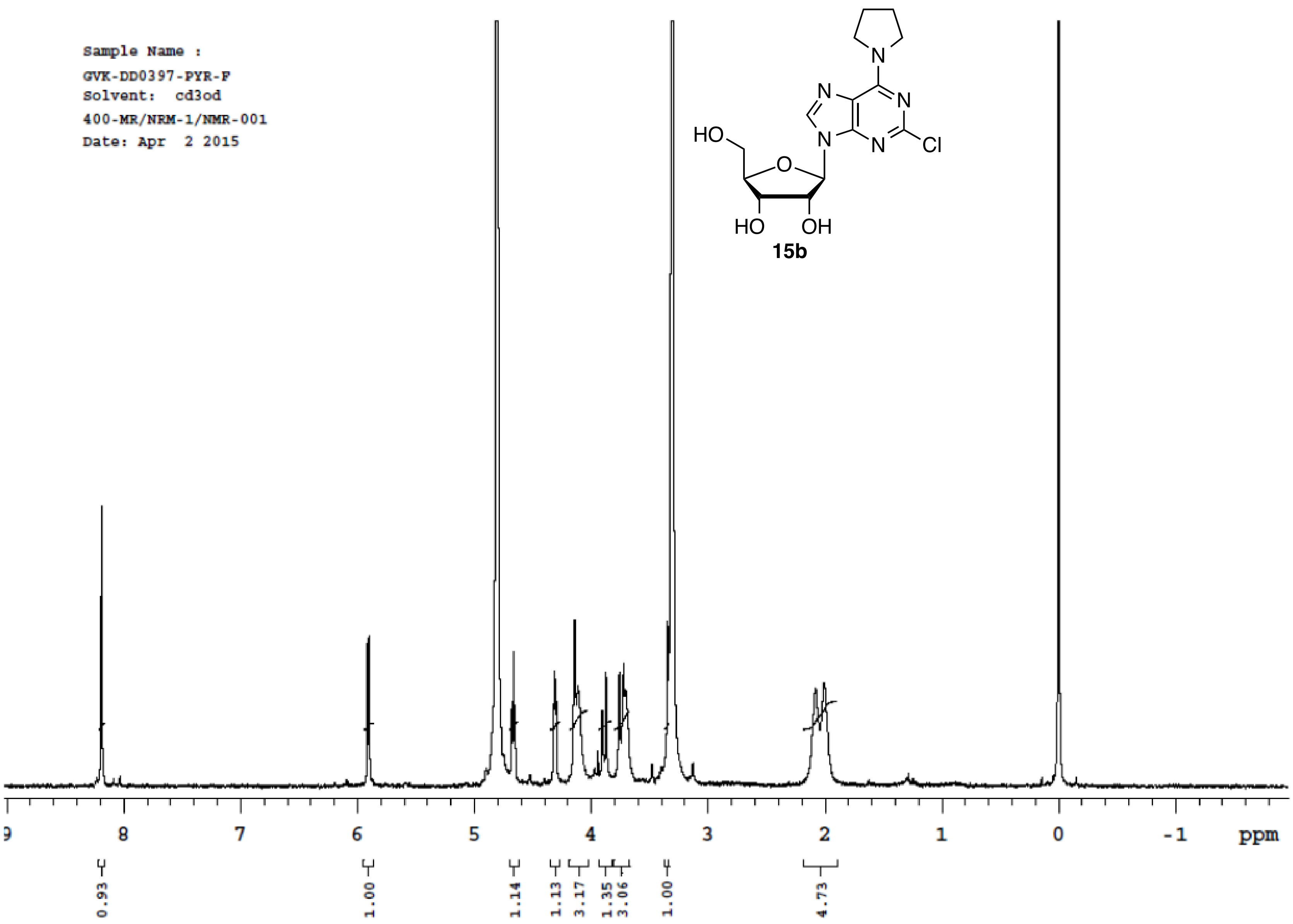
Sample Name :
GVK-DD0397-PYR-F
Solvent: cd3od
400-MR/NRM-1/NMR-001
Date: Apr 2 2015
HO
O
N
N
N
N
N
Cl
HO
OH
15b
9
8
7
6
5
4
3
2
1
0
-1
ppm
0.93
1.00
1.14
1.13
3.17
1.35
3.06
1.00
4.73

Sample Name:
GVK-DD0397-pyr-F

Solvent: dmso
Date: Apr 10 2015
Agilent 400-MR / NRM-3
Request No: 021504B1188_CARBON

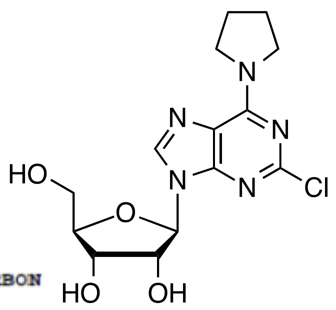

15b

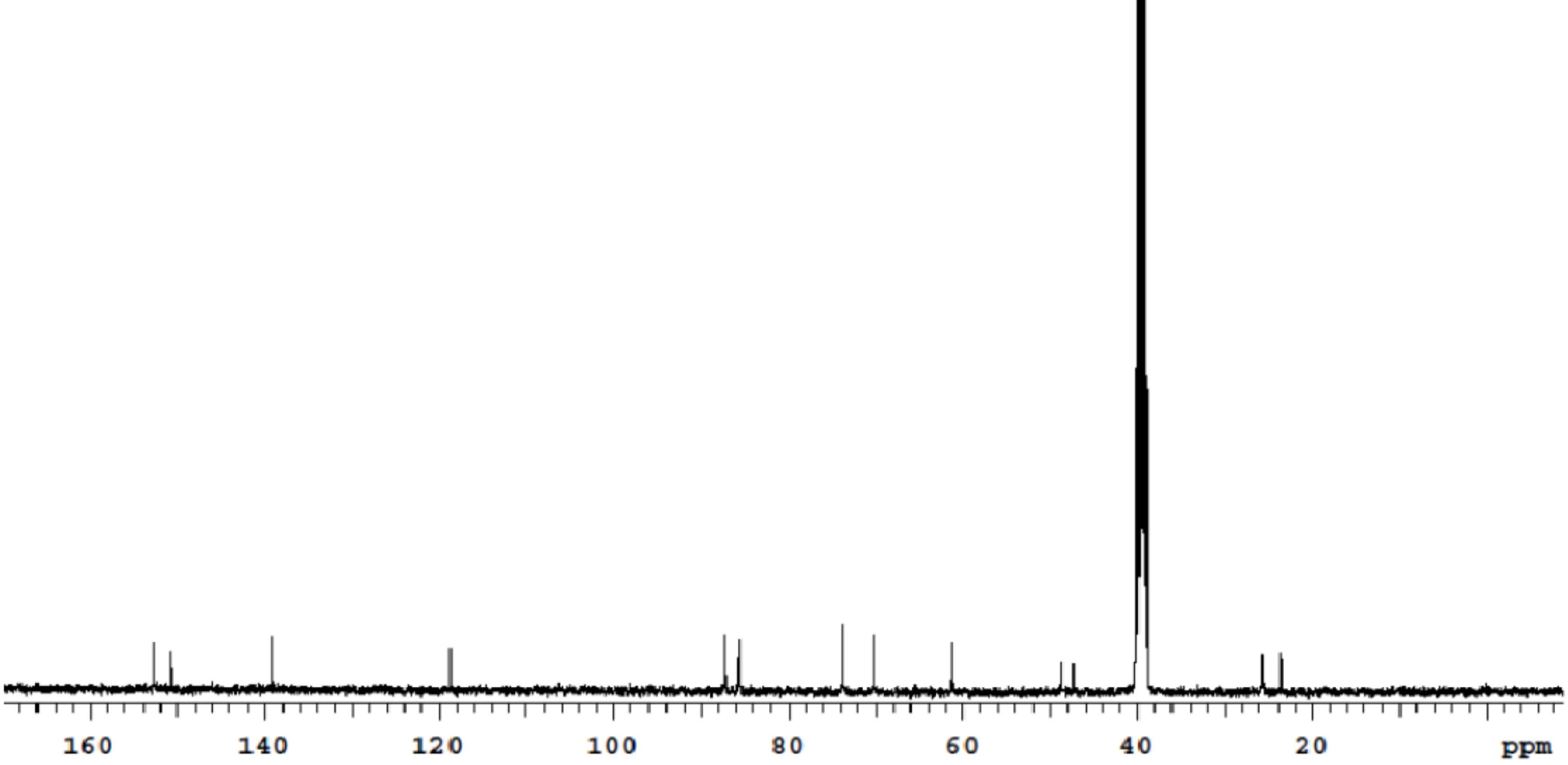

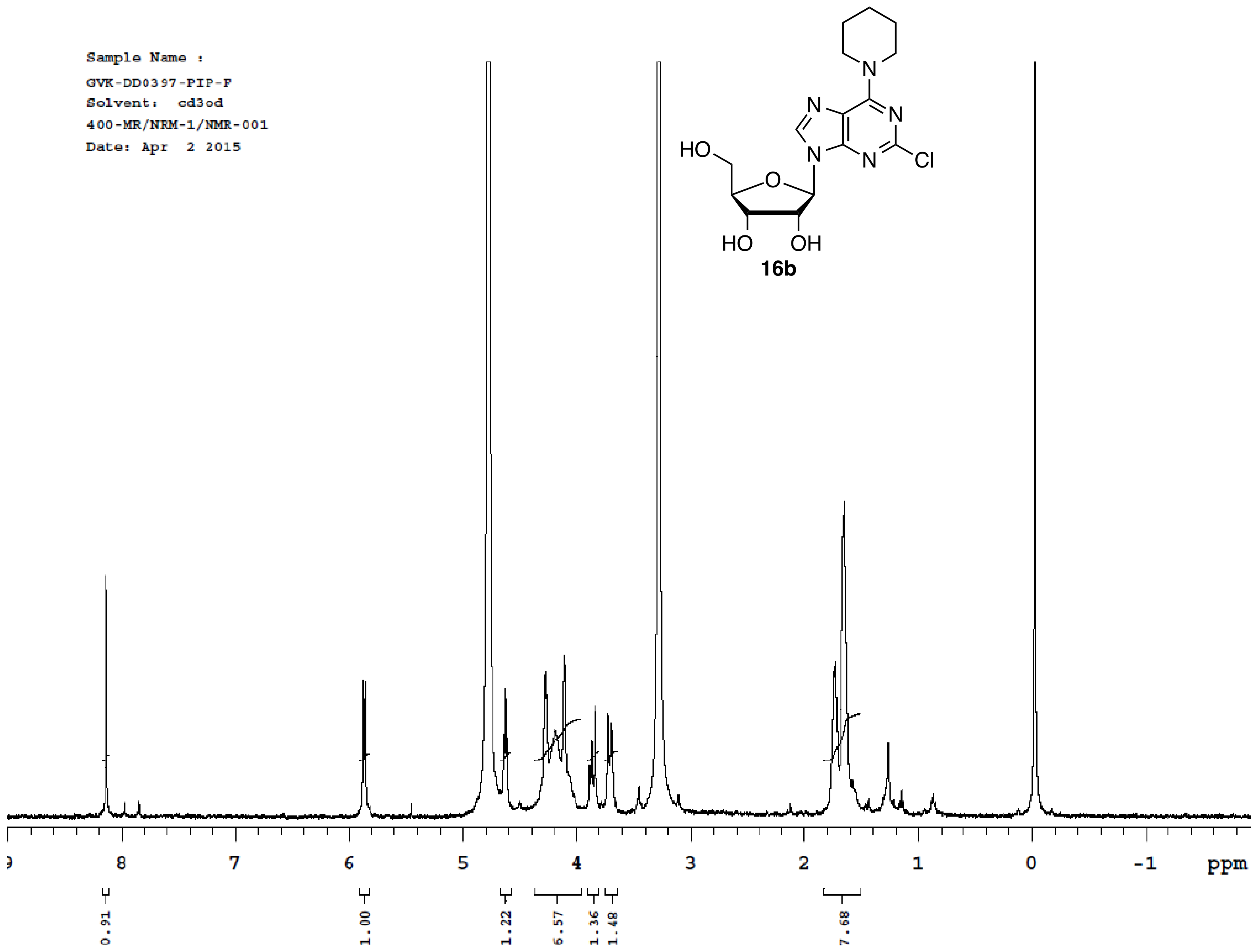
Sample Name :
GVK-DD0397-PIP-F
Solvent: cd3od
400-MR/NRM-1/NMR-001
Date: Apr 2 2015
16b
ppm
0.91
1.00
1.22
6.57
1.36
1.48
7.68

GVK-DD0397-PIP-F
Reference Code: 021504B2820
Solvent: dmso
Archive directory:
/home/gvkbio/data/2015/Apr
Agilent 400-MRDD2
Data collected on: Apr 11 2015
Experiment: CARBON

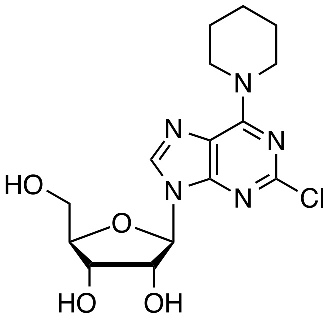

16b

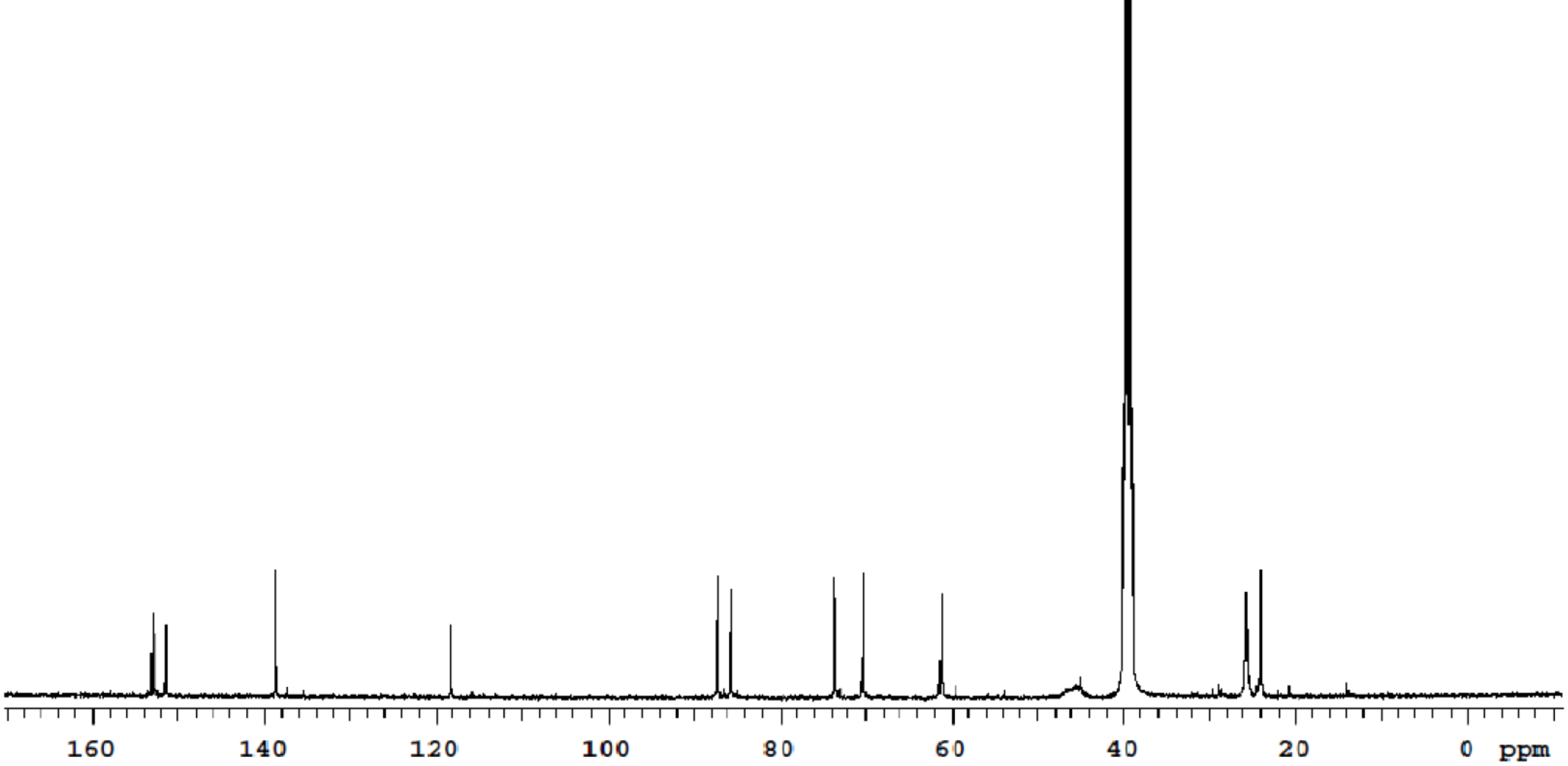

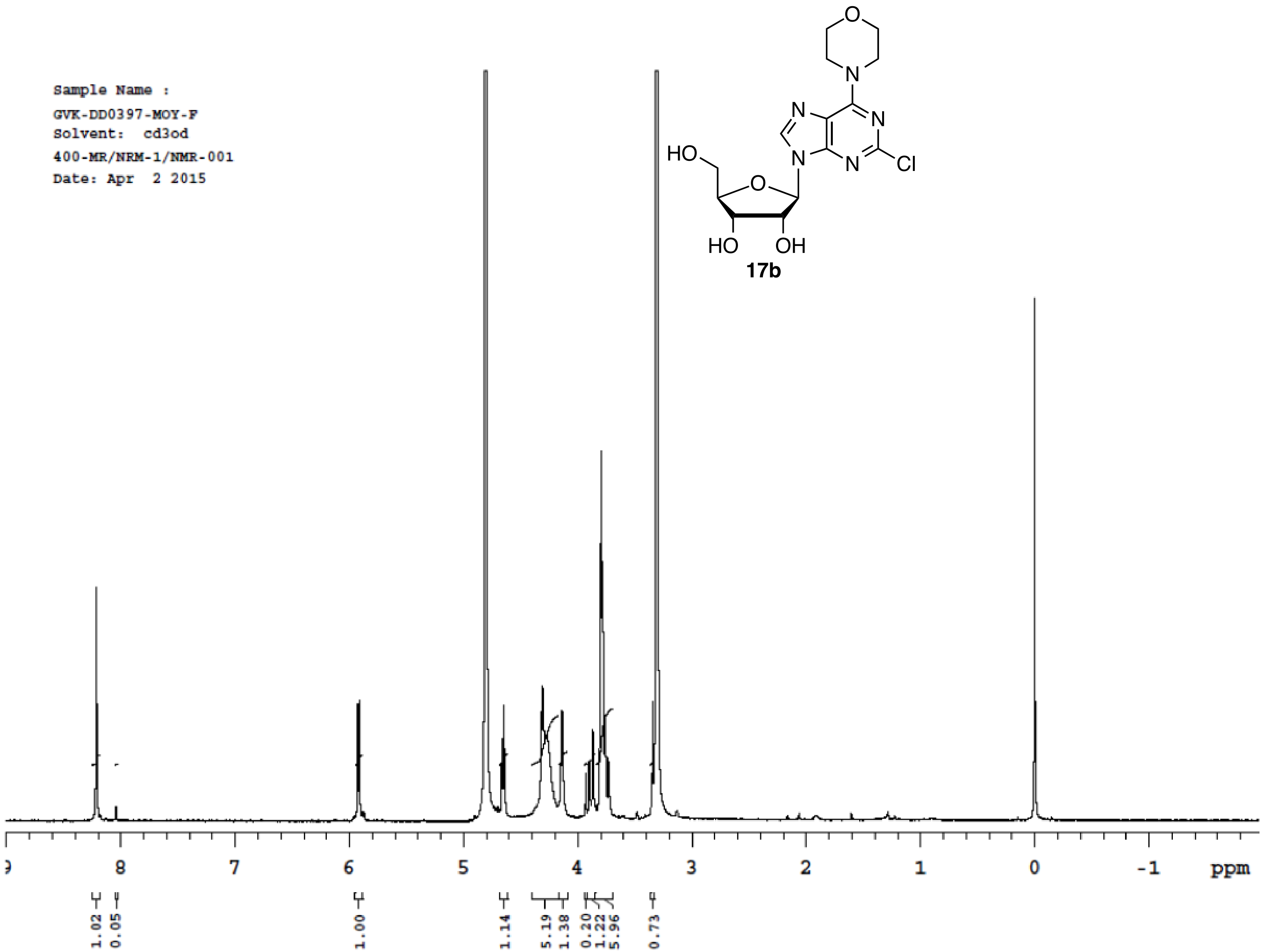
Sample Name :
GVK-DD0397-MOY-F
Solvent: cd3od
400-MR/NRM-1/NMR-001
Date: Apr 2 2015
HO
O
N
N
N
N
Cl
O
N
HO
OH
17b
9
8
7
6
5
4
3
2
1
0
-1
ppm
1.02
0.05
1.00
1.14
5.19
1.38
0.20
1.22
5.96
0.73

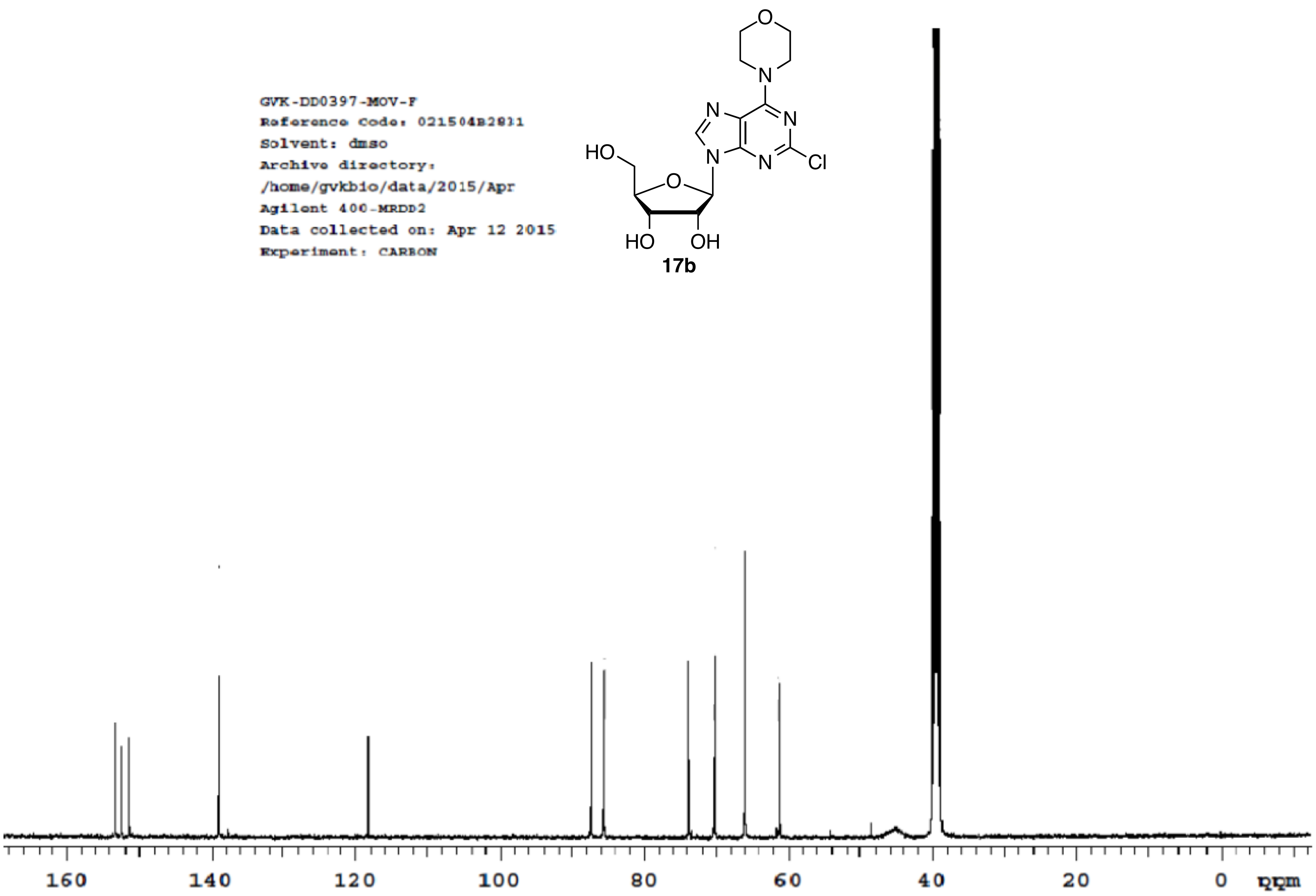
GVK-DD0397-MOV-F
Reference Code: 021504B2811
Solvent: dmso
Archive directory:
/home/gvkbio/data/2015/Apr
Agilent 400-MRDD2
Data collected on: Apr 12 2015
Experiment: CARBON
HO
O
N
N
N
N
N
Cl
O
HO
OH
17b
160
140
120
100
80
60
40
20
0
ppm

Sample Name :
GVK-DD0397-TME-F
Solvent: cd3od
400-MR/NRM-1/NMR-001
Date: May 10 2015

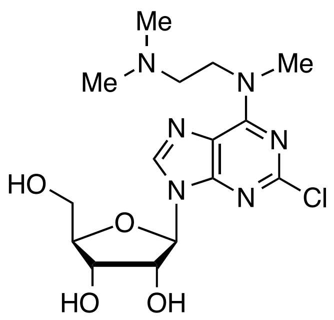

18b

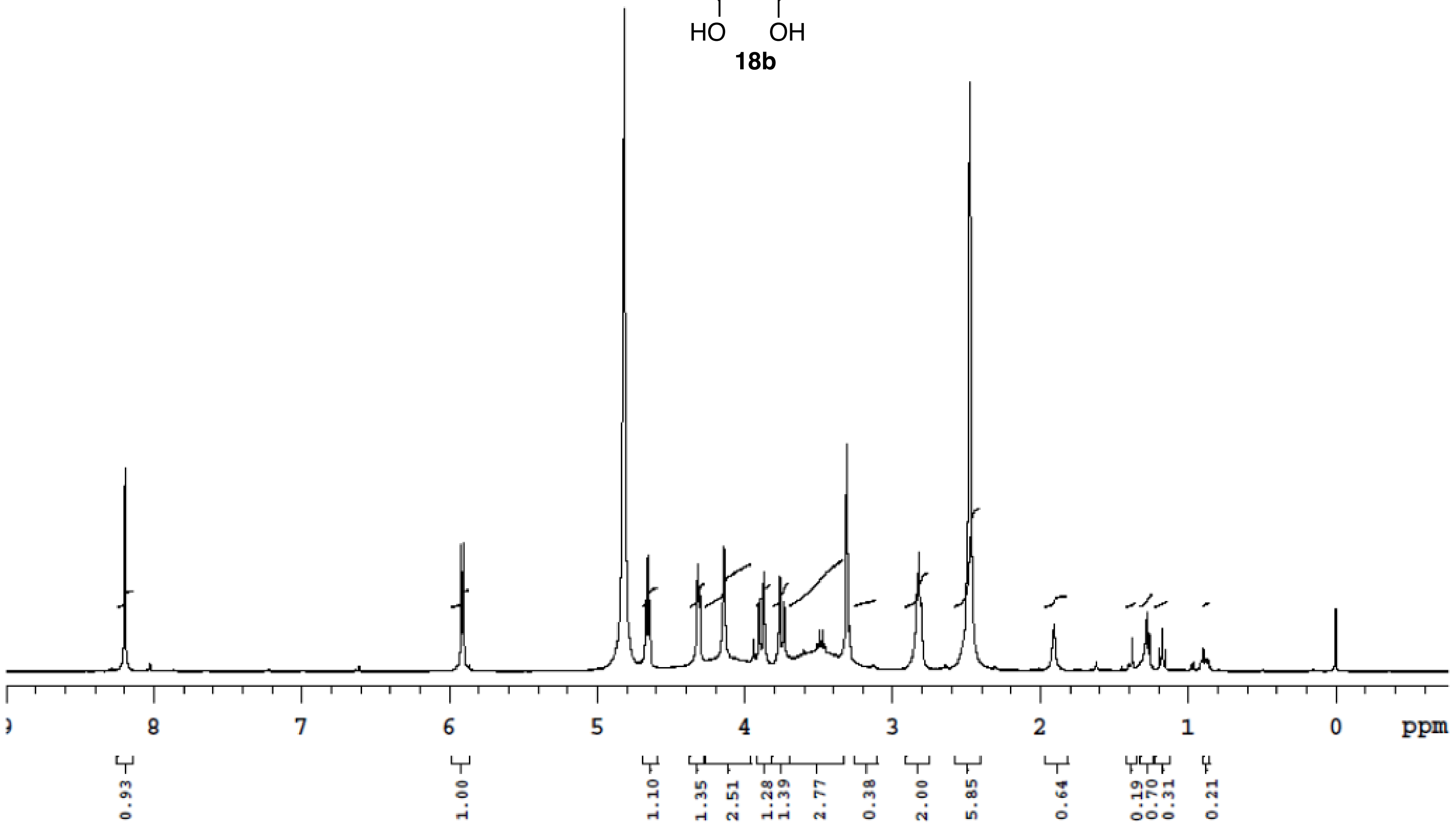

Sample Name :
GVK-DD0397-TME-F
Solvent: cd3od
400-MR/NRM-1/NMR-001
Date: May 10 2015

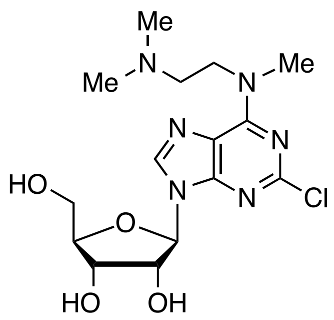

18b

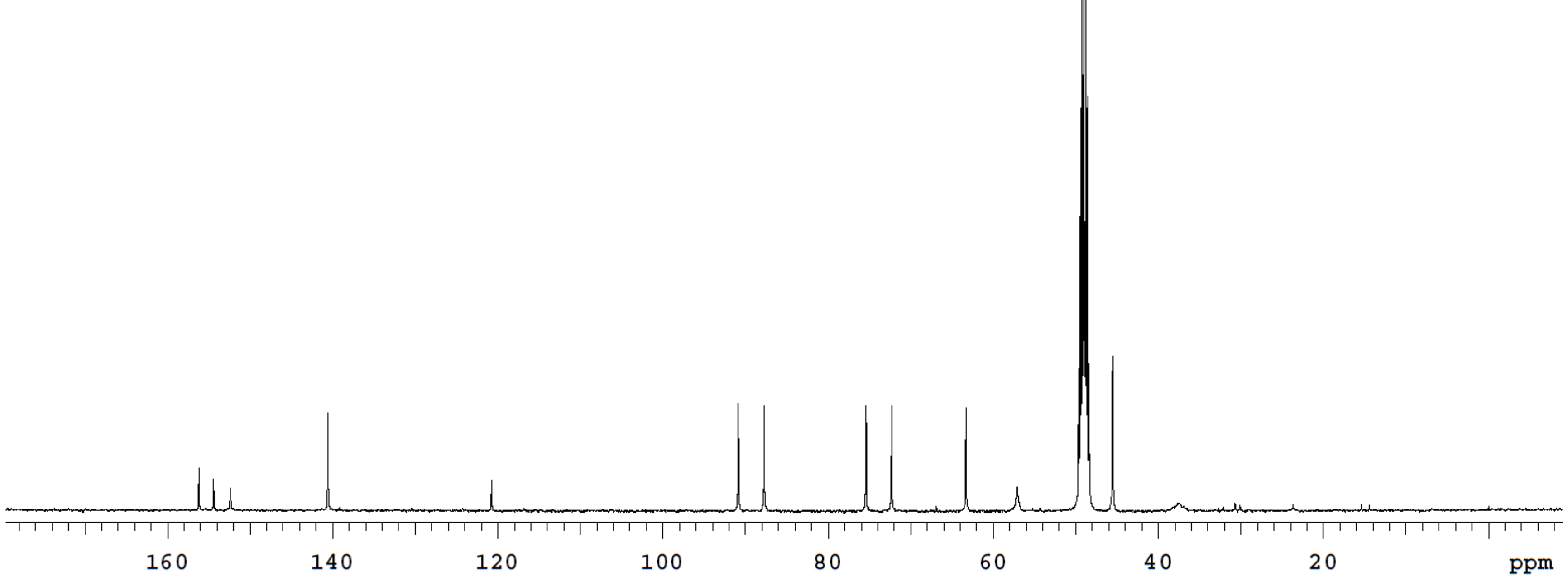

Plotname: 021505A7314_CARBON_01_plot04

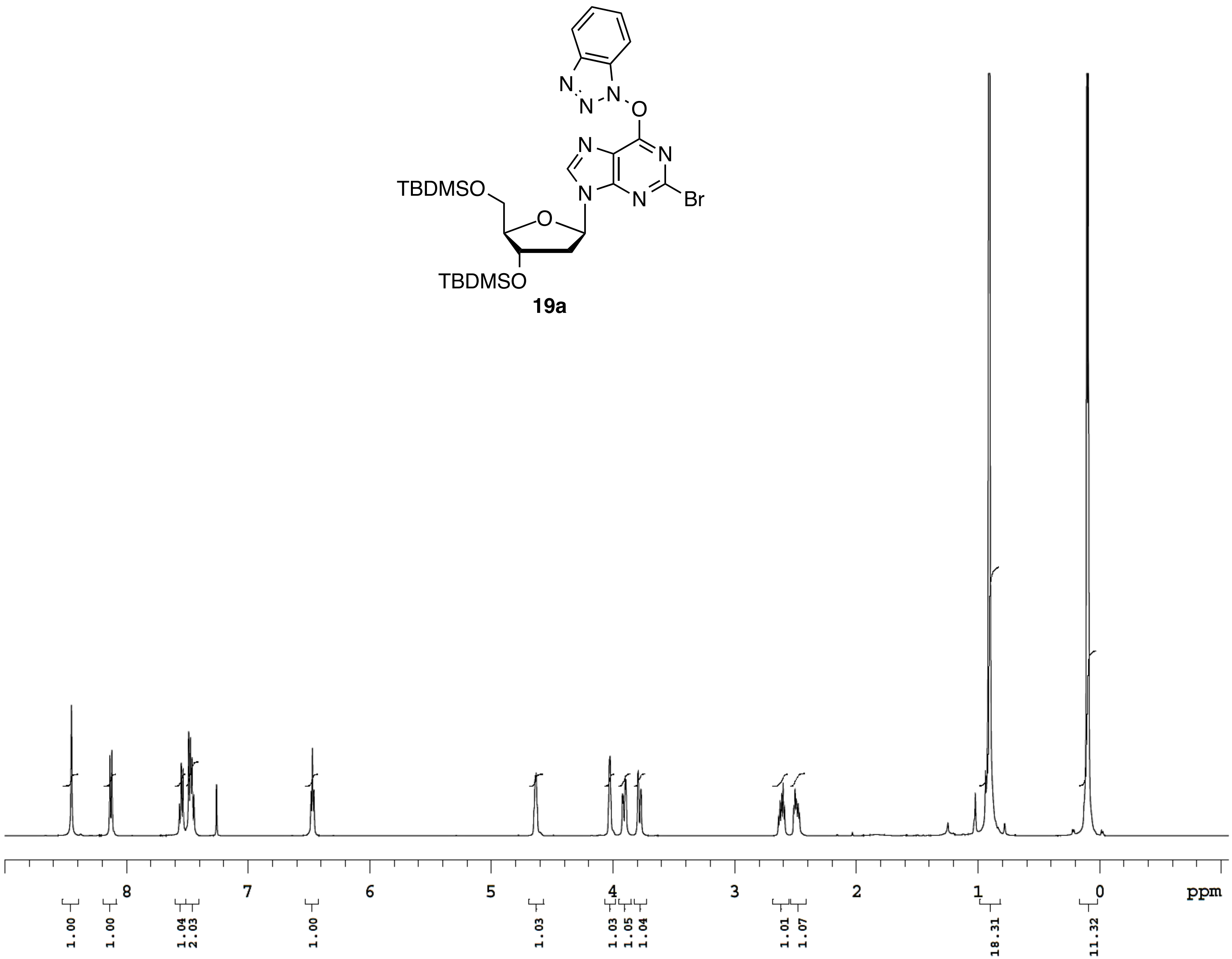
TBDMSO
TBDMSO
N
O
Br
19a
8
7
6
5
4
3
2
1
0
ppm
1.00
1.00
1.04
2.03
1.00
1.03
1.03
1.05
1.04
1.01
1.07
18.31
11.32

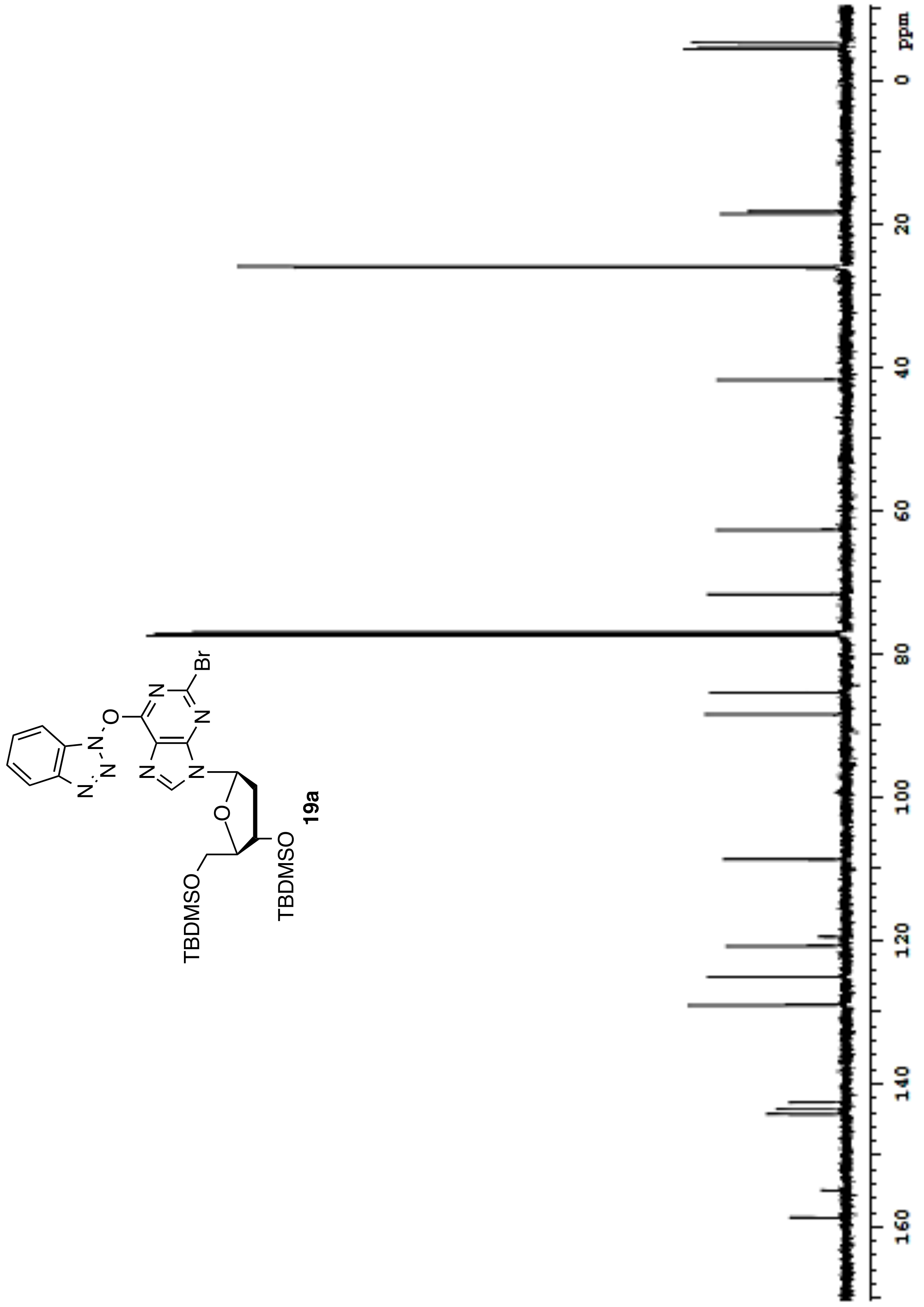
TBDMSO
TBDMSO
Br
19a
ppm
0
20
40
60
80
100
120
140
160

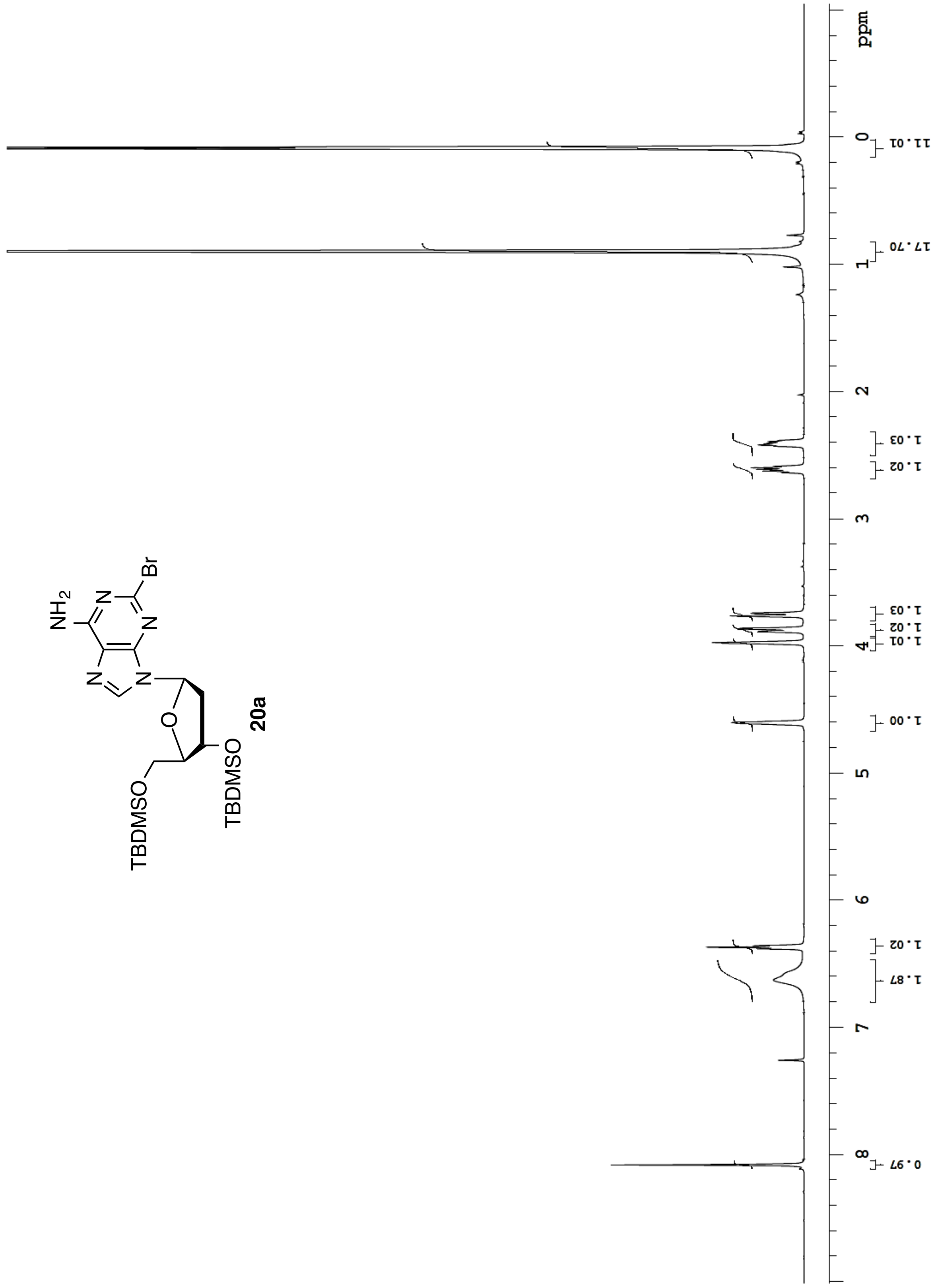
NH$_2$
N
Br
TBDMSO
TBDMSO
O
20a
ppm
0
1
2
3
4
5
6
7
8
11.01
17.70
1.03
1.02
1.03
1.02
1.01
1.00
1.02
1.87
0.97

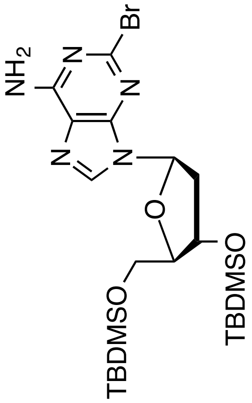

20a

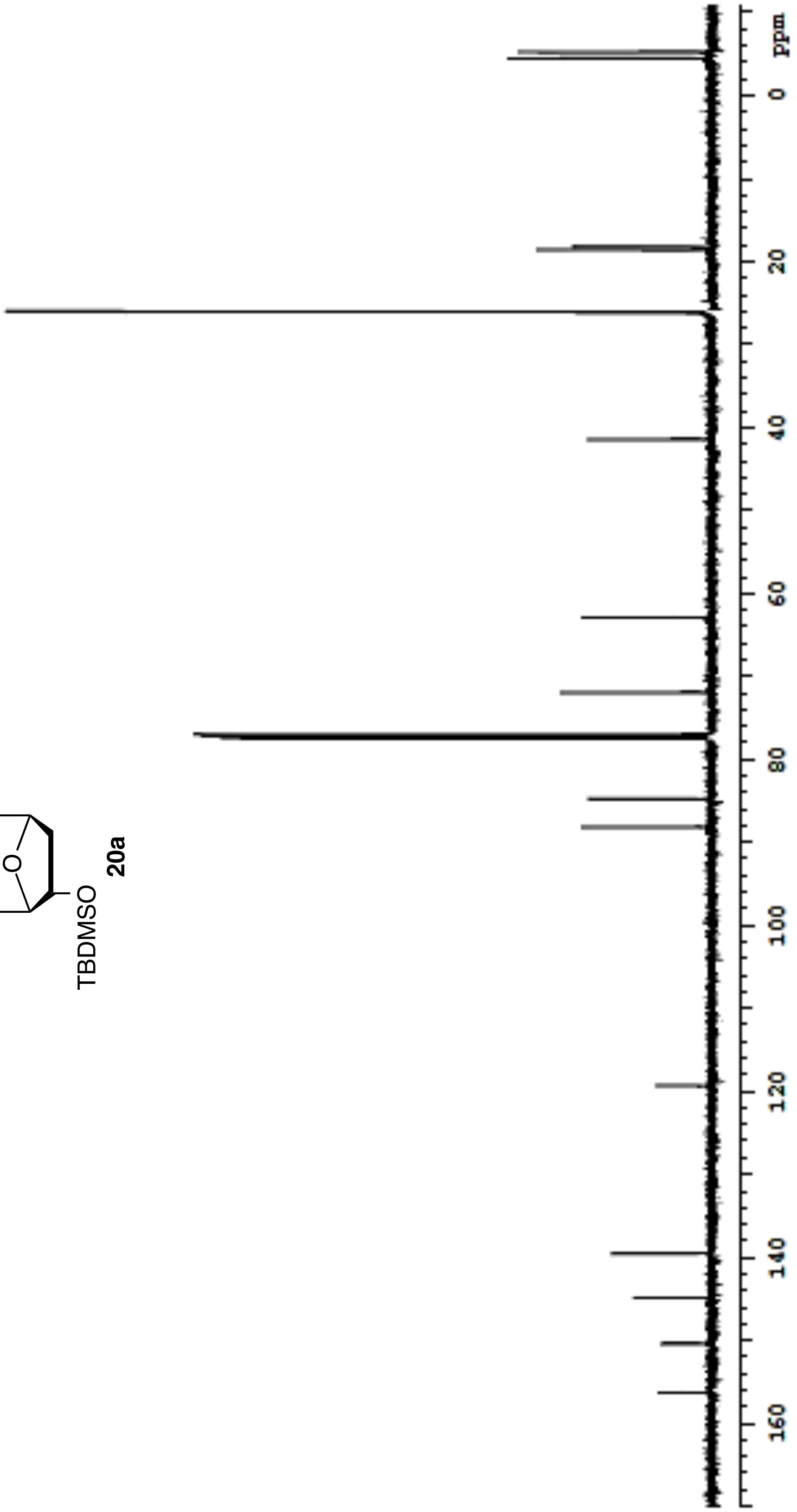

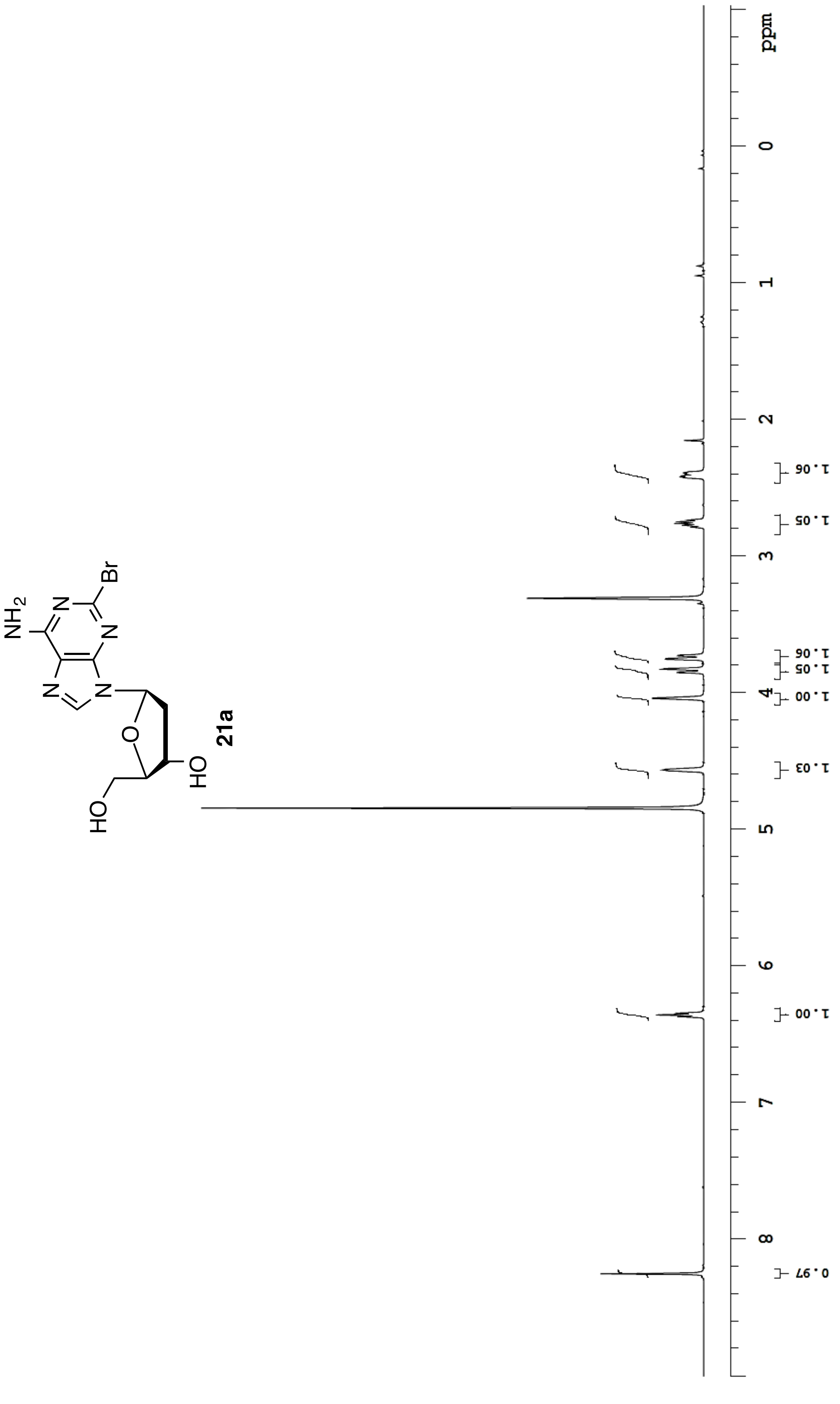
21a
ppm
0
1
2
3
4
5
6
7
8
1.06
1.05
1.06
1.05
1.00
1.03
1.00
0.97

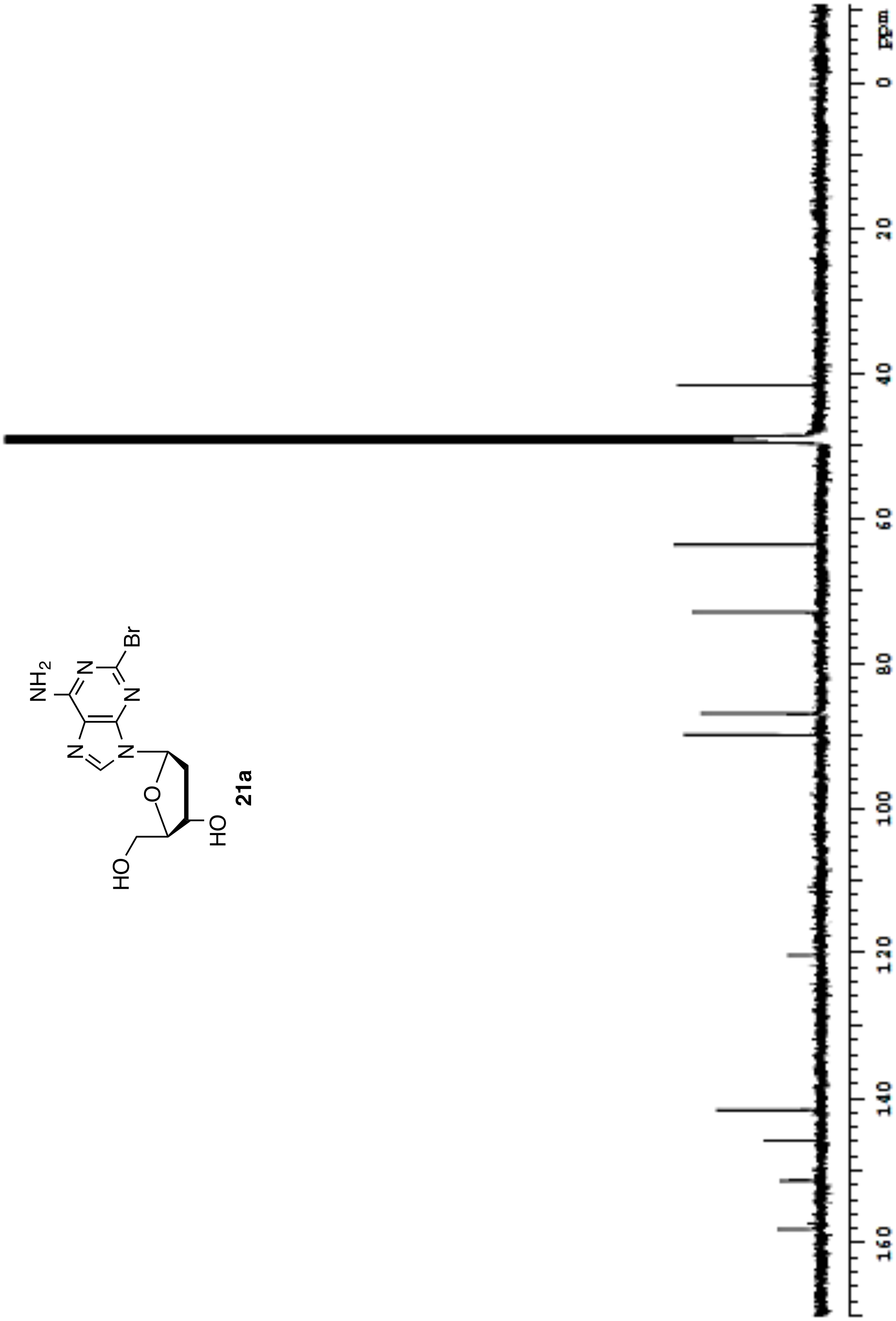

21a

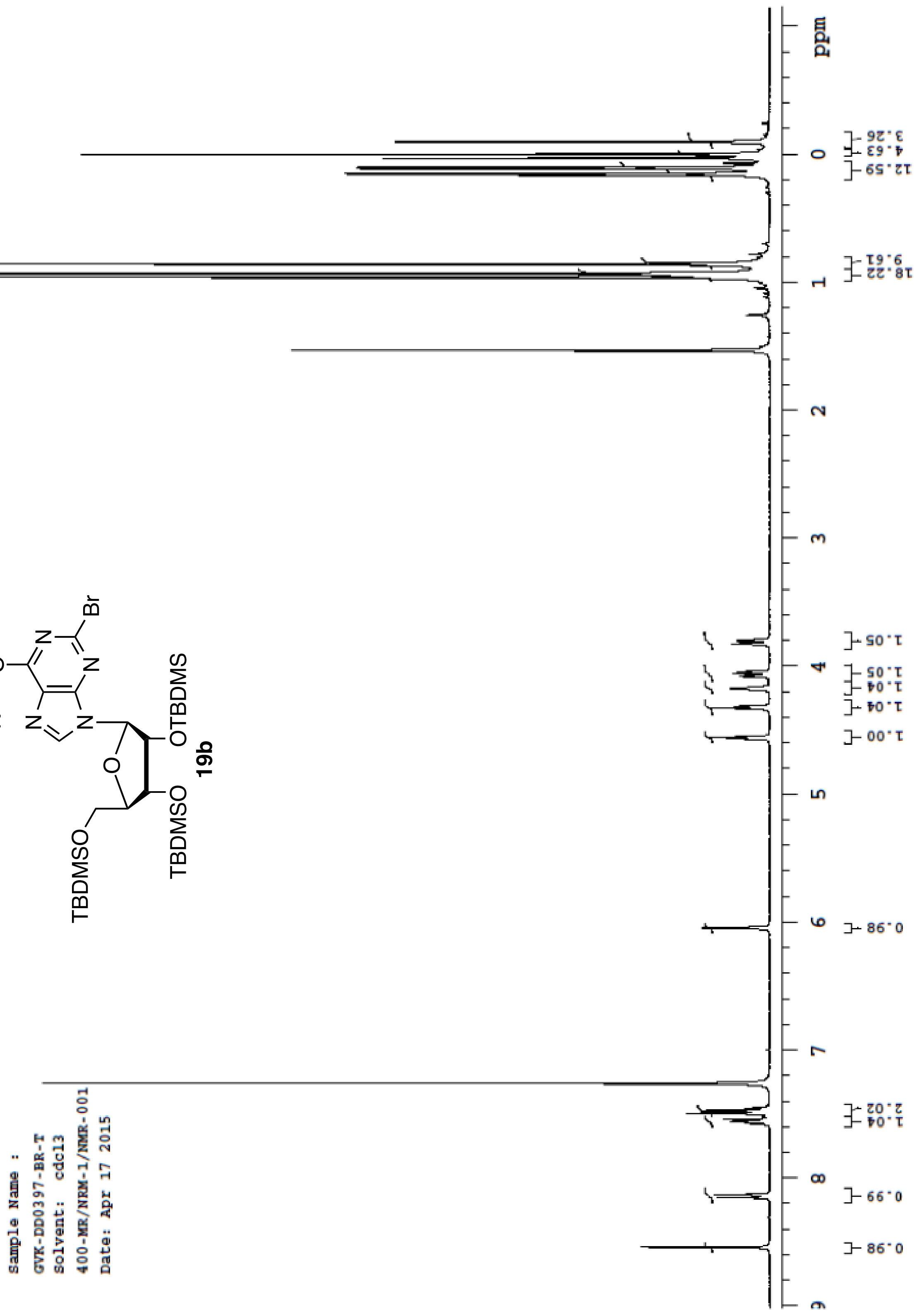
Sample Name :
GVK-DD0397-BR-T
Solvent: cdcl3
400-MR/NRM-1/NMR-001
Date: Apr 17 2015
TBDMSO
TBDMSO
OTBDMS
Br
19b
ppm

Sample Name :
GVK-DD0397-BR-OBT-OTB

Solvent: cdcl3
Date: Apr 22 2015
400 VNMRS/NRM-1/NMR-002

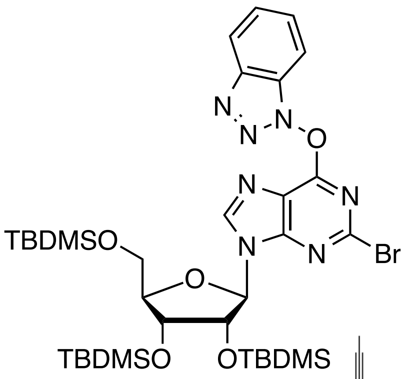

19b

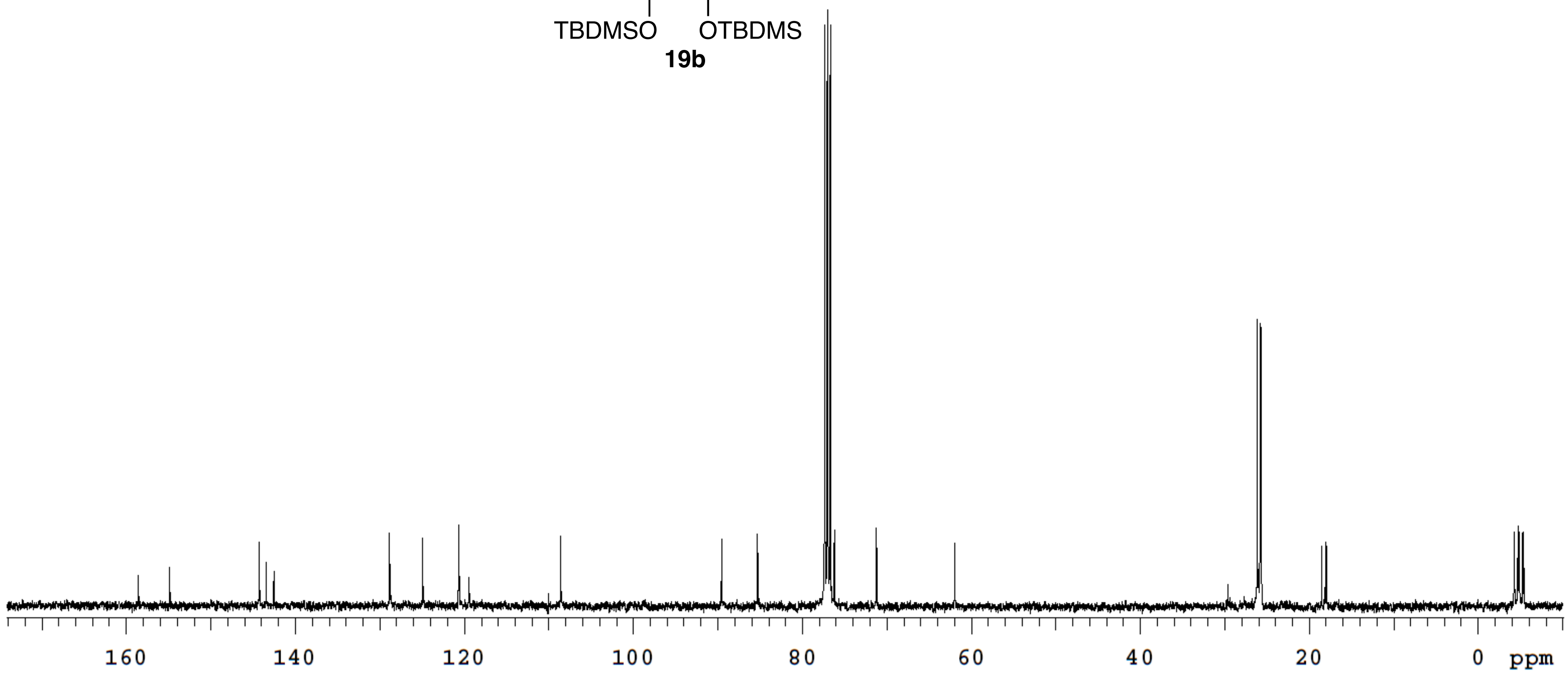

Plotname: 021504C5846_CARBON_01_plot05

Sample Name :
GVK-DD0397-BR-NH2-TBS

Solvent: cdcl3
Date: Apr 22 2015
400 VNMRS/NRM-1/NMR-002

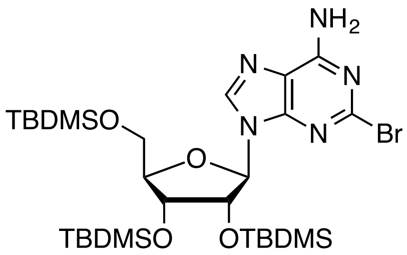

20b

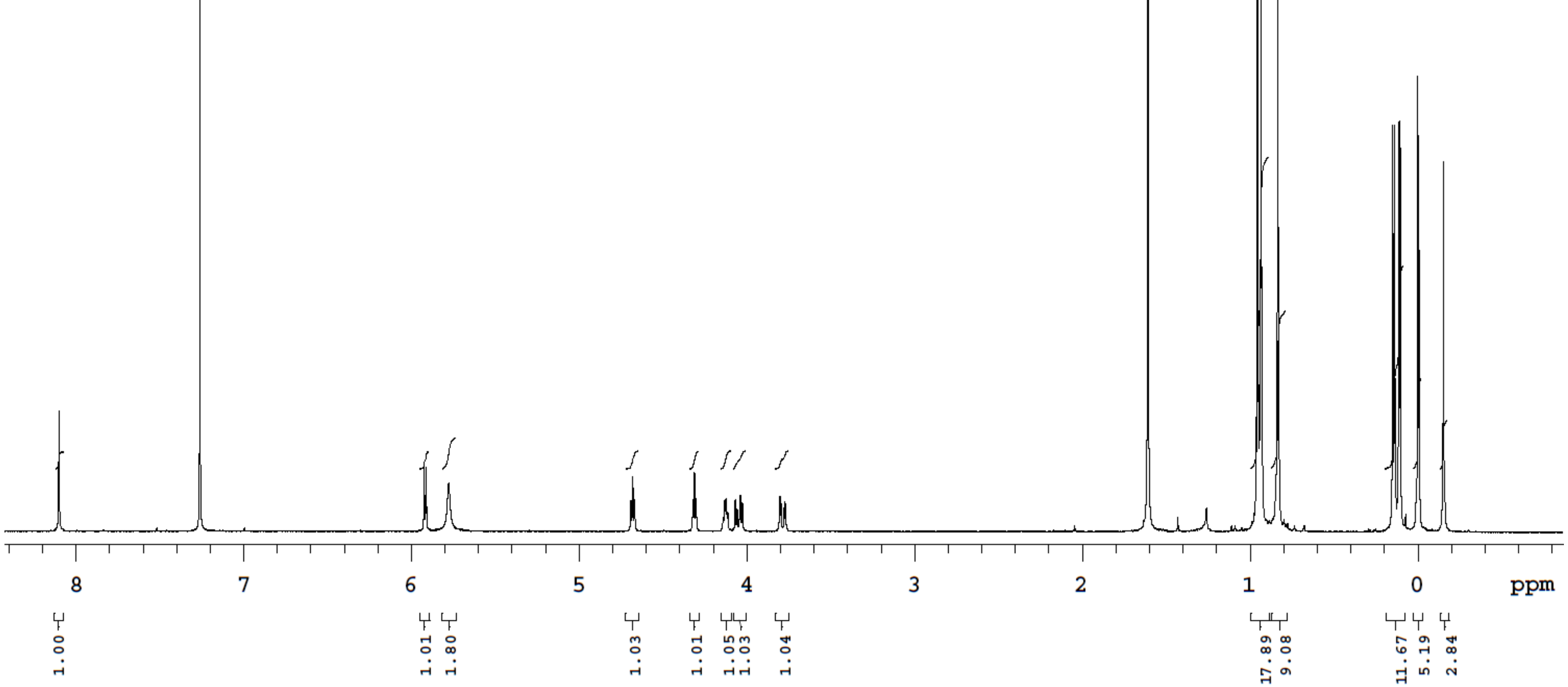

Plotname: 021504C5838_PROTON_01_plot07

Sample Name :
GVK-DD0397-BR-NH2-OTB

Solvent: cdcl3
VNMRS-400/NRM-1/NMR-002
Date: Apr 22 2015

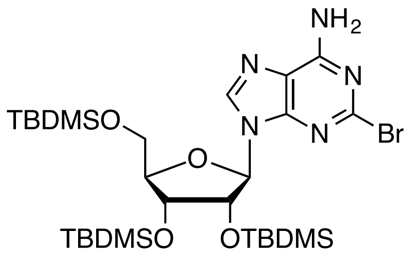

20b

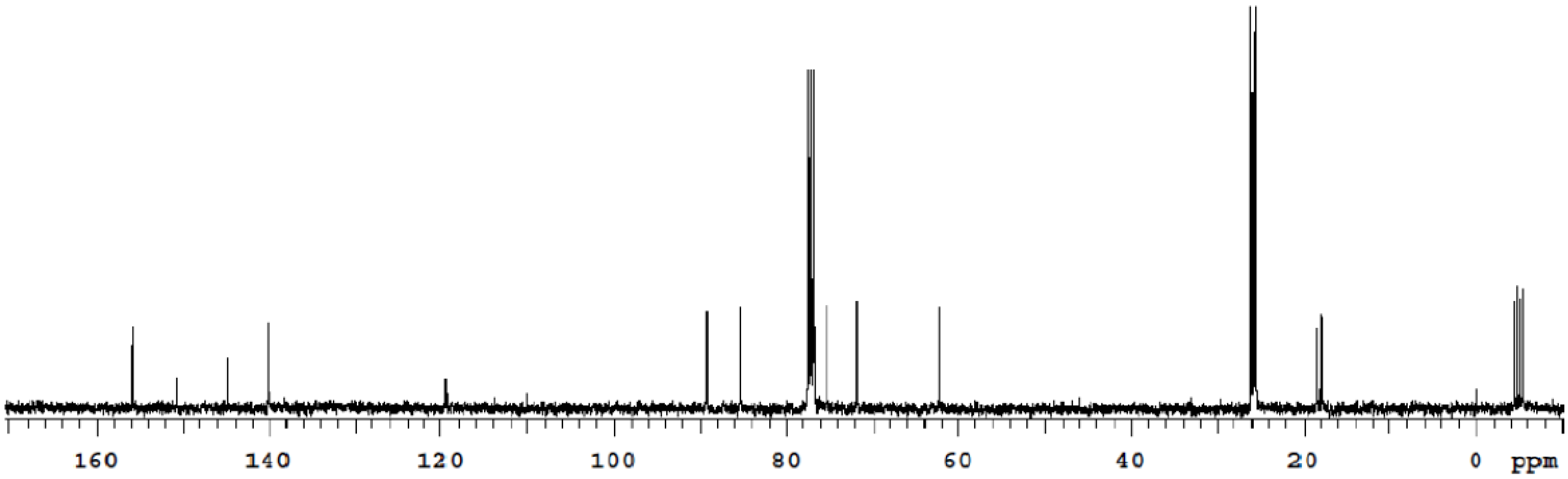

Sample Name :
GVI-DD0397-BR-NH2-OH

Solvent: cd3od
Date: Apr 20 2015
400 VNMRS/NRM-1/NMR-002

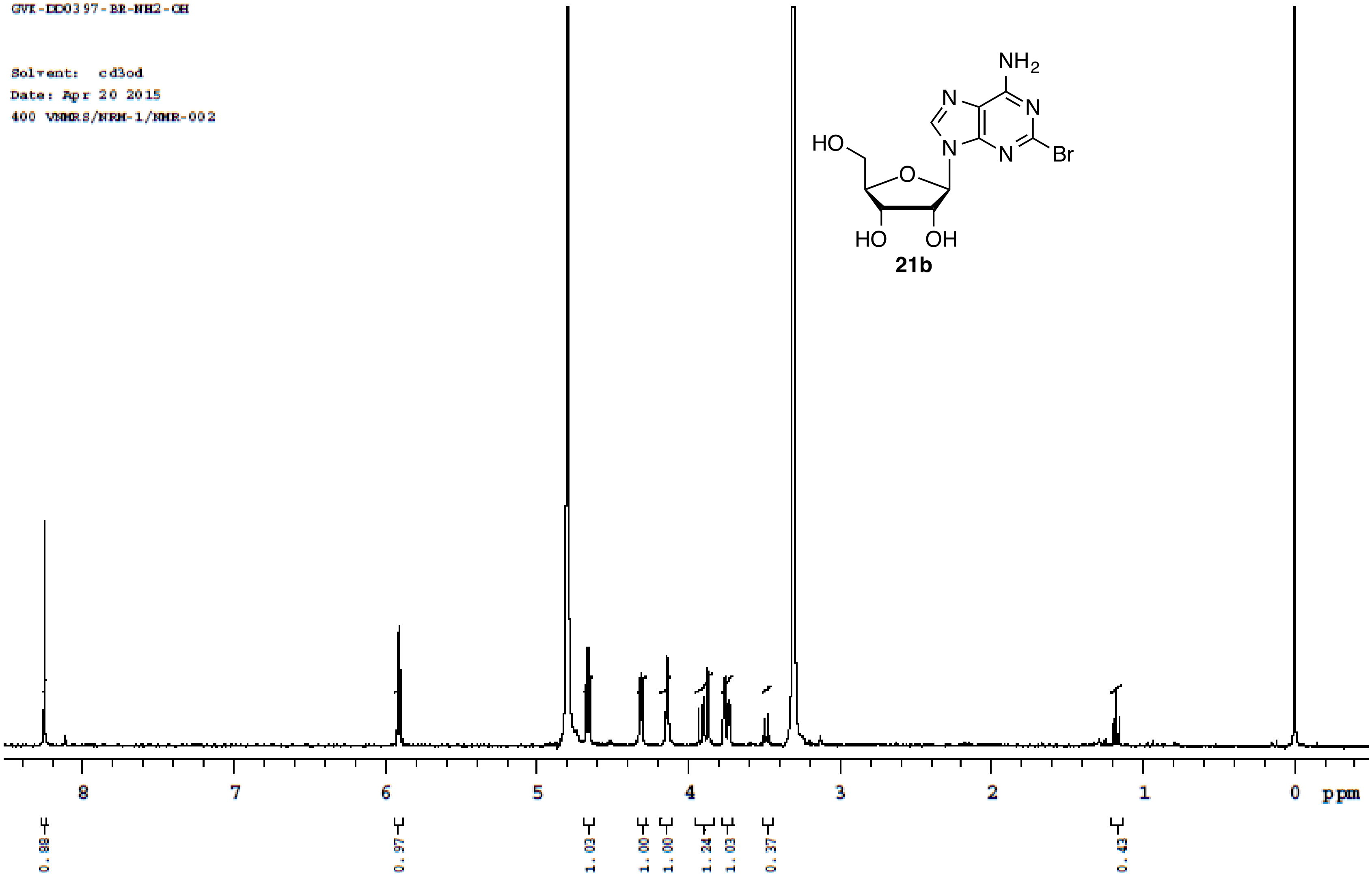

Plotname: 021504C3615_PROTON_01_plot10

Sample Name :
GVK-DD0397-BR-NH2-OH

Solvent: dmso
VNMRS-400/NRM-1/NMR-002
Date: Apr 22 2015

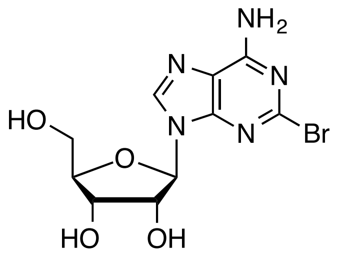

21b

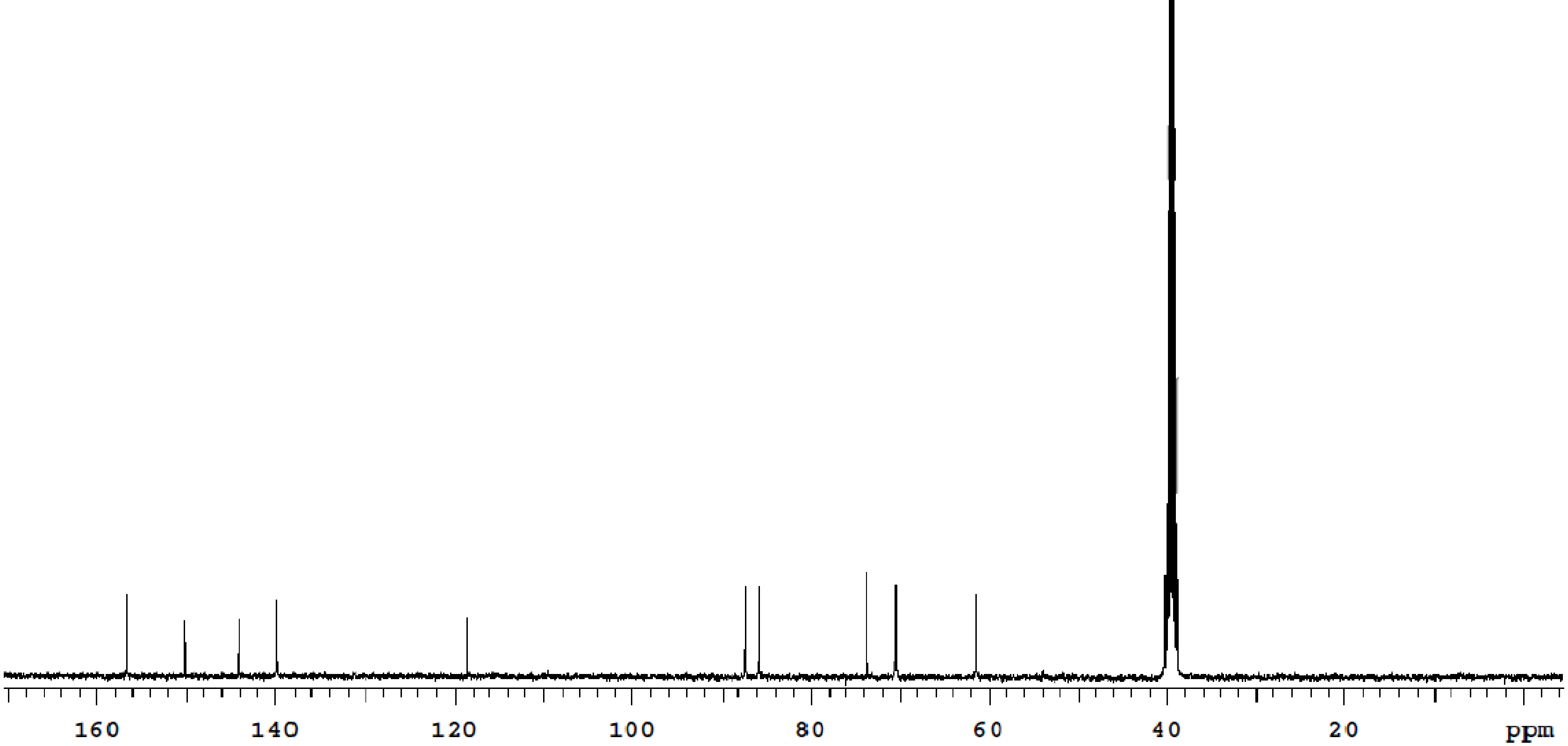

Supplement: Supplementary file 1 [file NIHMS775727-supplement-SI_Final_.pdf]
